# Publisher Correction: Discovery of coding regions in the human genome by integrated proteogenomics analysis workflow

**DOI:** 10.1038/s41467-018-04279-5

**Published:** 2018-05-08

**Authors:** Yafeng Zhu, Lukas M. Orre, Henrik J. Johansson, Mikael Huss, Jorrit Boekel, Mattias Vesterlund, Alejandro Fernandez-Woodbridge, Rui M. M. Branca, Janne Lehtiö

**Affiliations:** 1 Department of Oncology-Pathology, Science for Life Laboratory, Karolinska Institutet, Tomtebodavägen 23A, 171 65 Stockholm, Sweden; 2 Department of Biochemistry and Biophysics, The Arrhenius Laboratories for Natural Sciences, Science for Life Laboratory, Stockholm University, Tomtebodavägen 23A, 171 65 Stockholm, Sweden; 3 Department of Oncology-Pathology, NBIS (National Bioinformatics Infrastructure Sweden), Science for Life Laboratory, Karolinska Institutet, Tomtebodavägen 23A, 171 65 Stockholm, Sweden

Correction to: *Nature Communications* 10.1038/s41467-018-03311-y, published online 02 March 2018

In the original version of this Article, extraneous text not belonging to the Article was accidentally appended to the “Results” section. This error has now been corrected in both the PDF and HTML versions of the Article.

