# Supplementary material for: Discovery of coding regions in the human genome by integrated proteogenomics analysis workflow
Source: Nat Commun. 2018 Mar 2;9:903. doi: 10.1038/s41467-018-03311-y (PMC5834625; doi:10.1038/s41467-018-03311-y)
Supplement: Supplementary file 1 — Supplementary Information [file 41467_2018_3311_MOESM1_ESM.pdf]

Discovery of coding regions in the human genome by Integrated Proteogenomics Analysis Workflow

Zhu et al.

Supplementary Information

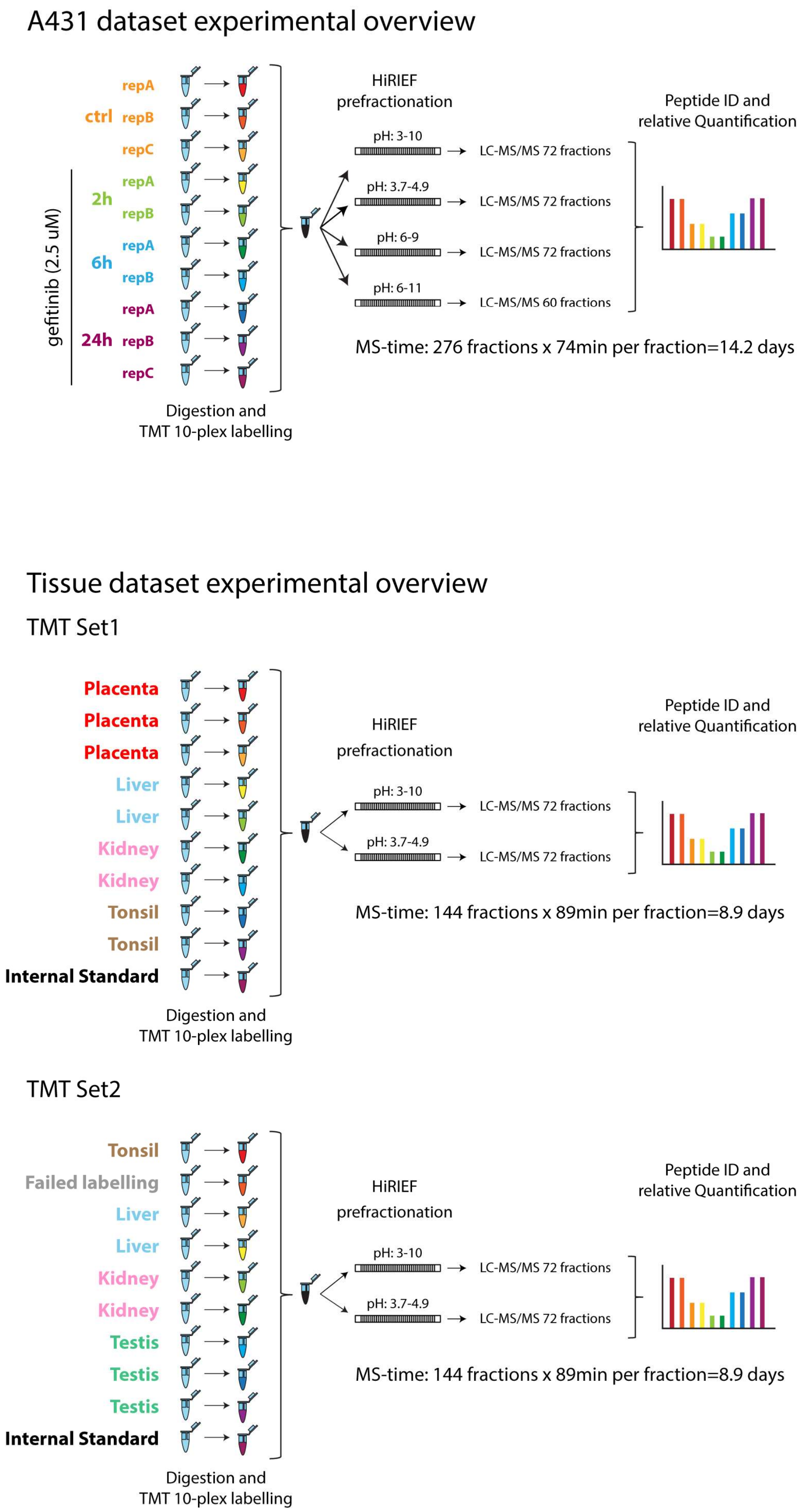

Supplementary Figure 1. Experimental overview of A431 and normal tissues datasets. One A431 TMT set and two normal tissues TMT sets were prepared and subsequently aliquoted for HiRIEF separation using multiple IPG strips of different pH ranges.

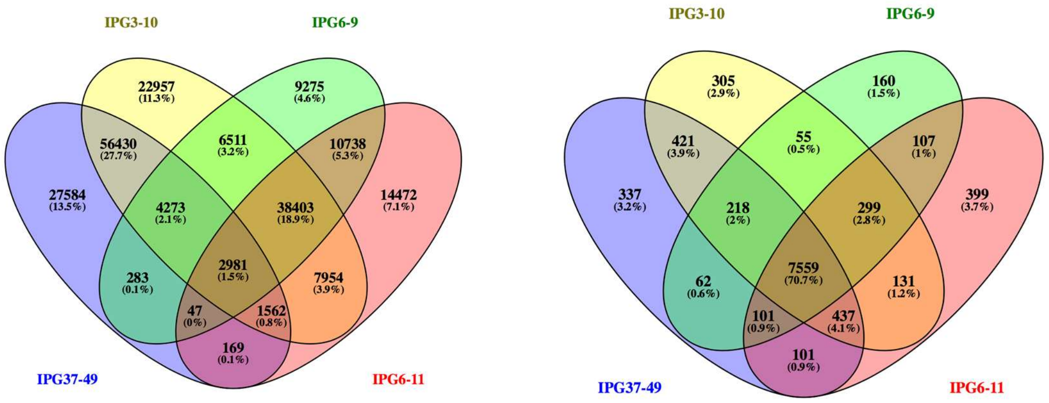

Supplementary Figure 2. Overlap of peptides (left) and gene protein products (right) identified from different IPG ranges in the A431 dataset. The peptides were filtered by 1% FDR for each individual plate. Gene protein products were filtered by 1% protein level FDR (using the "picked protein FDR" method by Savitski *et al*[1]).

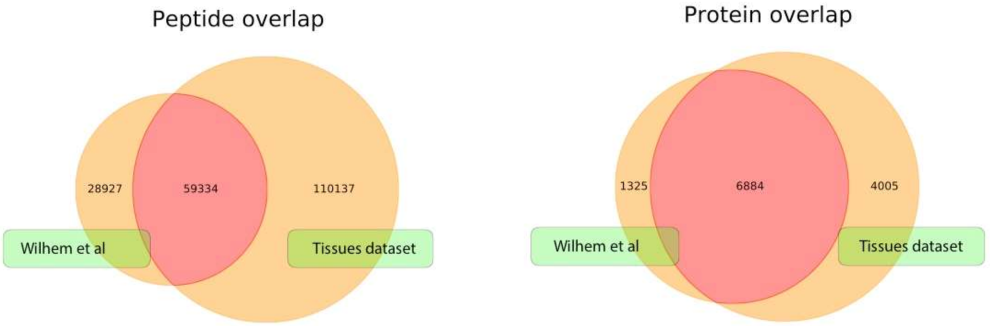

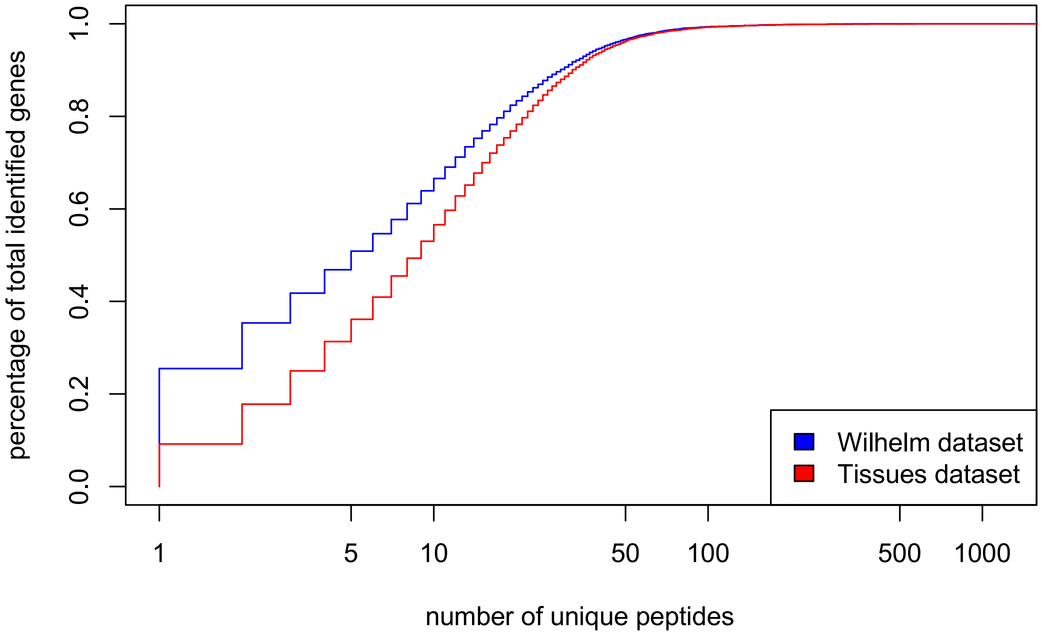

Supplementary Figure 3. Comparison of number of identified peptides and proteins (top) and number of unique peptides per protein (bottom) in the "normal tissues" dataset and the corresponding tissues in the Wilhelm *et al* publication. We downloaded the MS raw files corresponding to the five normal tissues (kidney, liver, tonsil, placenta, and testis) from the Wilhelm *et al*[2] draft proteome publication, and searched them in the same pipeline as used for our MS data. The data in Wilhelm dataset comes from a total of 5 samples (one per tissue type) that were run in label free mode for a total MS acquisition time of 7 days. Our data comes from 17 samples (each one from a different individual) which were pooled into two TMT sets run on LCMS for a total acquisition time of 16 days. All peptides and proteins reported are at 1% FDR (peptide and protein level). The bottom figure shows the cumulative fraction of proteins identified ranked by their respective number of unique peptide identifications. About 25% of all proteins identified in the Wilhelm dataset had only a single peptide identified whereas only 10% of all proteins identified in our "normal tissues" dataset were single-peptide identifications.

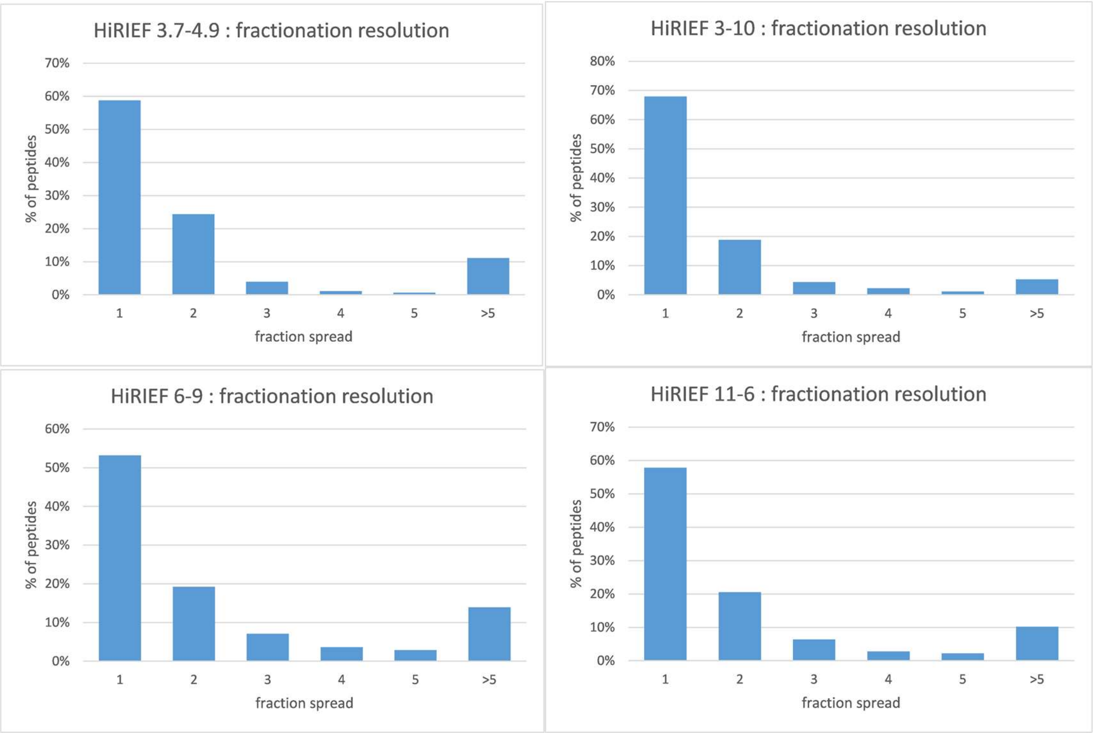

Supplementary Figure 4. Evaluation of HiRIEF focusing sharpness, or pI based fractionation resolution for different IPG ranges. Histogram of fraction spreads of all identified peptides in each IPG range. The fraction spread of each peptide is calculated as: number of highest fraction wherein a peptide is present – number of lowest fraction where the same peptide is present + 1. A peptide is considered present in a fraction if the MS1 area of the peptide in that fraction is at least 1% of the maximal MS1 area of that peptide in any fraction.

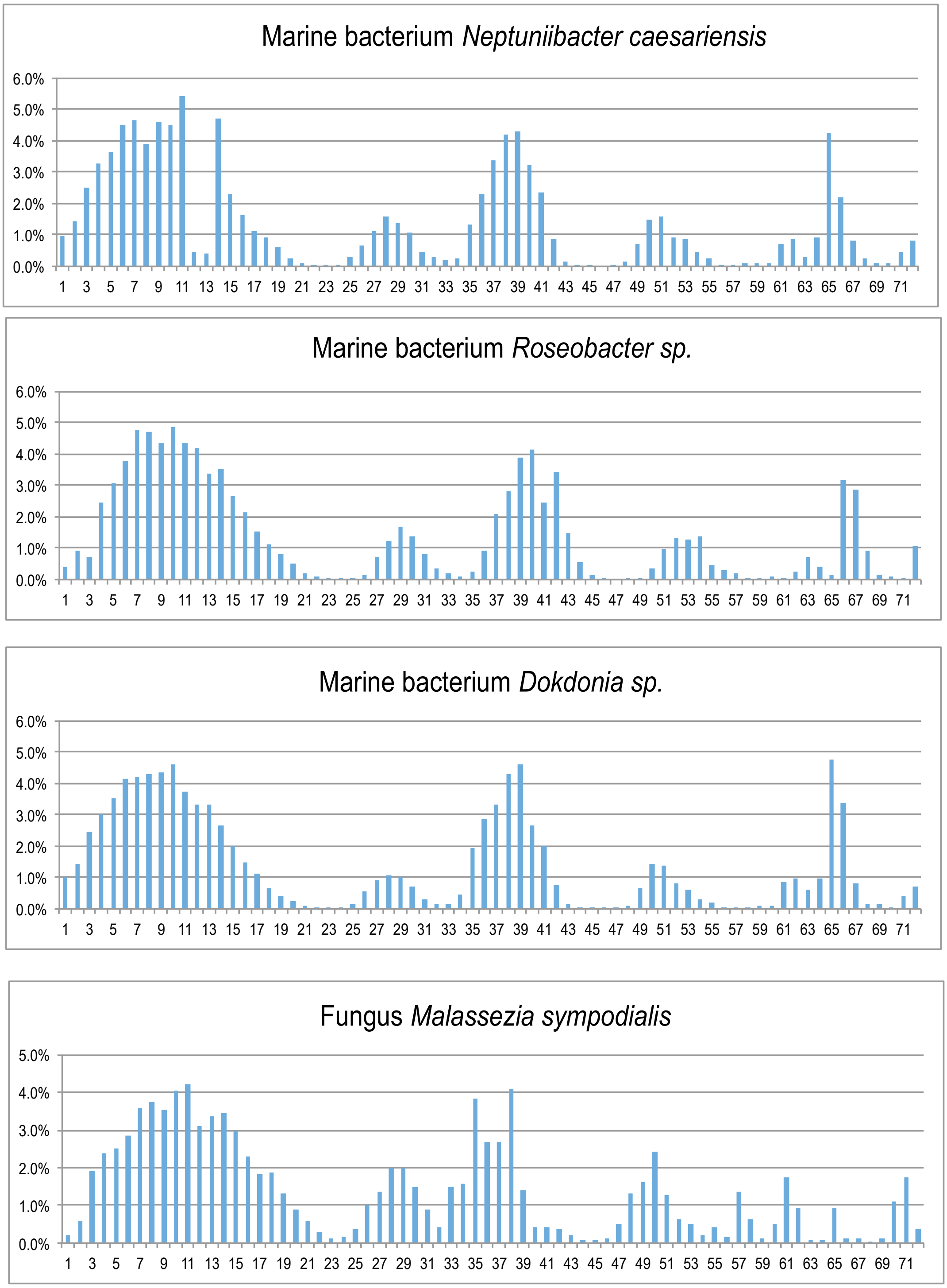

Supplementary Figure 5. Distribution of unique peptides over the 72 fractions of the IPG3-10 HiRIEF strip in different species. The percentages (of all unique peptides identified) contributed by each IPG3-10 fraction (numbered on the X-axis) are plotted. IPG3-10 strips have a linear pH gradient. For *Neptuniibacter caesariensis* (top), the drop of peptide yield at fractions 12 and 13 was due to poor extraction from the IPG strip to the 96 well plate. The distribution of unique peptides over the pI range appears to be universal across the different species analyzed. Marine bacteria datasets are from Muthusamy *et al*[3] and the fungus dataset is from Zhu Y *et al*[4].

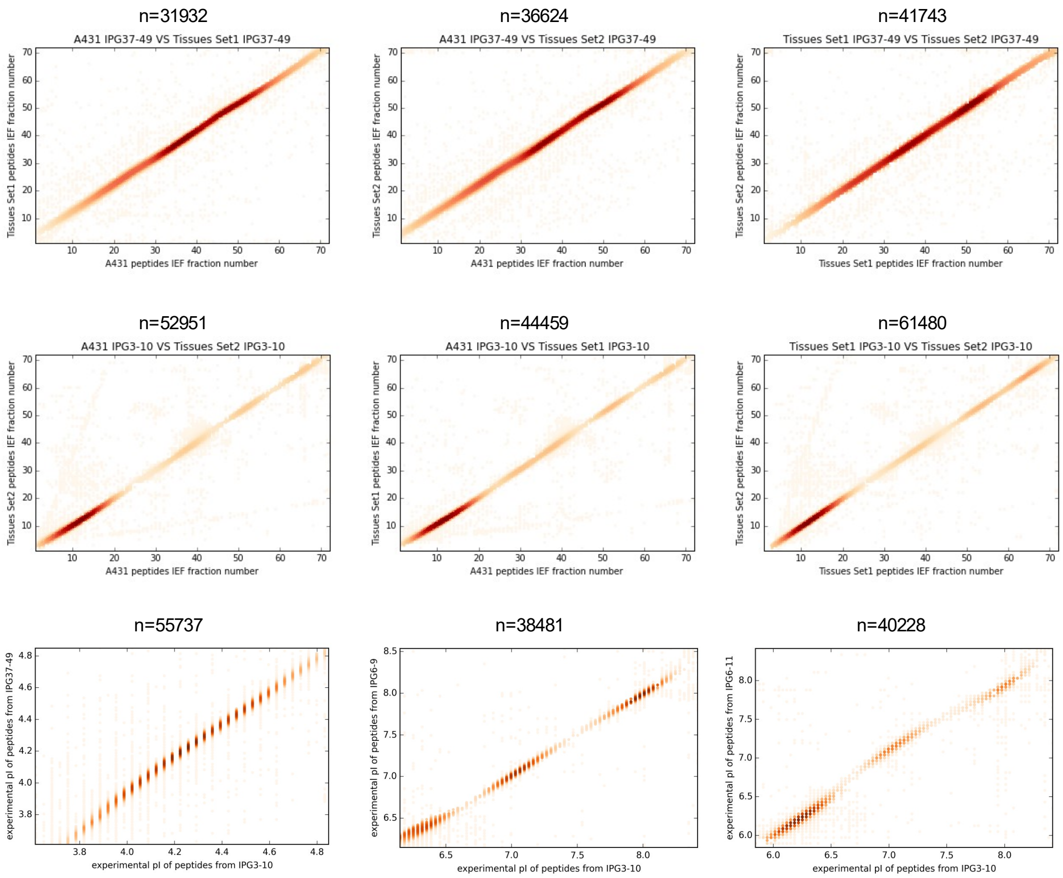

Supplementary Figure 6. Reproducibility of HiRIEF based fractionation. In the top two rows, the focusing fraction of each peptide is compared between different samples for the same IPG range. Only peptides that focused in one or at most two consecutive fractions were plotted. In the bottom row, different IPG ranges are compared for the same sample. Experimental pI values were calculated based on linear equations obtained by calibrations with fluorescently labelled pI markers (see Methods).

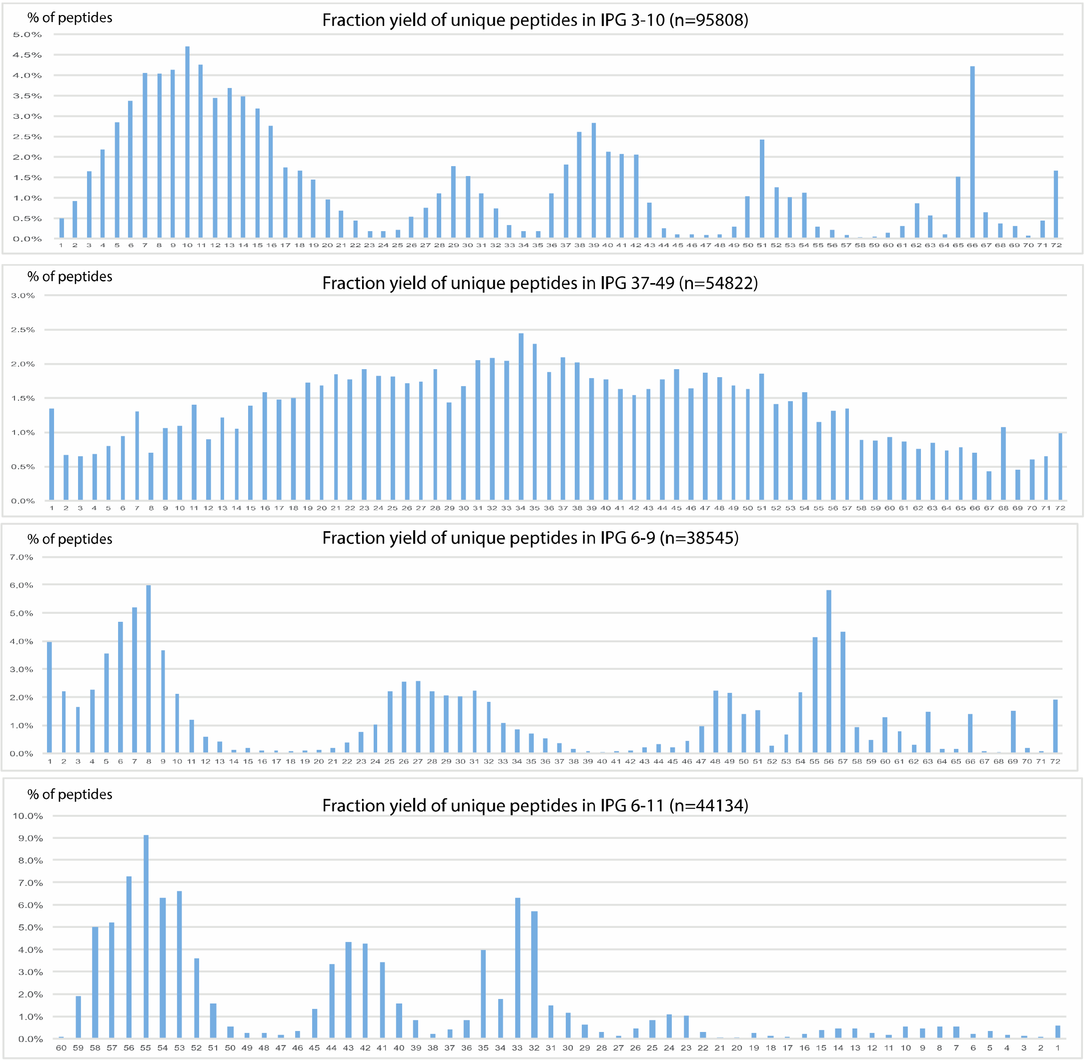

Supplementary Figure 7. Fraction specific yield of unique peptides in different IPG strips. The distribution of unique peptides per IPG fraction in each HiRIEF range employed to analyze the A431 cells is shown. Only peptides focusing in a single fraction are plotted. Y-axis is the percentage of unique peptides identified from each fraction. X-axis is the fraction number in the order of low to high pI values.

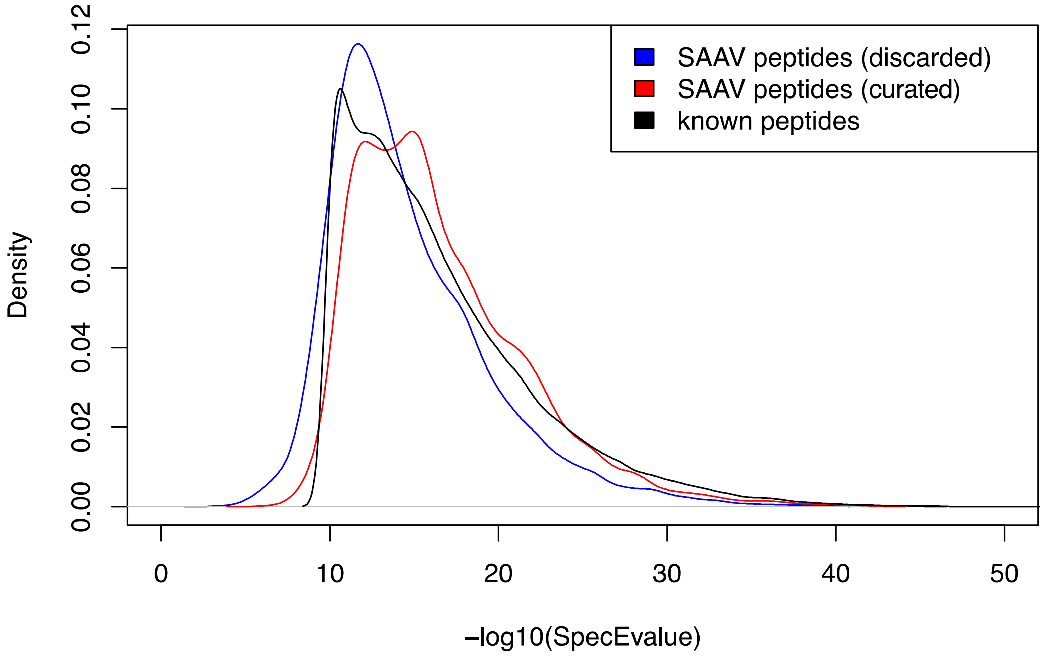

Supplementary Figure 8. Search engine score (SpecEvalue) distribution of single amino acid variant peptides compared to known peptides. The SAAV peptides shown here were identified from A431 data at 1% class specific FDR at the *discovery* stage. The score distributions of discarded and curated SAAV peptides (by SpectrumAI) are shown as blue and red curves, respectively. Known peptides plotted here are unique peptides identified from the standard proteomics database search at 1% peptide level FDR.

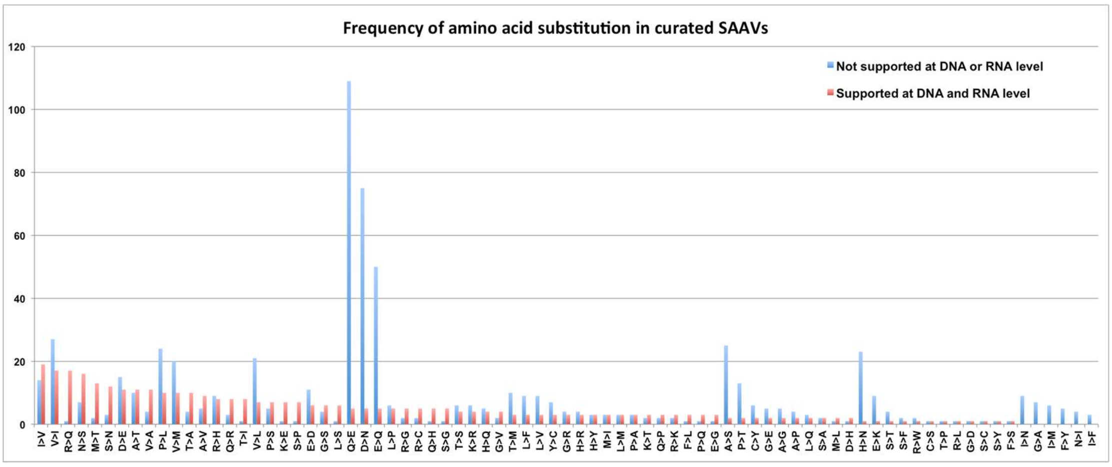

Supplementary Figure 9. Frequency of amino acid substitution in curated SAAVs identified in A431 dataset. The SAAVs plotted here were curated by SpectrumAI. Substitutions supported both by DNA and RNA sequencing shown as red bars; substitutions with no evidence from either DNA or RNA sequencing shown as blue bars. Several types of amino acid substitutions, particularly Q>E, D>N, and E>Q, were over-represented in SAAVs without evidence in the sequencing data. Some of these enrichments could be explained by chemical artifacts occurring during sample preparation. For example, Q>E could be explained by deamidation.

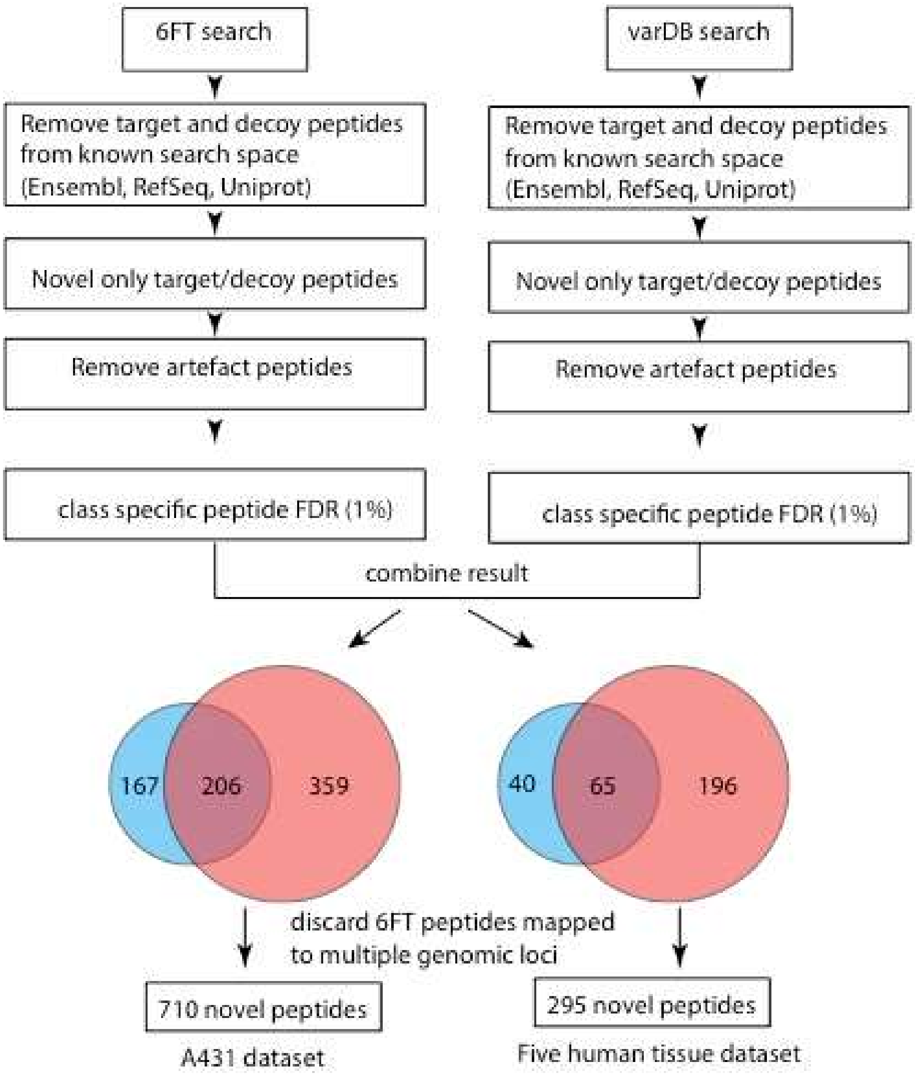

Supplementary Figure 10. Combined proteogenomics results of 6FT and VarDB searches in "A431 cells" and "normal tissues". All novel peptides displayed were filtered applying a 1% class-specific FDR according to Nesvizhskii[5].

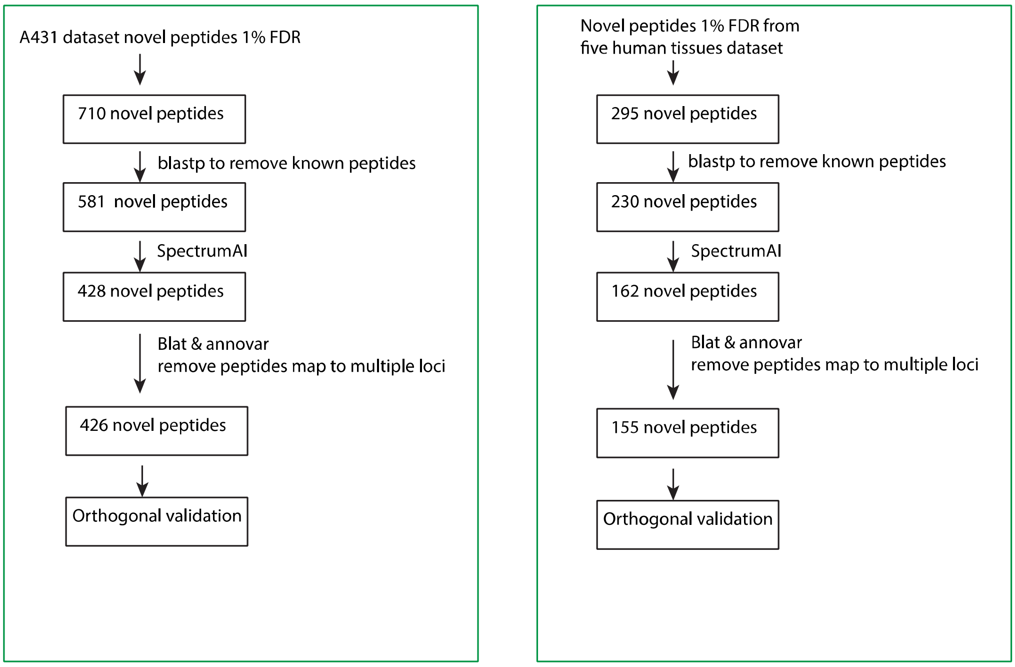

Supplementary Figure 11. Curation stage results of the proteogenomics pipeline for A431 cells and normal tissues dataset. The number of novel peptide candidates passing each curation step is shown. SpectrumAI only inspects novel peptides that possess a single amino acid substitution compared a known peptide sequence.

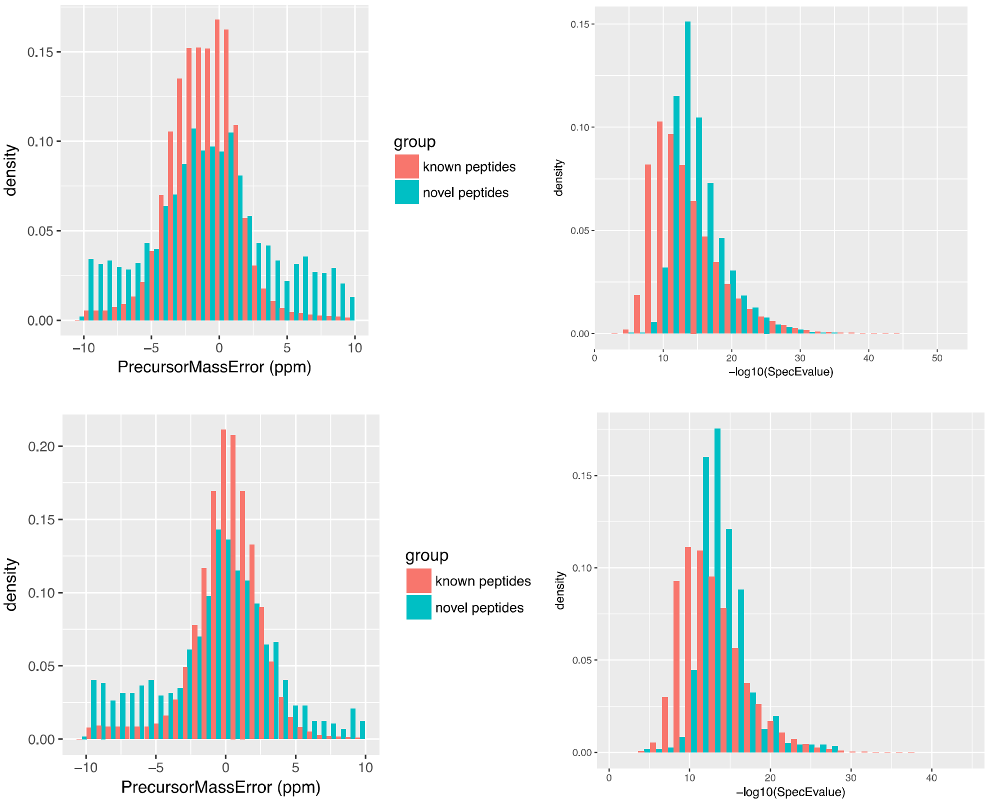

Supplementary Figure 12. Precursor mass error and score distribution of novel peptides that passed curation stage from A431 cells (top) and normal tissues (bottom) datasets. Search engine scores (such as SpecEvalue) are typically better for novel peptides than for known peptides, a natural consequence of the search space size influence, and therefore this parameter is of limited use for curation of candidate novel peptides post class-specific FDR cut. The shoulders present in the precursor mass error distribution of novel peptides suggest that some false discoveries still survived the curation steps. Novel peptides are from the proteogenomics searches whereas known peptides are from the standard proteomics searches.

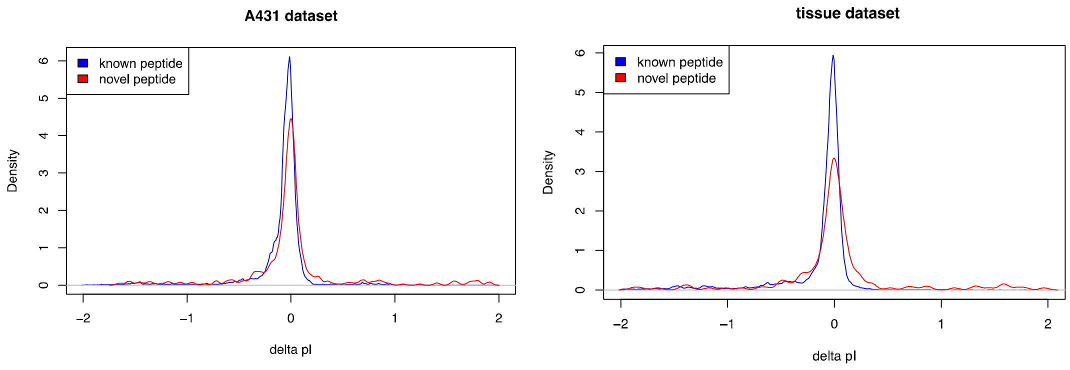

Supplementary Figure 13. Delta pI distribution of novel peptides identified from A431 and normal tissues dataset. Delta pI was calculated as the difference between experimental pI and theoretical pI. Novel peptides are from the proteogenomics searches whereas known peptides are from the standard proteomics searches.

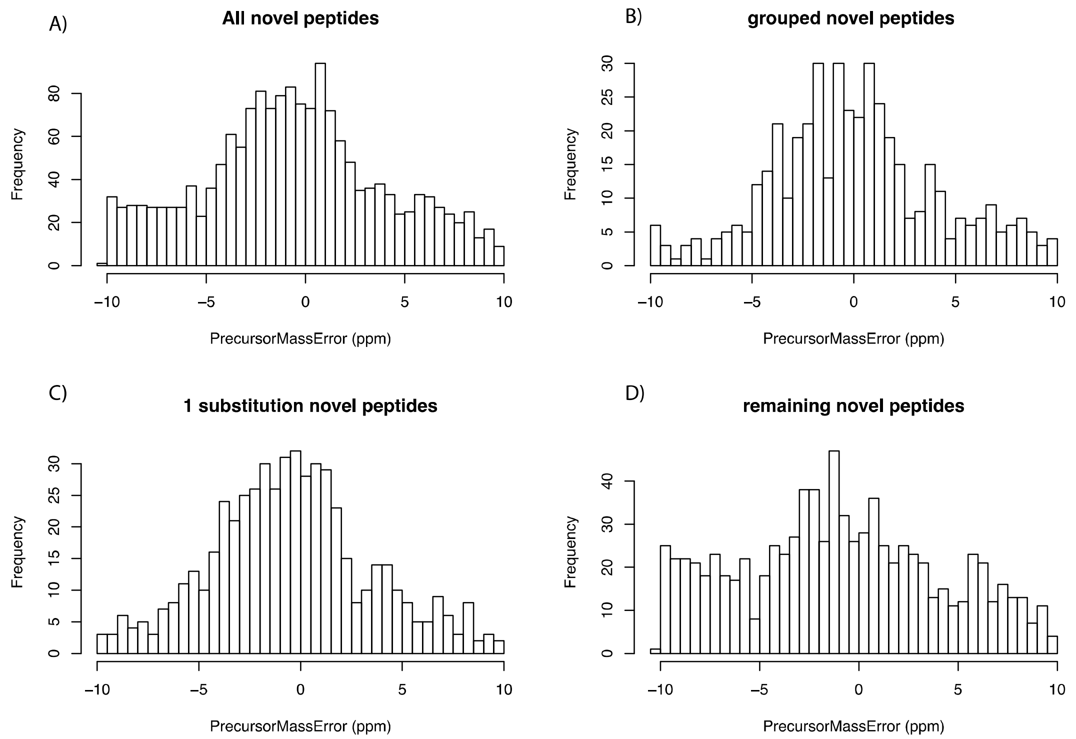

Supplementary Figure 14. Precursor mass error distributions of different populations of curated novel peptides. a) All novel peptides that passed curation stage. **b)** Novel peptides with neighboring peptides within 10 kb. **c)** Novel peptides with one amino acid substitution compared to a known peptide. **d)** Remaining novel peptides that don't belong to **b)** or **c)**.

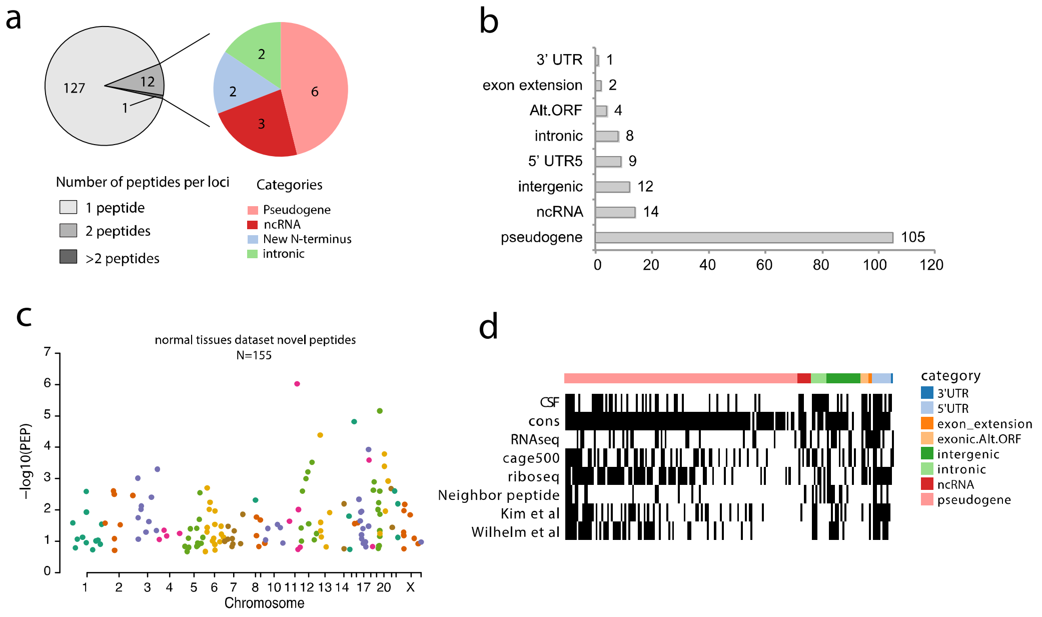

Supplementary Figure 15. Novel protein-coding loci findings in normal tissues dataset. a) The left pie chart shows the number of novel protein-coding loci supported by one, two or more peptides (peptides within 10kb distance were grouped into one loci); the right pie chart shows the different types of novel coding events supported by multiple peptides. **b)** An automatic categorization of novel peptides based on Refseq gene annotation. **c)** Chromosome Manhattan plot of novel peptides. y-axis represents the posterior error probability (PEP) in -log10 scale. **d)** Orthogonal data support for novel peptides including conservation analysis, PhyloCSF coding potential, A431 cell line RNA-seq reads evidence, Ribosome profiling, CAGE (up to 500 bp upstream from peptide location), presence of peptides in draft proteome studies of Kim *et al*[6] and Wilhelm *et al*[2]. Continuous data was discretized into binary value 0 or 1 for visualization purposes.

Gene: CTD-2620I22.3; ENSG00000267943; Q6ZRZ8_HUMAN

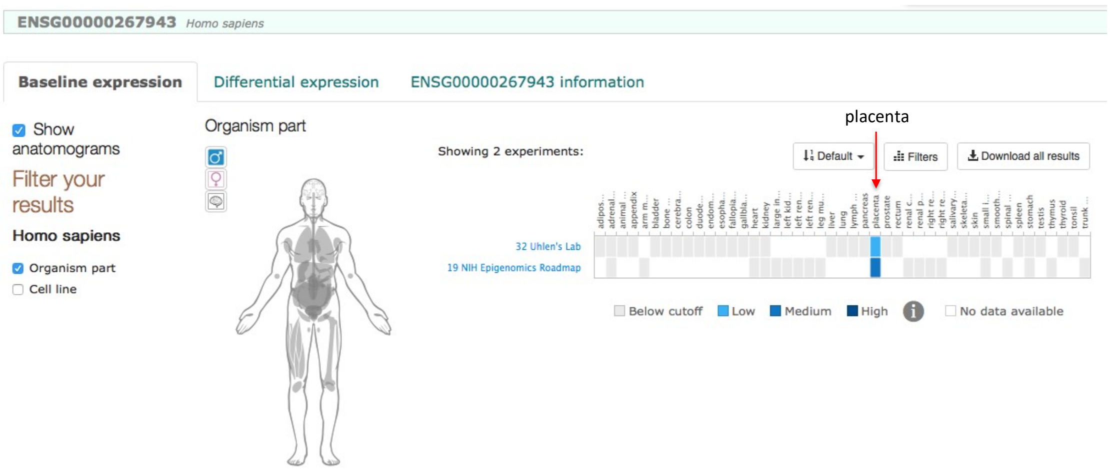

Supplementary Figure 16. LncRNA CTD-2620I22.3 (ENSG00000267943) expression in public transcriptomics data. We assessed orthogonal transcriptomics support for the peptides found for this gene using the EMBL-EBI Expression Atlas (www.ebi.ac.uk/gxa). In two experiments therein, "The Human Protein Atlas" (32 Uhlen's Lab); and "RNA-seq of coding RNA of 19 human tissues from fetuses with congenital defects" (19 NIH epigenomics roadmap), this lncRNA transcript showed specificity to placenta, in agreement with the quantitative preference for placenta shown by the four novel peptides in our normal tissues dataset.

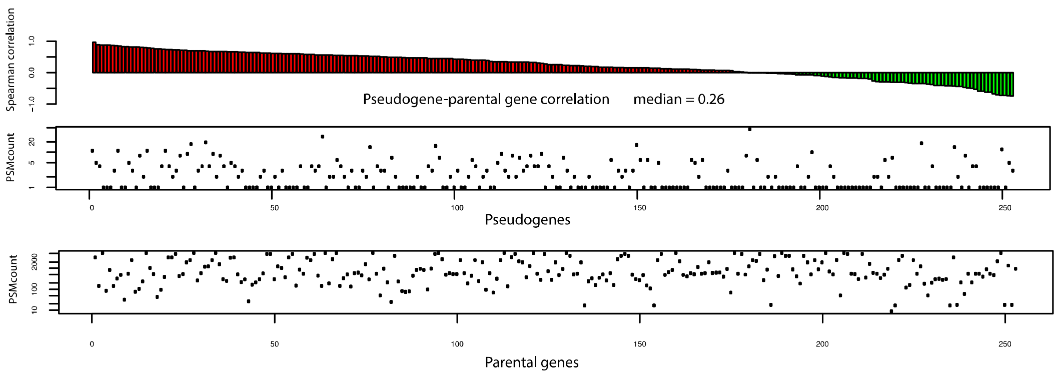

Supplementary Figure 17. Pseudogene and parental gene correlation. Pseudogenes and respective parental genes identified in the A431 cells treated with EGFR inhibitor (gefitinib) are plotted here. Pseudogene and parental gene protein ratios (from 10 samples – TMT reporter ion channels – at four time points, with the control channels used as denominators) were calculated using the median ratio of their unique peptides. On the top, with spearman correlation shown in y-axis, pseudogene-parental gene pairs were ranked from high to low correlation. PSM count of corresponding pseudogene and parental genes are shown below in the same order. Based on the PSM count plots, it can be observed that high or low correlation was not biased to genes identified with high or low number of PSMs.

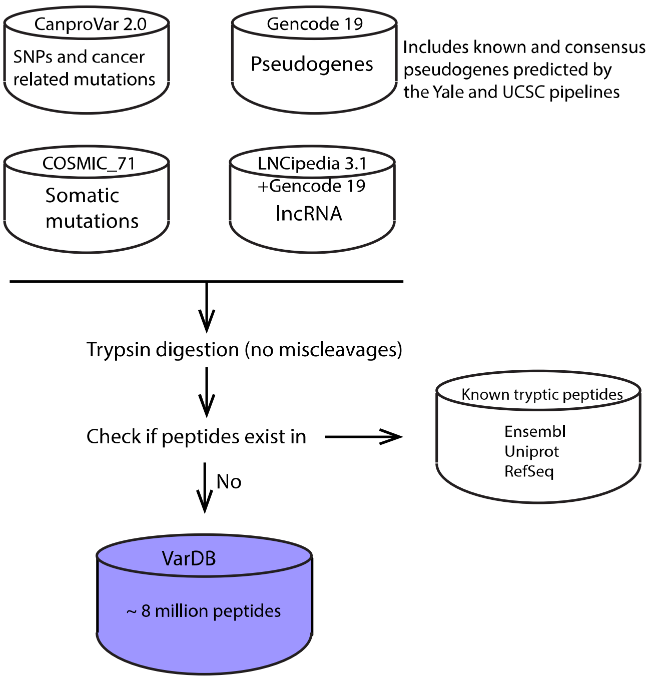

Supplementary Figure 18. VarDB database composition. It combines hypothetical peptide sequences from four different sources: CanproVar 2.0[7], COSMIC 71[8], Gencode 19[9,10] and LNCipedia 3.1[11]. The sequences of pseudogenes and lncRNAs were translated in three reading frames to generate hypothetical peptide sequences and then *in silico* digested by trypsin. Redundant tryptic peptides found in known protein databases were discarded before concatenating to VarDB.

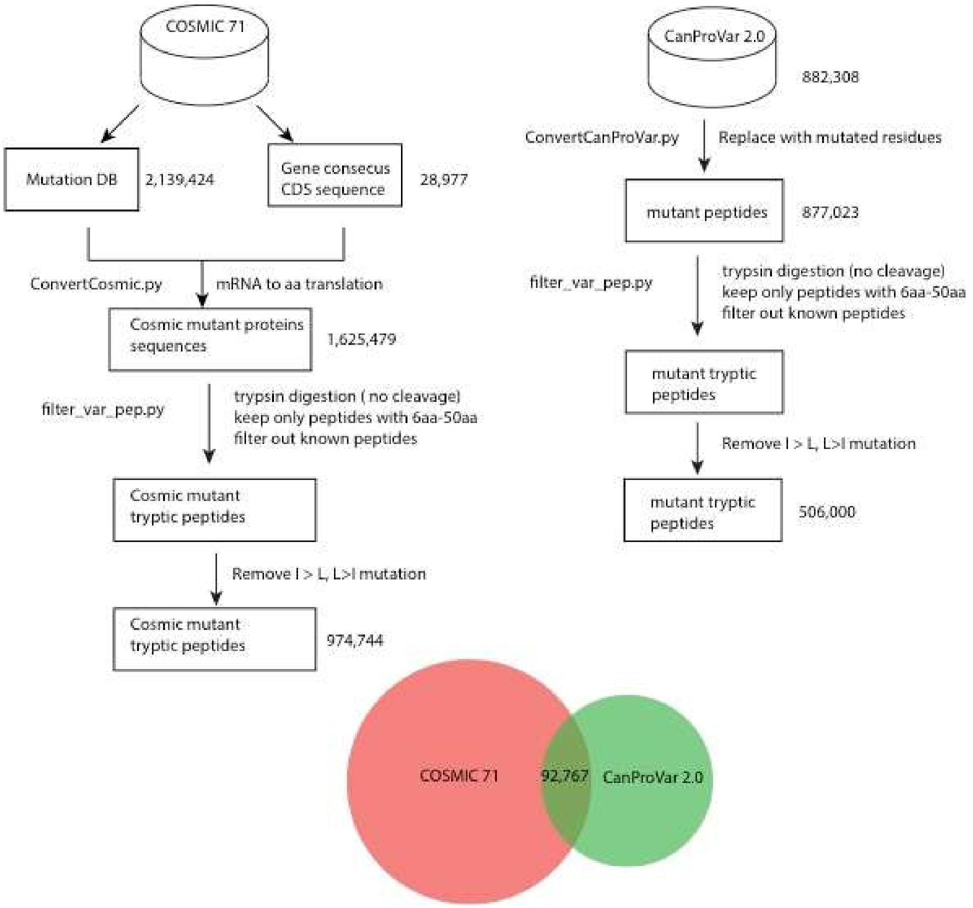

Supplementary Figure 19. Curation of mutant peptides from COSMIC and CanProVar database. Entries from COSMIC[8] were downloaded from http://cancer.sanger.ac.uk/cosmic (version 71) and were converted to mutant protein sequences by customized python script ConvertCosmic.py, then in silico digested into tryptic peptides with the script filter_var_pep.py, which also filtered away redundant known peptide sequences.

CanProVar 2.0 database[7] MSCanProVar_ensemblV79.fasta was downloaded from http://canprovar2.zhang-lab.org. Corresponding variant peptide sequences were generated by python script ConvertCanProVar.py and redundant known sequences were removed. Substitutions of isoleucine to leucine and vice versa were removed in both databases.

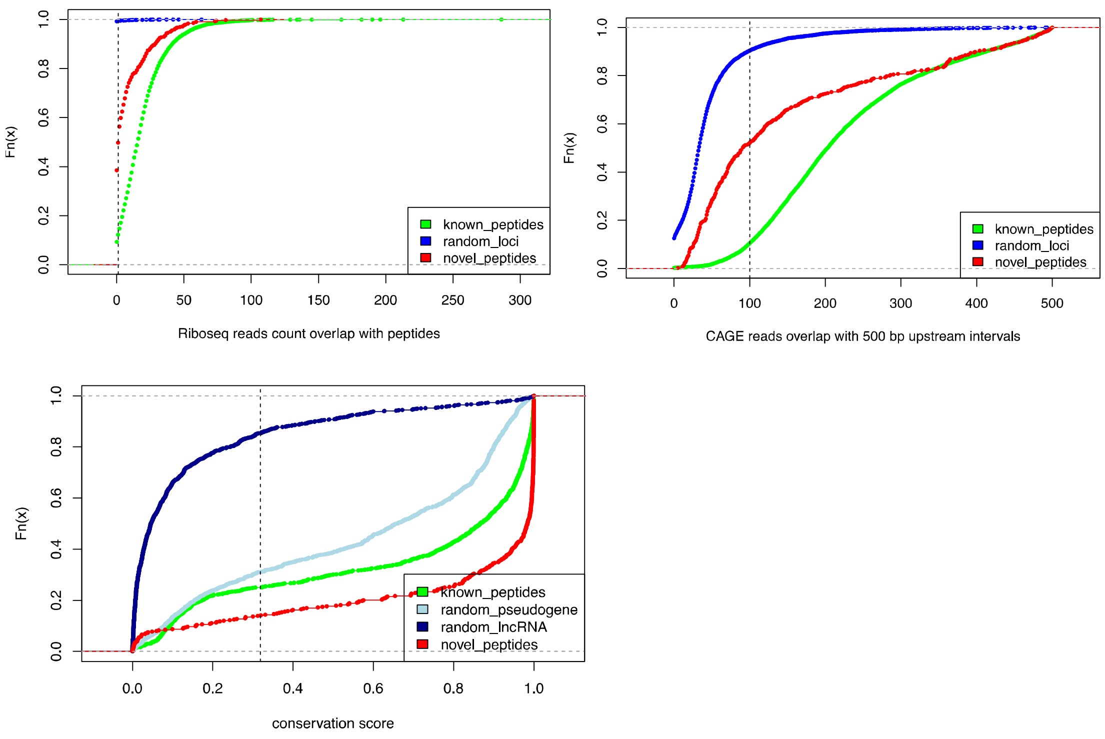

Supplementary Figure 20. The distribution of Ribo-seq reads, CAGE reads and conservation score for known peptides (green), random loci (blue) and novel peptides (red). To choose a threshold to convert Ribo-seq and CAGE evidence[12] to binary values, 10000 random genomic loci were generated to compare their Ribo-seq and CAGE reads counts with those of known and novel peptides. For Ribo-seq and CAGE reads, 1 and 100 were used, respectively, as thresholds to determine if a novel peptide has support or not. For conservation, 0.3184 (first quartile of known peptides) was used as the cutoff.

Supplementary References:

1. Savitski MM, Wilhelm M, Hahne H, Kuster B, Bantscheff M. A Scalable Approach for Protein False Discovery Rate Estimation in Large Proteomic Data Sets. *Molecular & cellular proteomics : MCP* **14**, 2394-2404 (2015).

2. Wilhelm M*, et al.* Mass-spectrometry-based draft of the human proteome. *Nature* **509**, 582-587 (2014).

3. Muthusamy S*, et al.* Comparative proteomics reveals signature metabolisms of exponentially growing and stationary phase marine bacteria. *Environmental Microbiology* **19**, 2301-2319 (2017).

4. Zhu Y*, et al.* Proteogenomics produces comprehensive and highly accurate protein-coding gene annotation in a complete genome assembly of Malassezia sympodialis. *Nucleic Acids Research* **45**, 2629-2643 (2017).

5. Nesvizhskii AI. Proteogenomics: concepts, applications and computational strategies. *Nature methods* **11**, 1114-1125 (2014).

6. Kim S, Pevzner PA. MS-GF+ makes progress towards a universal database search tool for proteomics. *Nature Communications* **5**, (2014).

7. Li J, Duncan DT, Zhang B. CanProVar: a human cancer proteome variation database. Human mutation 31, 219-228 (2010).

8. Forbes SA, et al. COSMIC: somatic cancer genetics at high-resolution. Nucleic acids research 45, D777-D783 (2017).

9. Pei B*, et al.* The GENCODE pseudogene resource. *Genome Biology* **13**, R51 (2012).

10. Harrow J*, et al.* GENCODE: the reference human genome annotation for The ENCODE Project. *Genome research* **22**, 1760-1774 (2012).

11. Volders PJ*, et al.* LNCipedia: a database for annotated human lncRNA transcript sequences and structures. *Nucleic acids research* **41**, D246-251 (2013).

12. Forrest AR*, et al.* A promoter-level mammalian expression atlas. *Nature* **507**, 462-470 (2014).
