# Supplementary material for: Discovery of coding regions in the human genome by integrated proteogenomics analysis workflow
Source: Nat Commun. 2018 Mar 2;9:903. doi: 10.1038/s41467-018-03311-y (PMC5834625; doi:10.1038/s41467-018-03311-y)
Supplement: Supplementary file 3 — Description of Additional Supplementary Files [file 41467_2018_3311_MOESM3_ESM.pdf]

Description of Additional Supplementary Files

File Name: Supplementary Data 1
Description: Excel file containing three sheets with updated pK constants to be used with the PredpI algorithm which is available with the Branca *et al.* 2014 Nat Methods publication (doi: 10.1038/nmeth.2732). The three sheets are for (i) plain (label free), (ii) TMT6plex-labeled (or TMT10plex-labeled), and (iii) iTRAQ8plex-labeled peptides. In order to be used with the PredpI program, they must first be exported into individual tab-separated text (.txt) files.

File Name: Supplementary Data 2
Description: Excel table with single amino acid variant (SAAV) peptides identified, respective support at DNA and RNA levels, and result of SpectrumAI inspection.

File Name: Supplementary Data 3
Description: PDF file with 128 mirror plots comparing, for each peptide, the "endogenous" (*i.e.* from either the A431 or normal tissues datasets) peptide MS2 spectrum with the corresponding "synthetic" (purchased) peptide MS2 spectrum to visually assess correctness of identification. Endogenous peptides in pages 20-30 and 122-128 are deemed incorrect identifications.

File Name: Supplementary Data 4
Description: Excel table with novel peptides identified from the A431 cells dataset, with details on genomic coordinates, type of event (*e.g.* pseudogene, AltORF, etc), and level of support from various orthogonal data.

File Name: Supplementary Data 5
Description: Excel table with novel peptides identified from the normal tissues dataset, with details on genomic coordinates, type of event (*e.g.* pseudogene, AltORF, etc), and level of support from various orthogonal data.

File Name: Supplementary Data 6
Description: PDF file with TargetP 1.1 analysis (http://www.cbs.dtu.dk/services/TargetP/) of three N-terminally extended proteins found, and their respective canonical proteins.

File Name: Supplementary Data 7
Description: PDF file with the gene models for 40 novel protein coding regions, all with multiple peptides that are linked to in-frame initiation start codons.

File Name: Supplementary Data 8
Description: PDF file with TMT-based quantification of novel protein coding events and their respective canonical counterparts (where applicable) in both the normal tissues dataset (tissue specificity) and the A431 cells dataset (regulation upon gefitinib treatment). Each dot in the TMT ratio plots represents quantification by an individual PSM, the centre bar is the mean and error bars represent the standard deviation.
