# Supplementary figures and images for: Discovery of coding regions in the human genome by integrated proteogenomics analysis workflow
Source: Nat Commun. 2018 Mar 2;9:903. doi: 10.1038/s41467-018-03311-y (PMC5834625; doi:10.1038/s41467-018-03311-y)

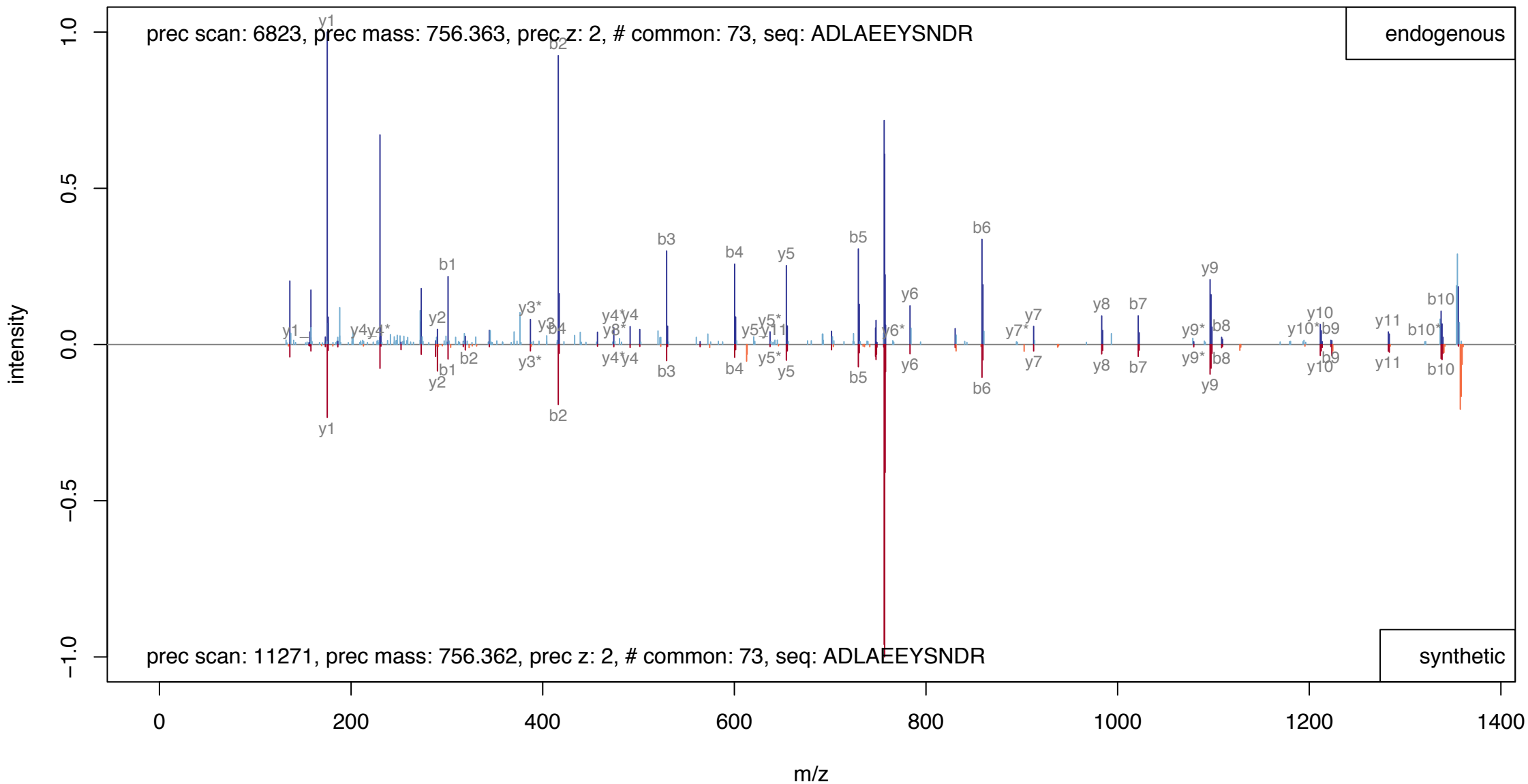

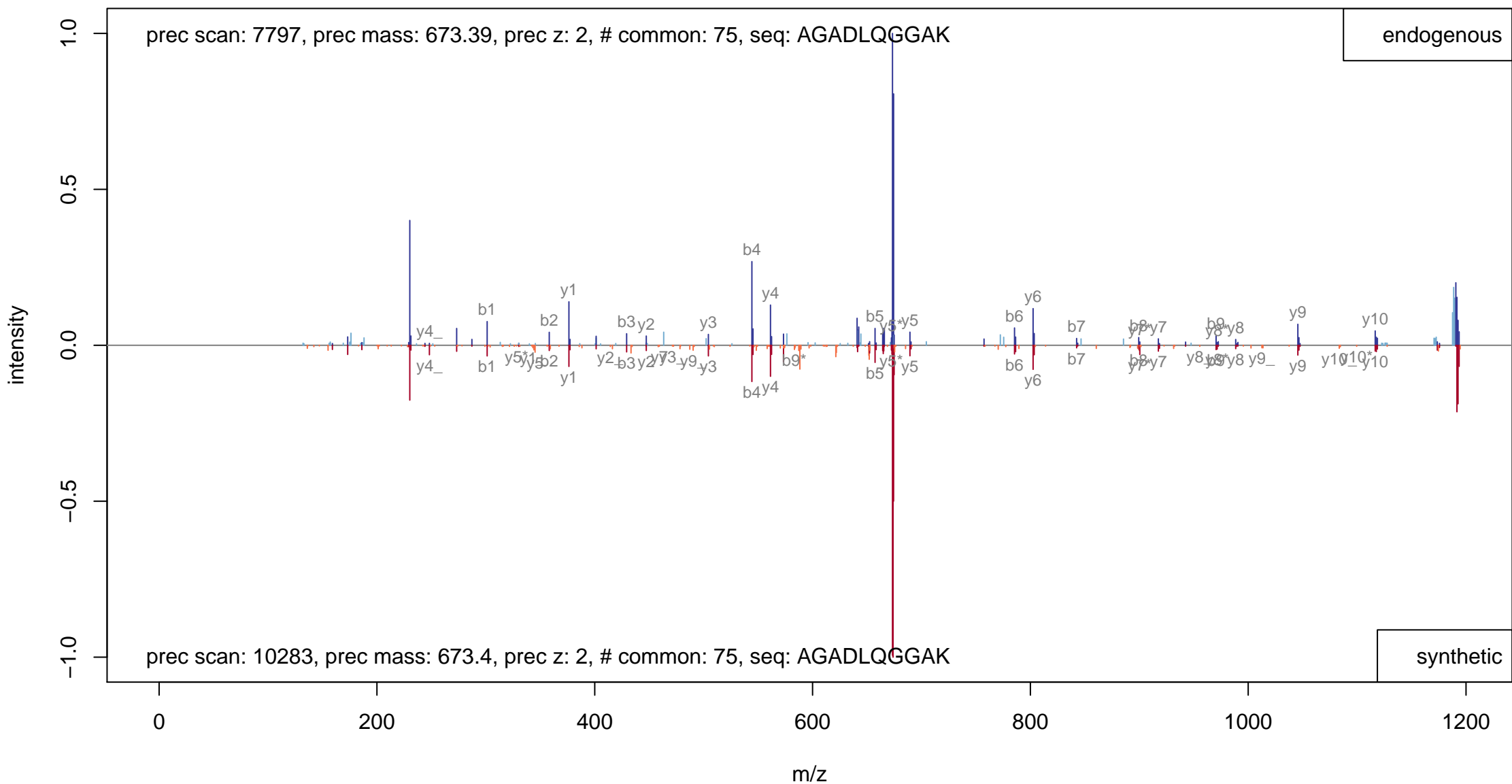

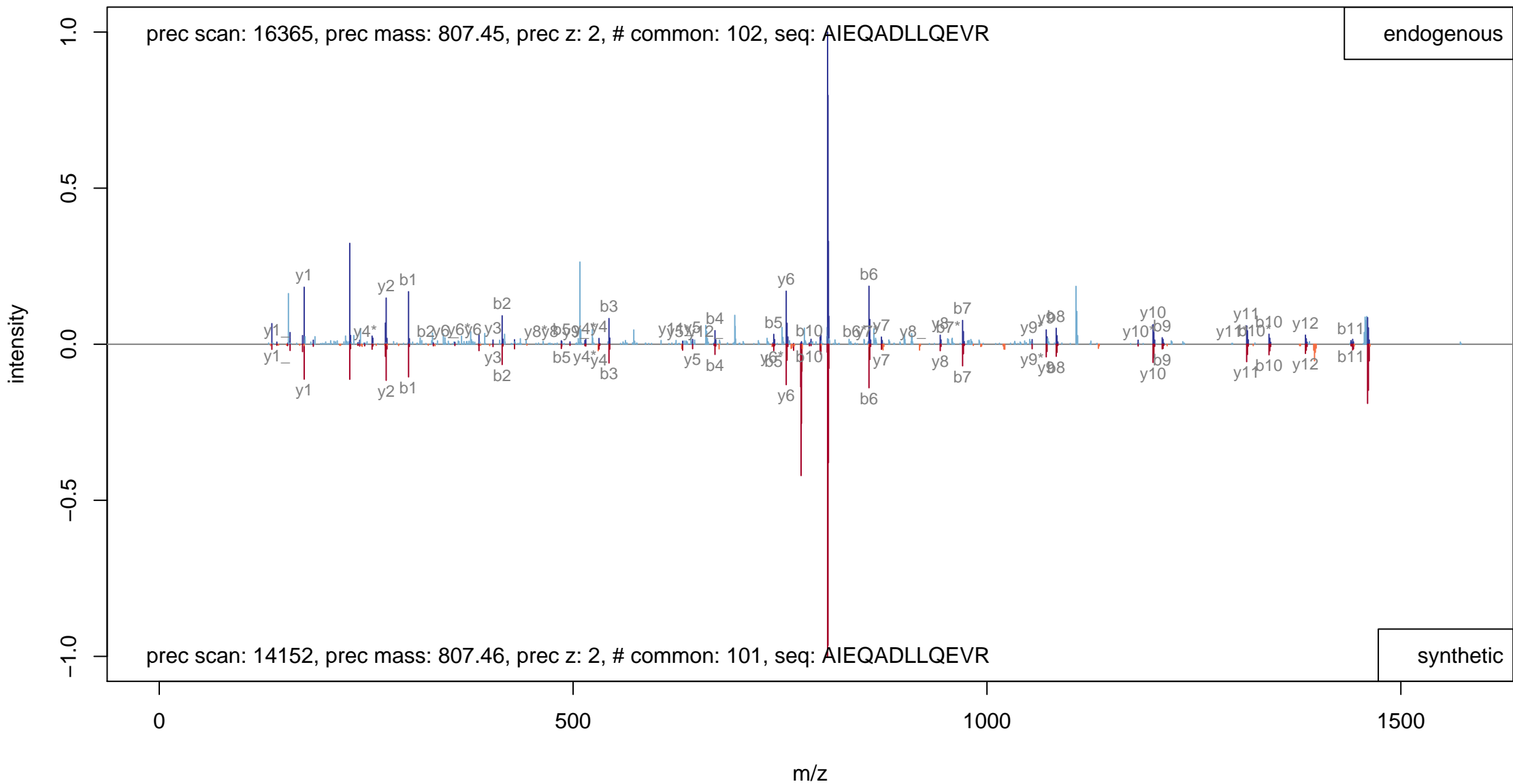

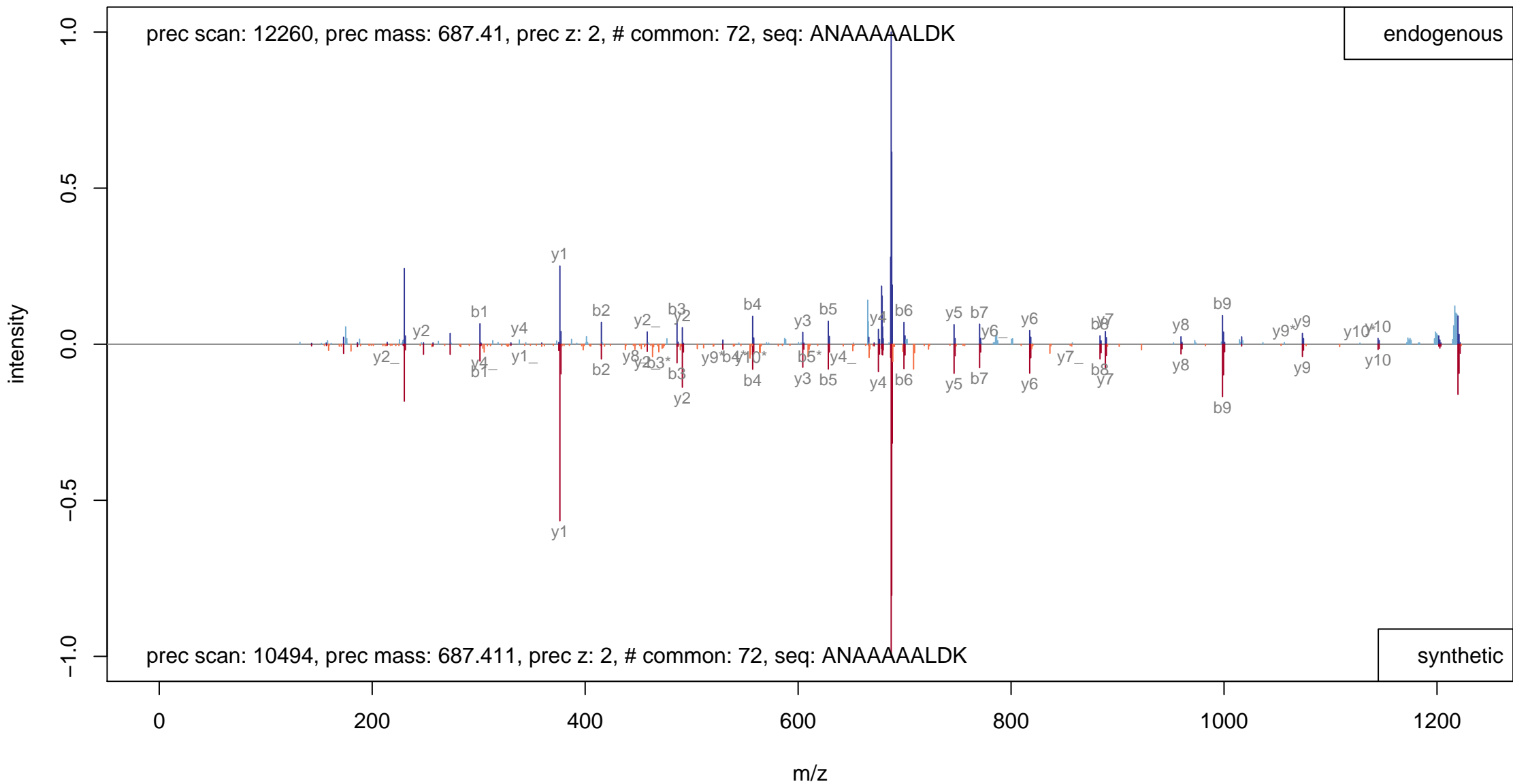

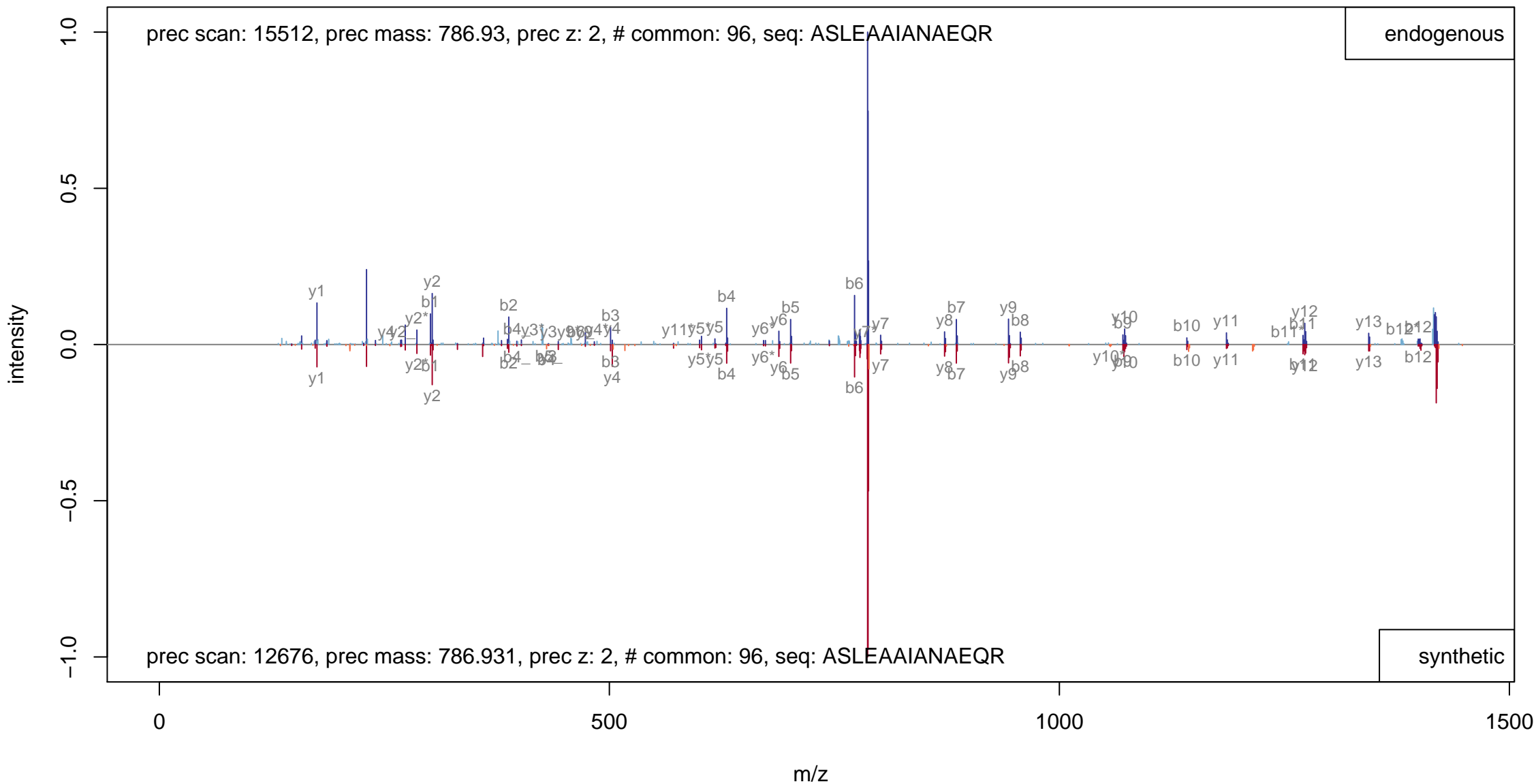

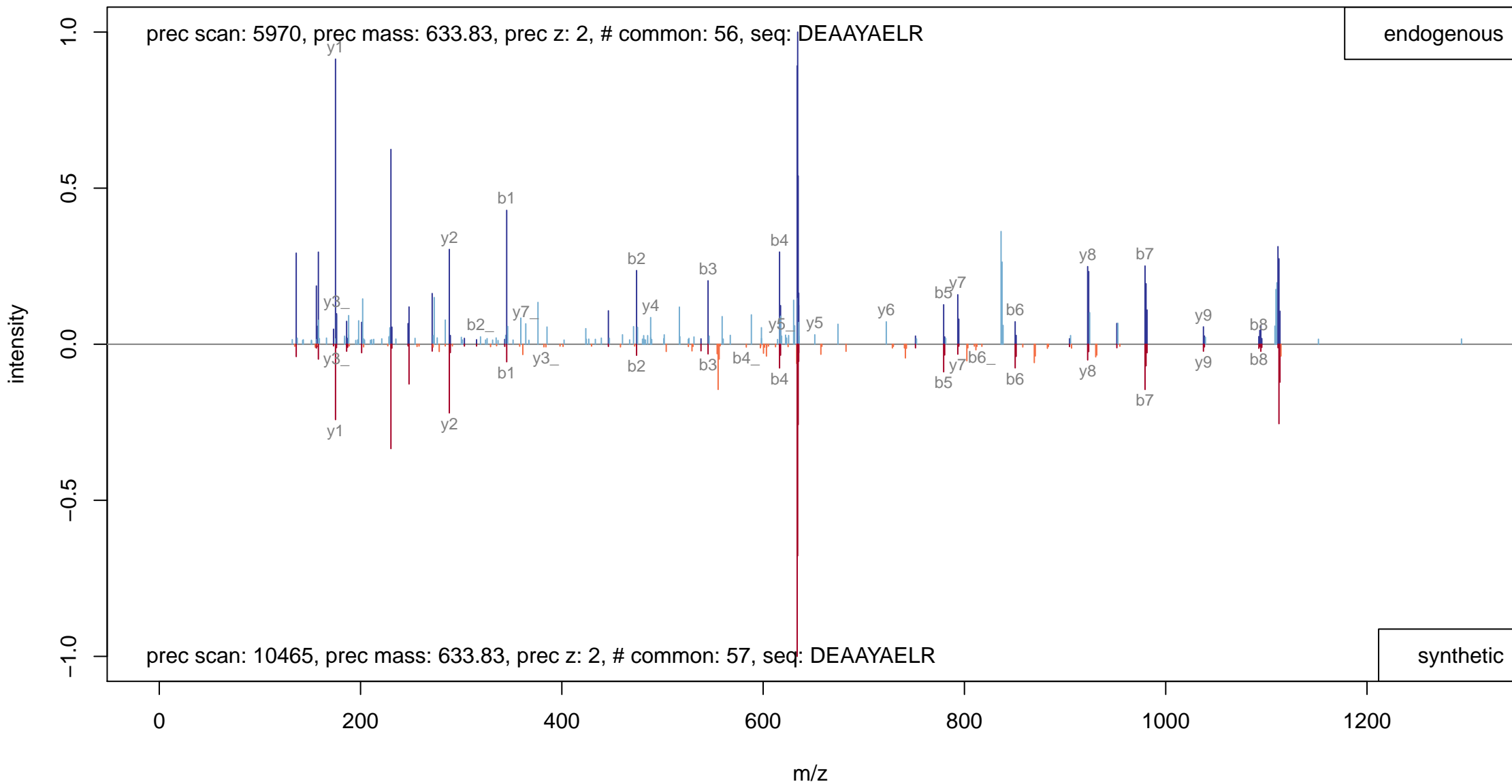

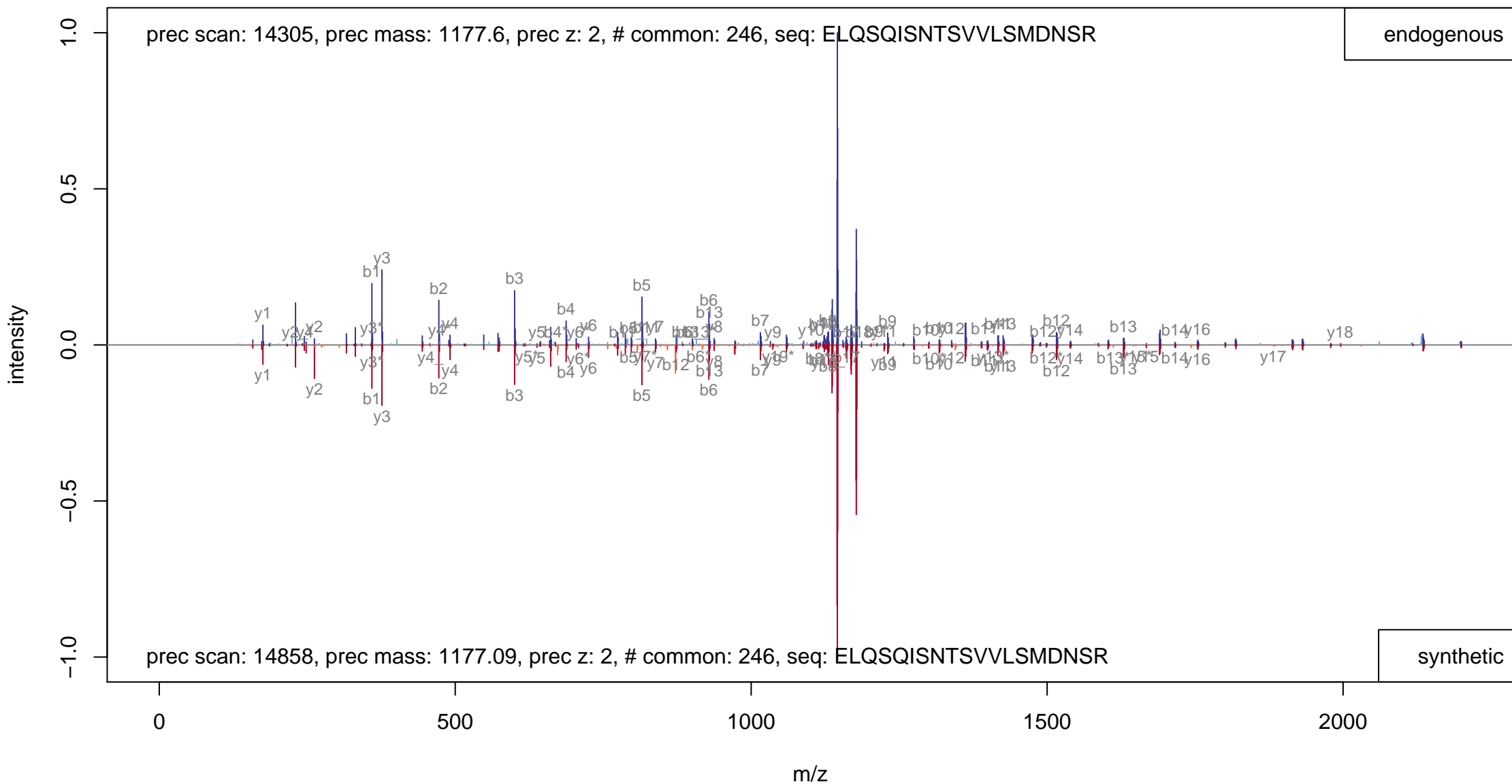

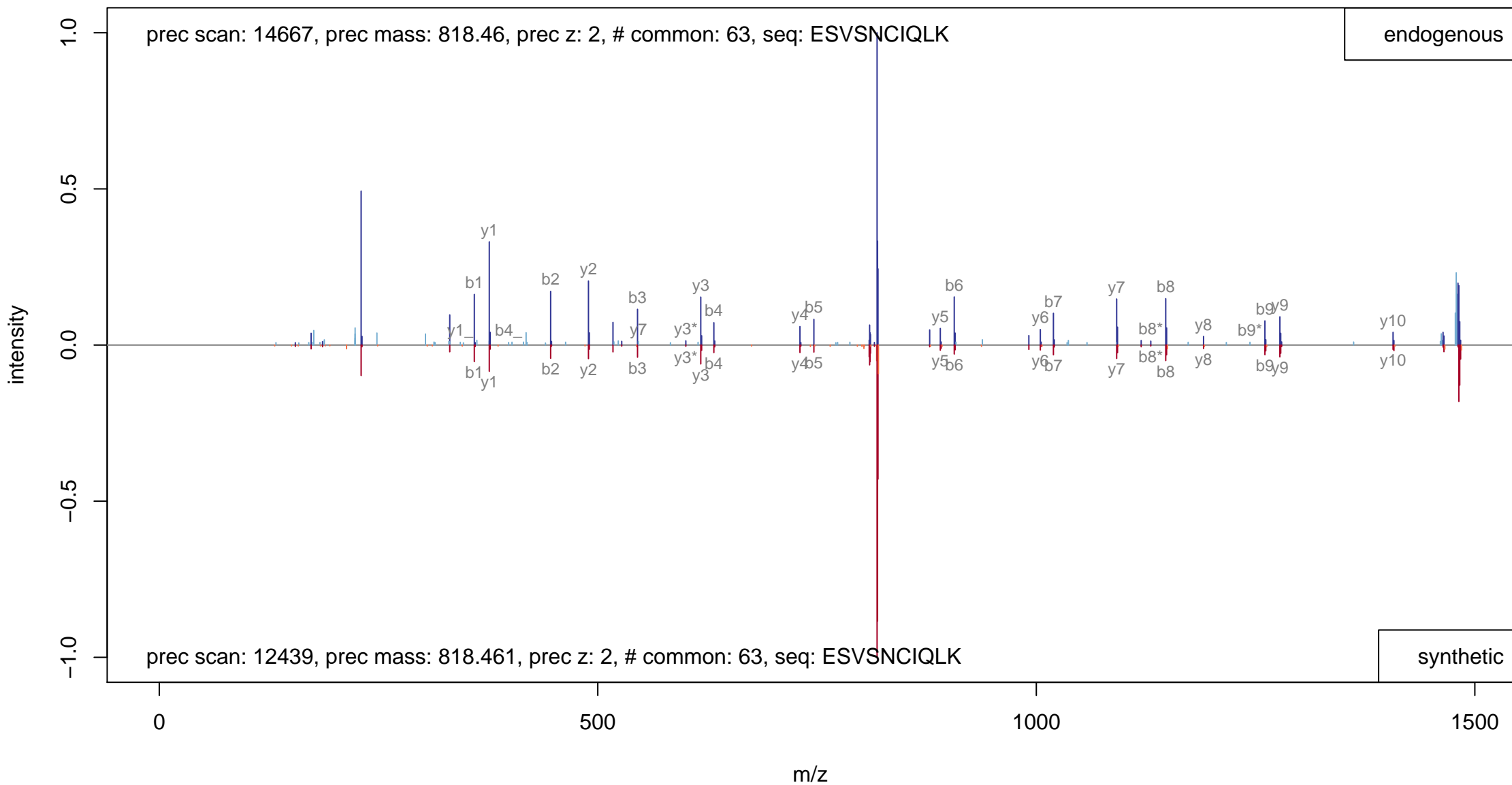

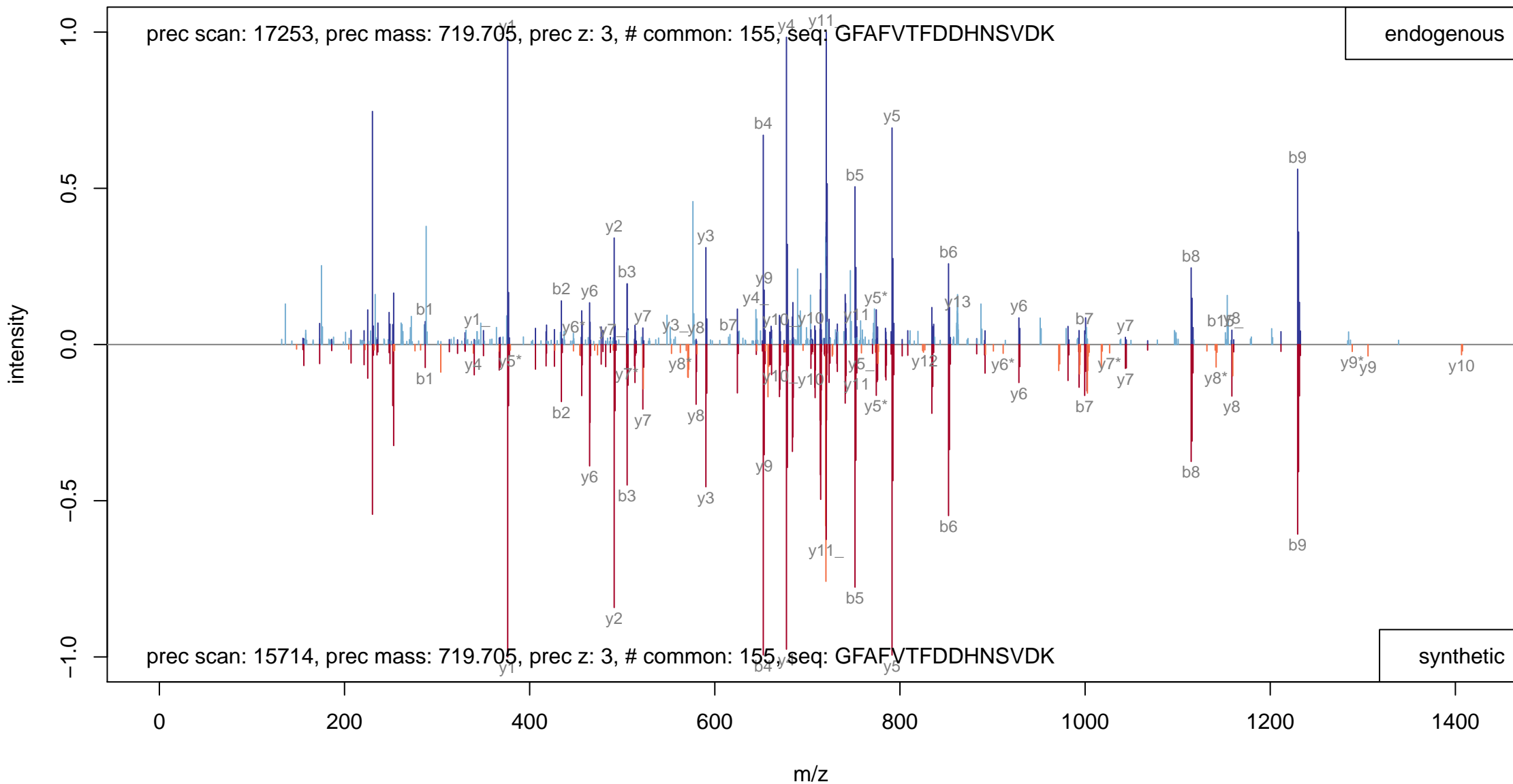

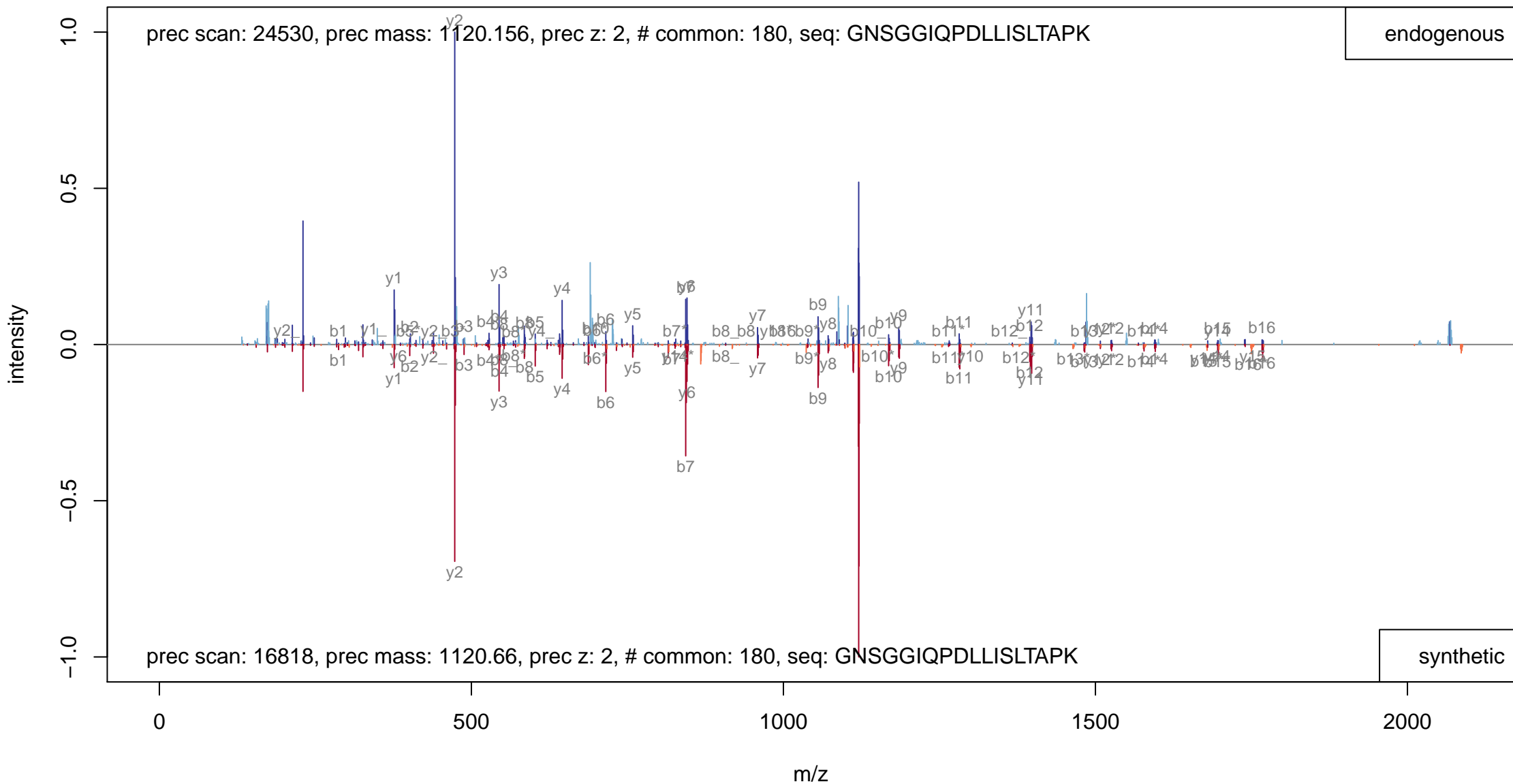

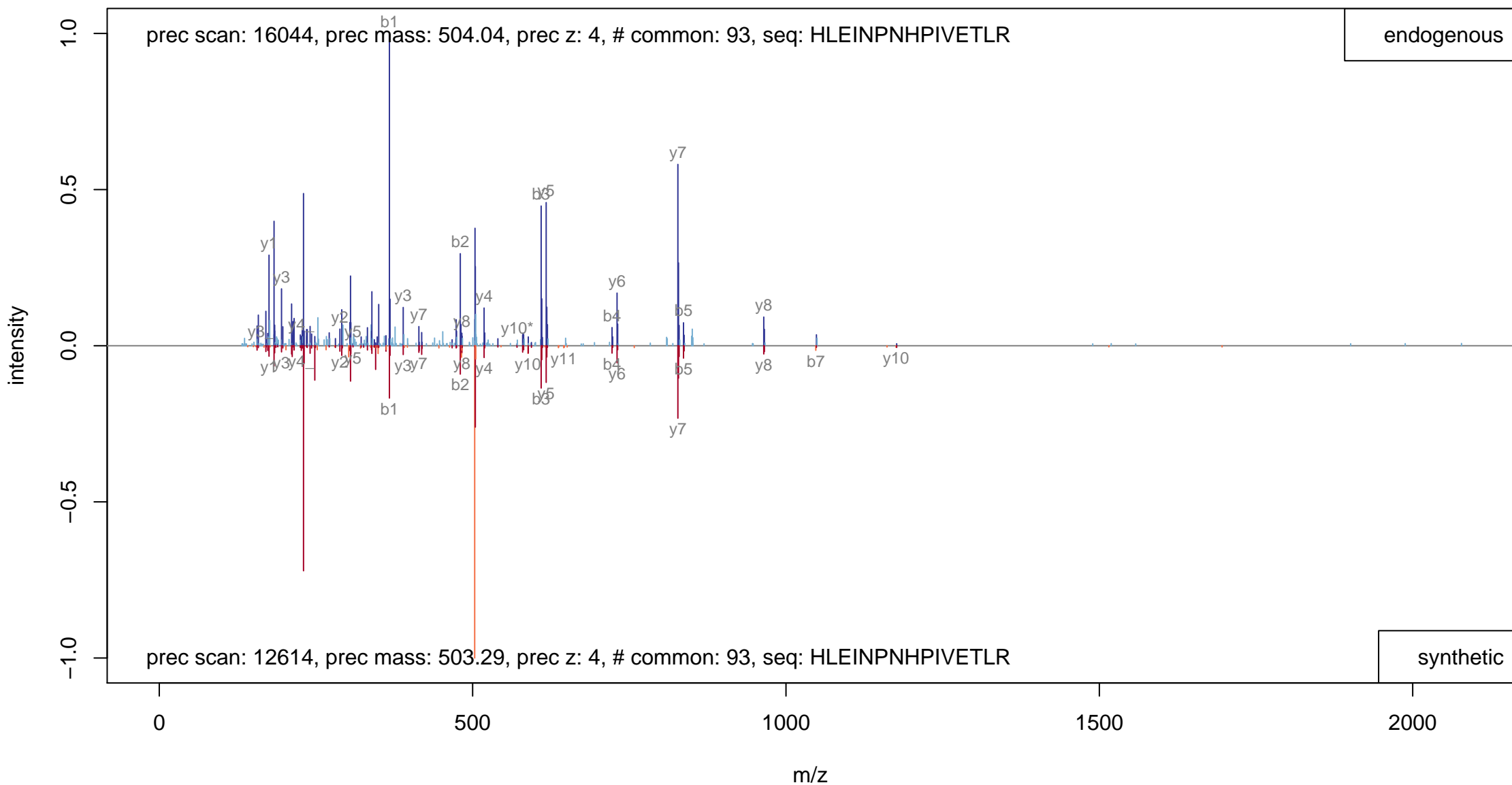

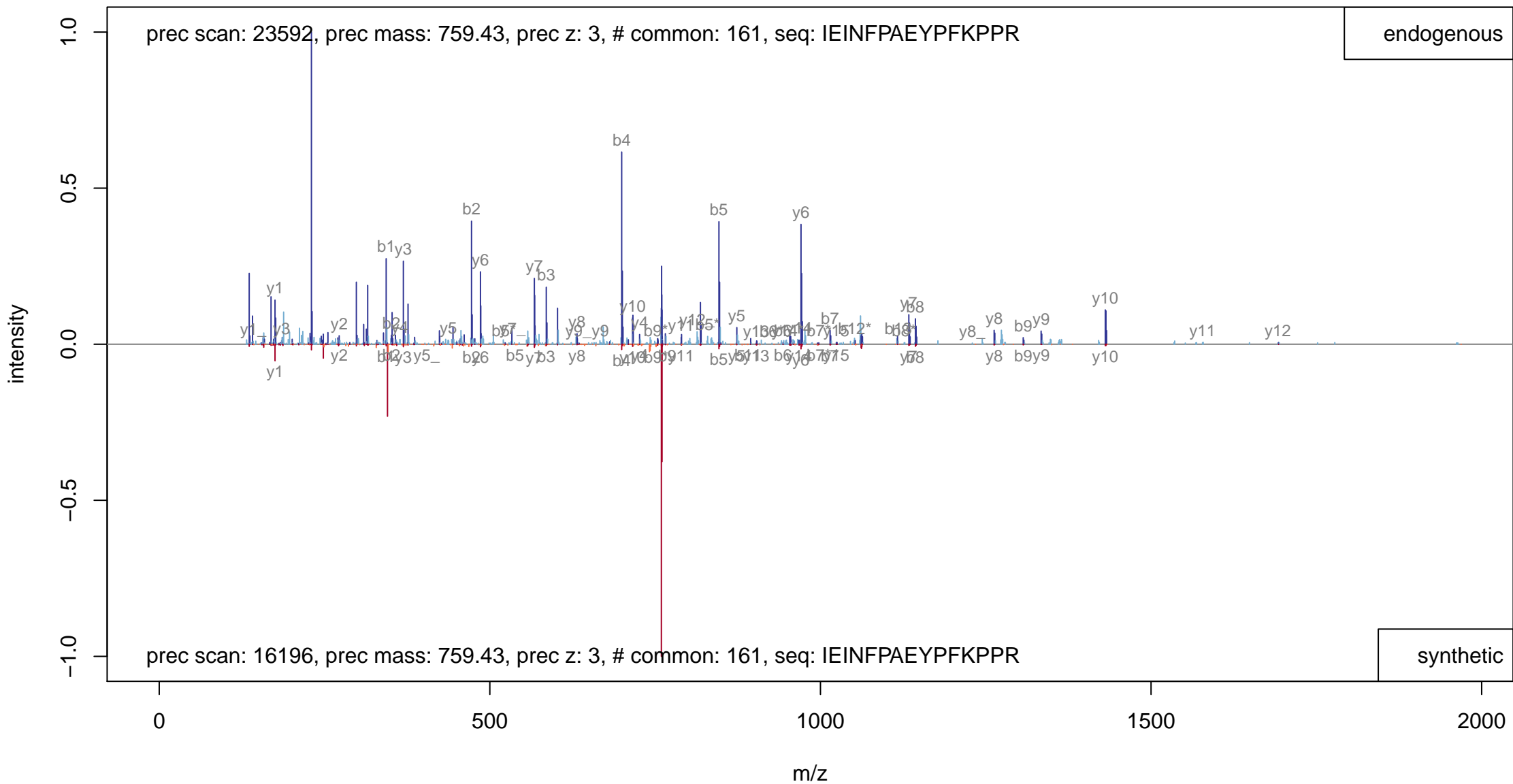

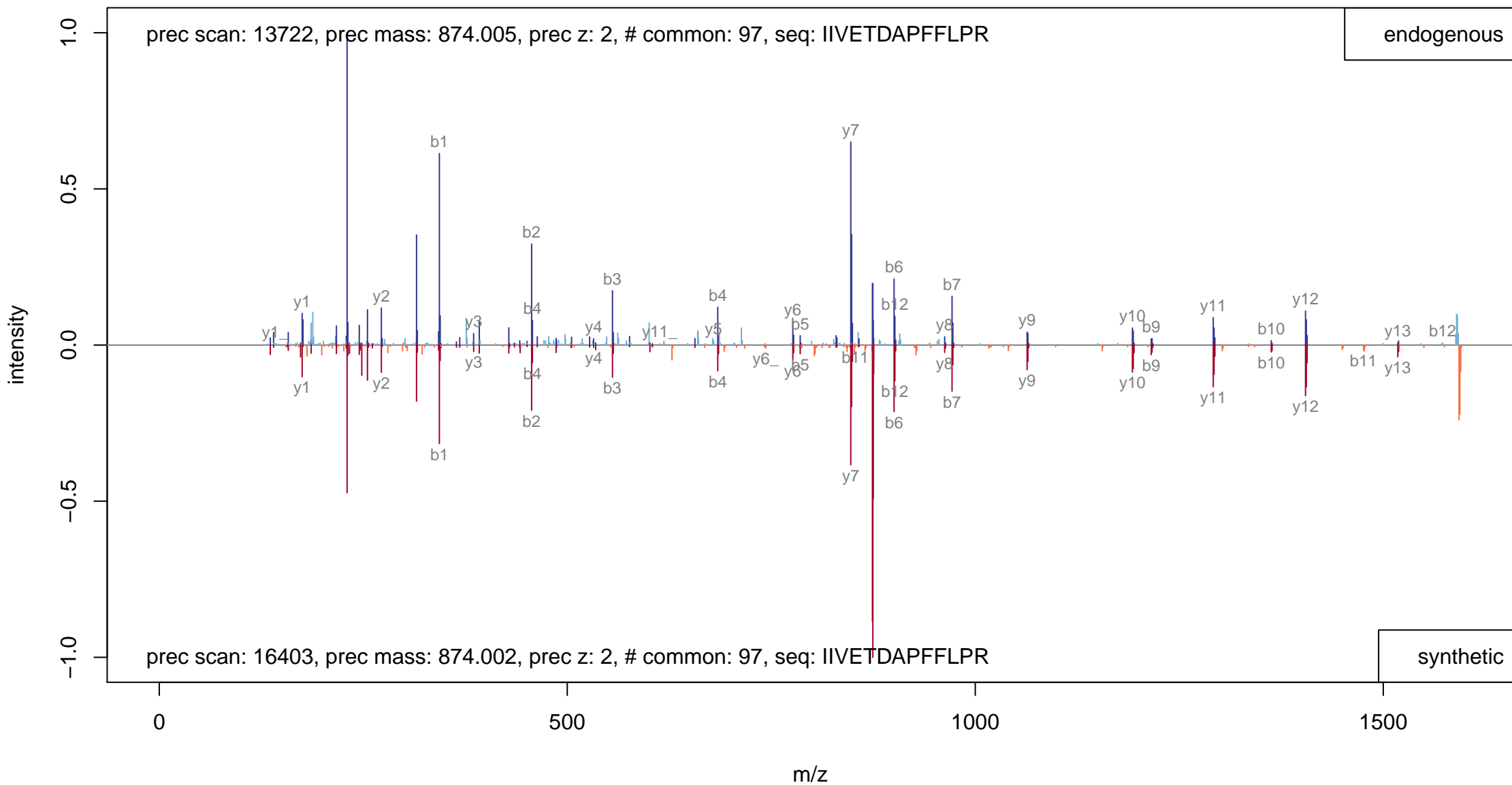

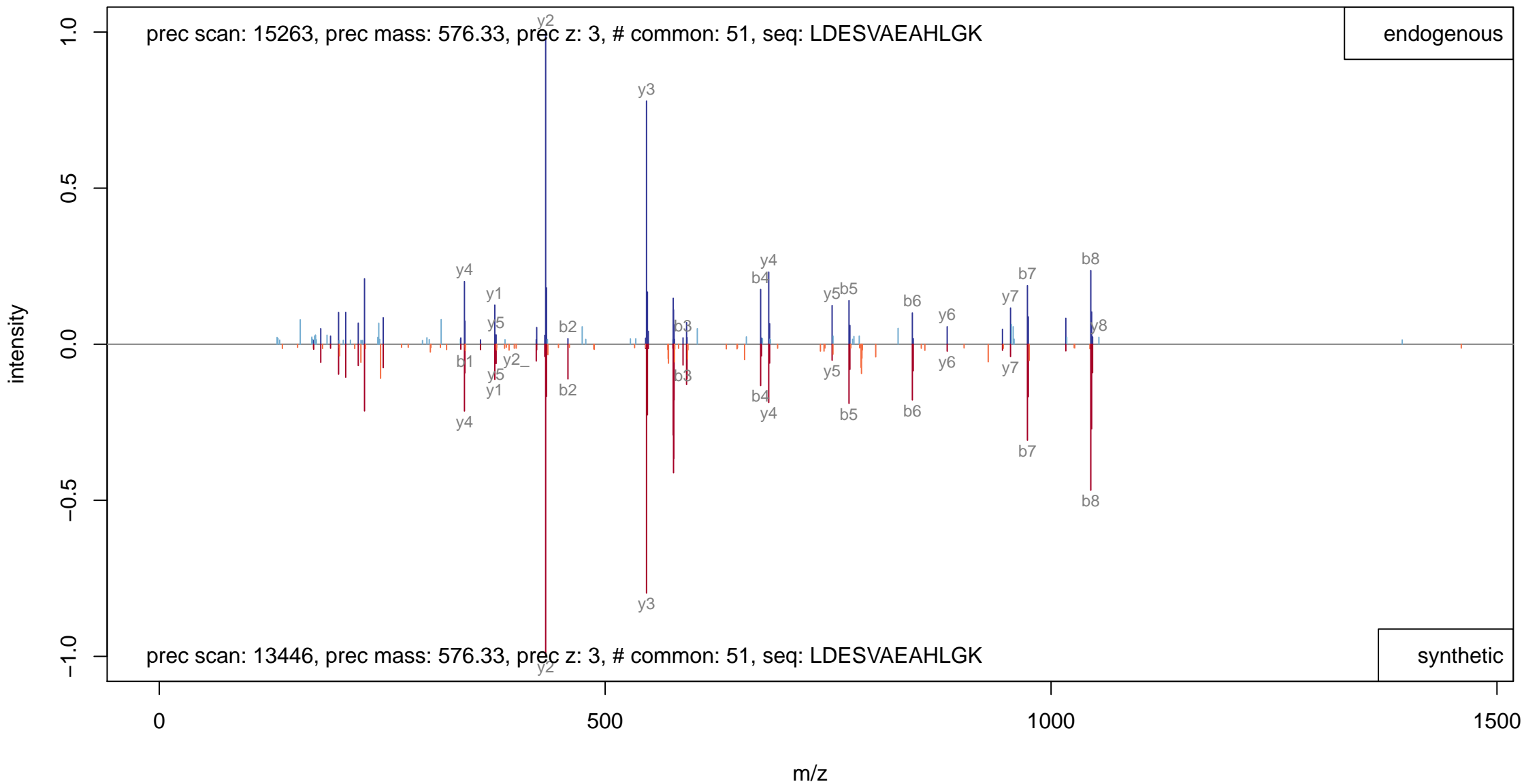

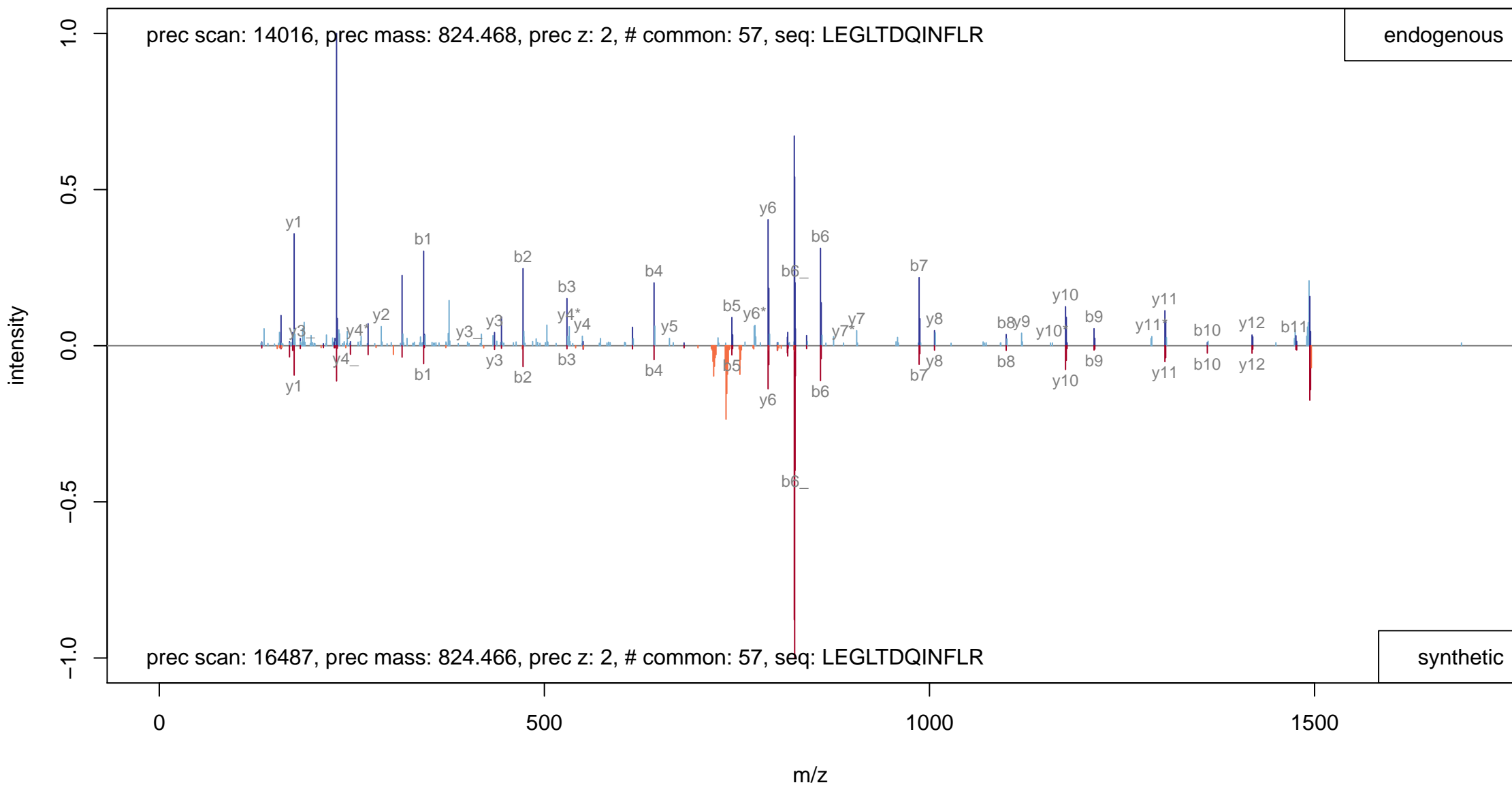

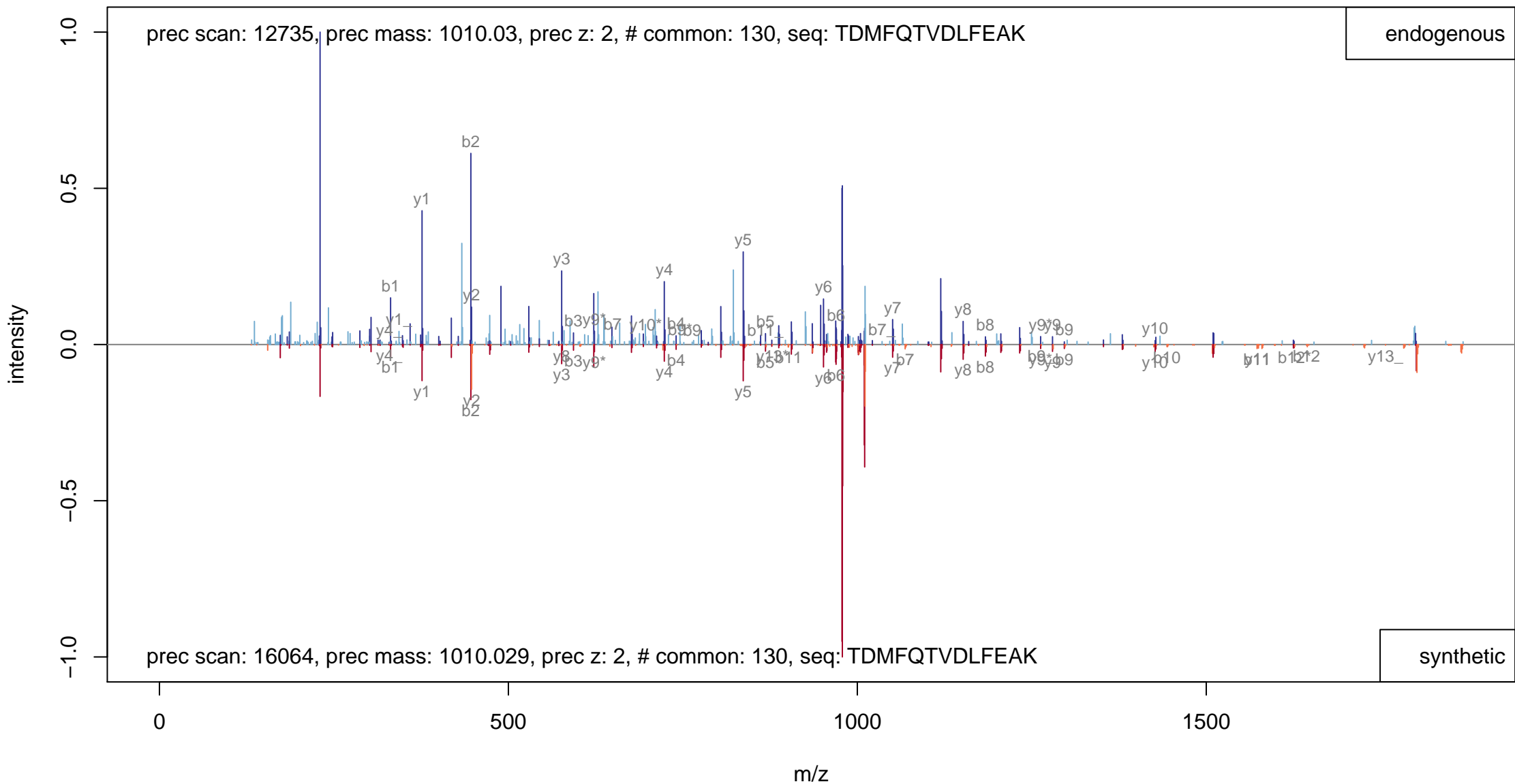

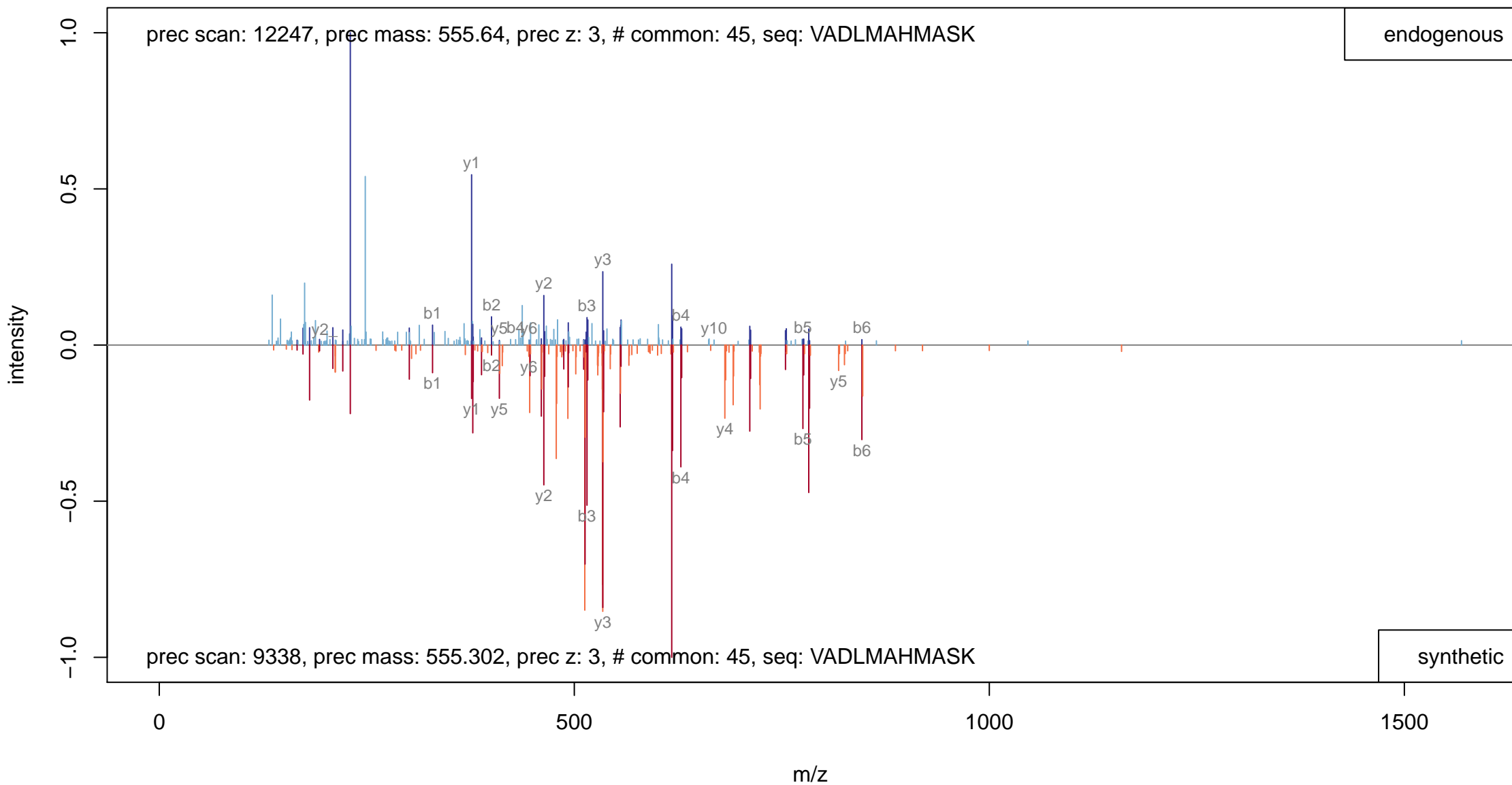

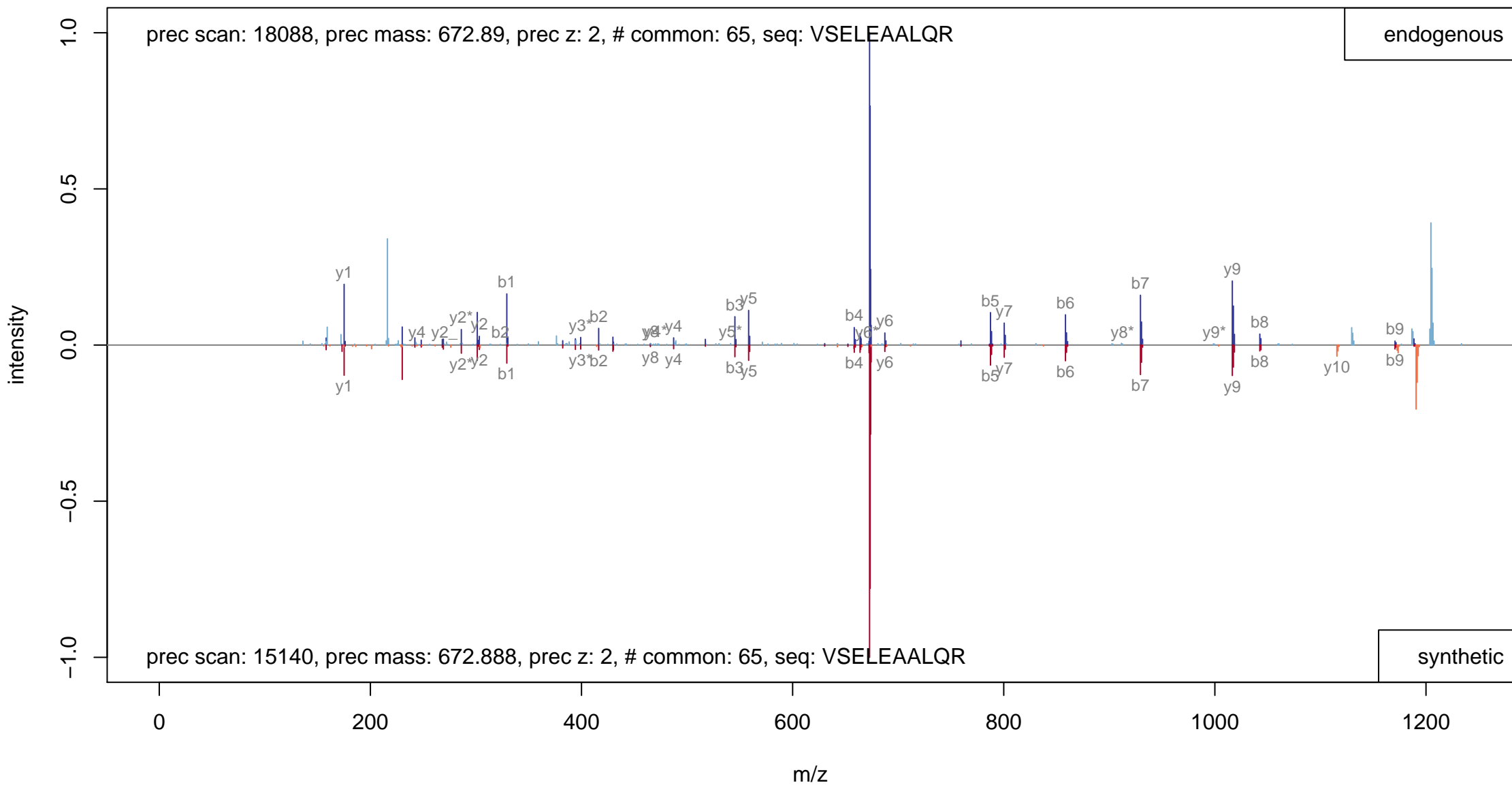

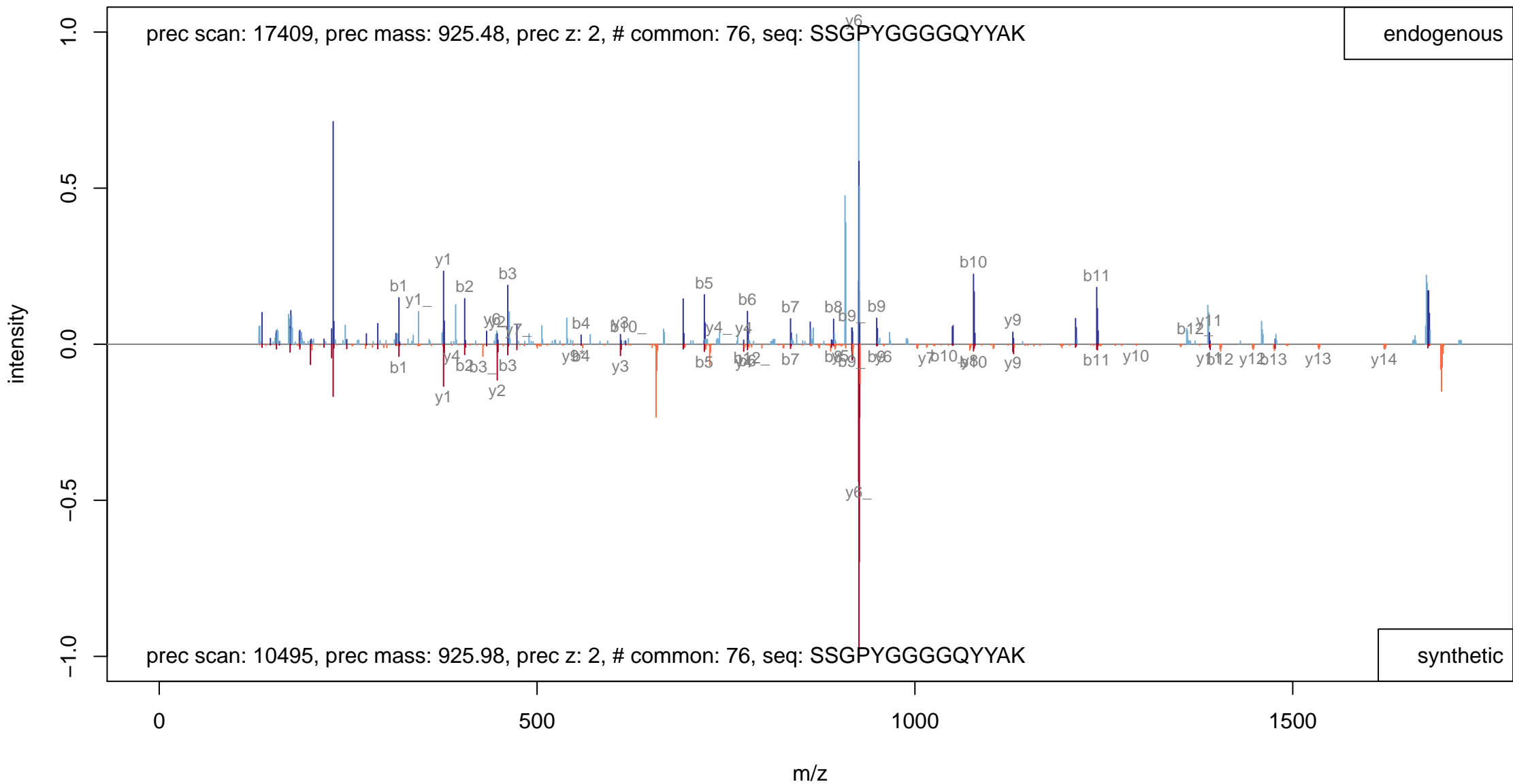

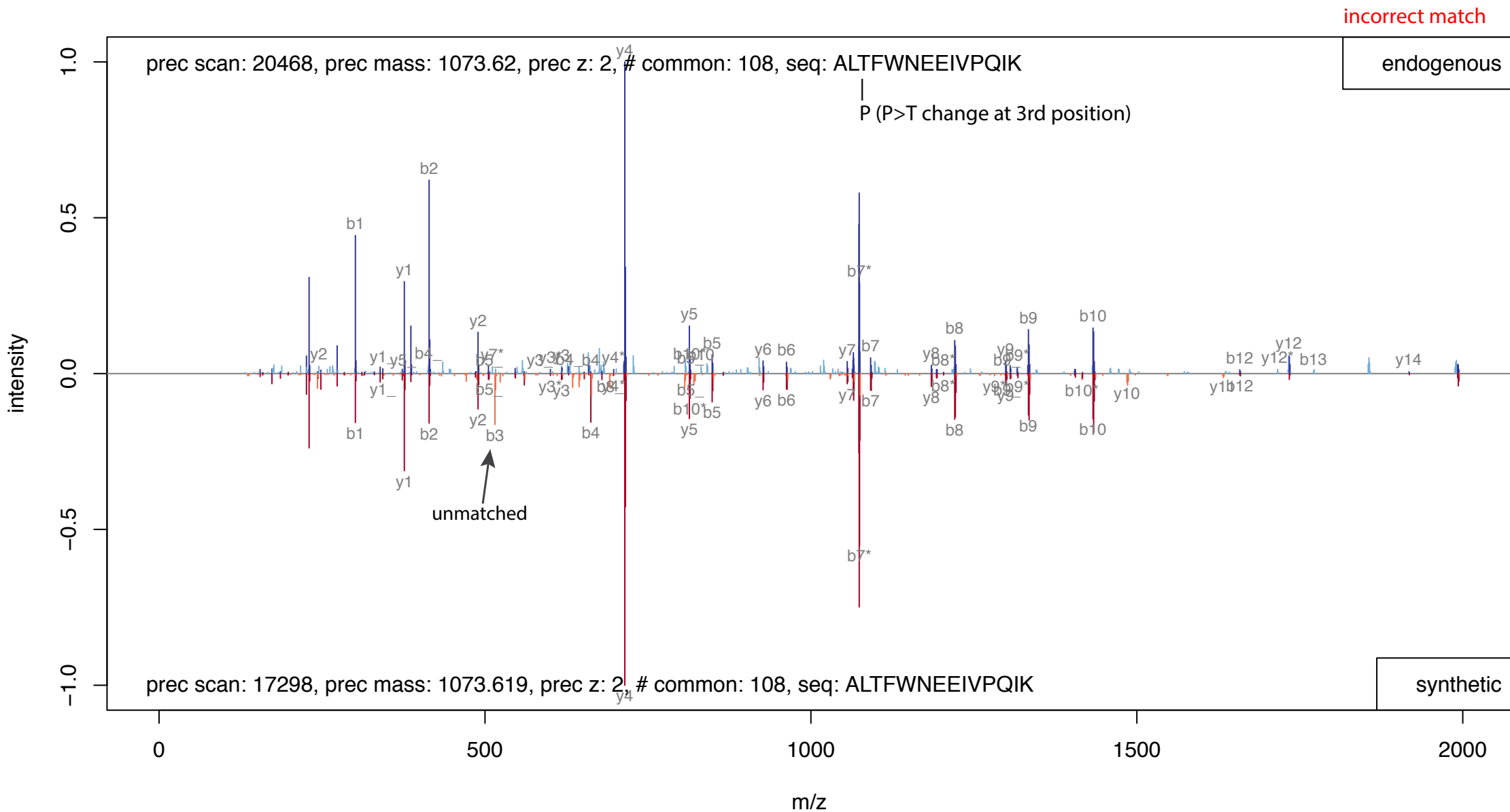

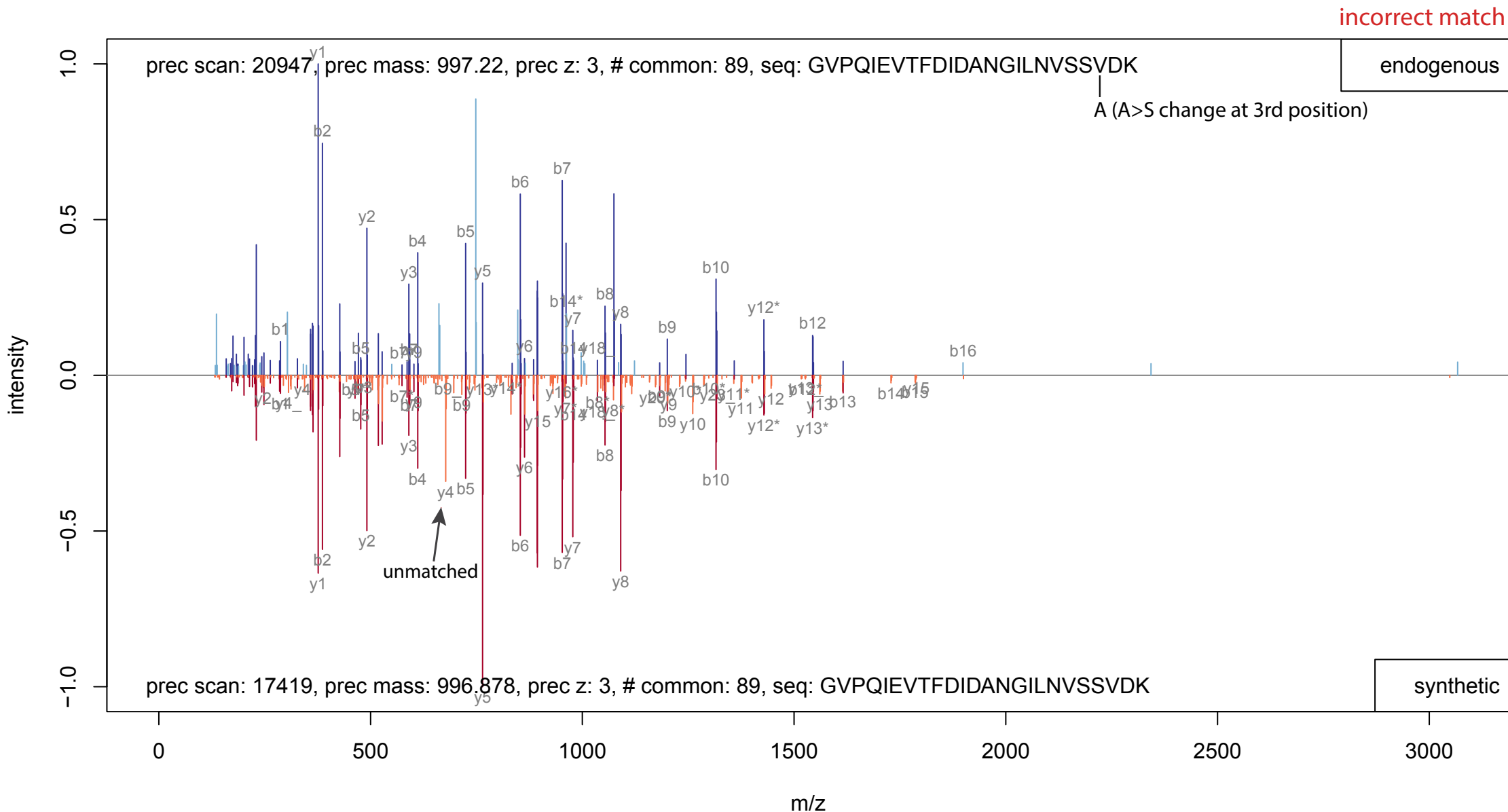

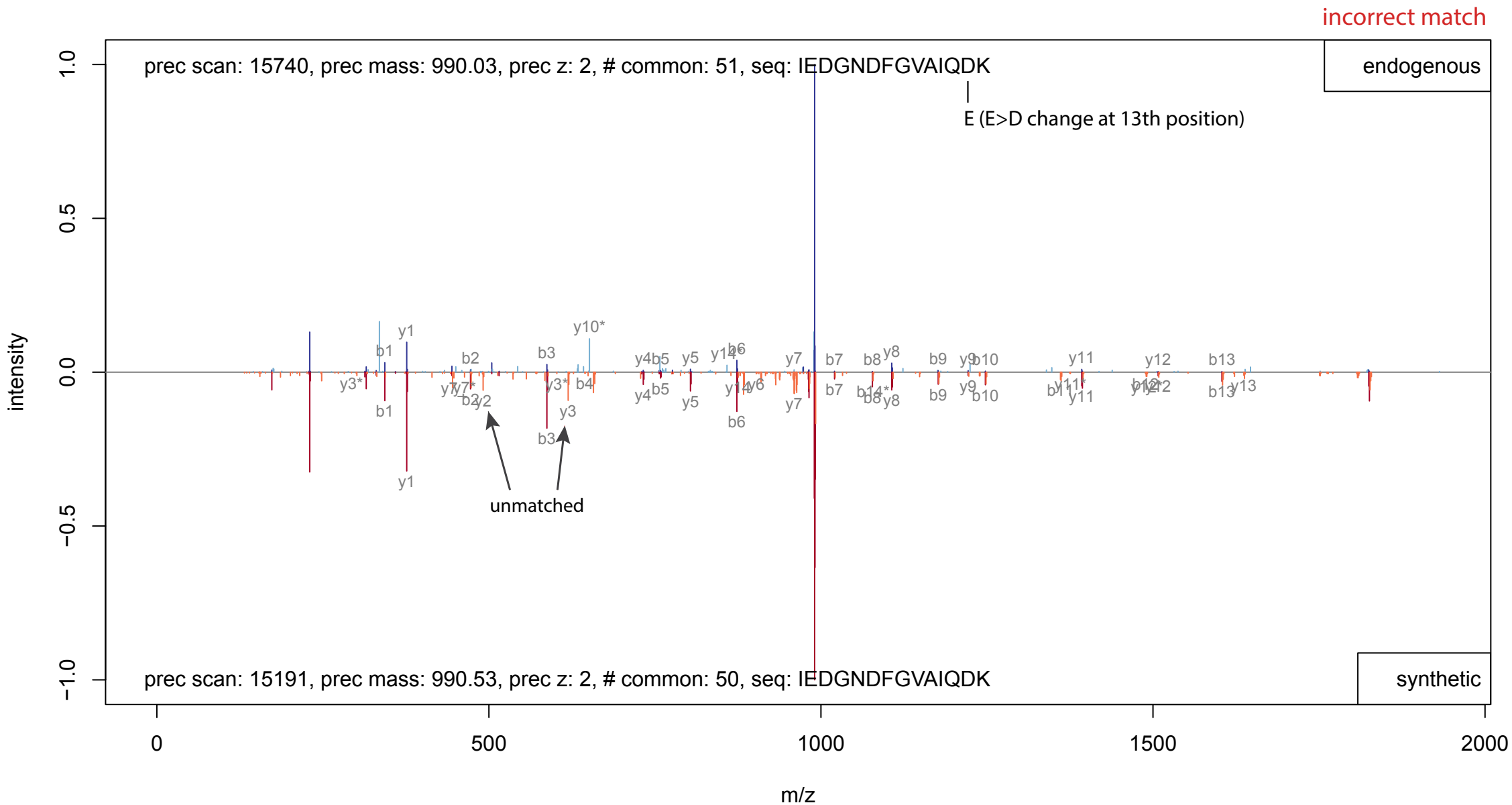

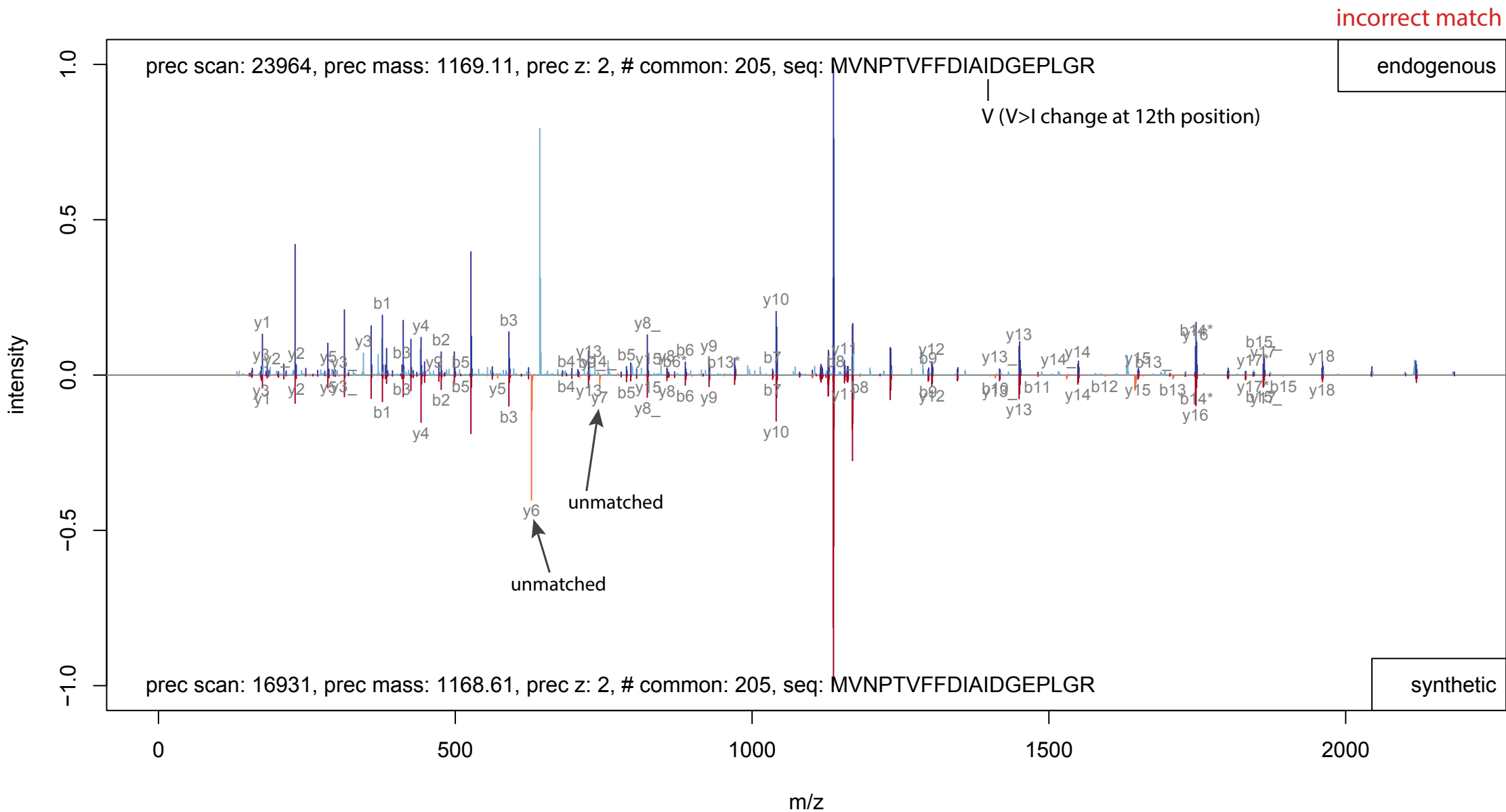

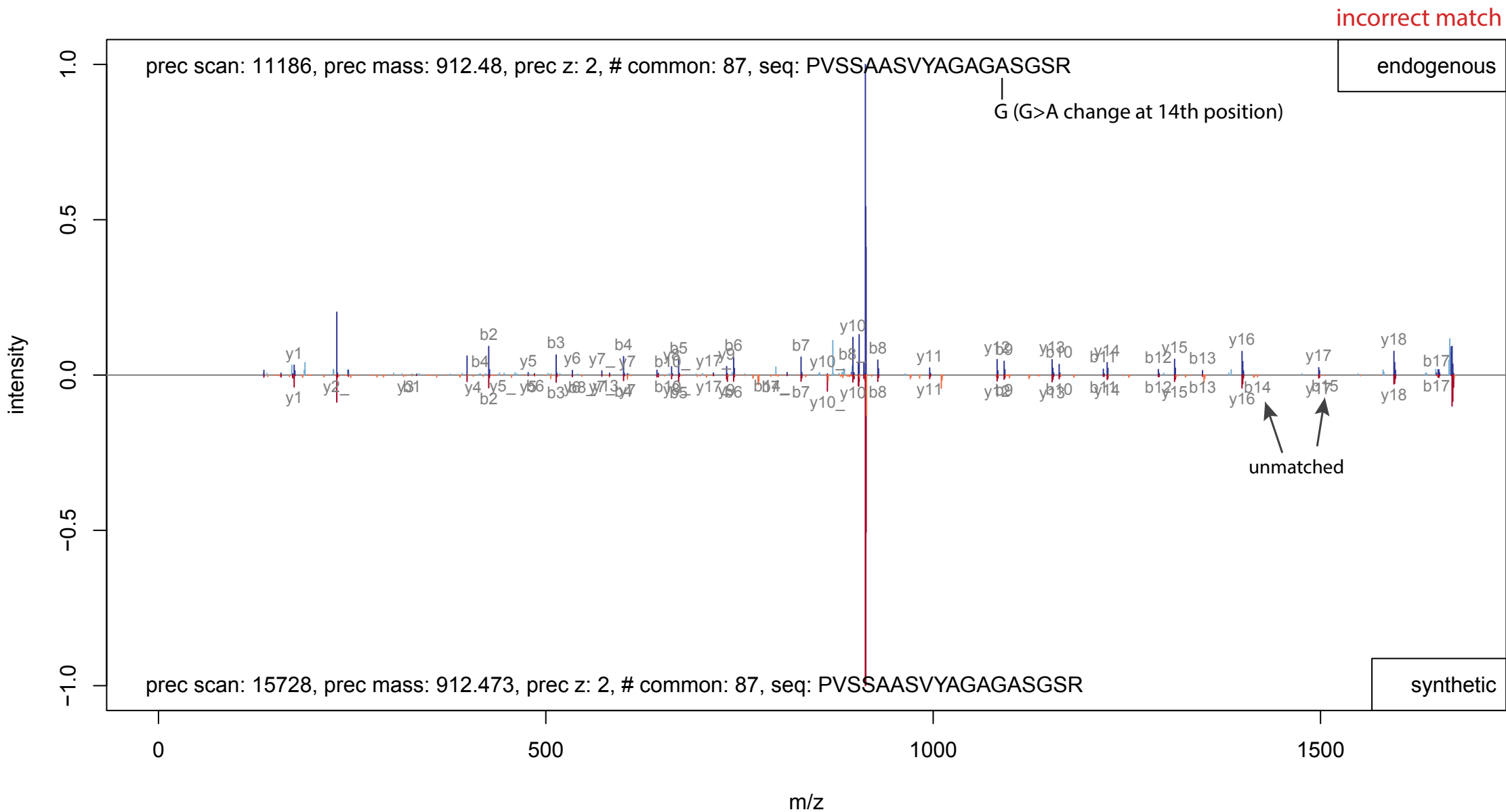

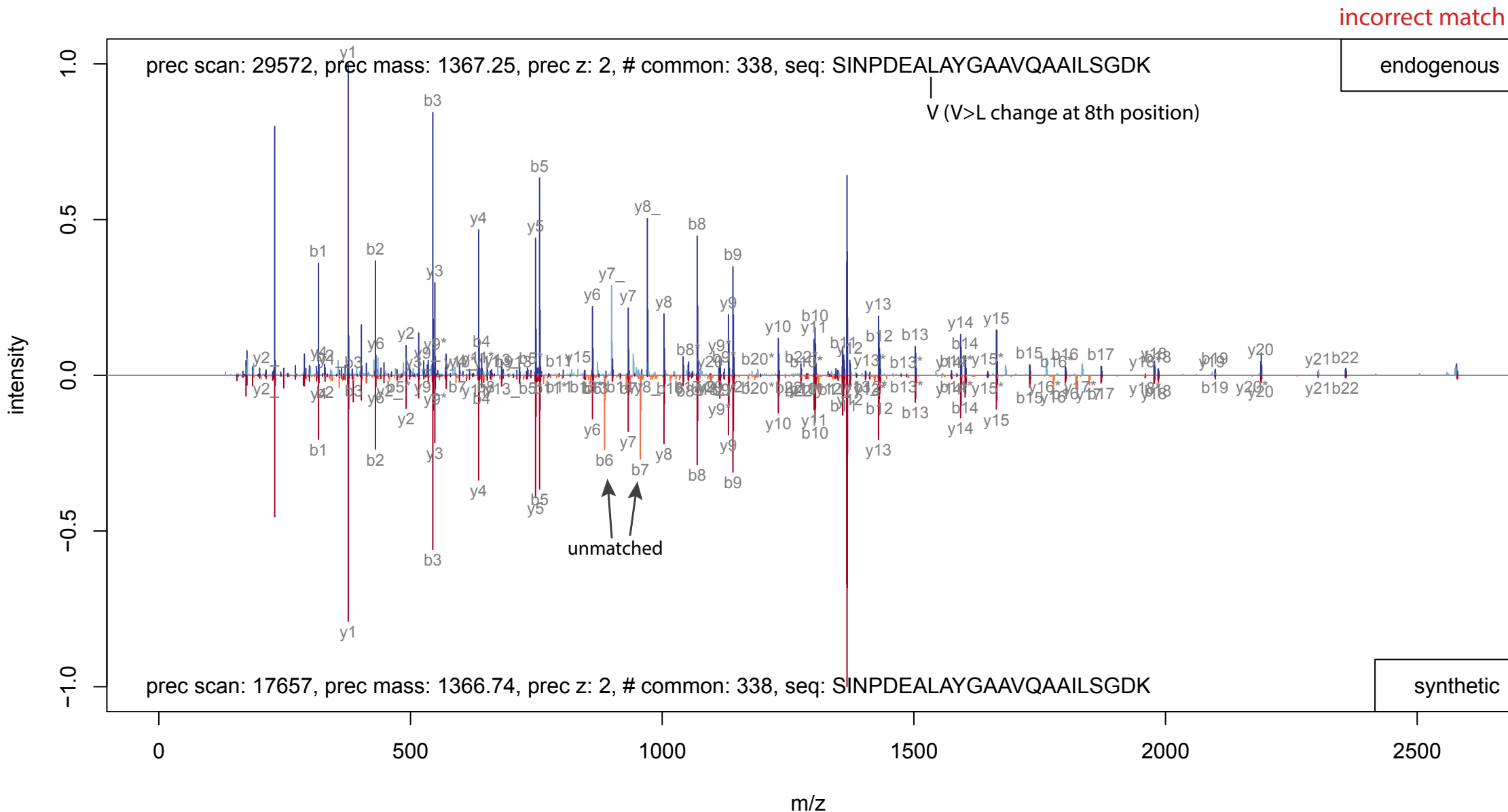

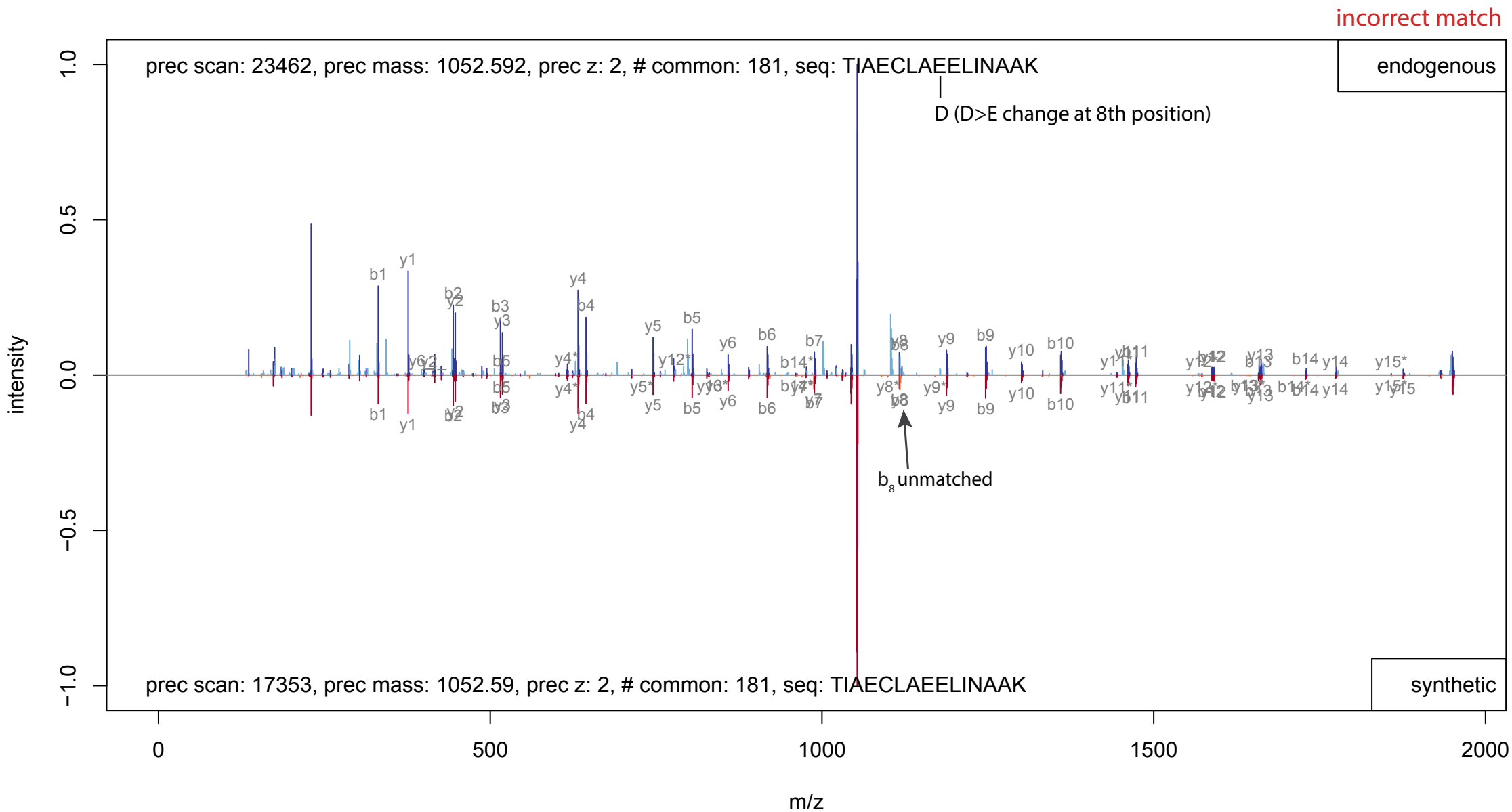

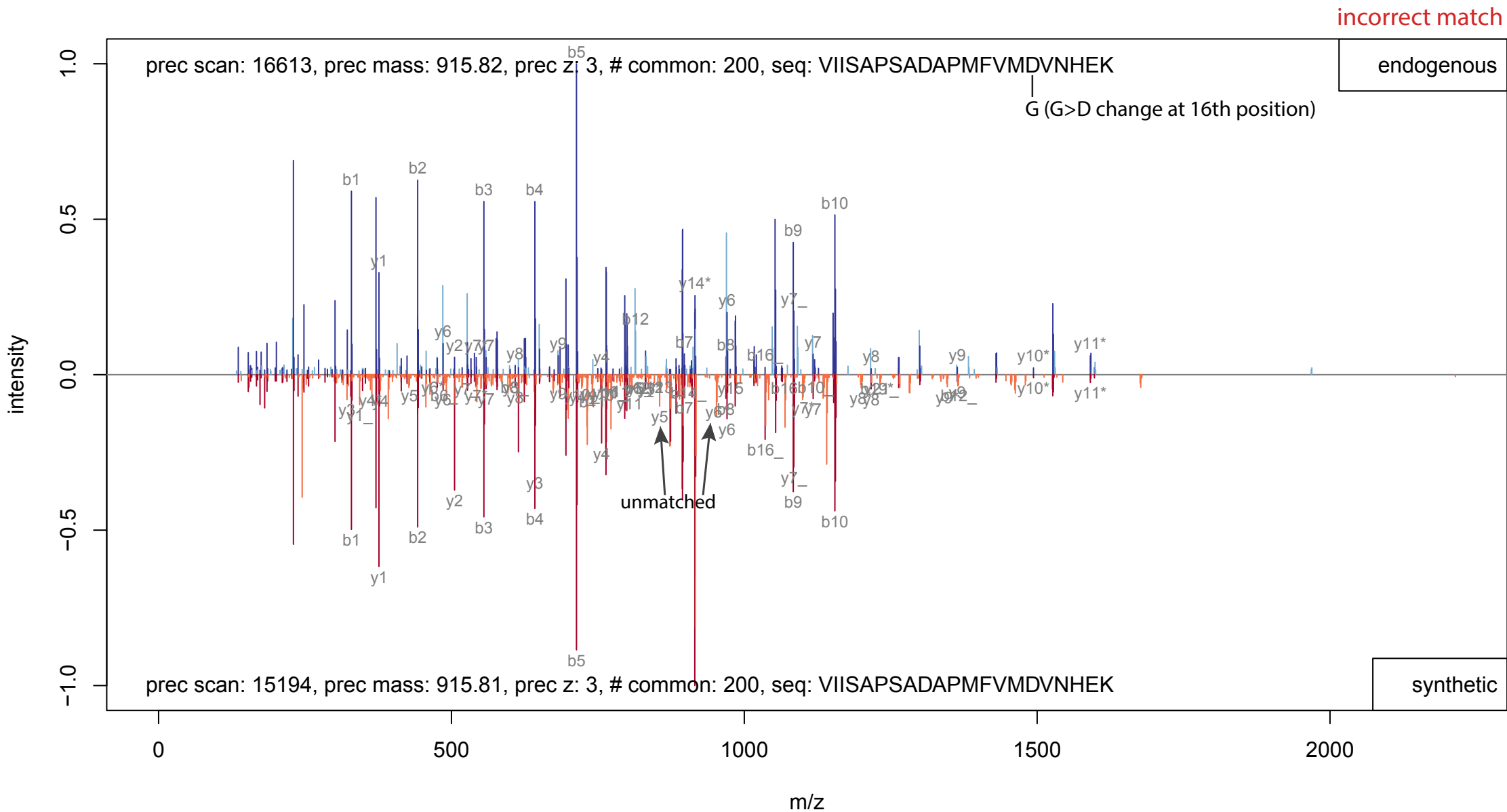

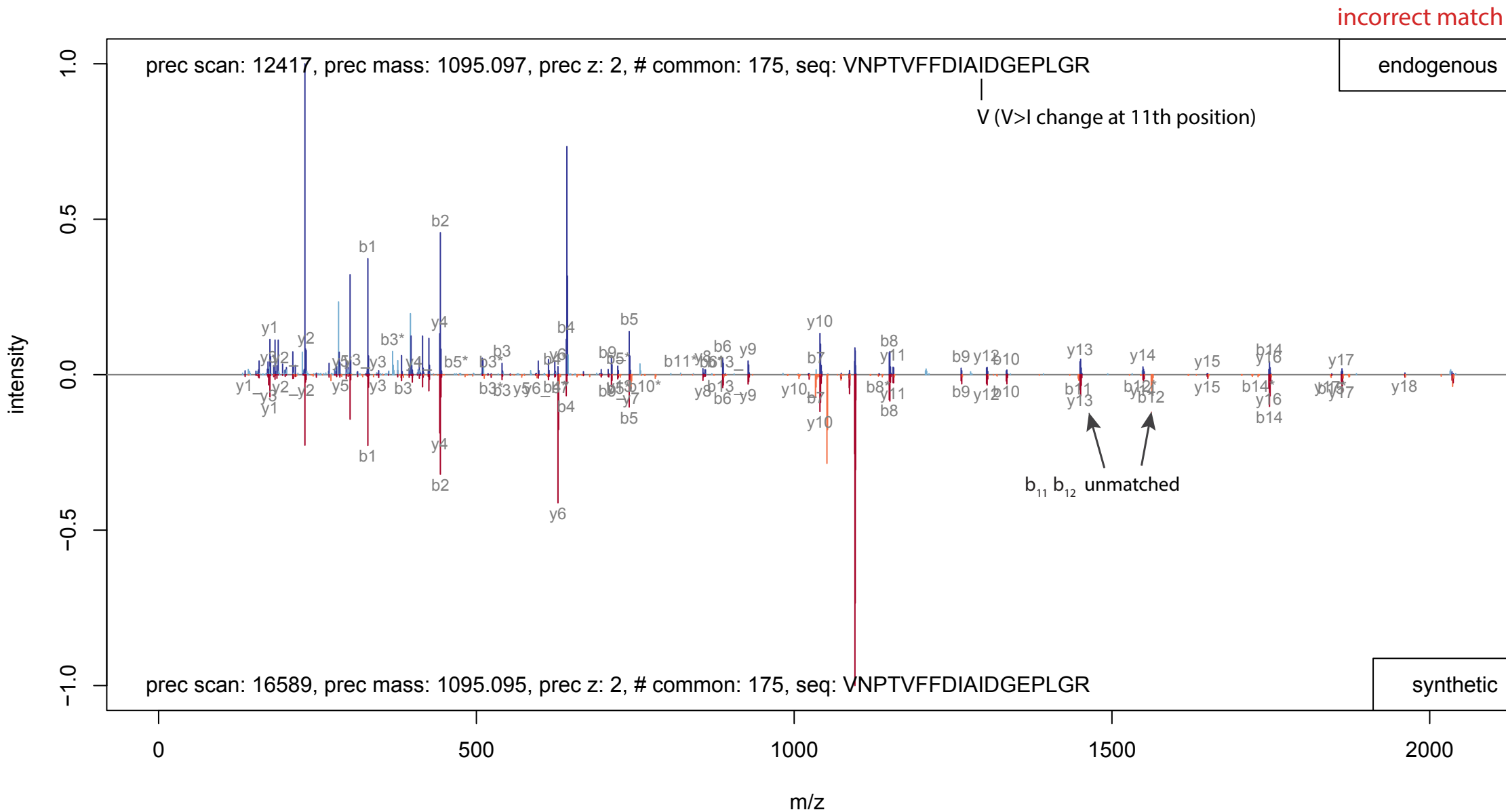

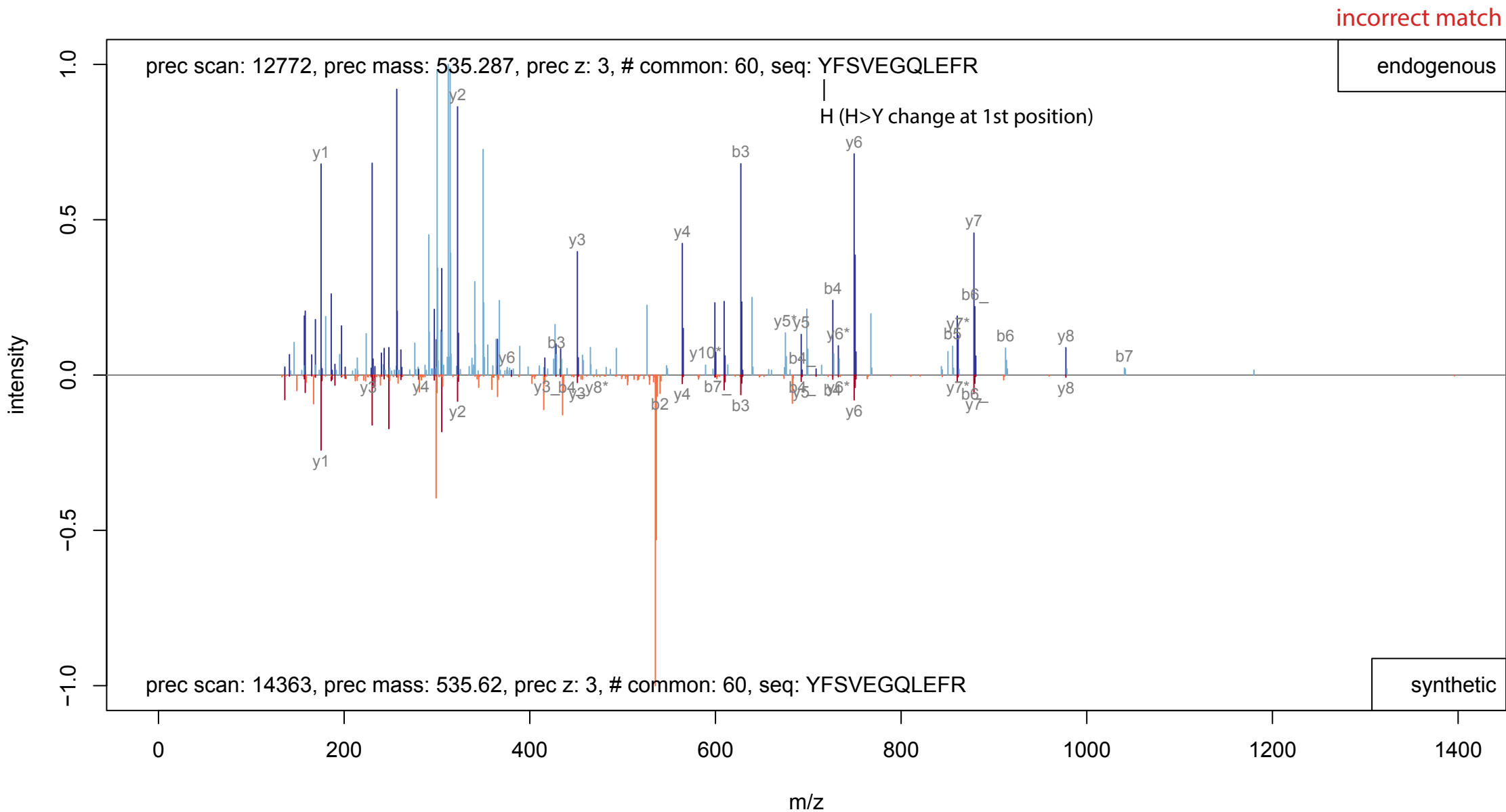

Incorrect match

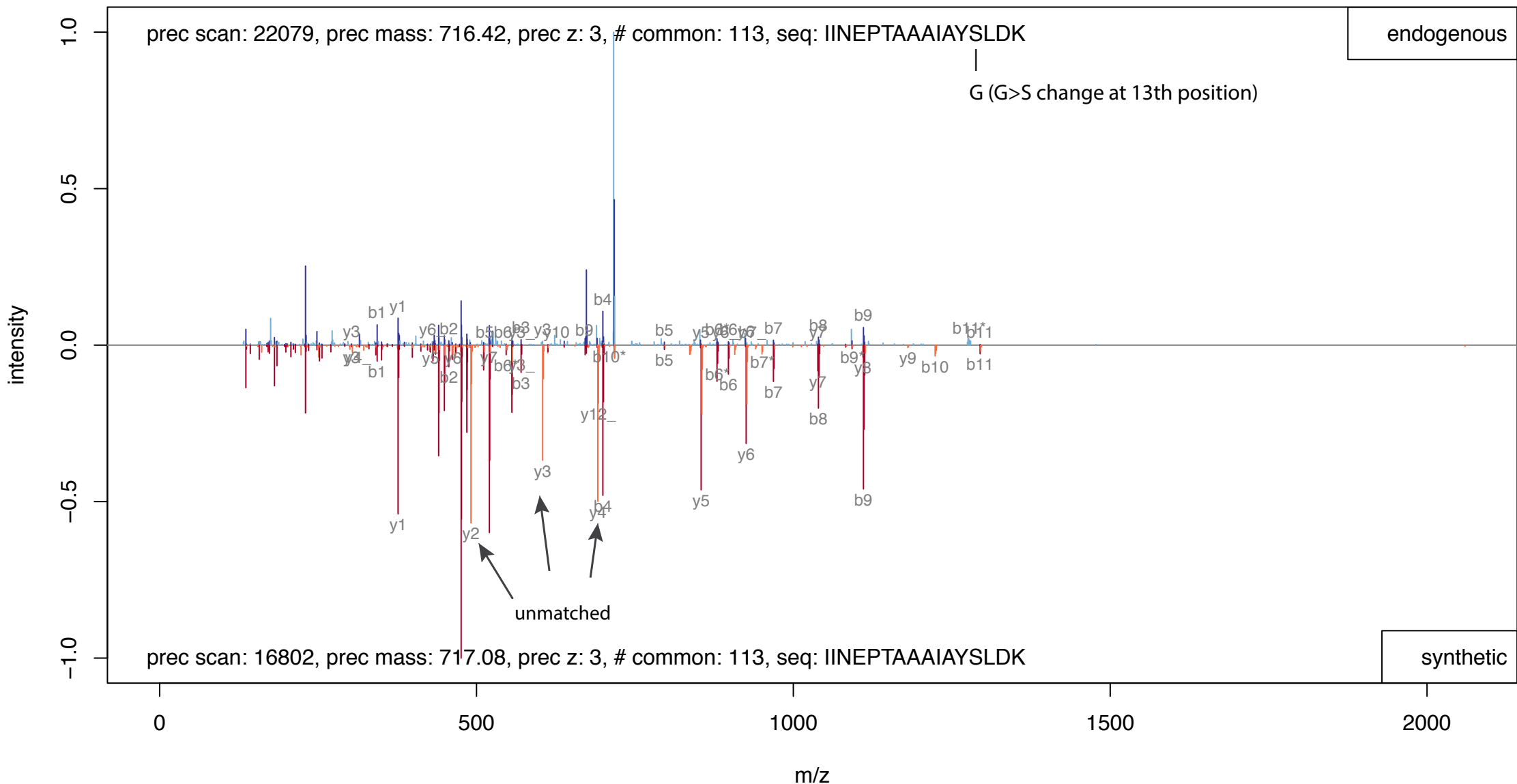

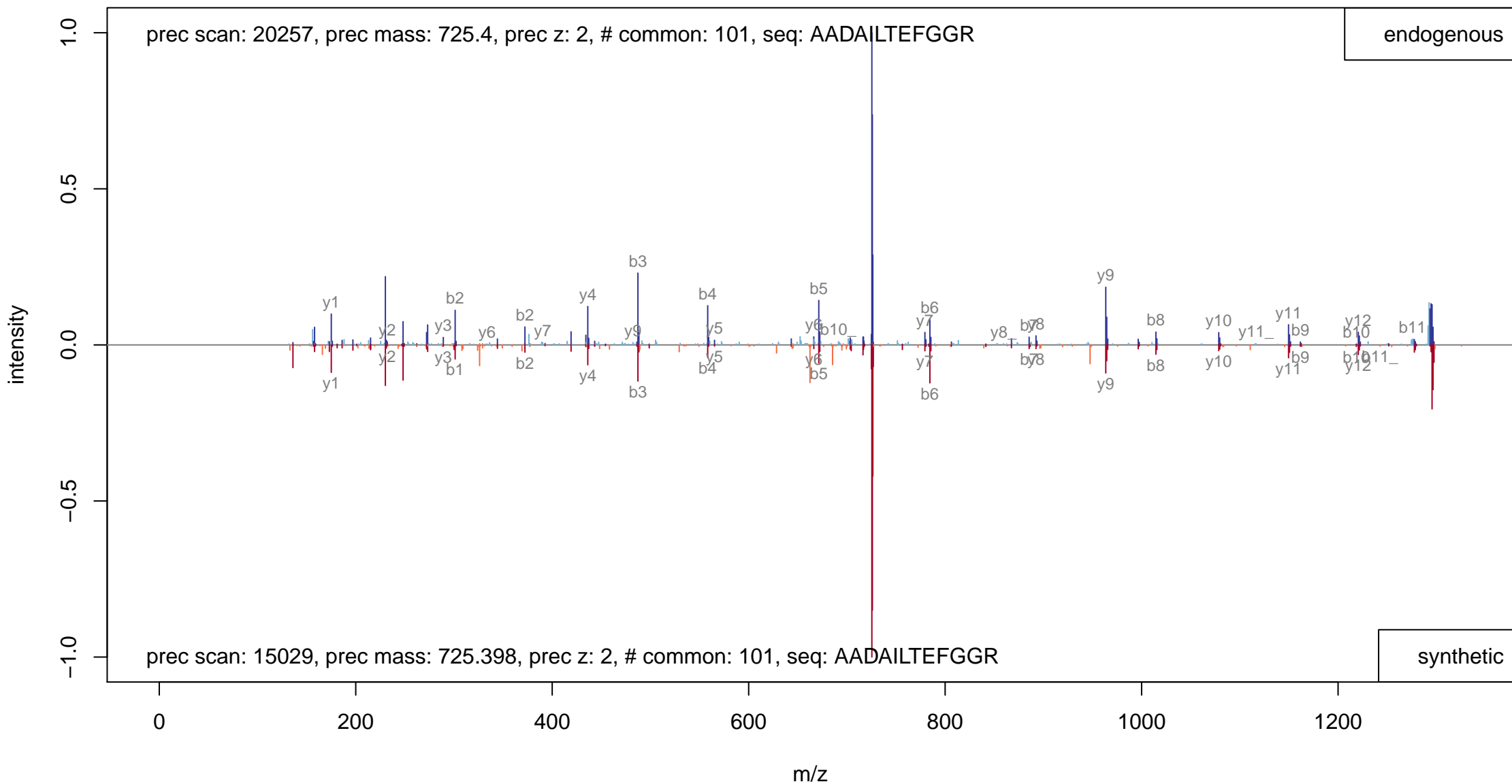

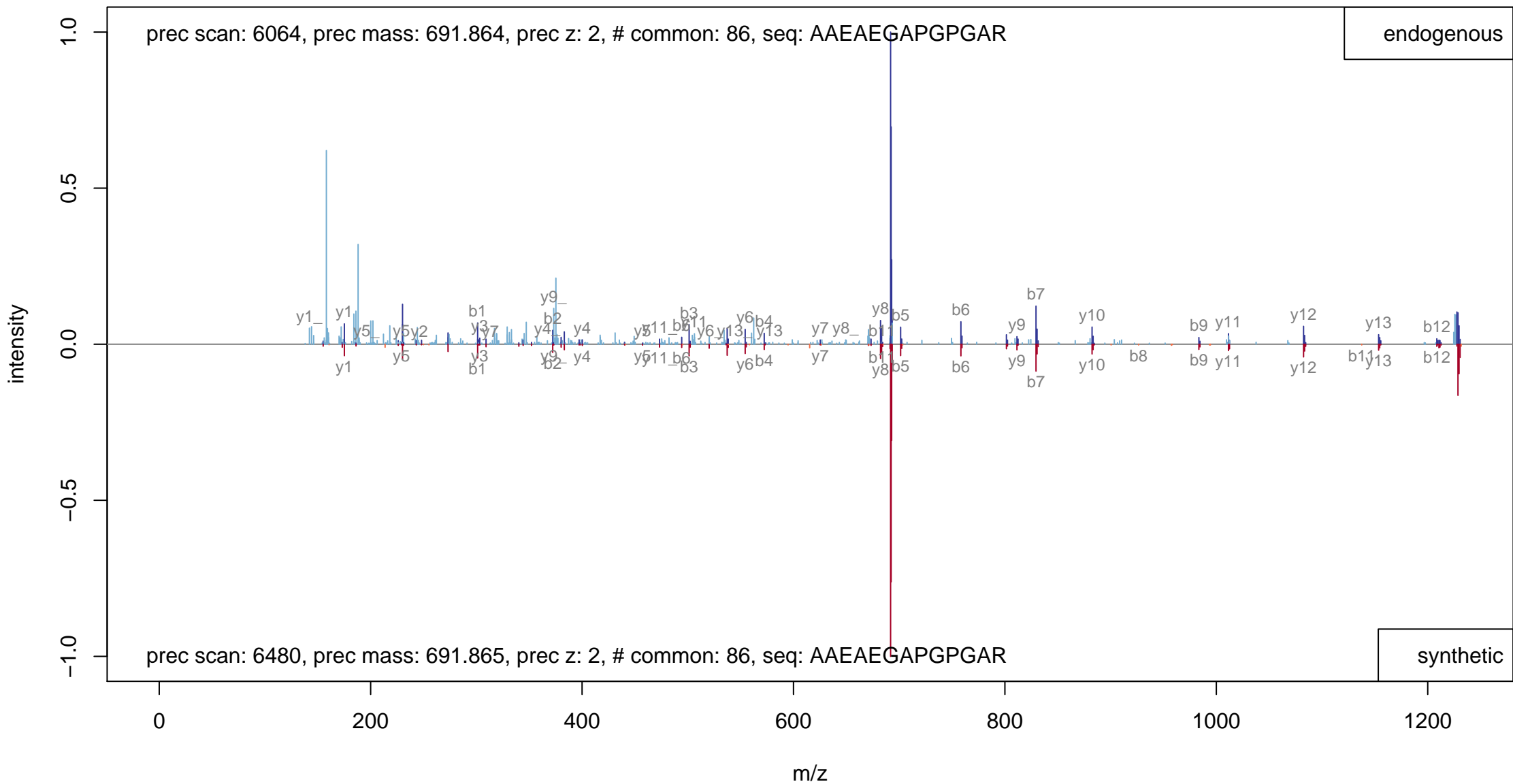

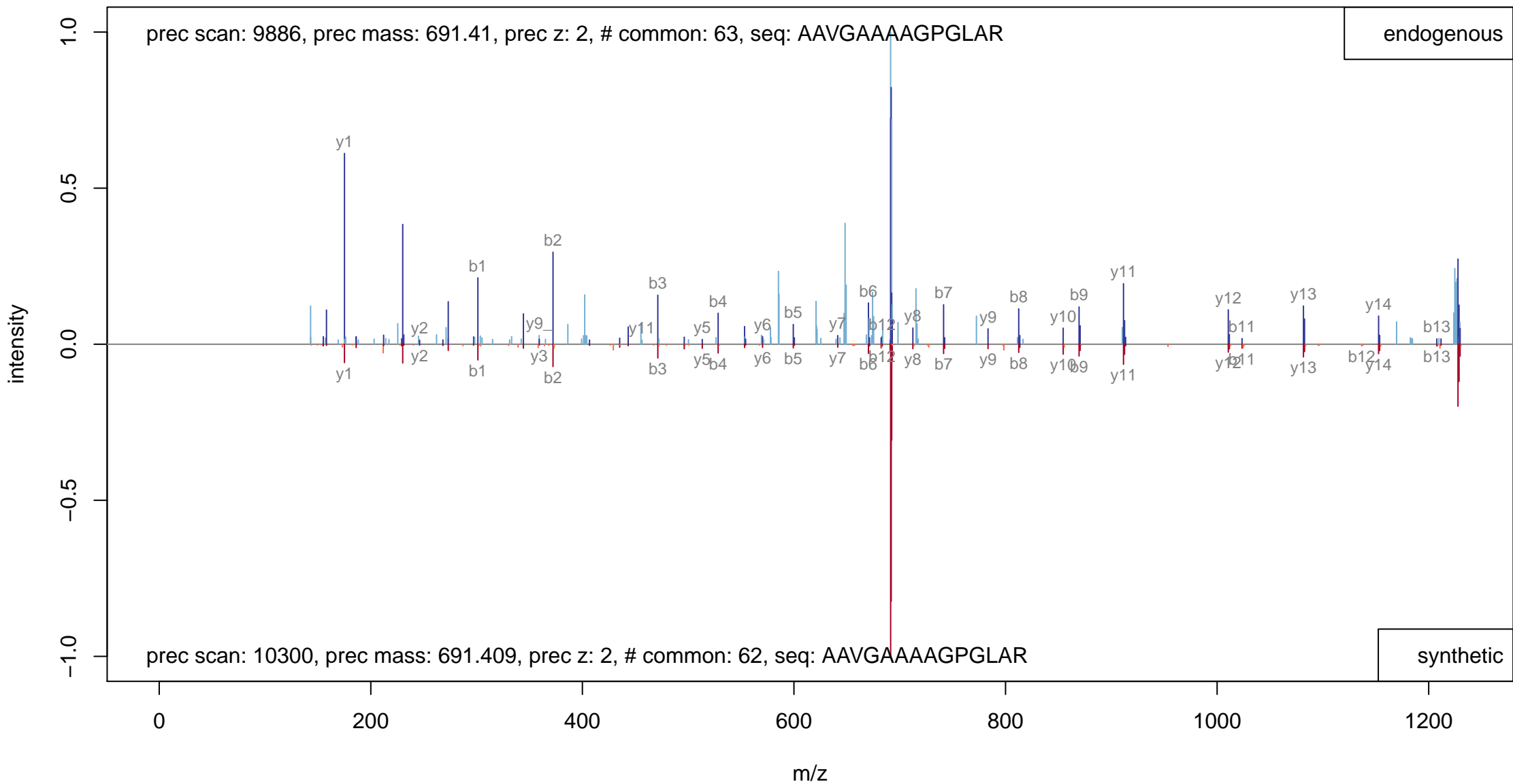

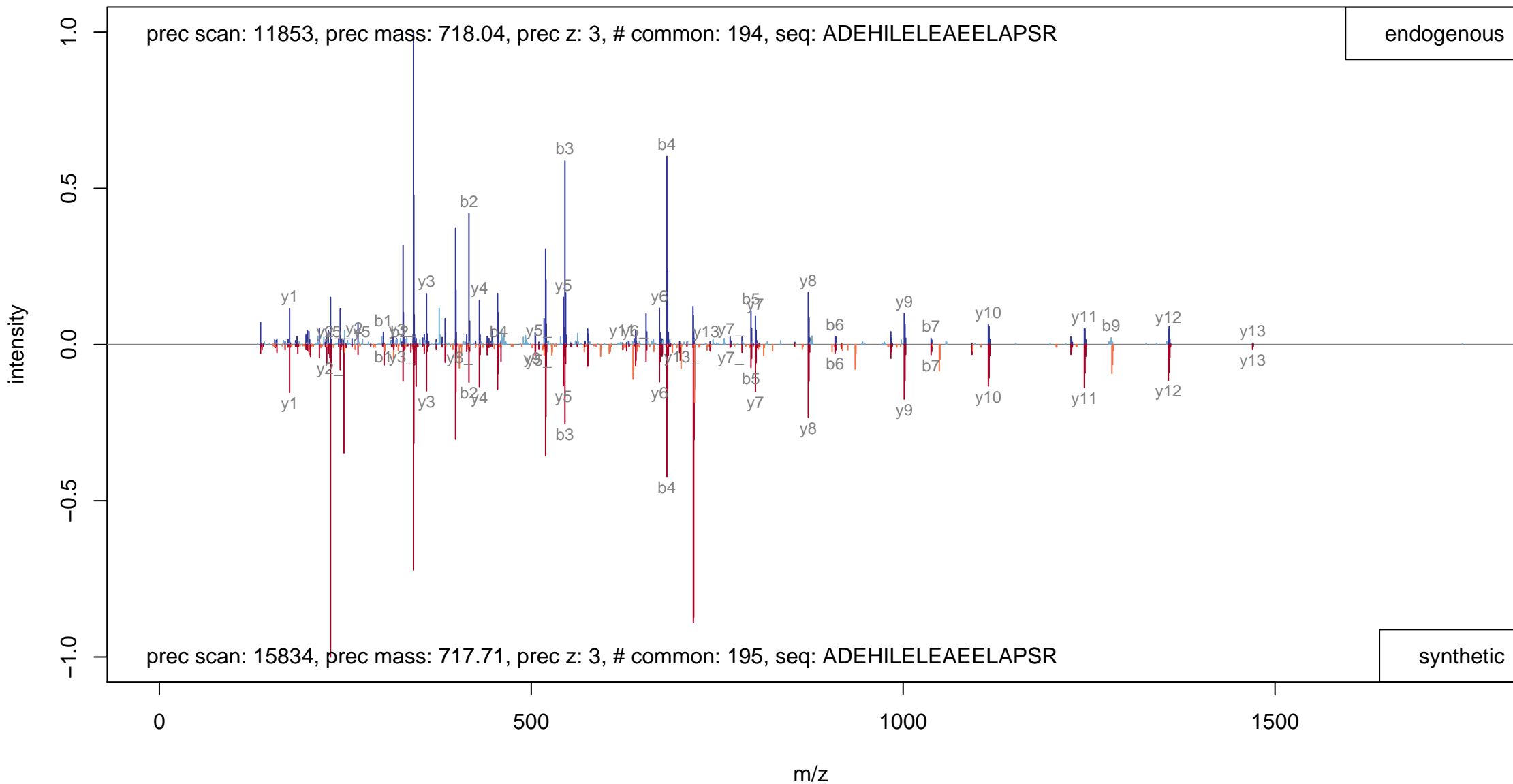

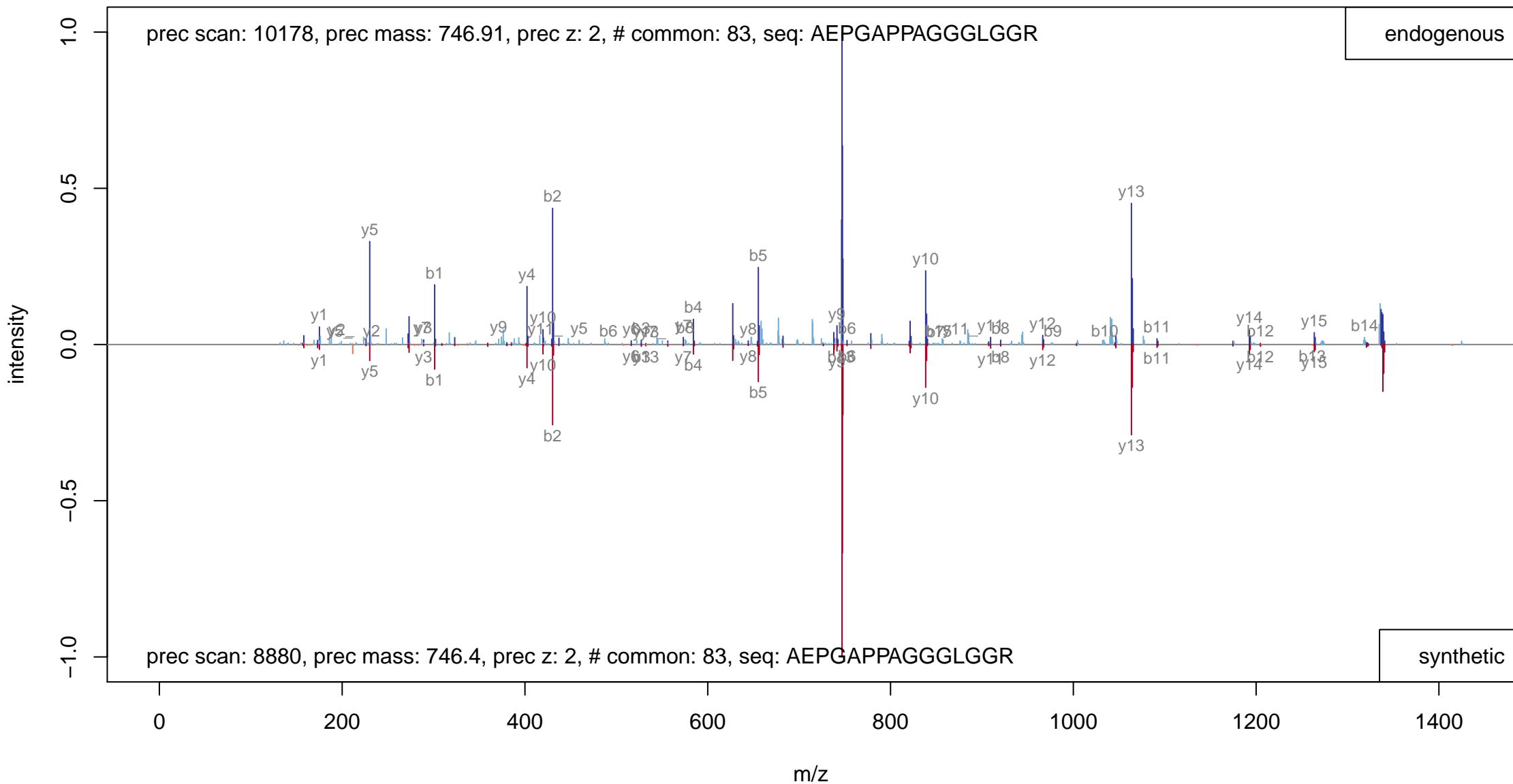

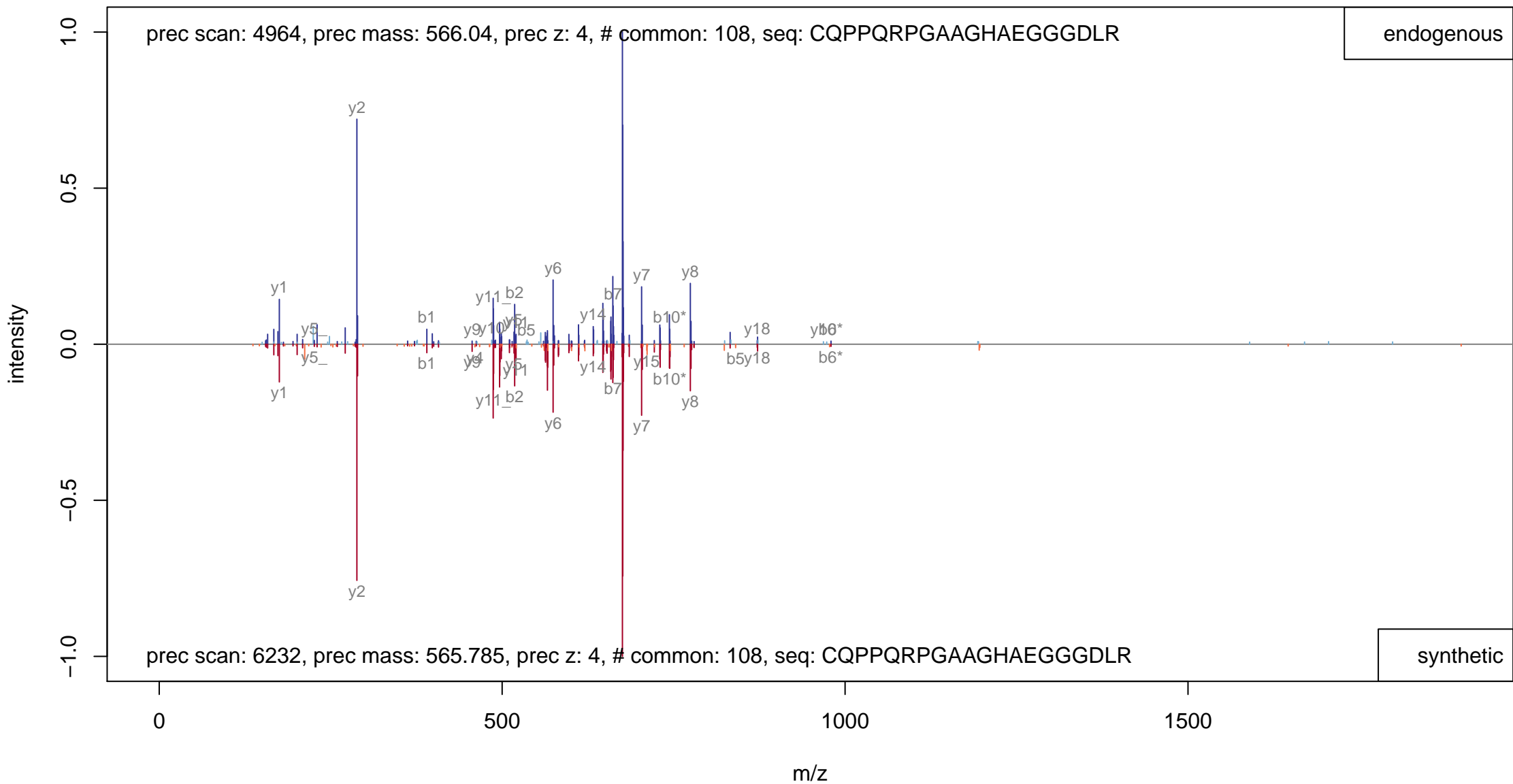

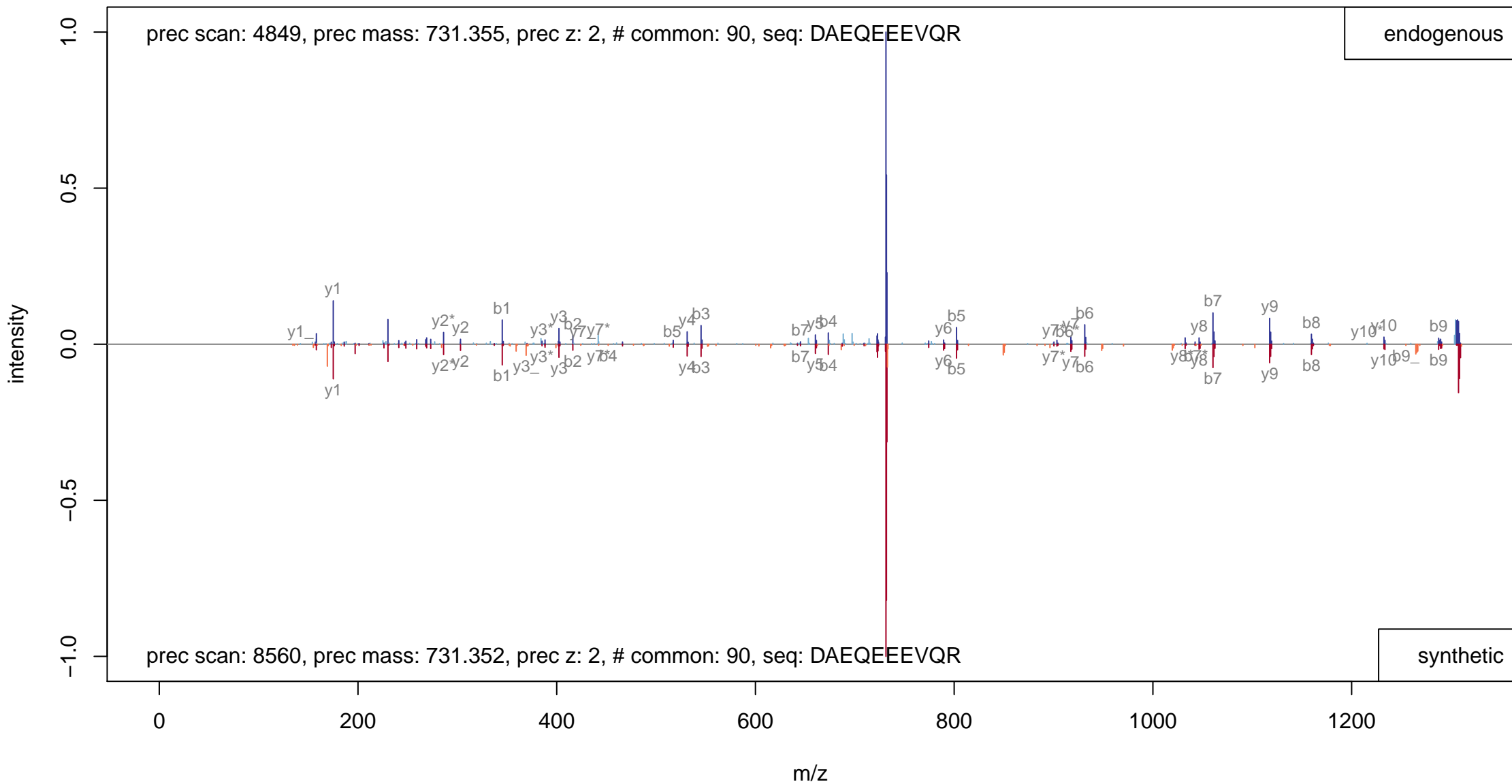

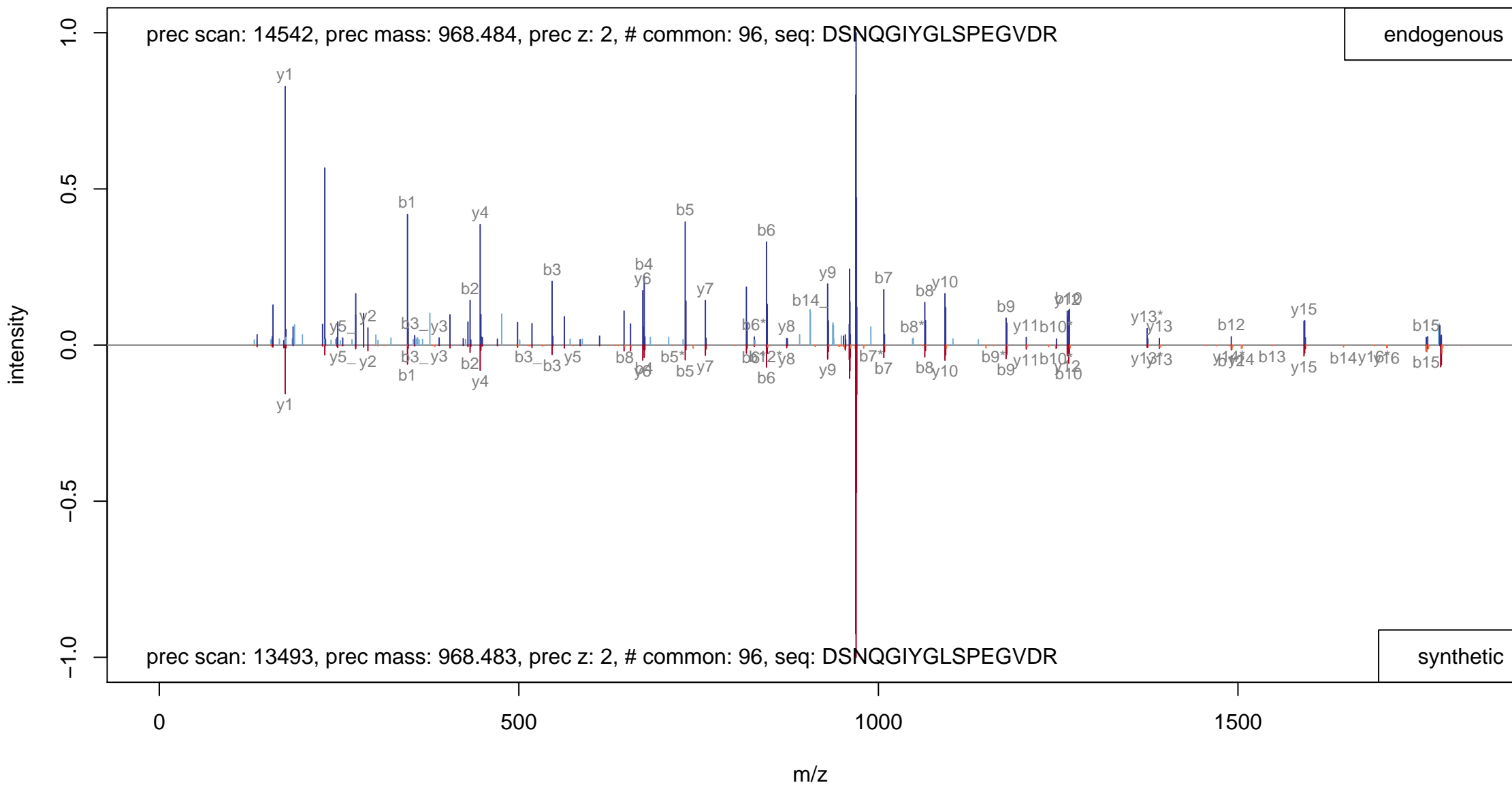

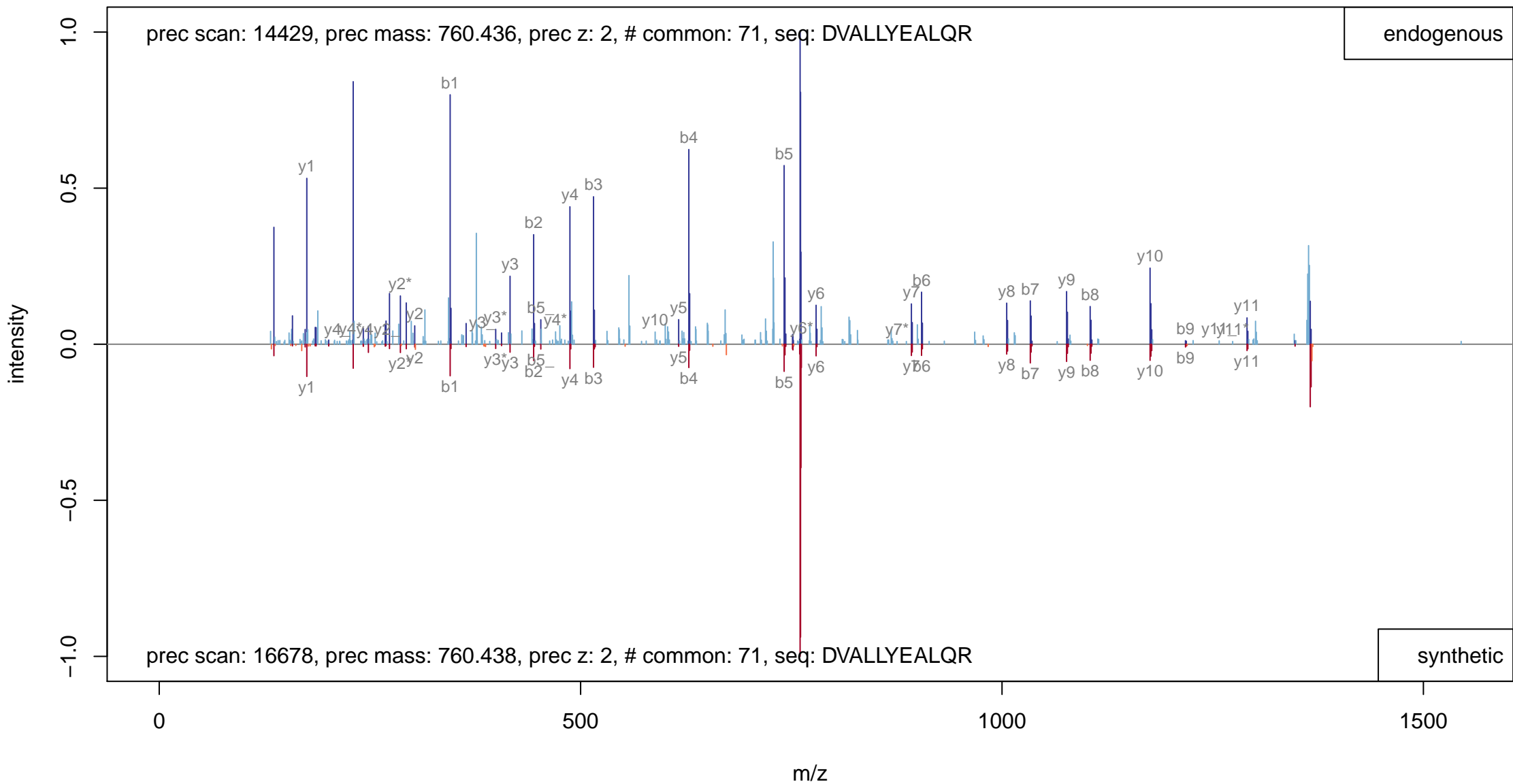

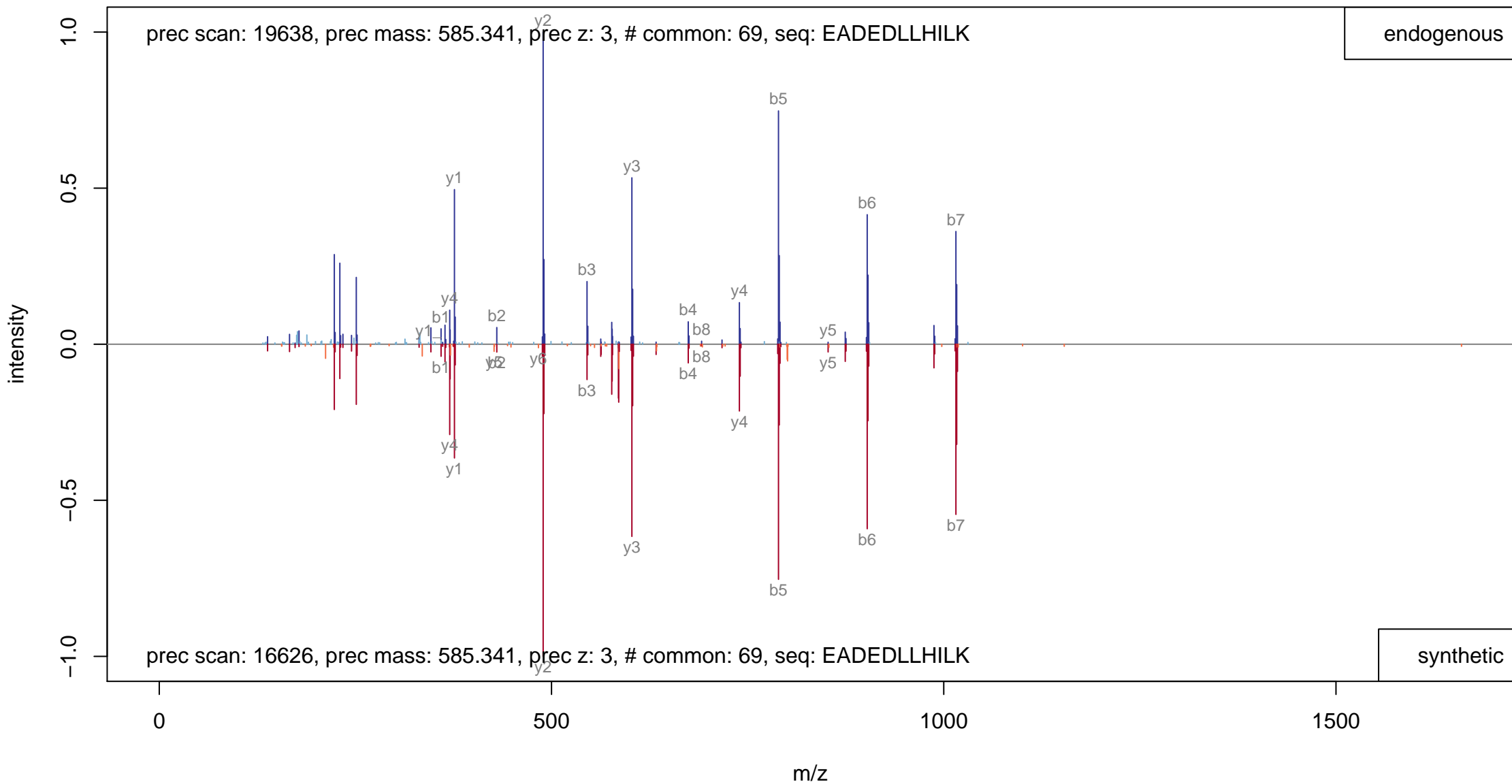

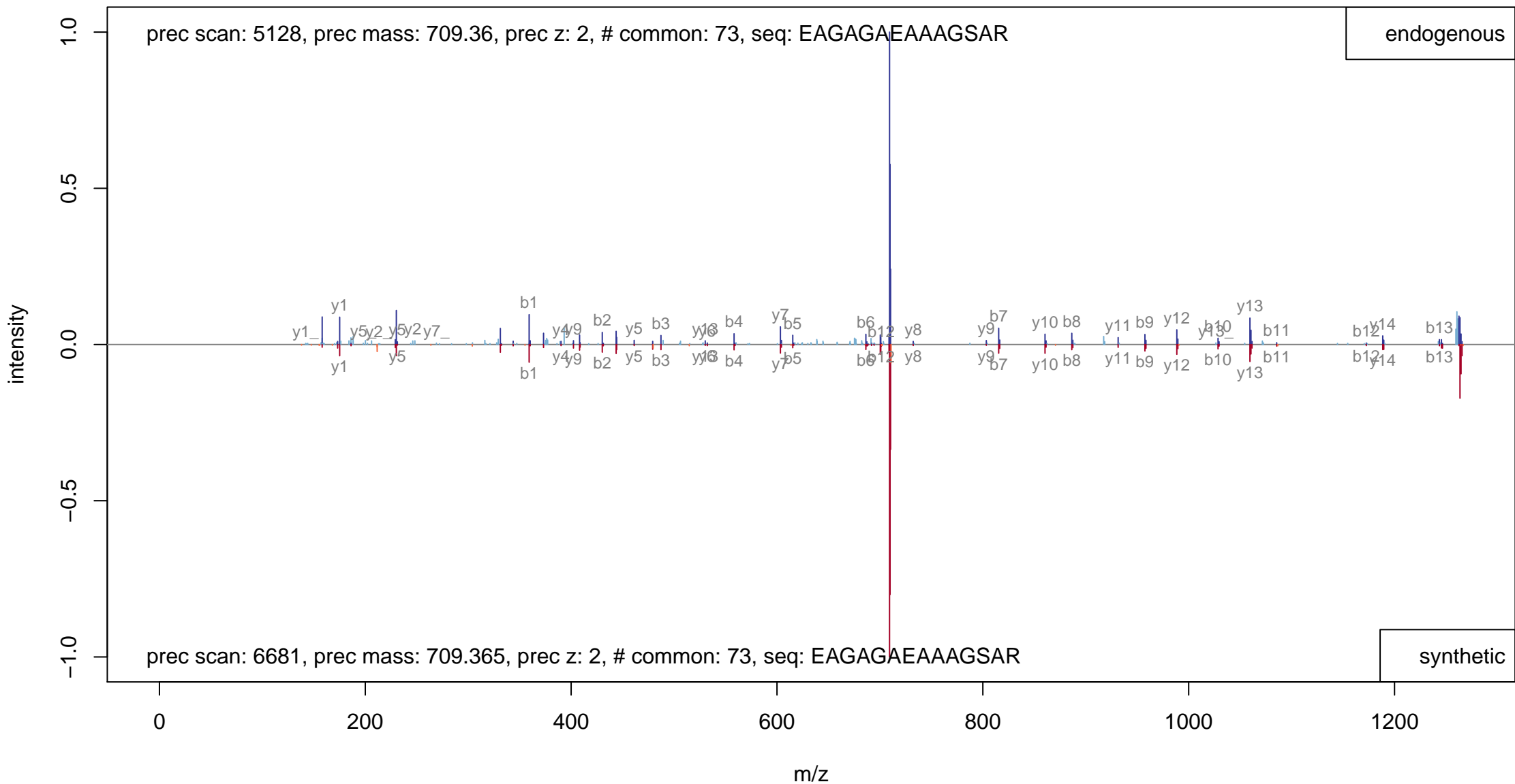

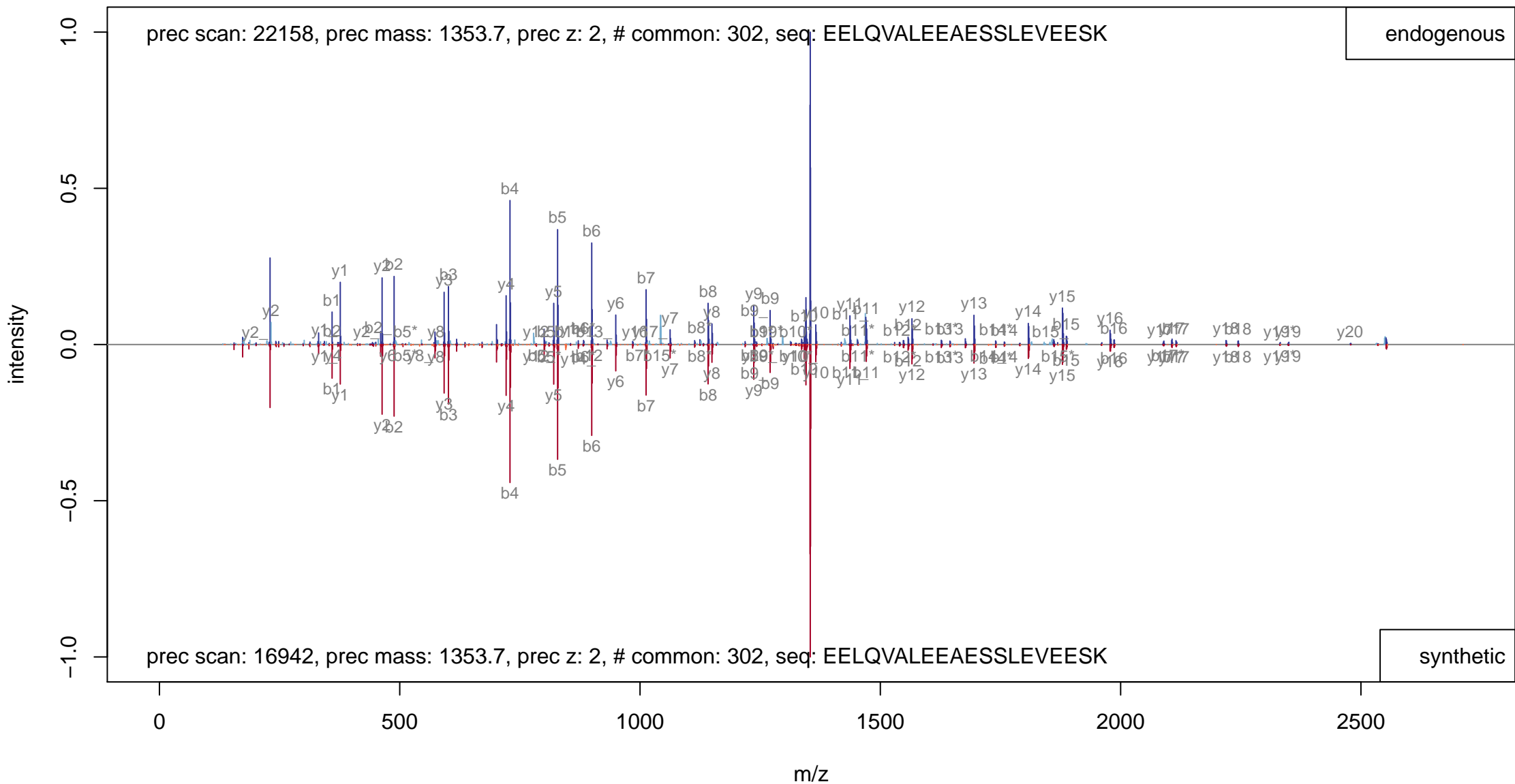

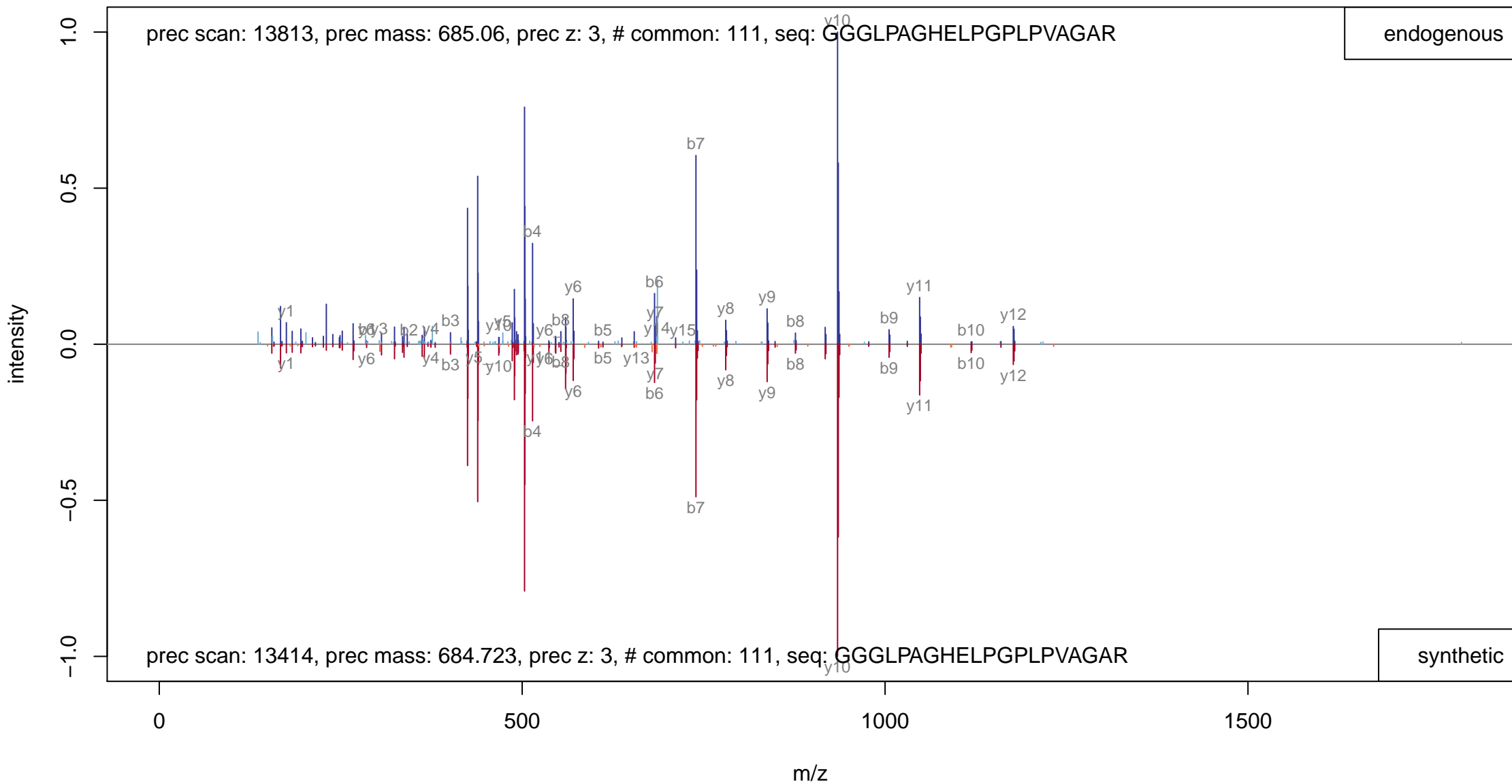

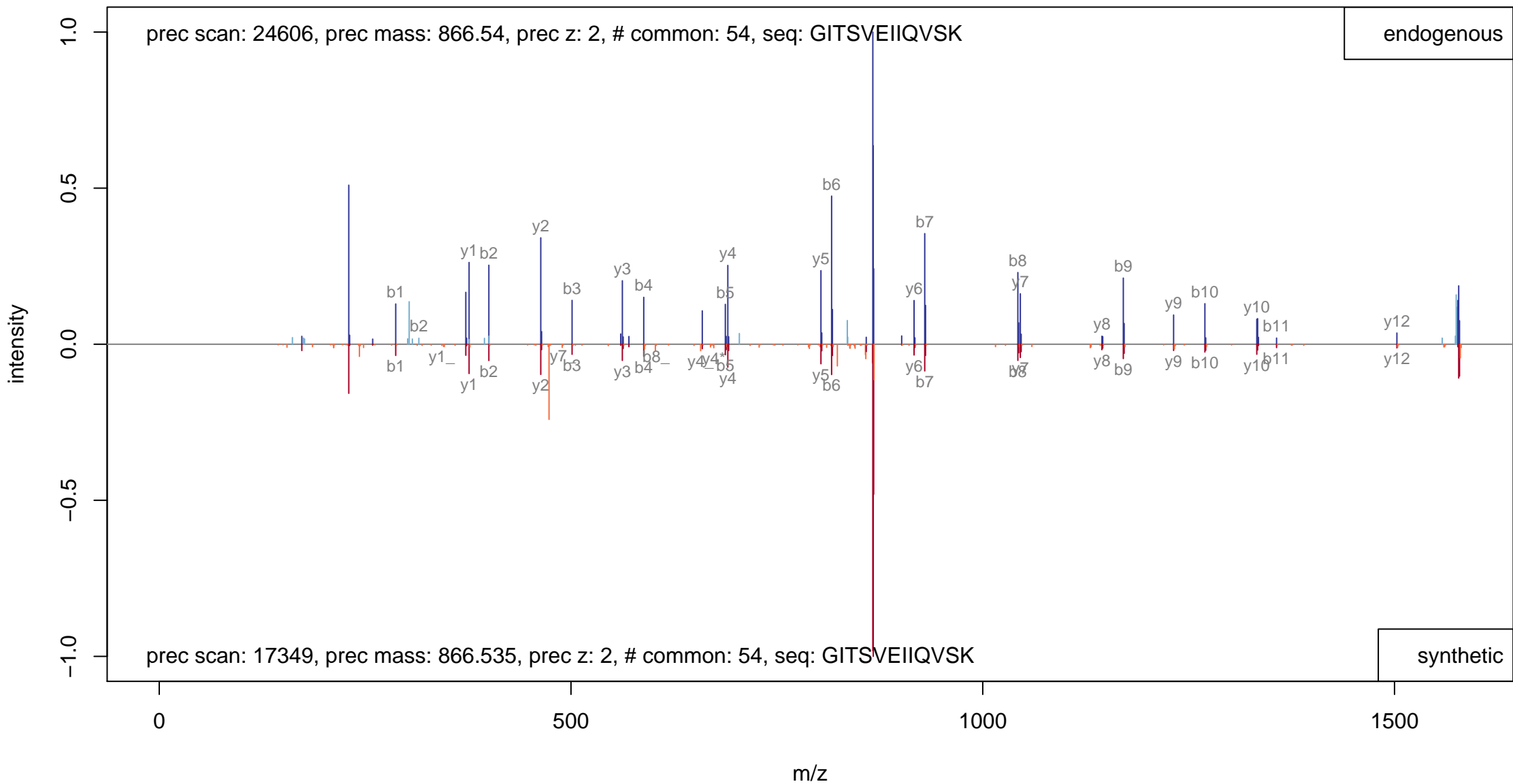

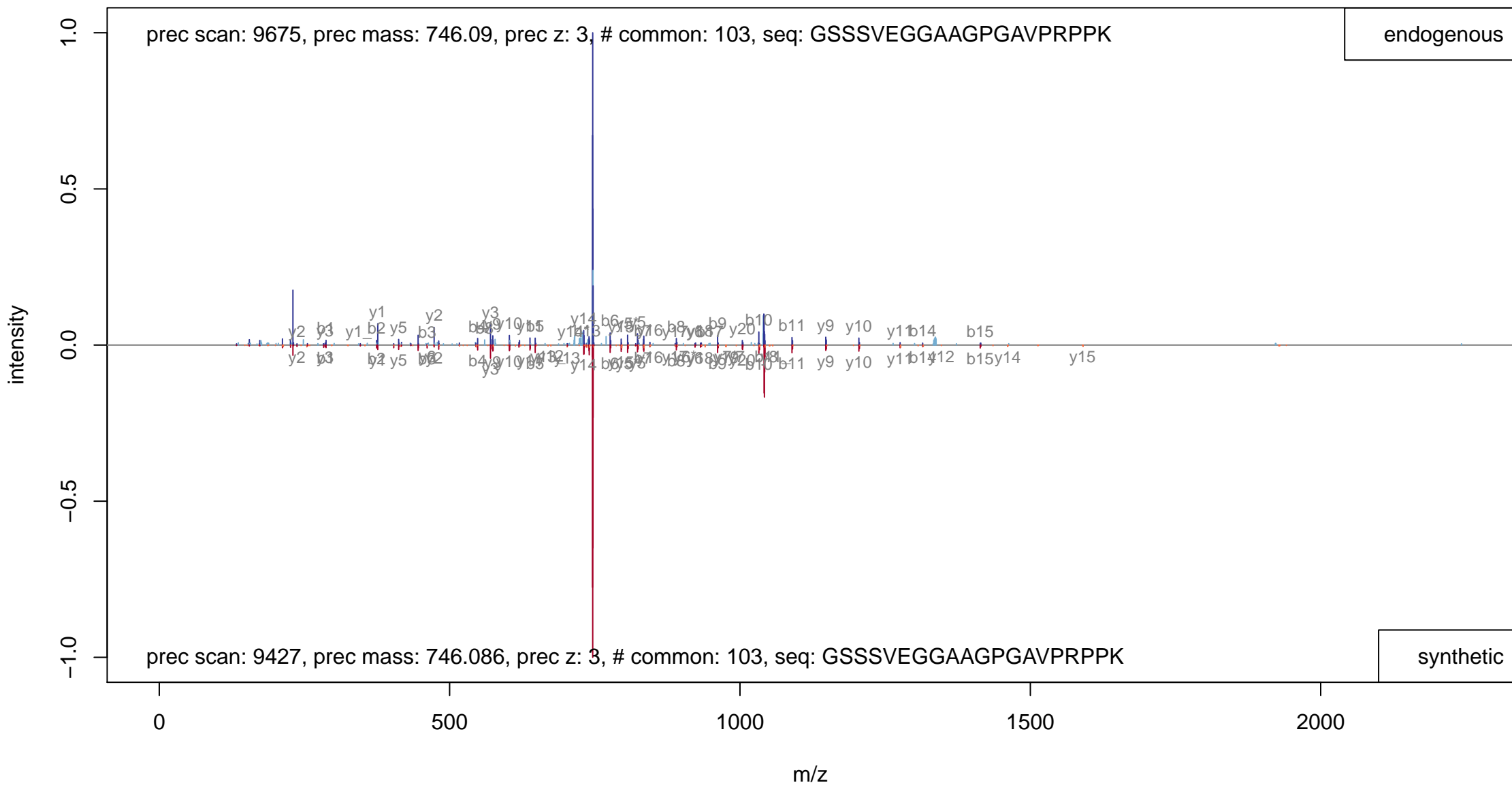

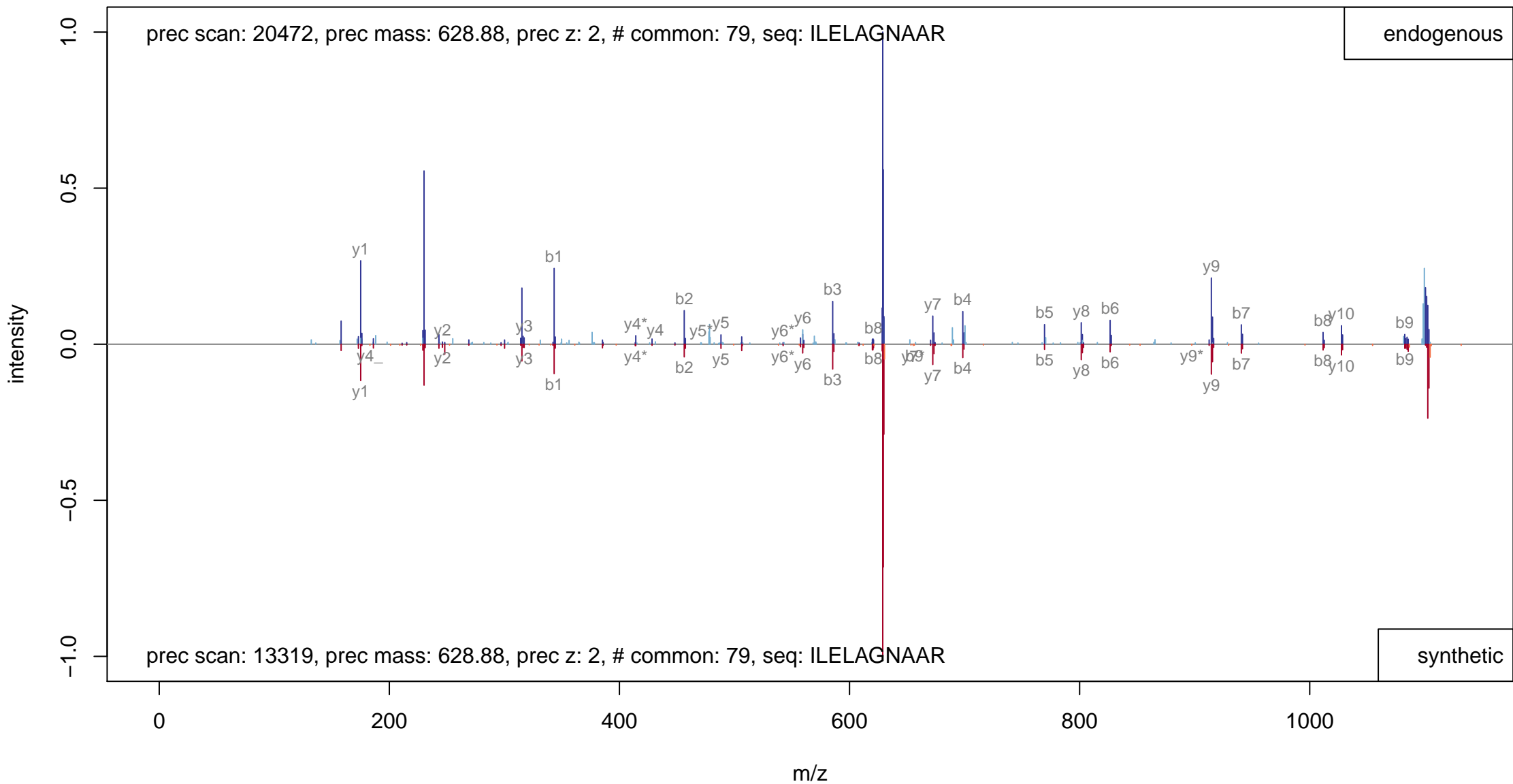

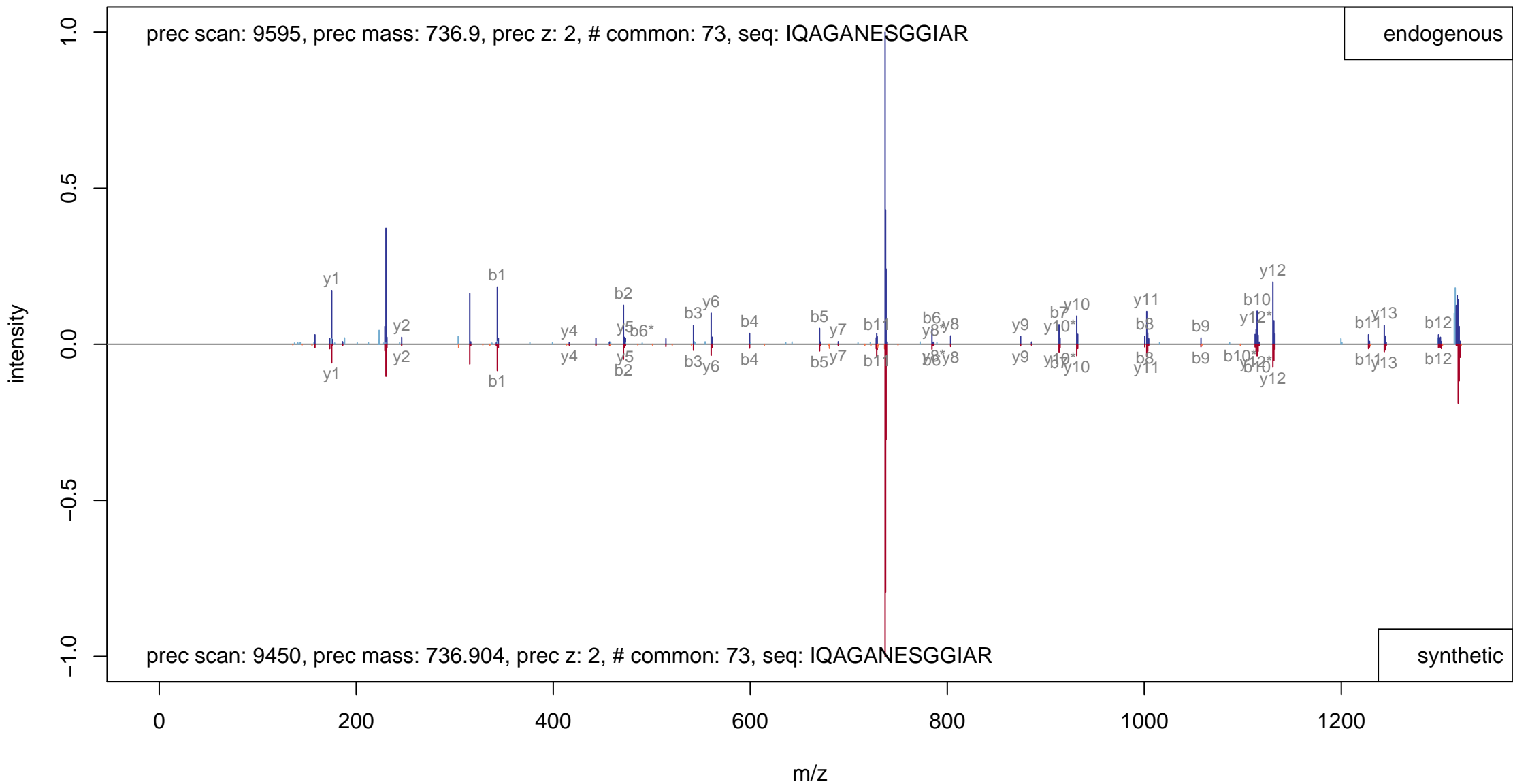

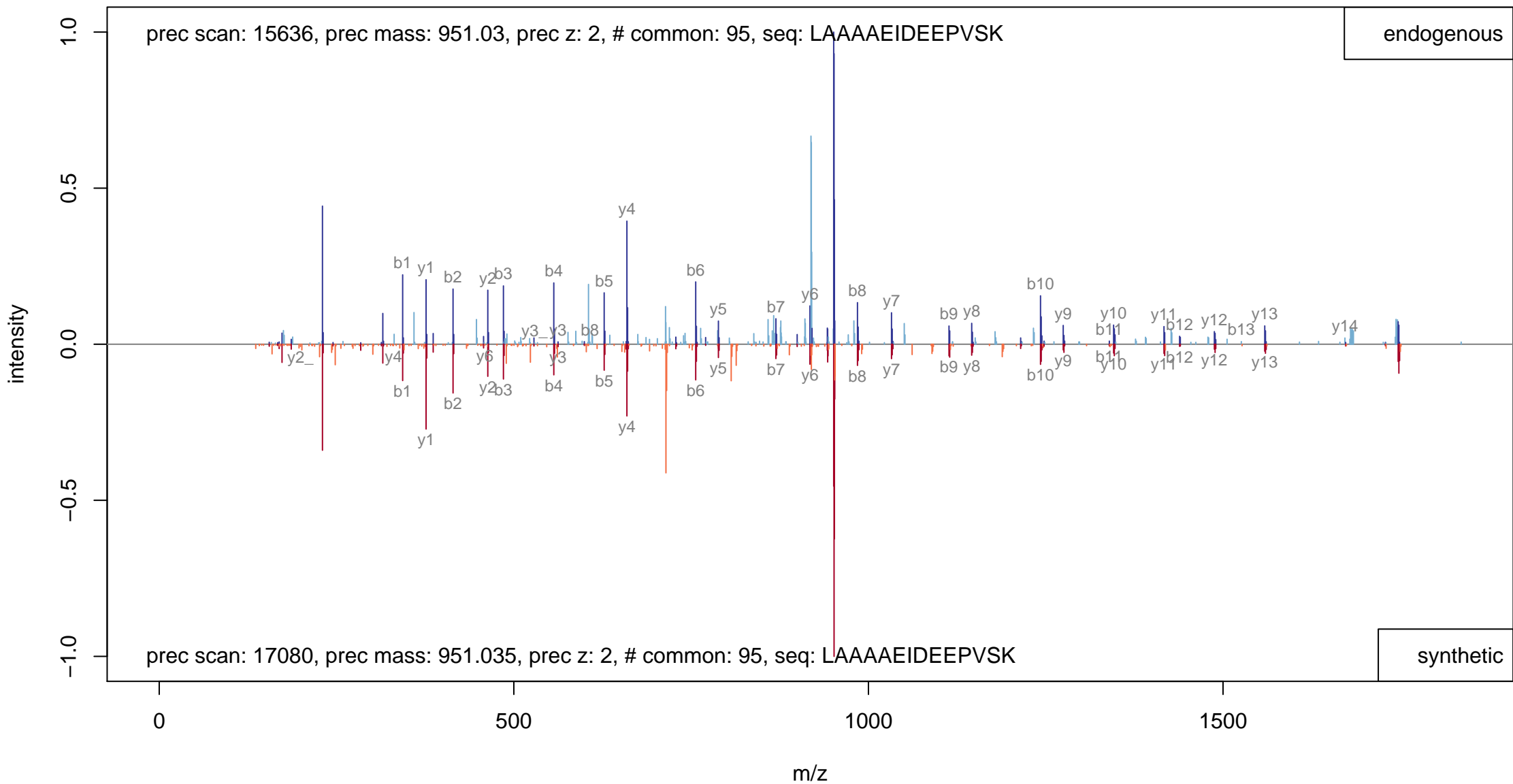

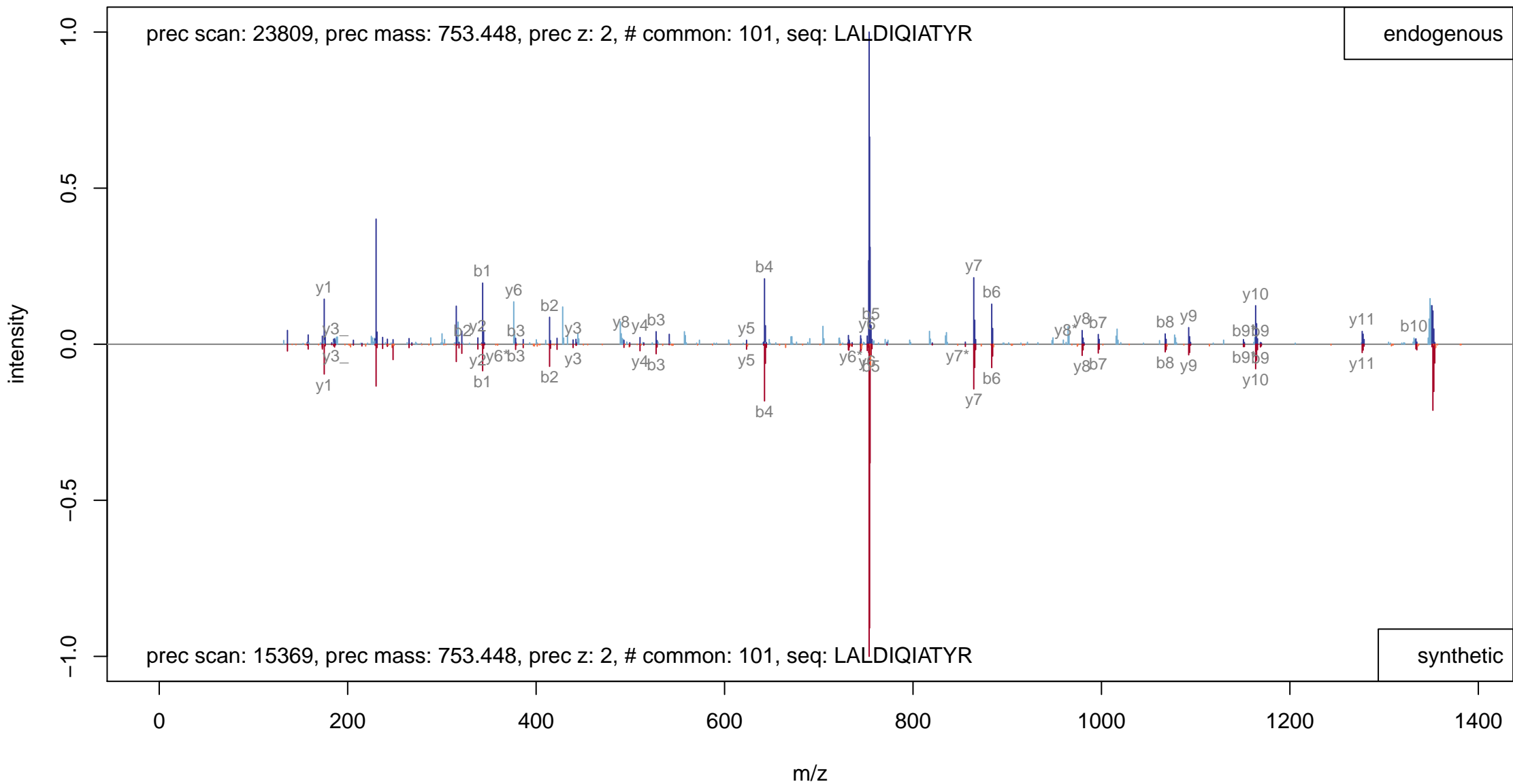

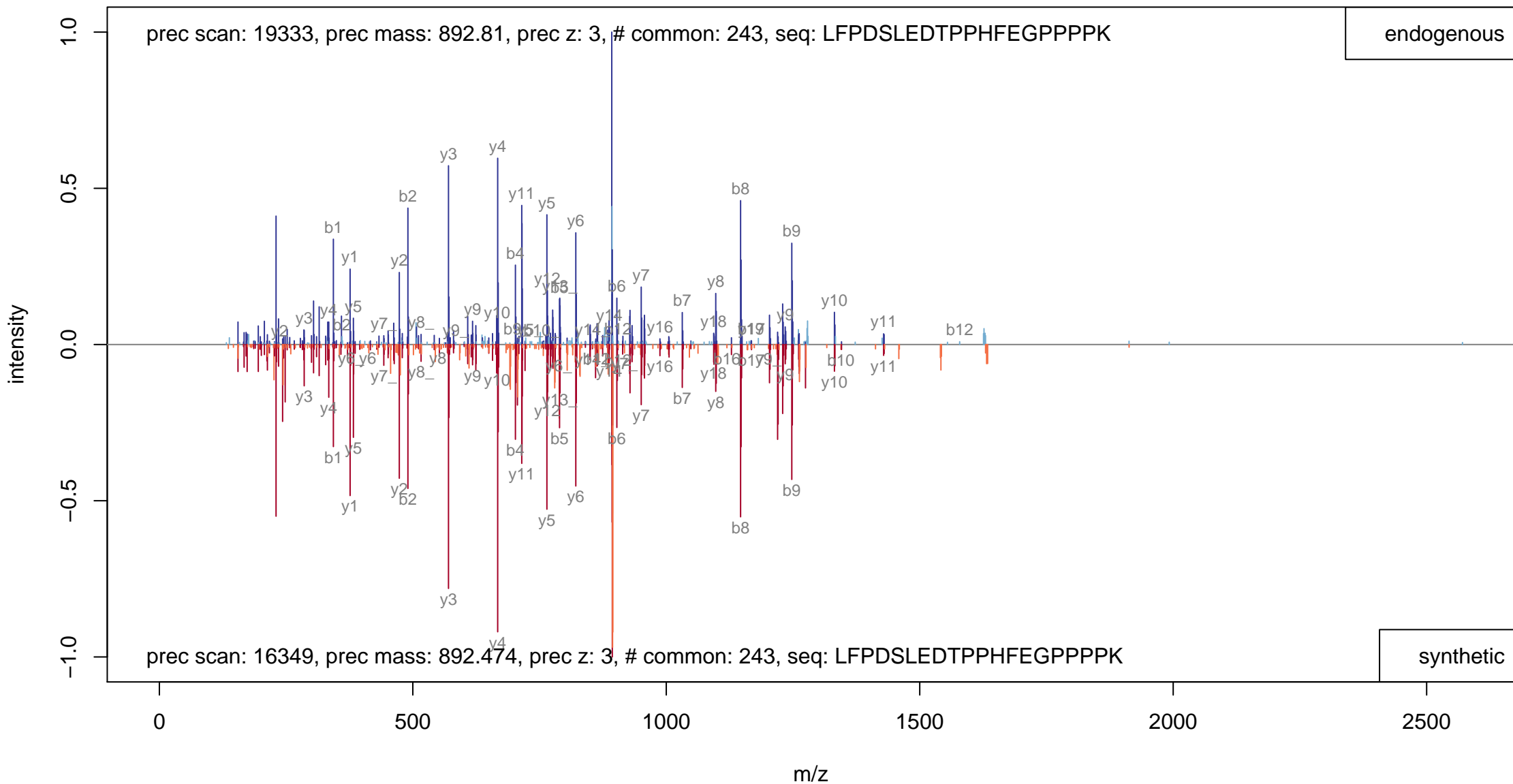

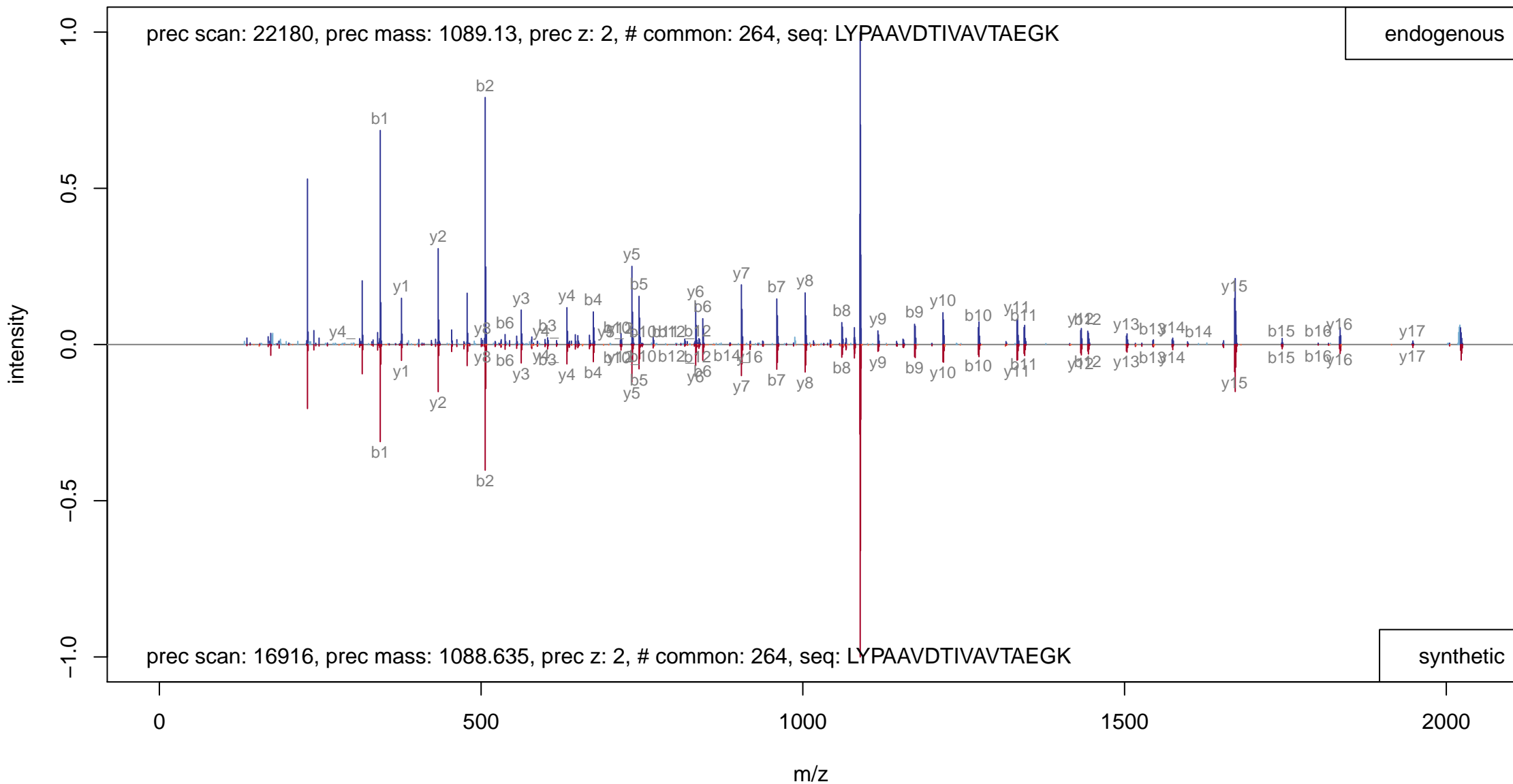

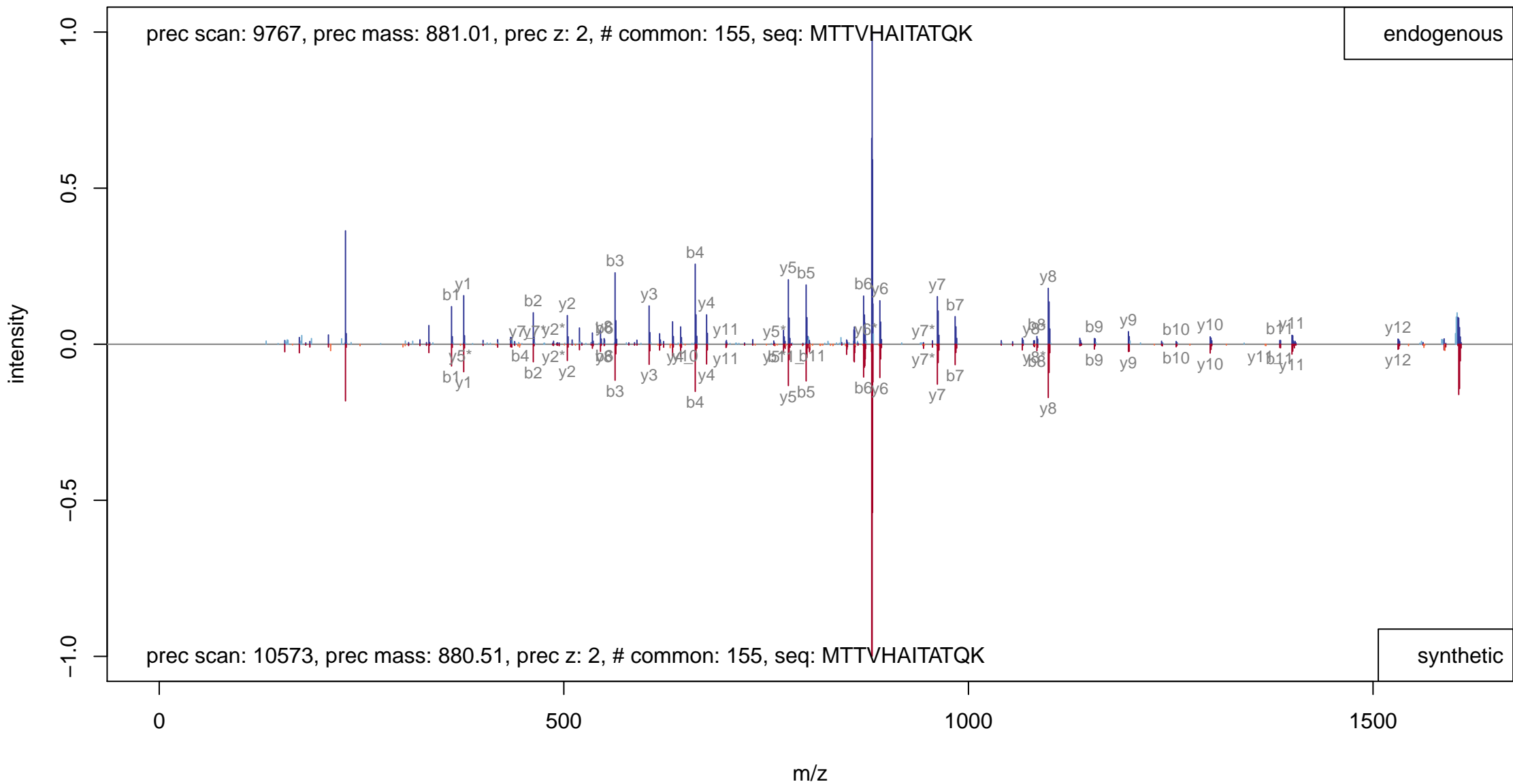

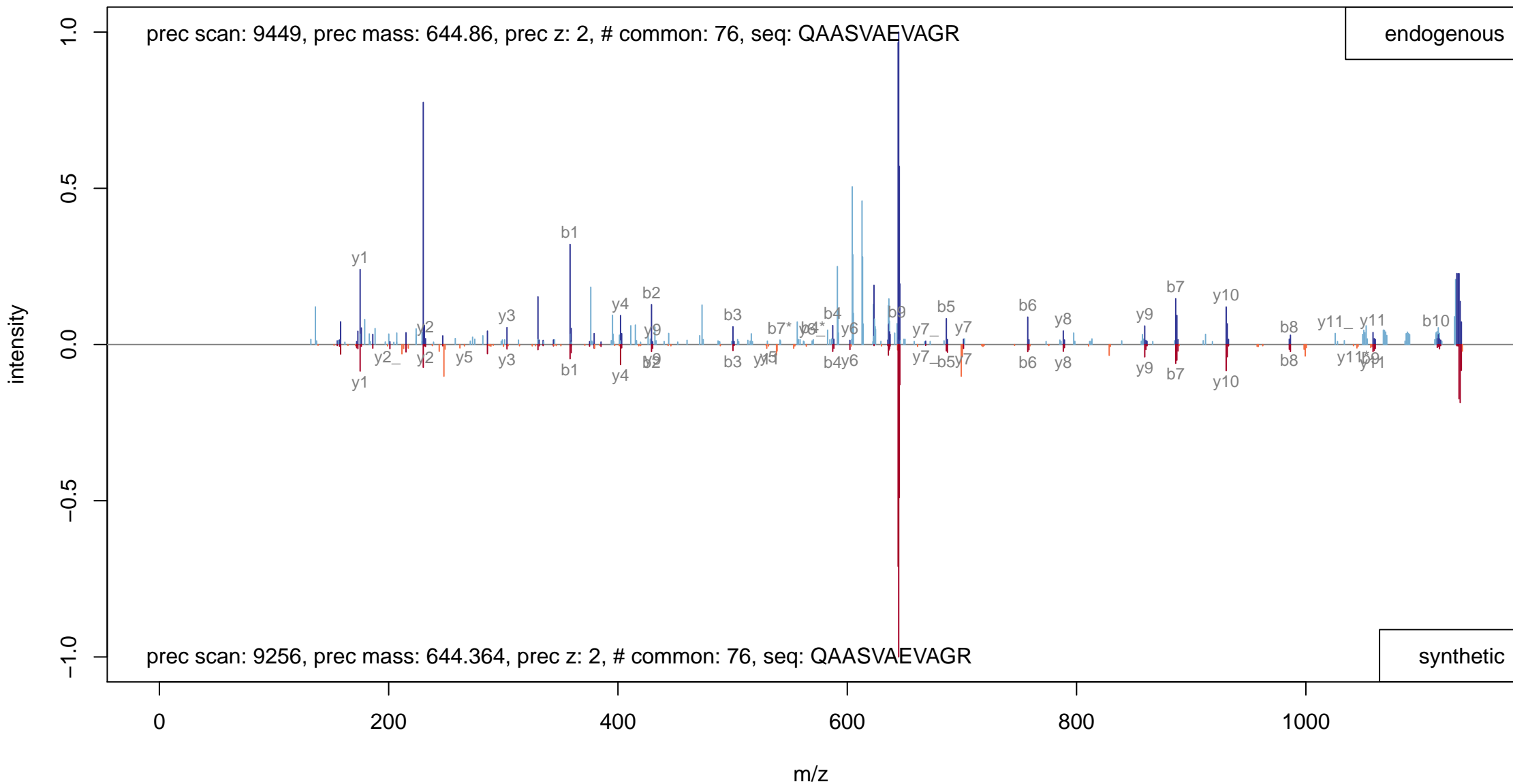

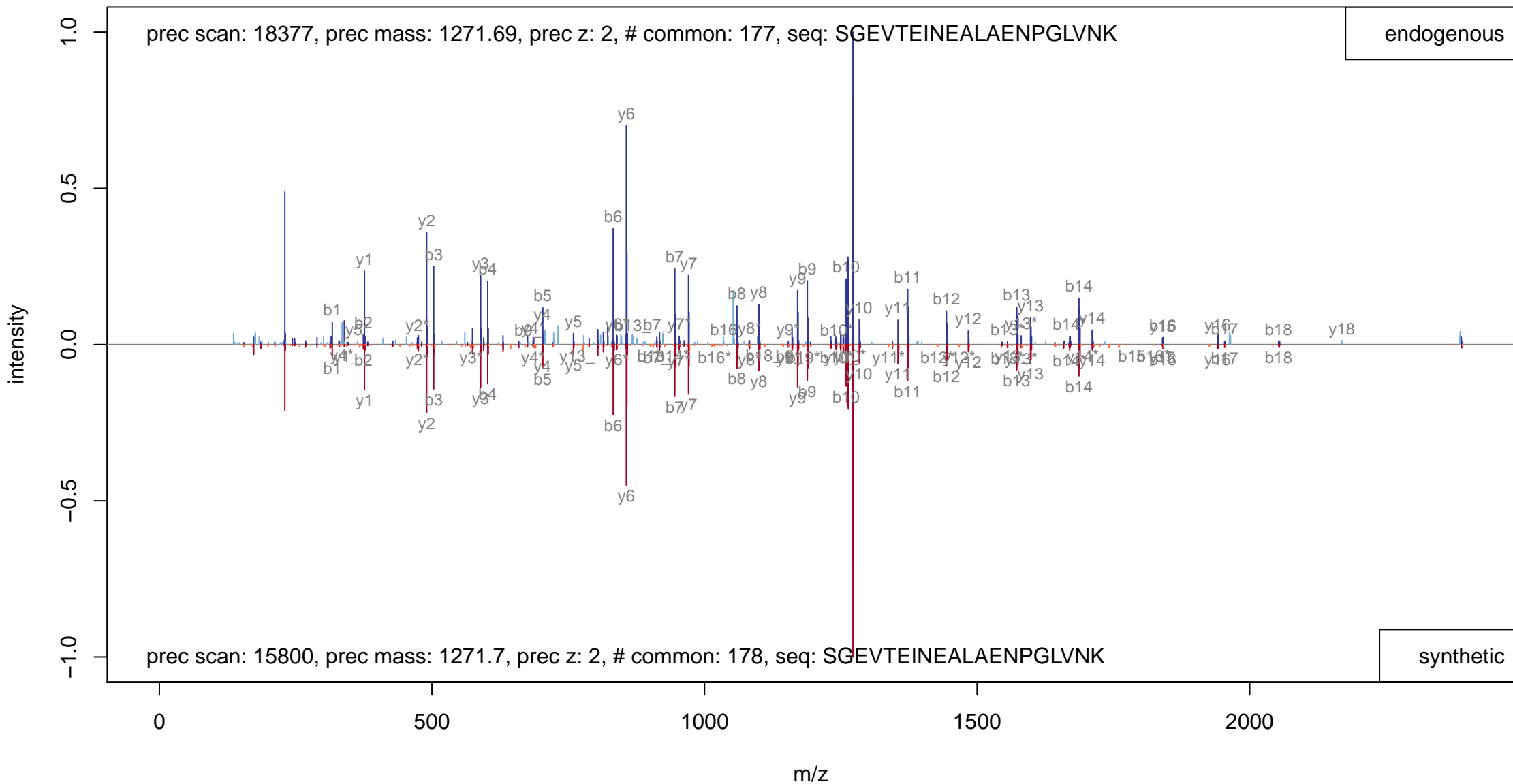

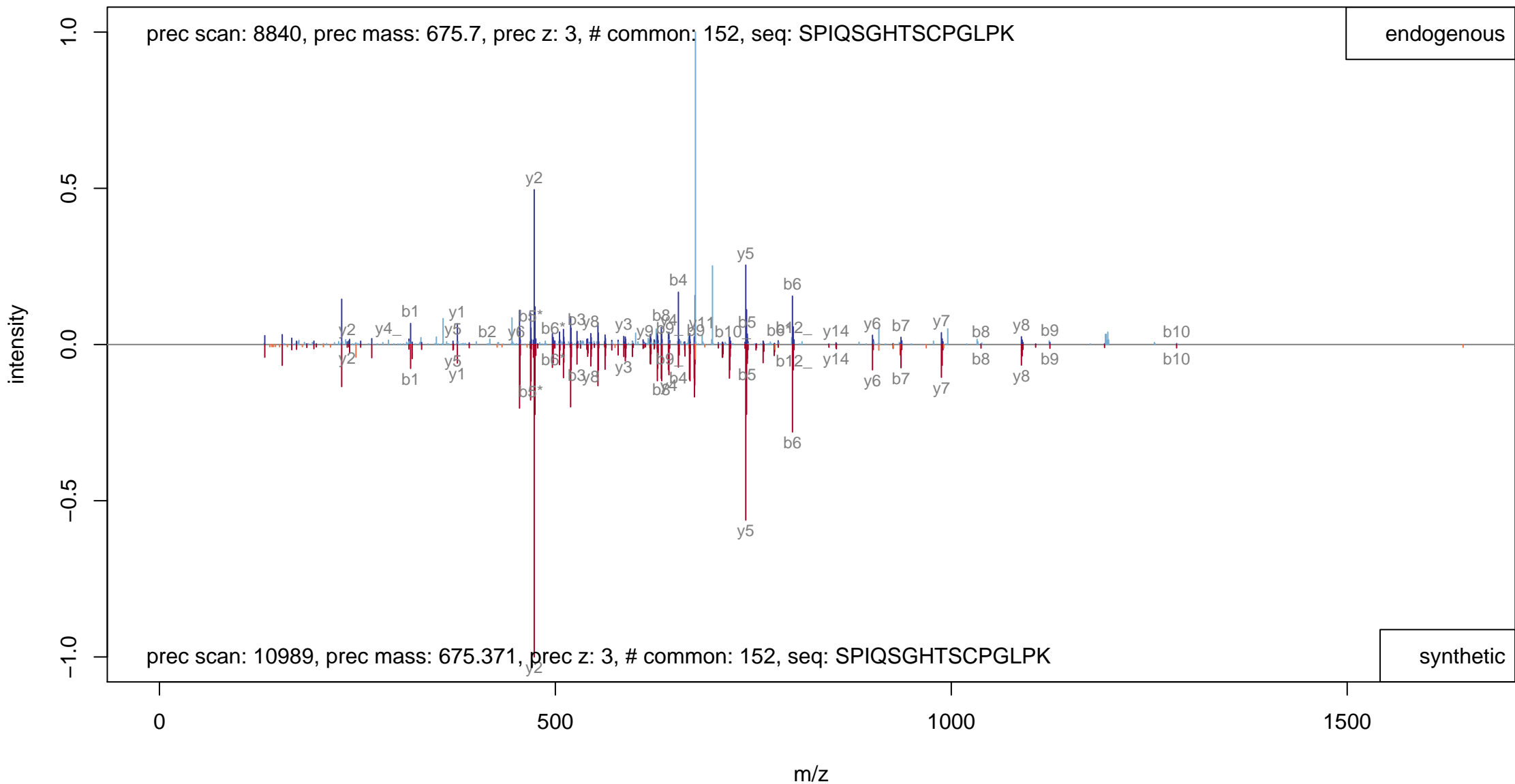

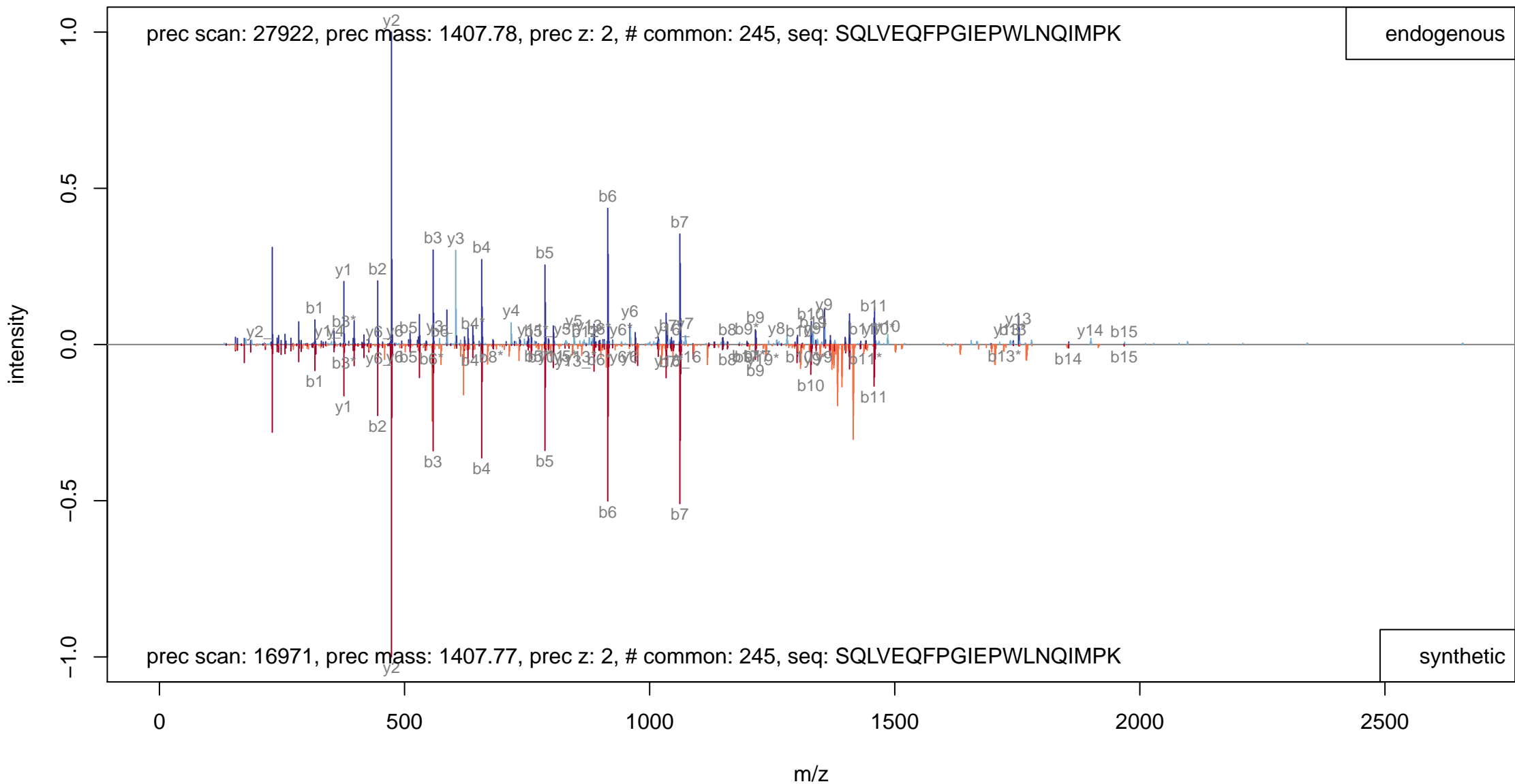

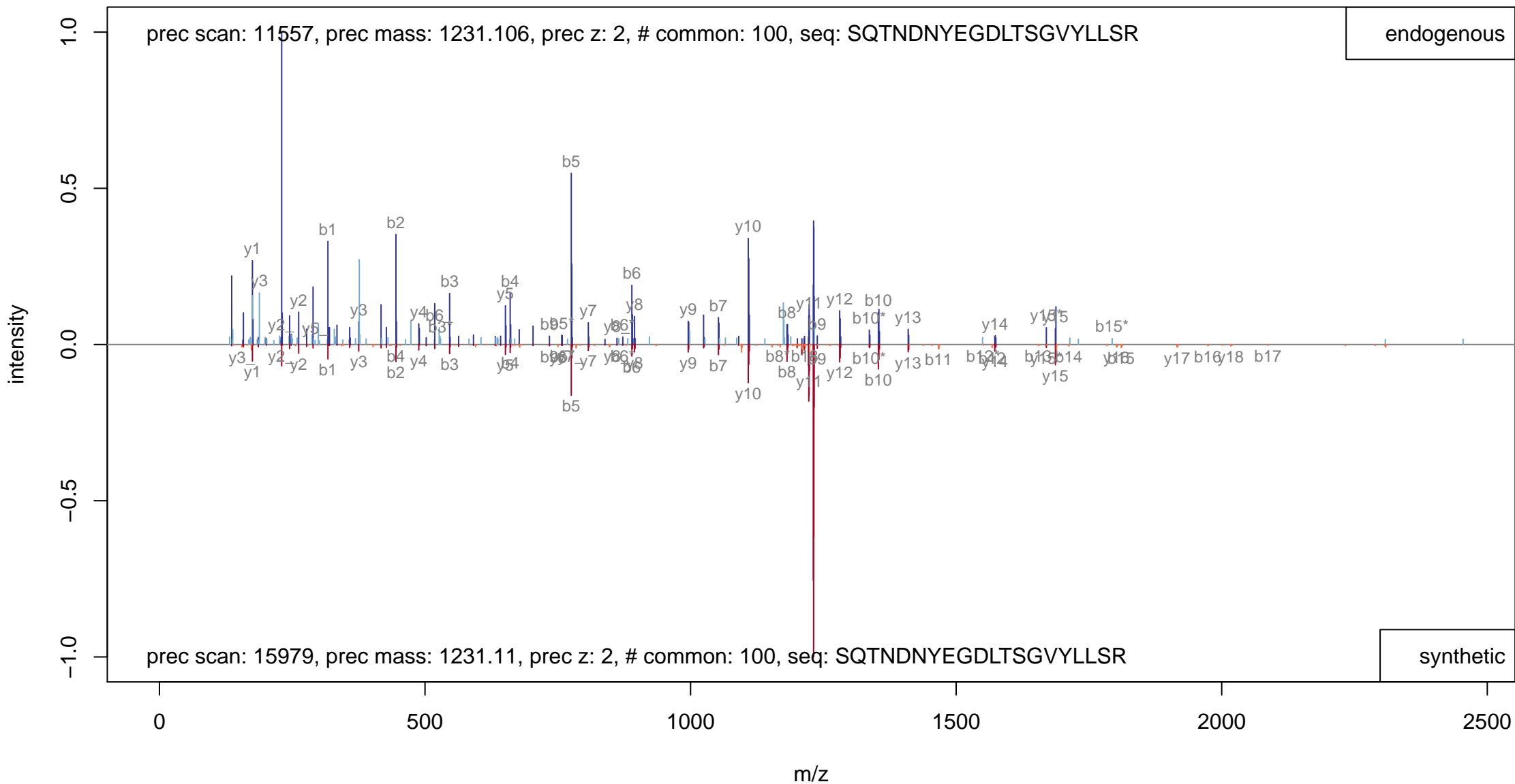

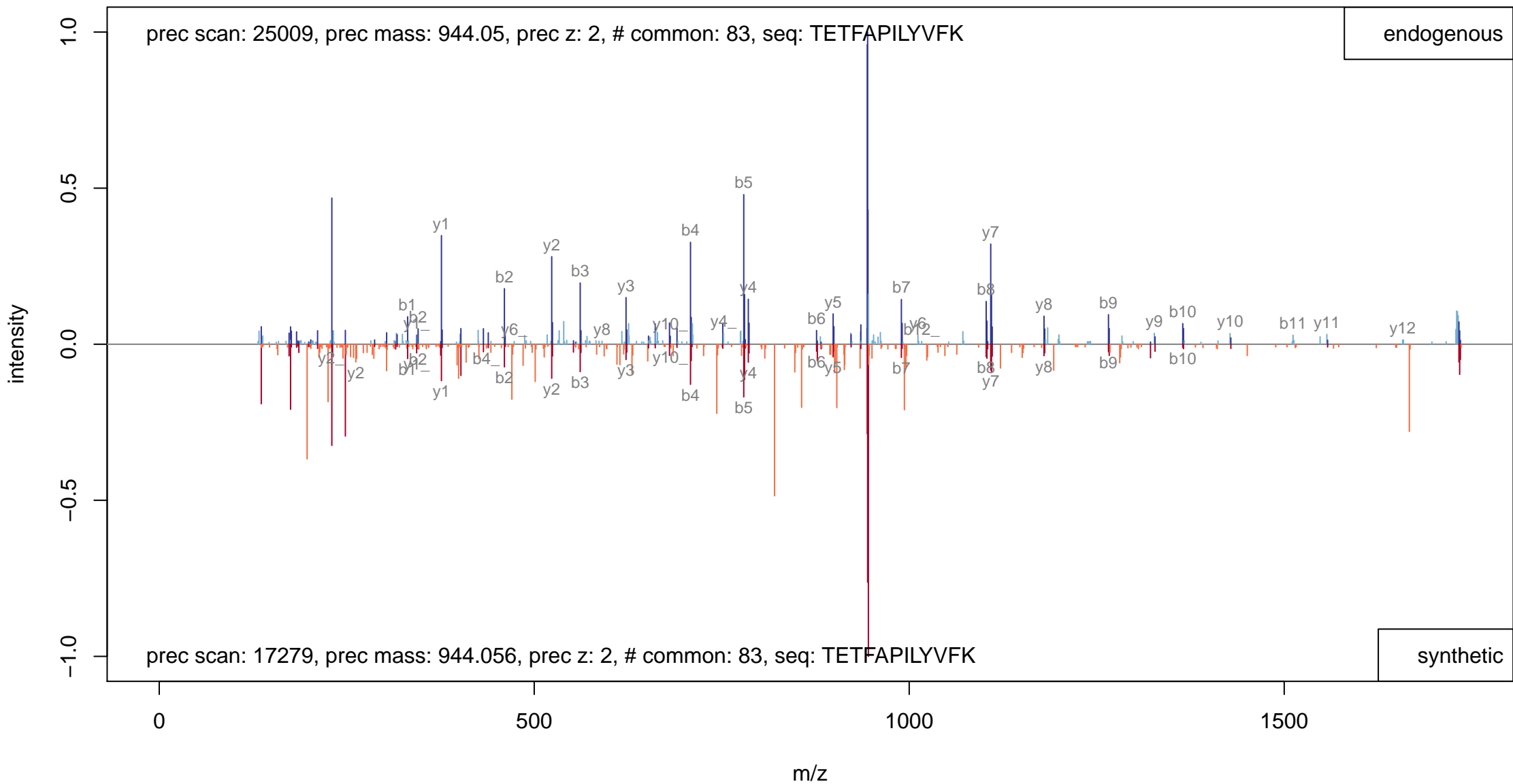

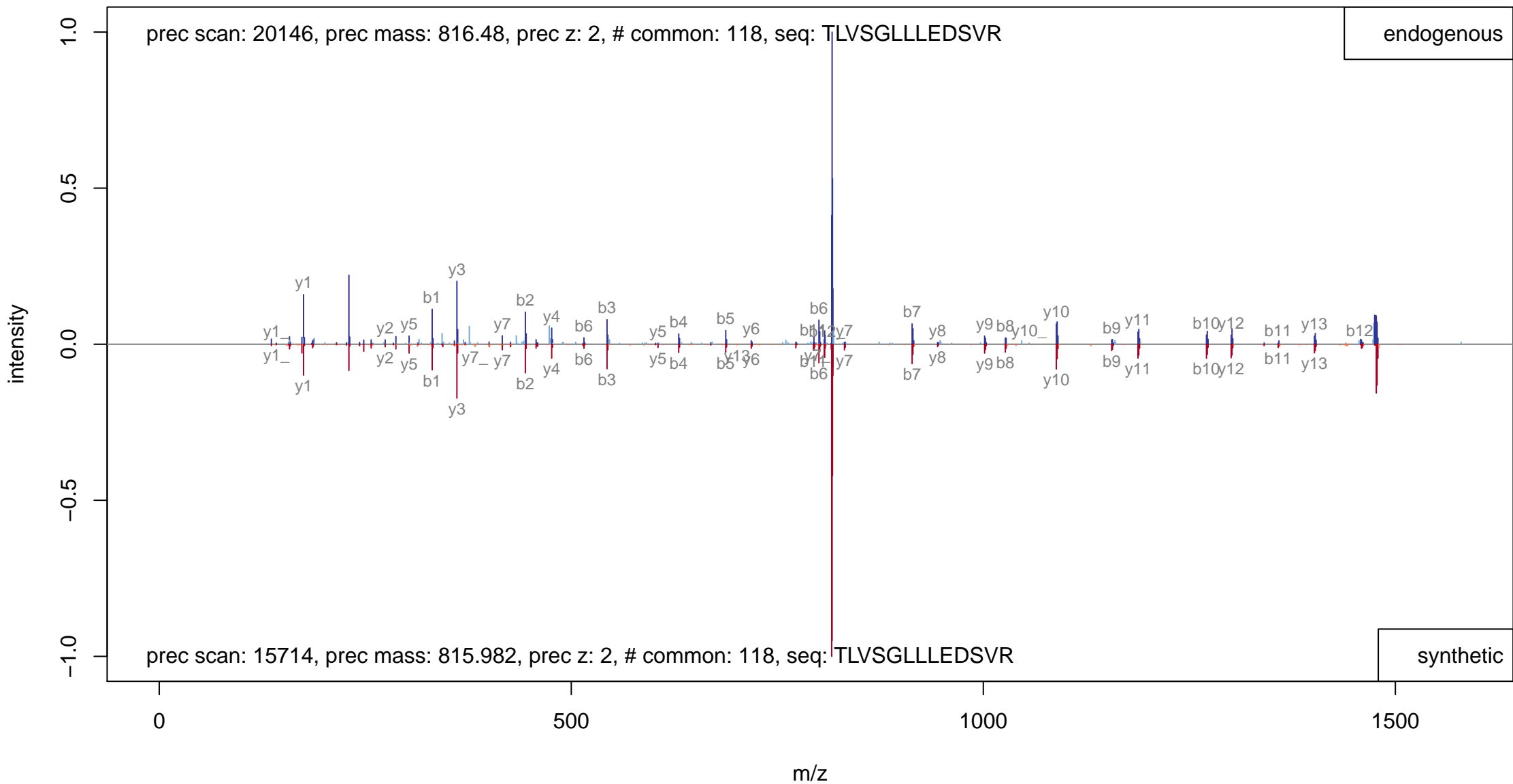

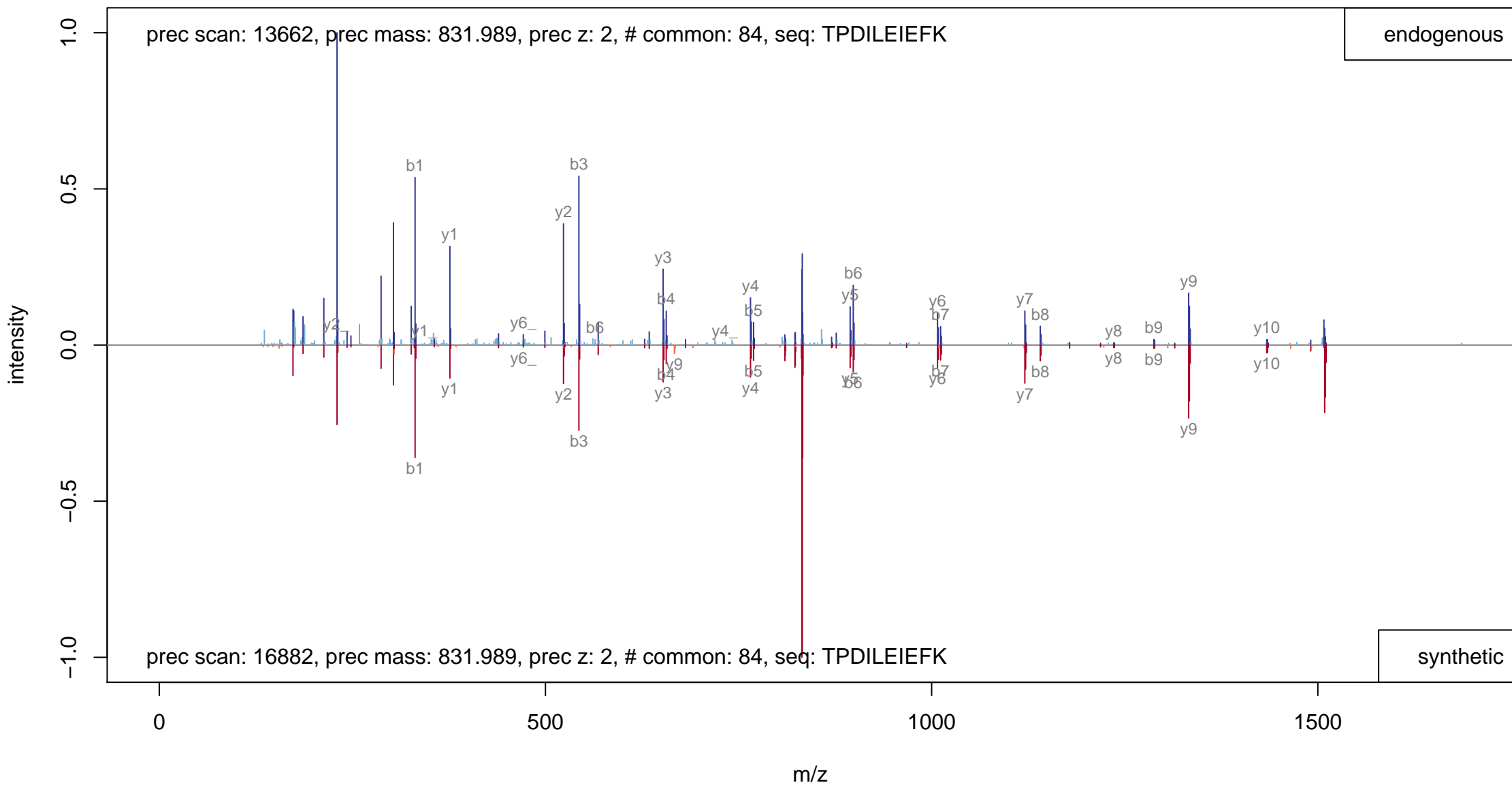

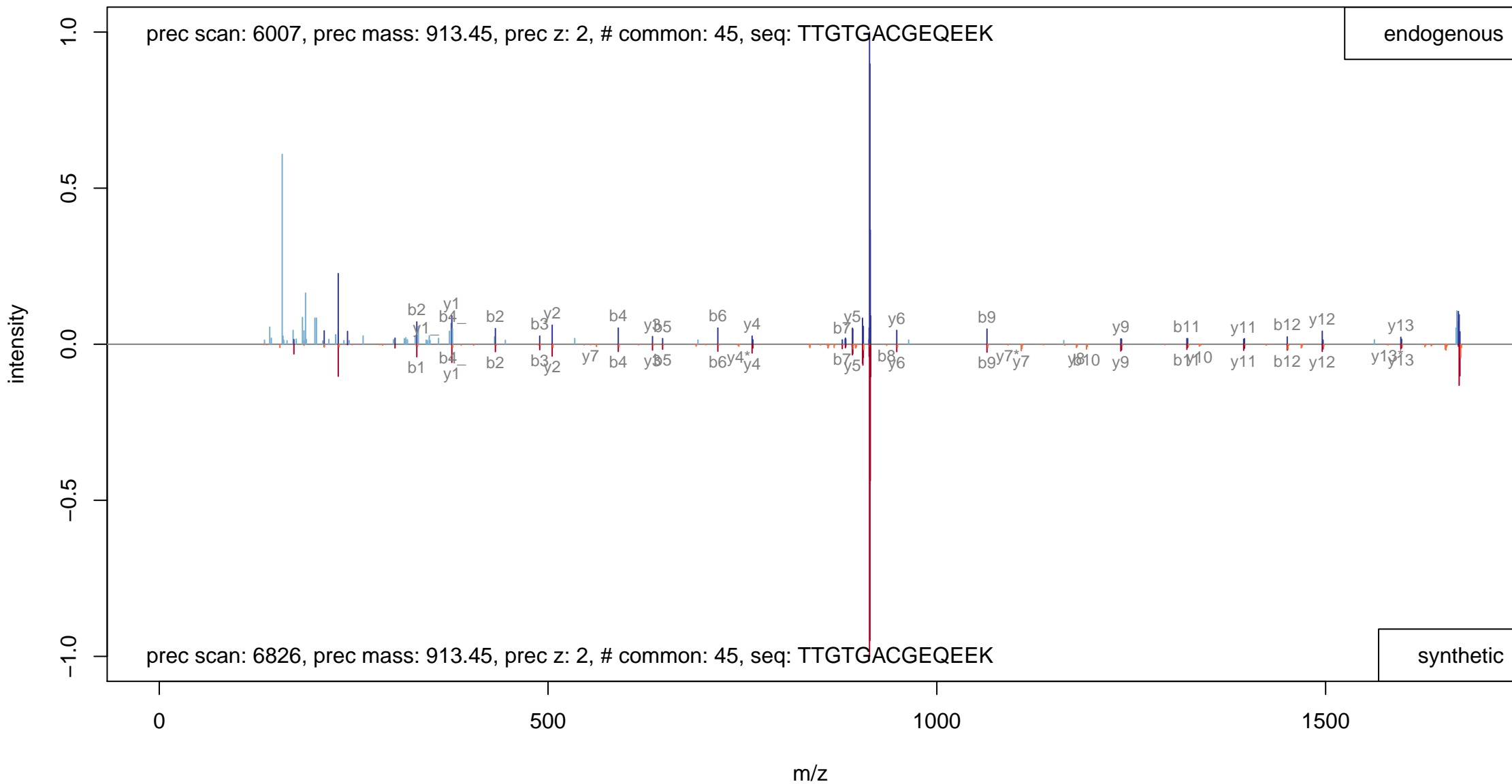

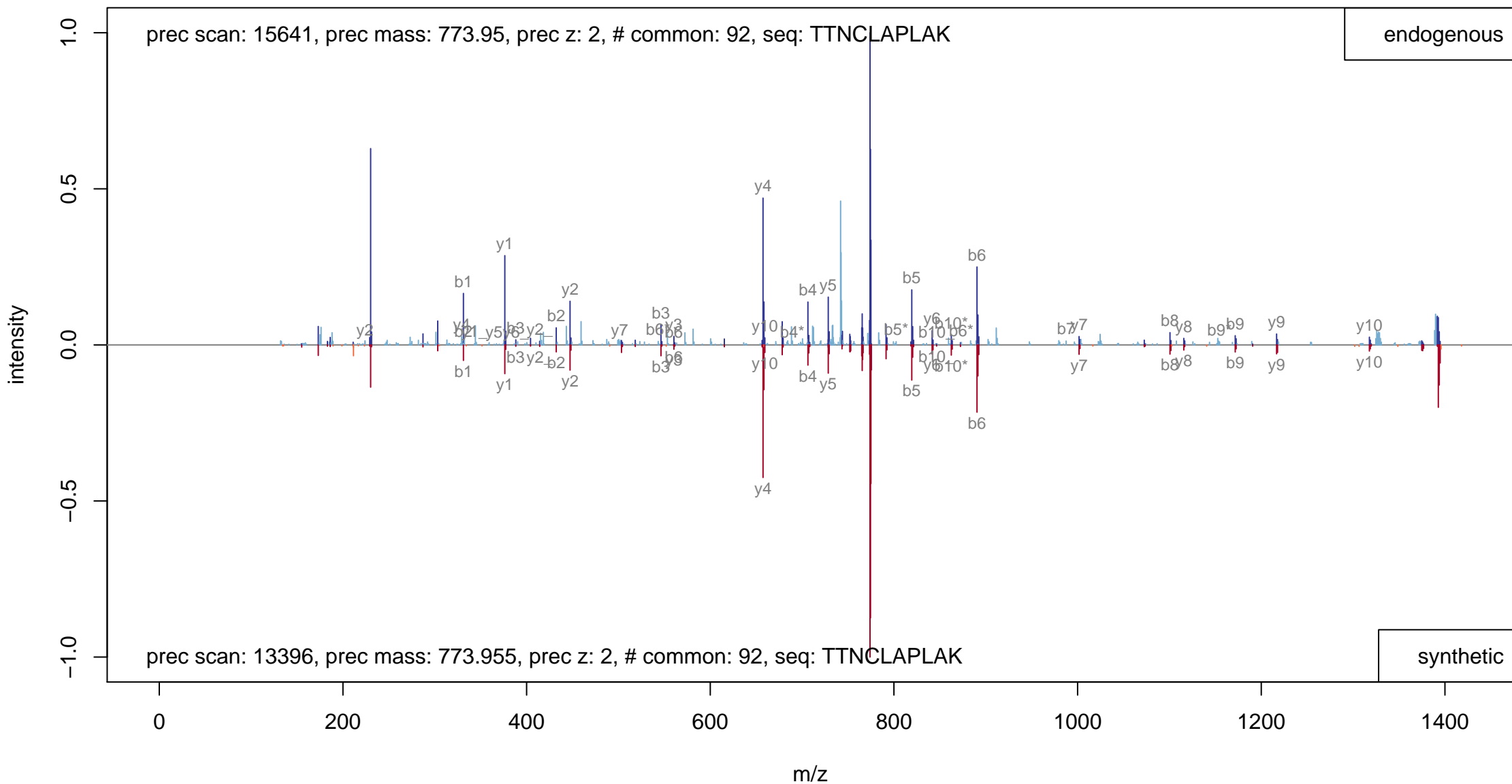

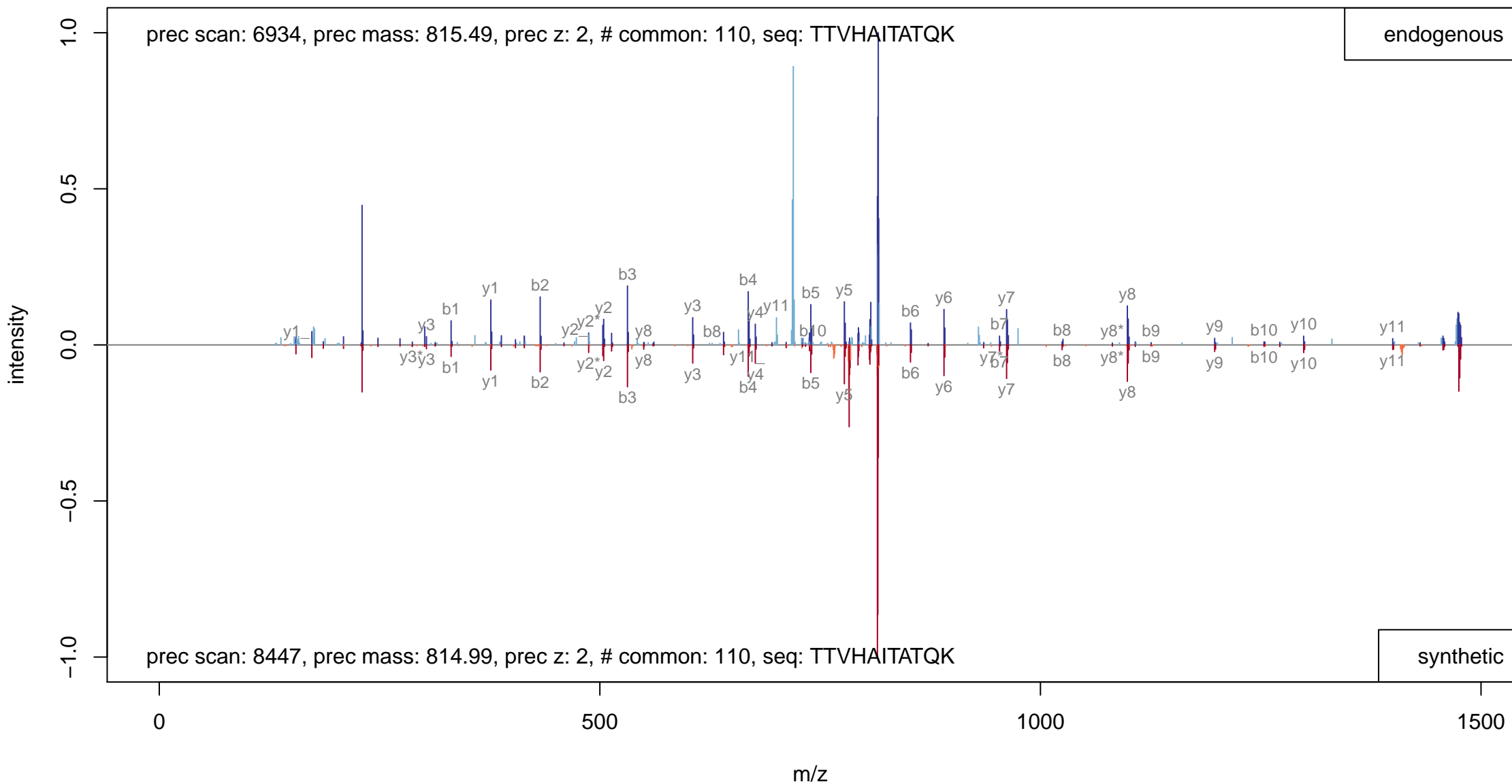

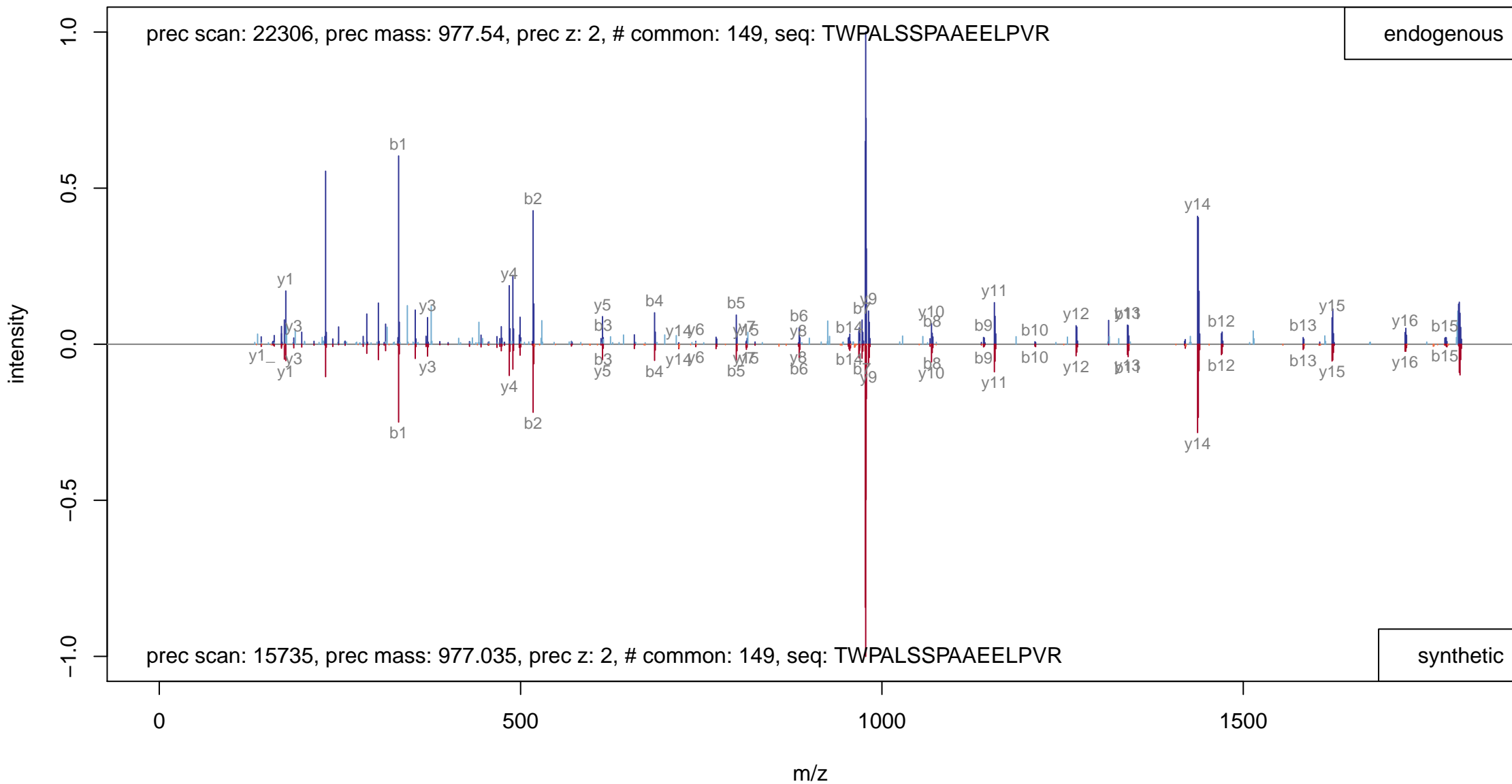

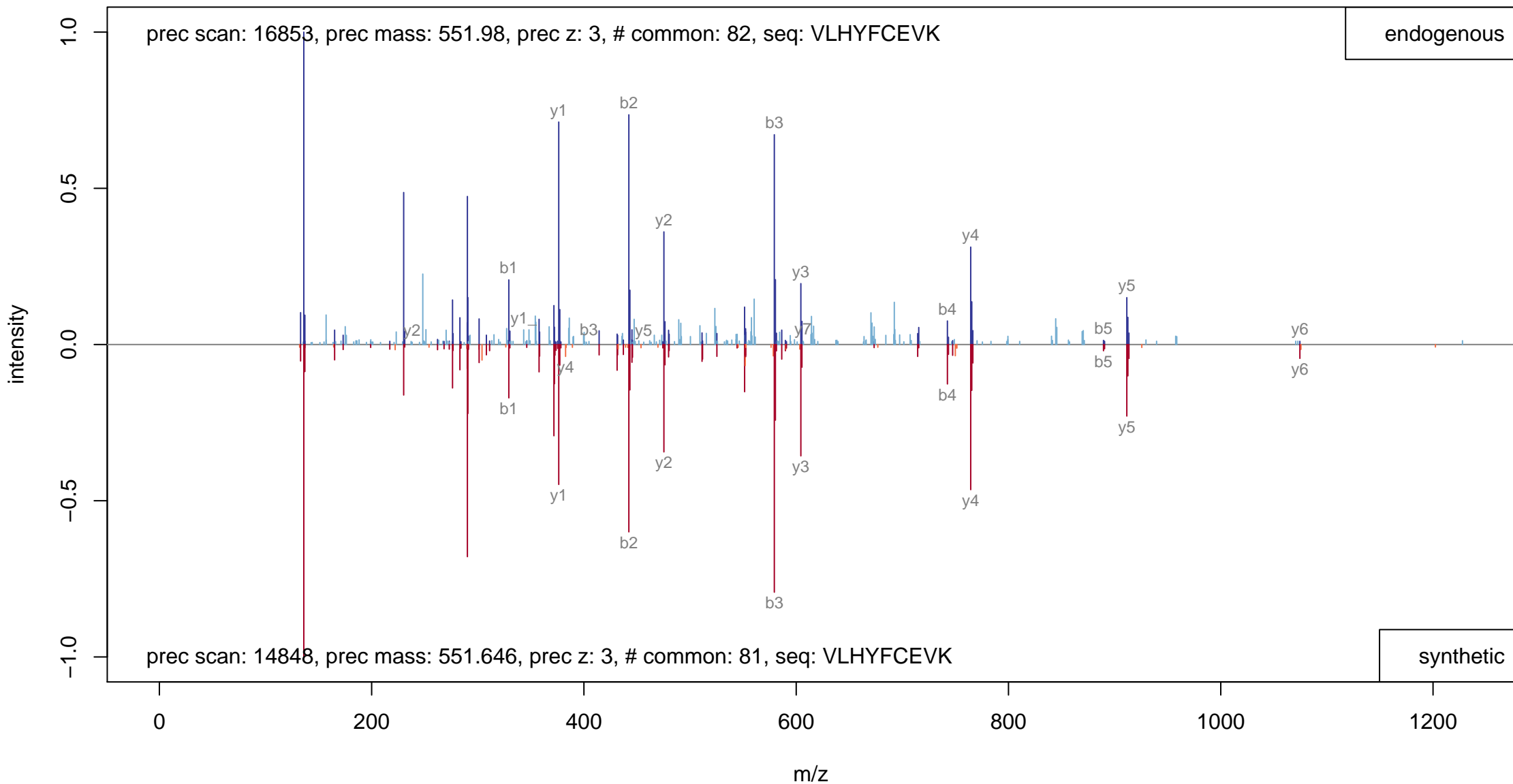

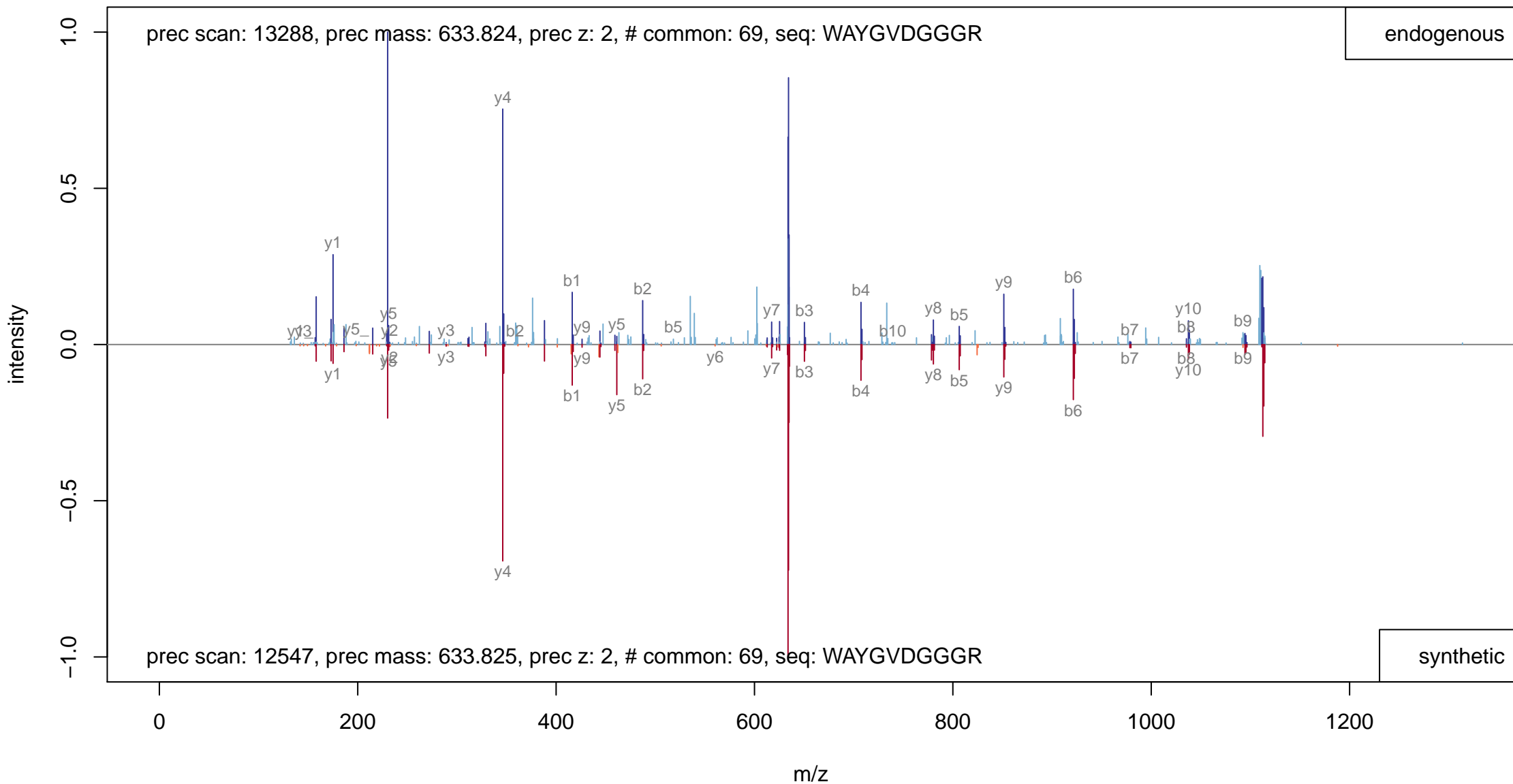

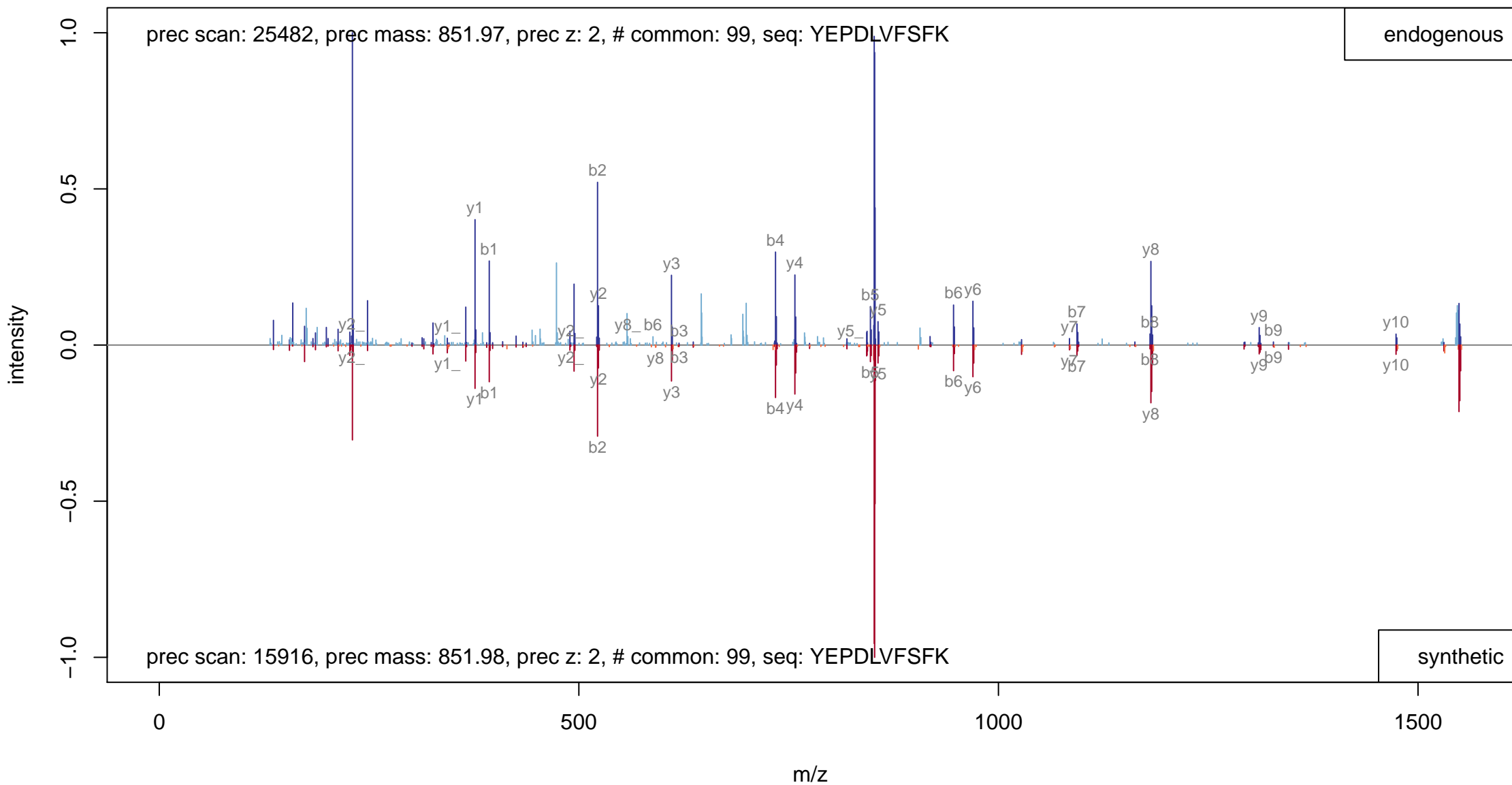

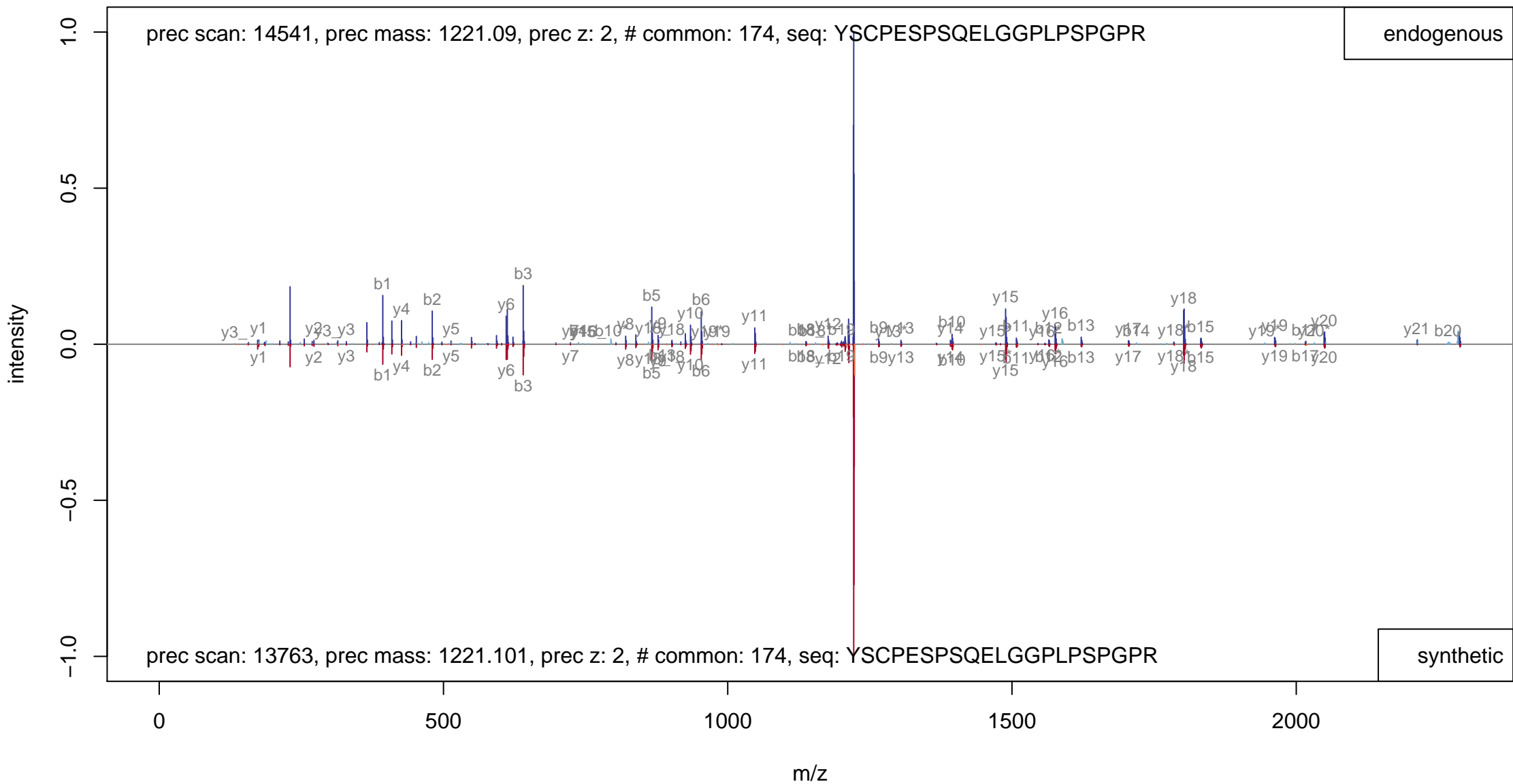

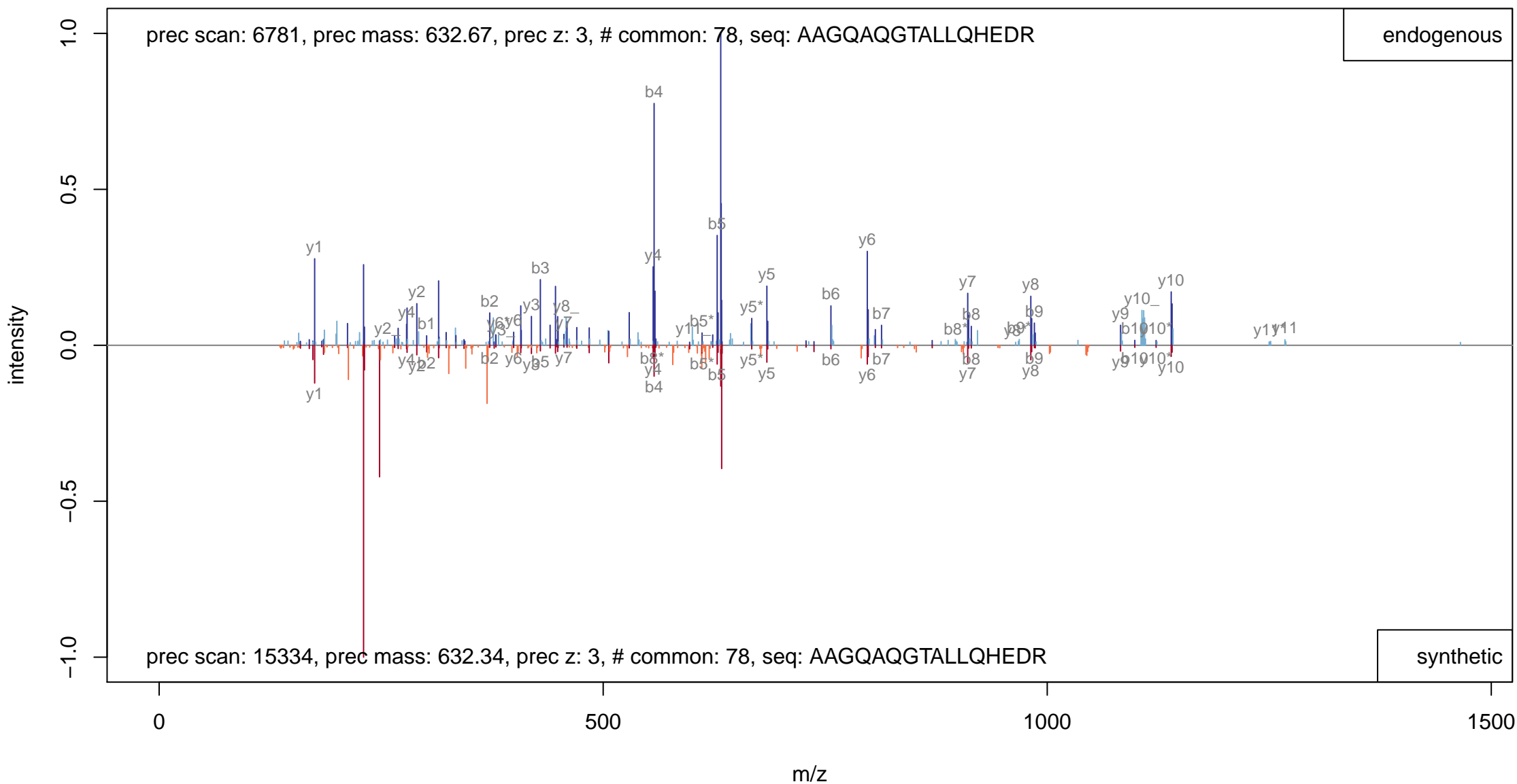

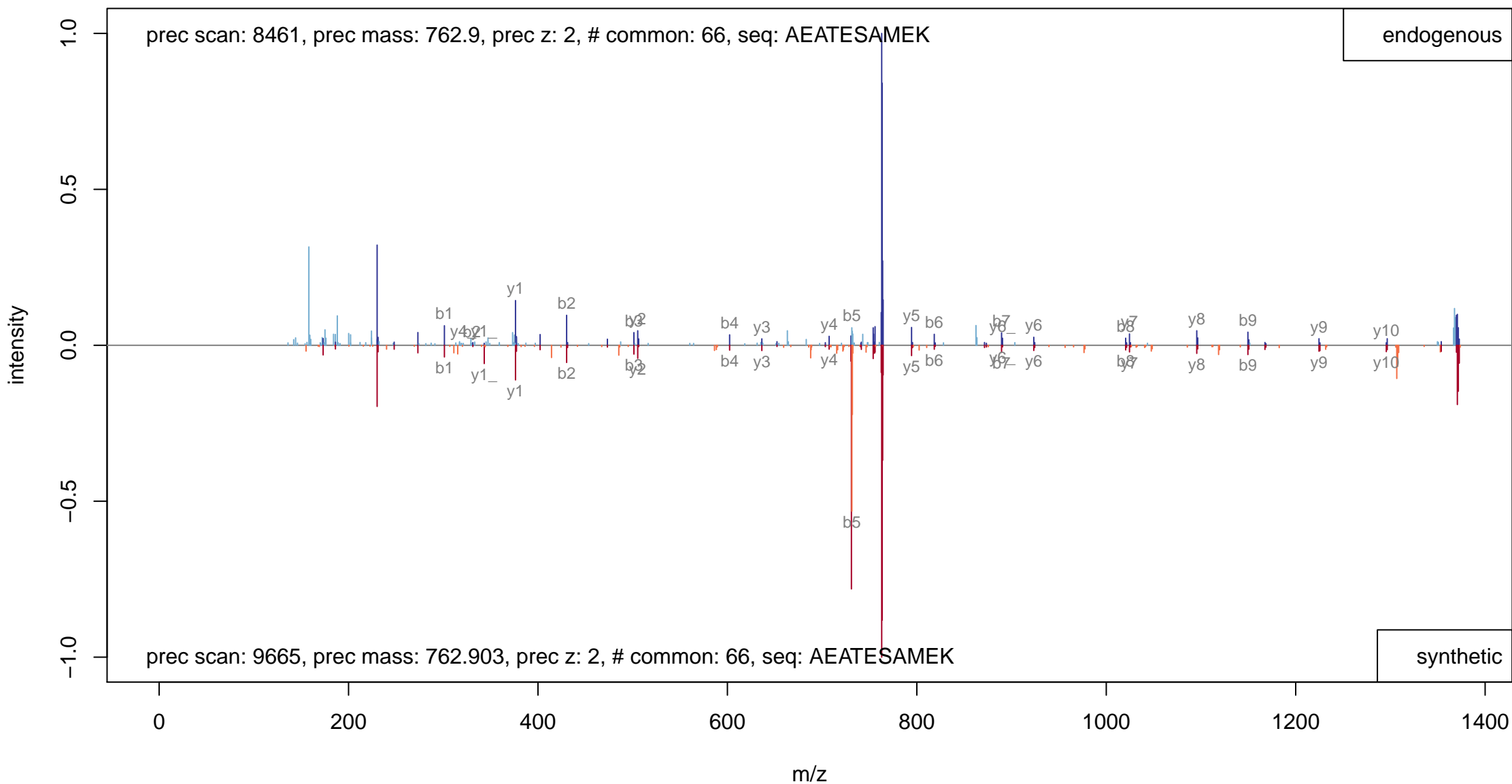

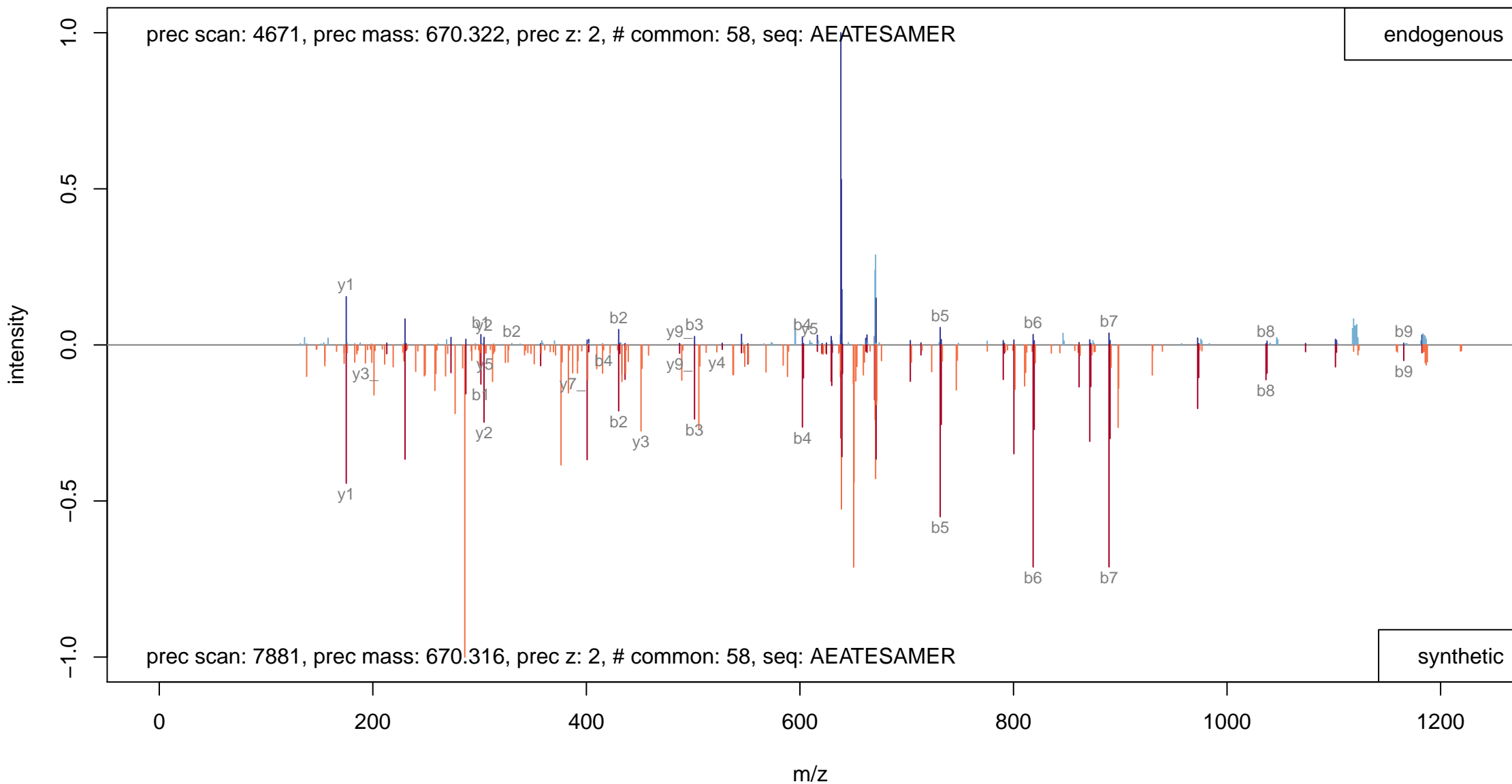

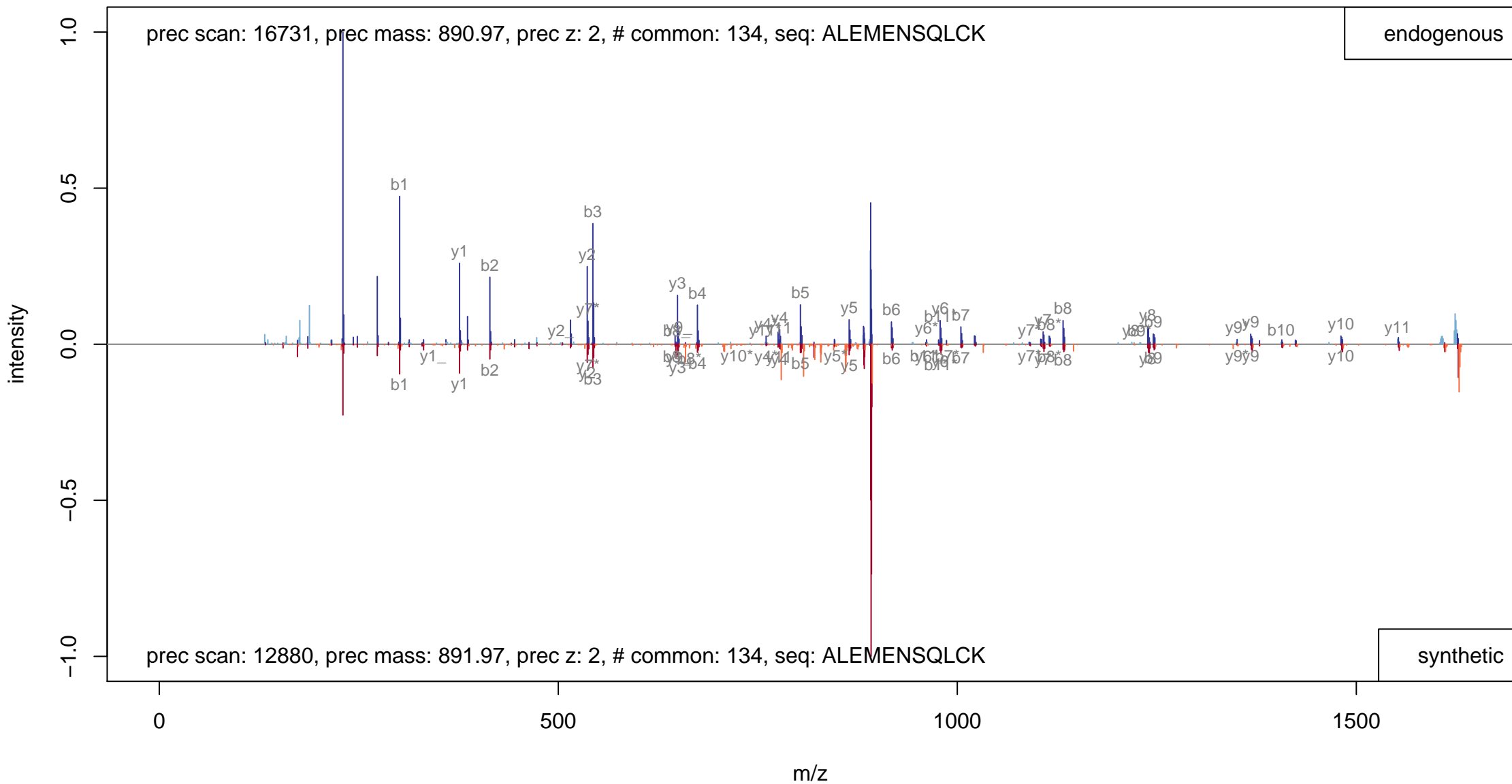

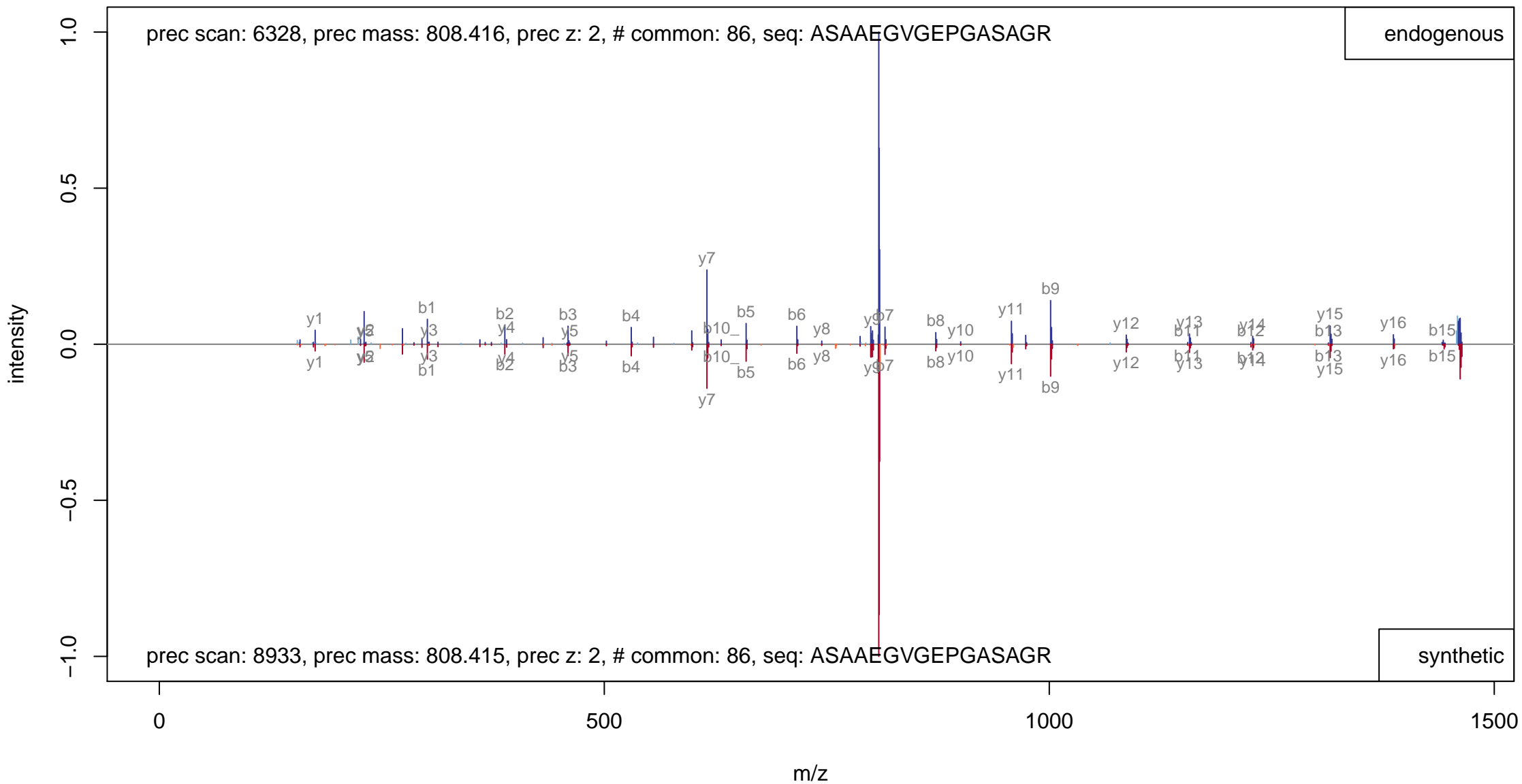

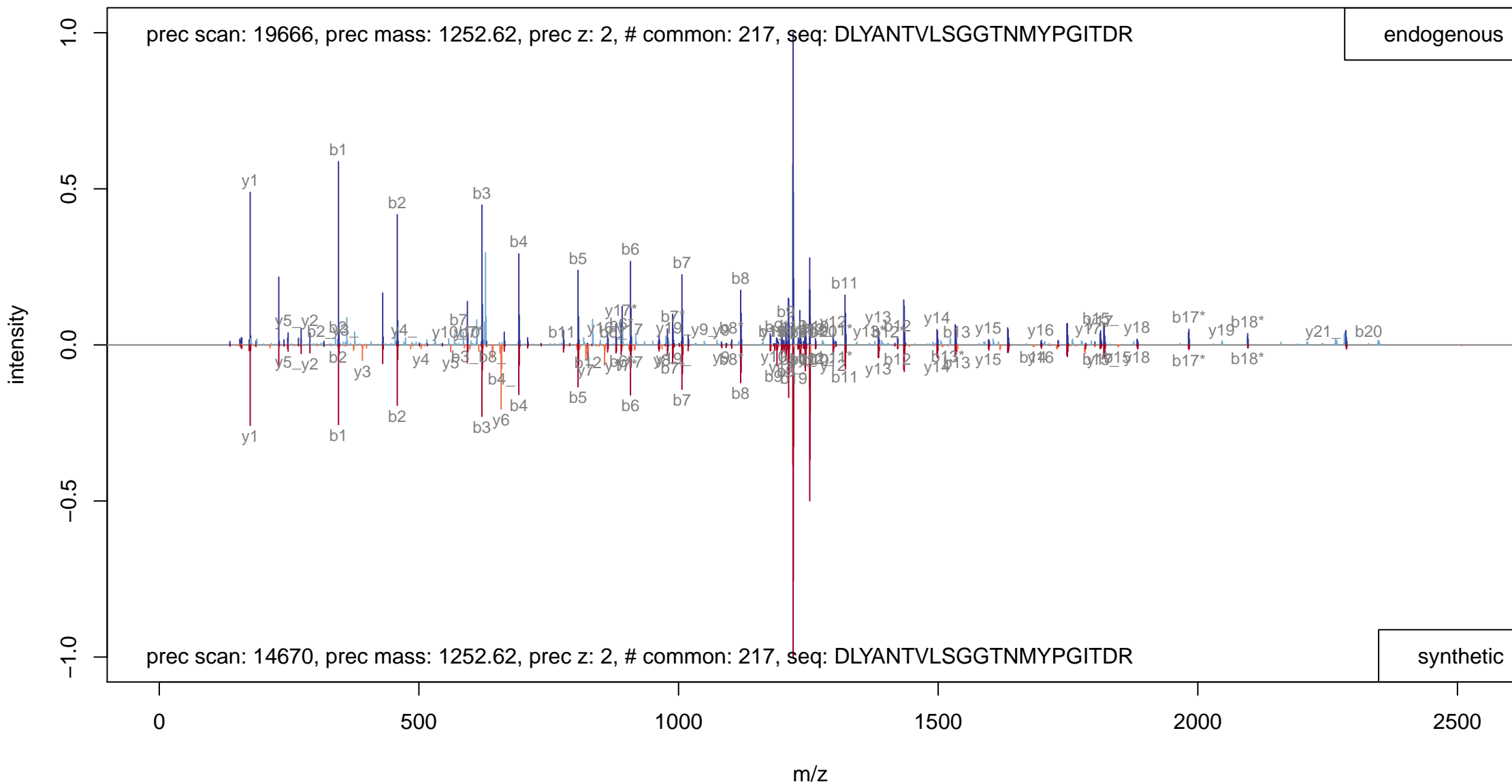

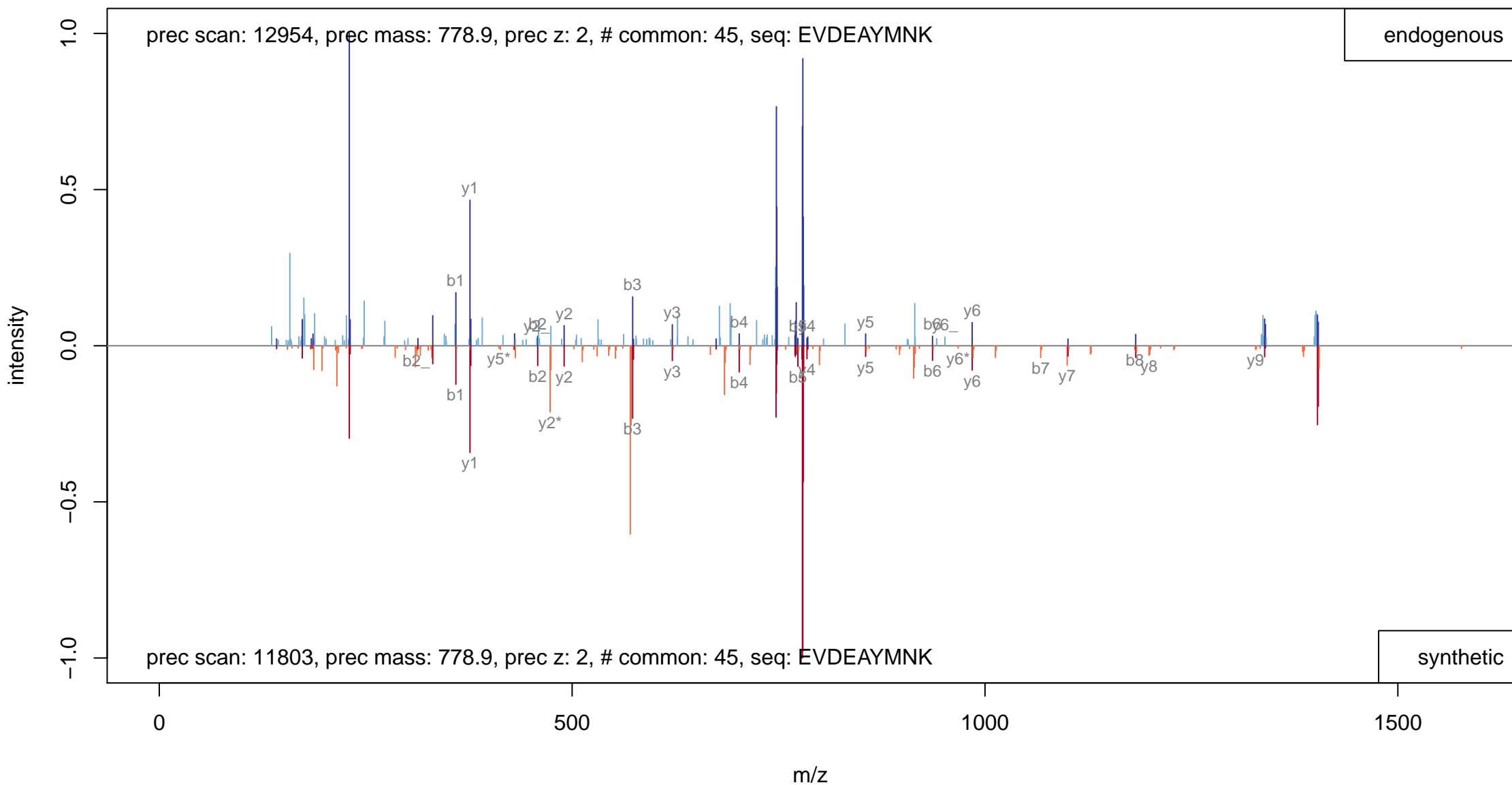

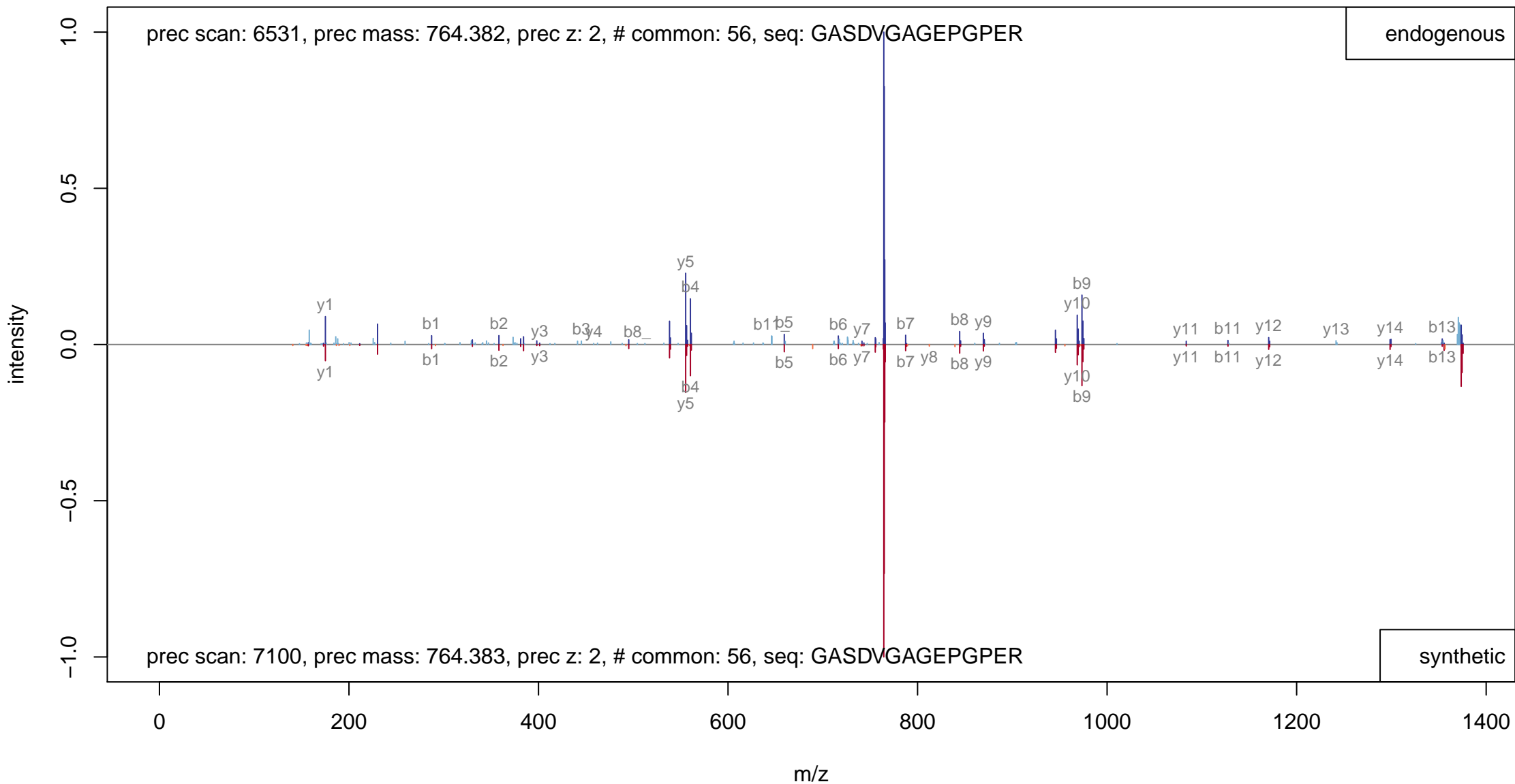

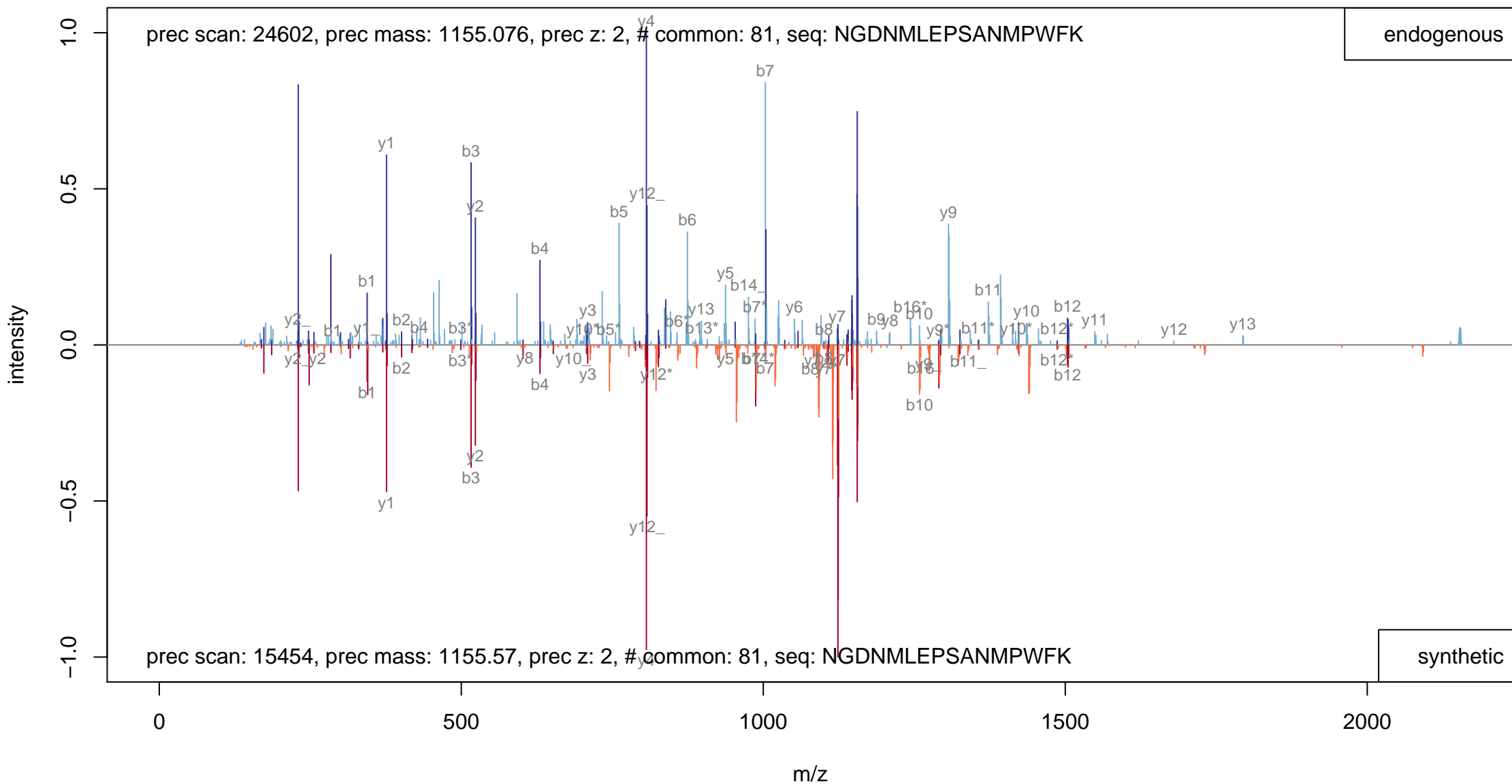

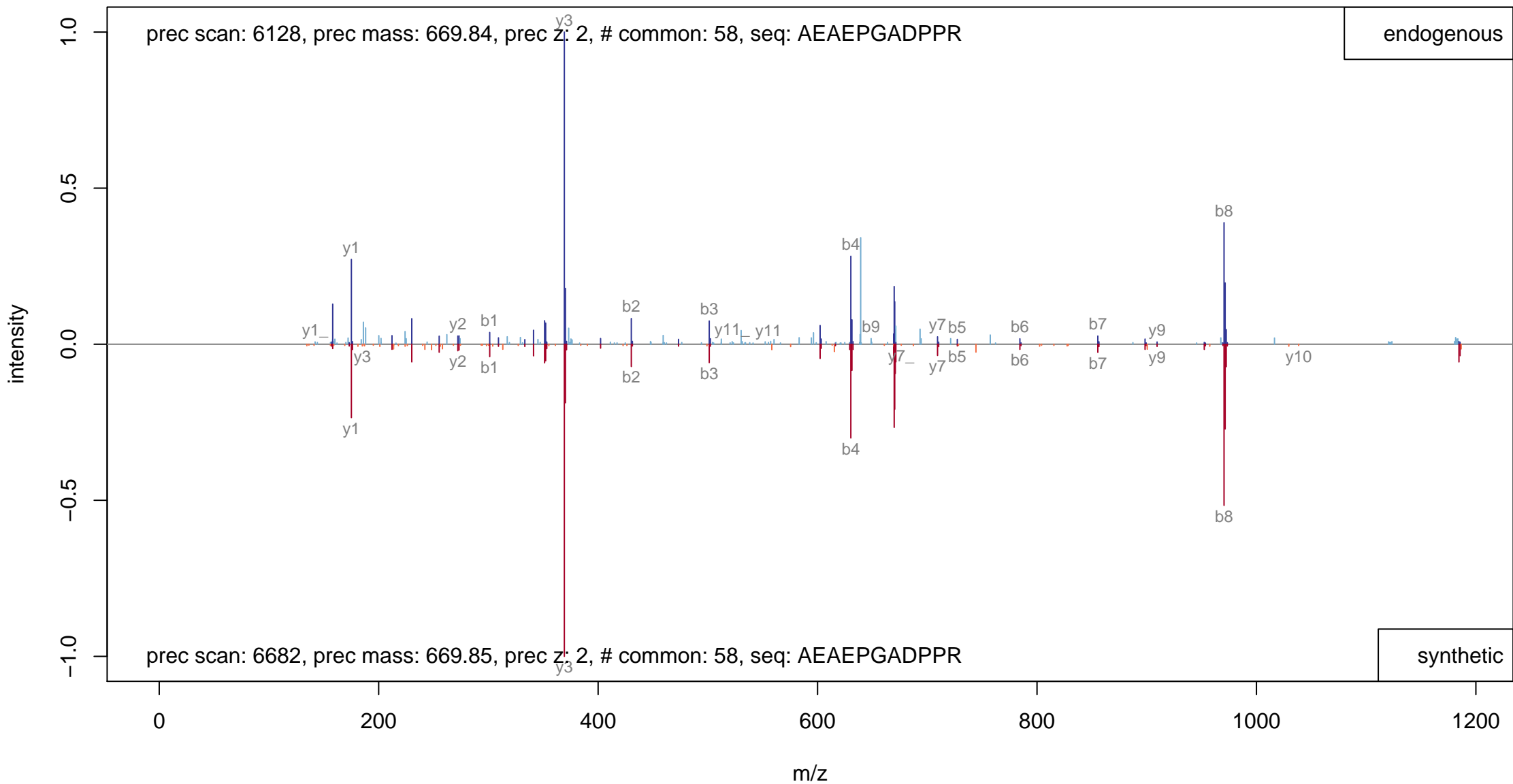

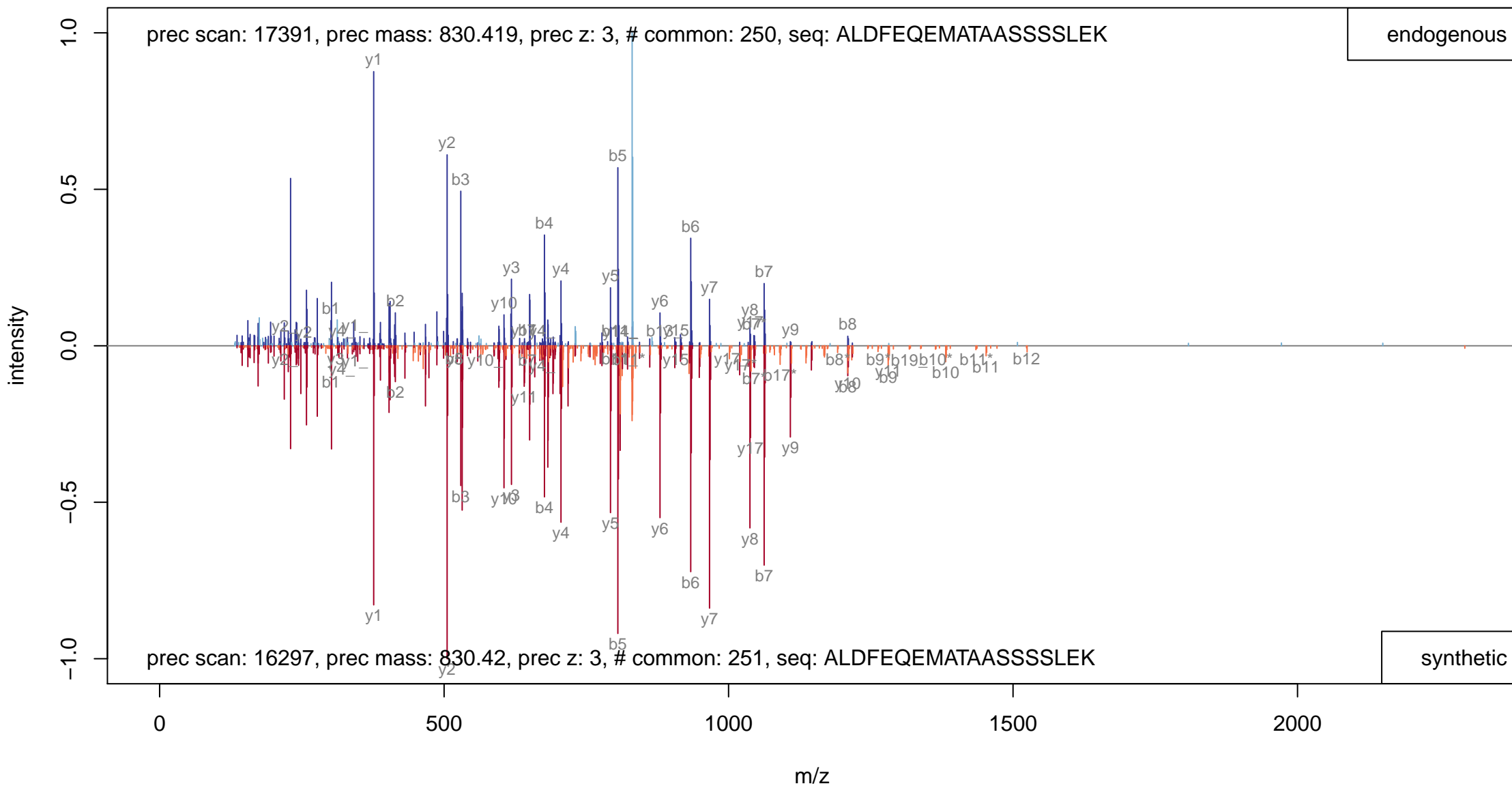

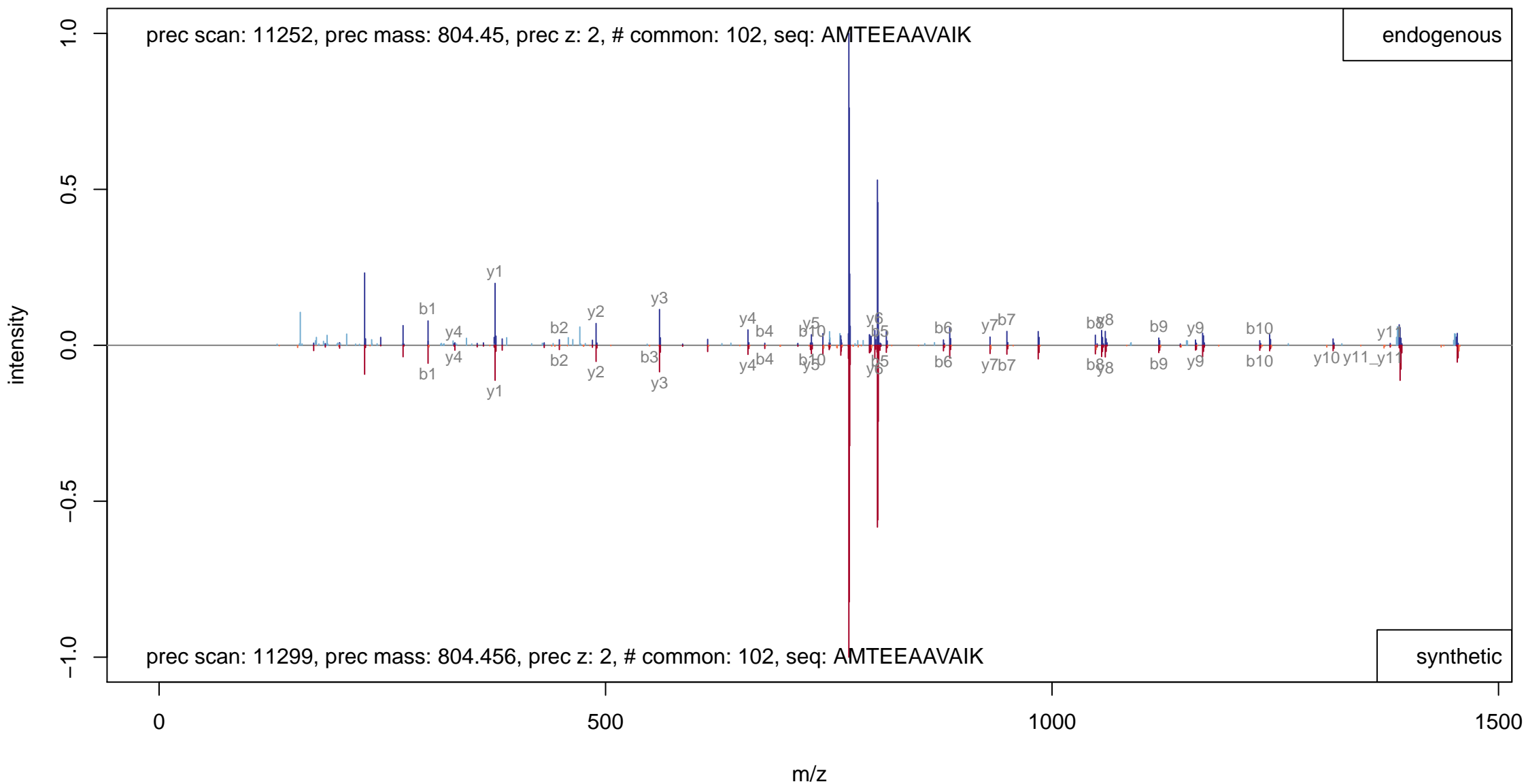

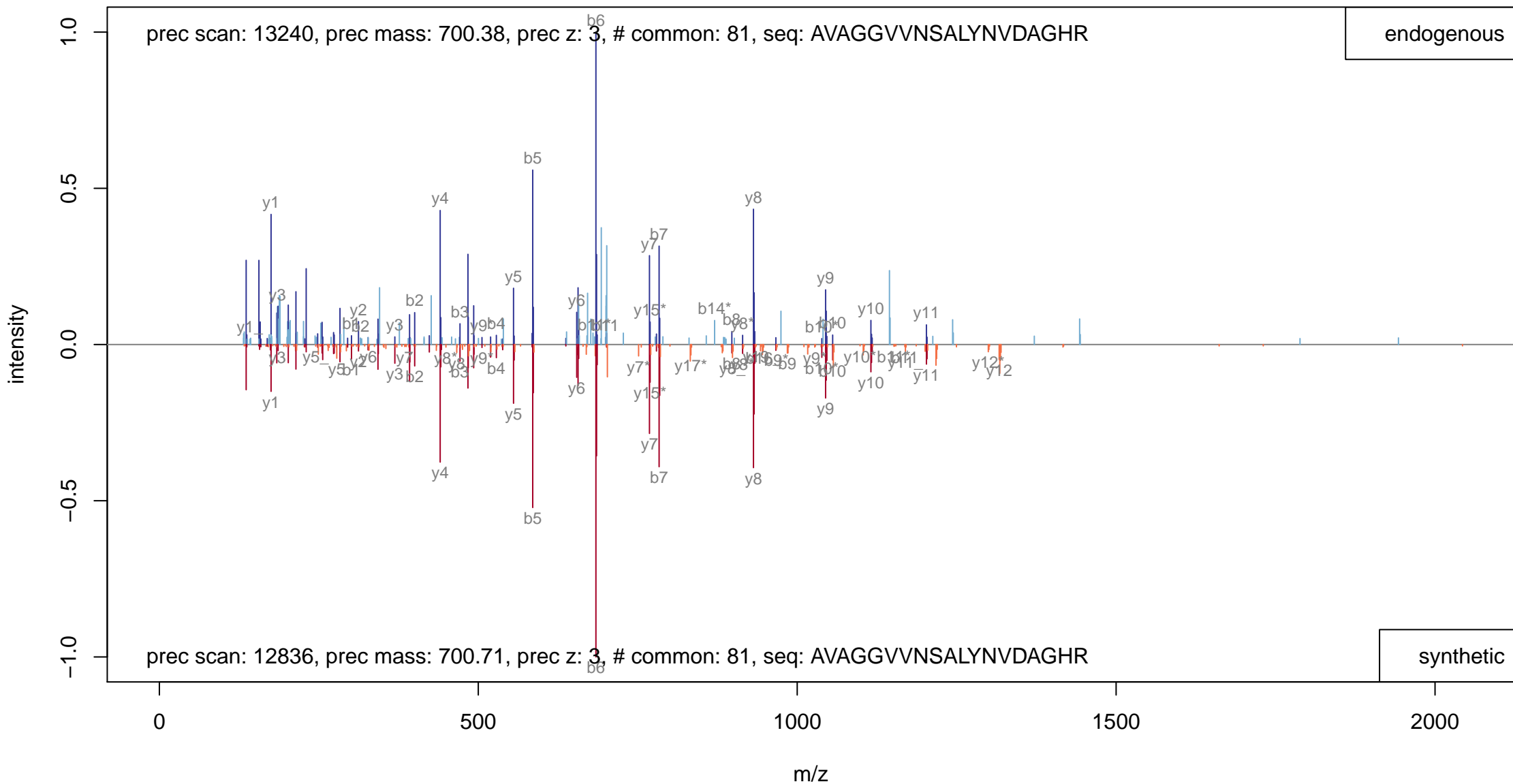

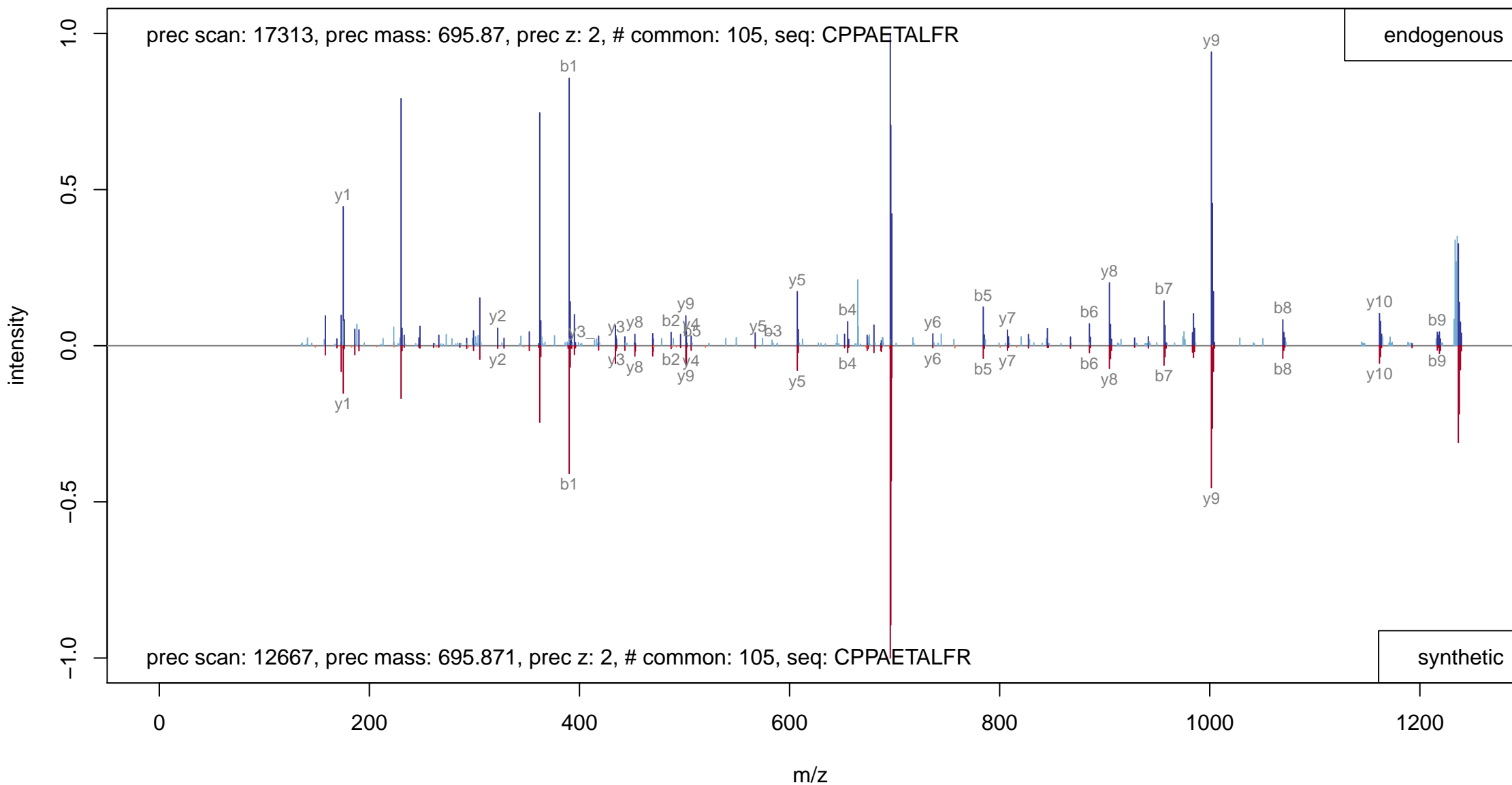

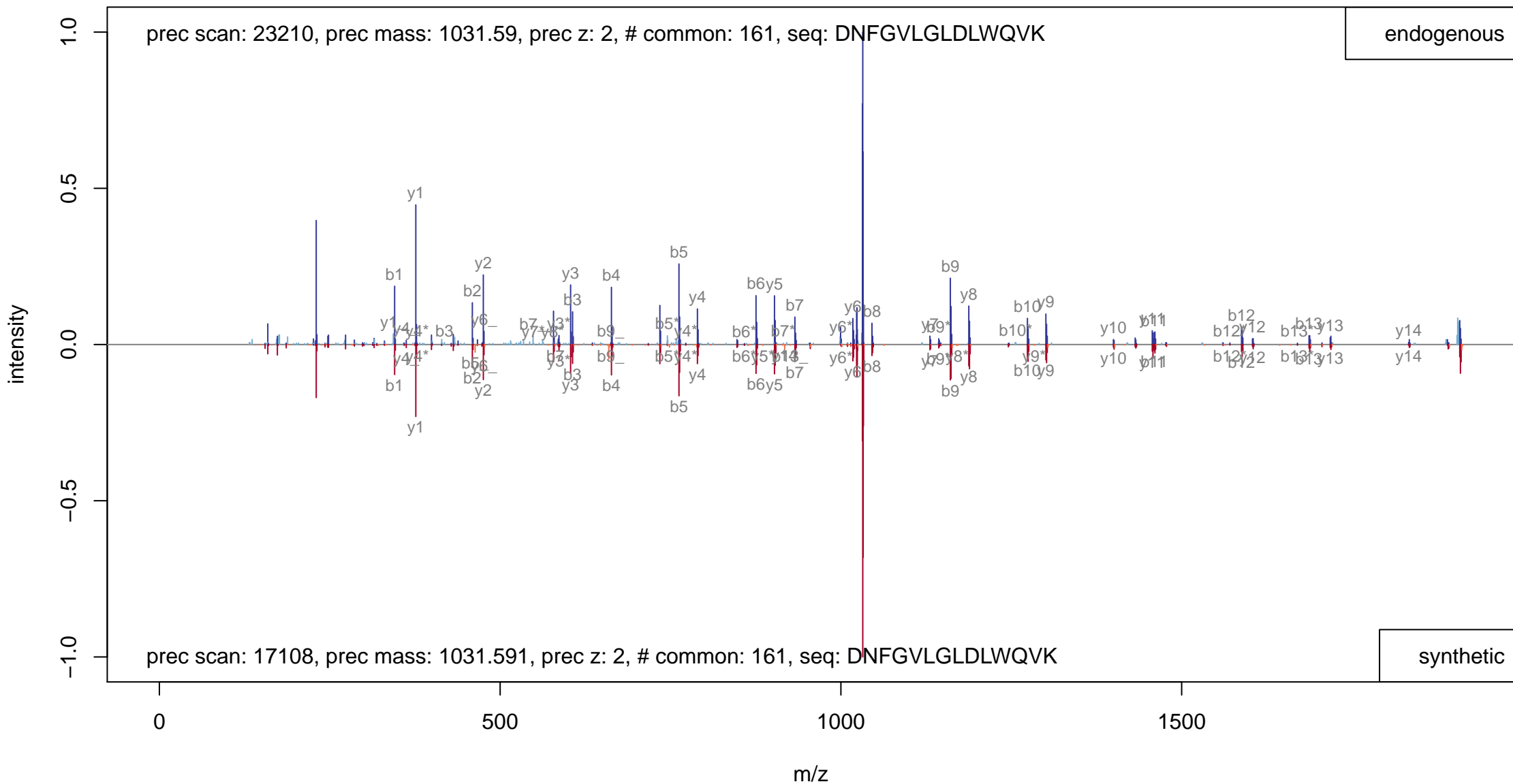

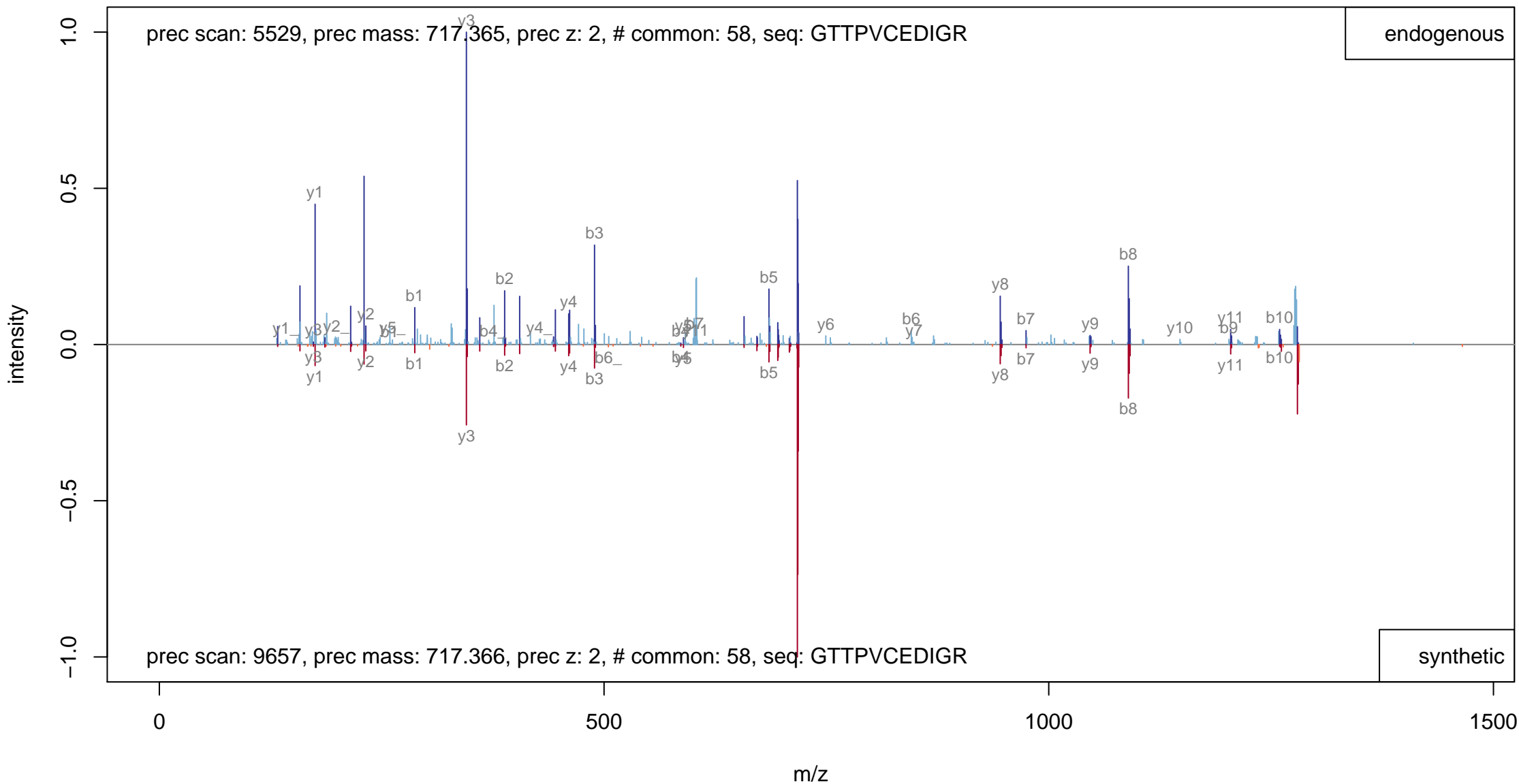

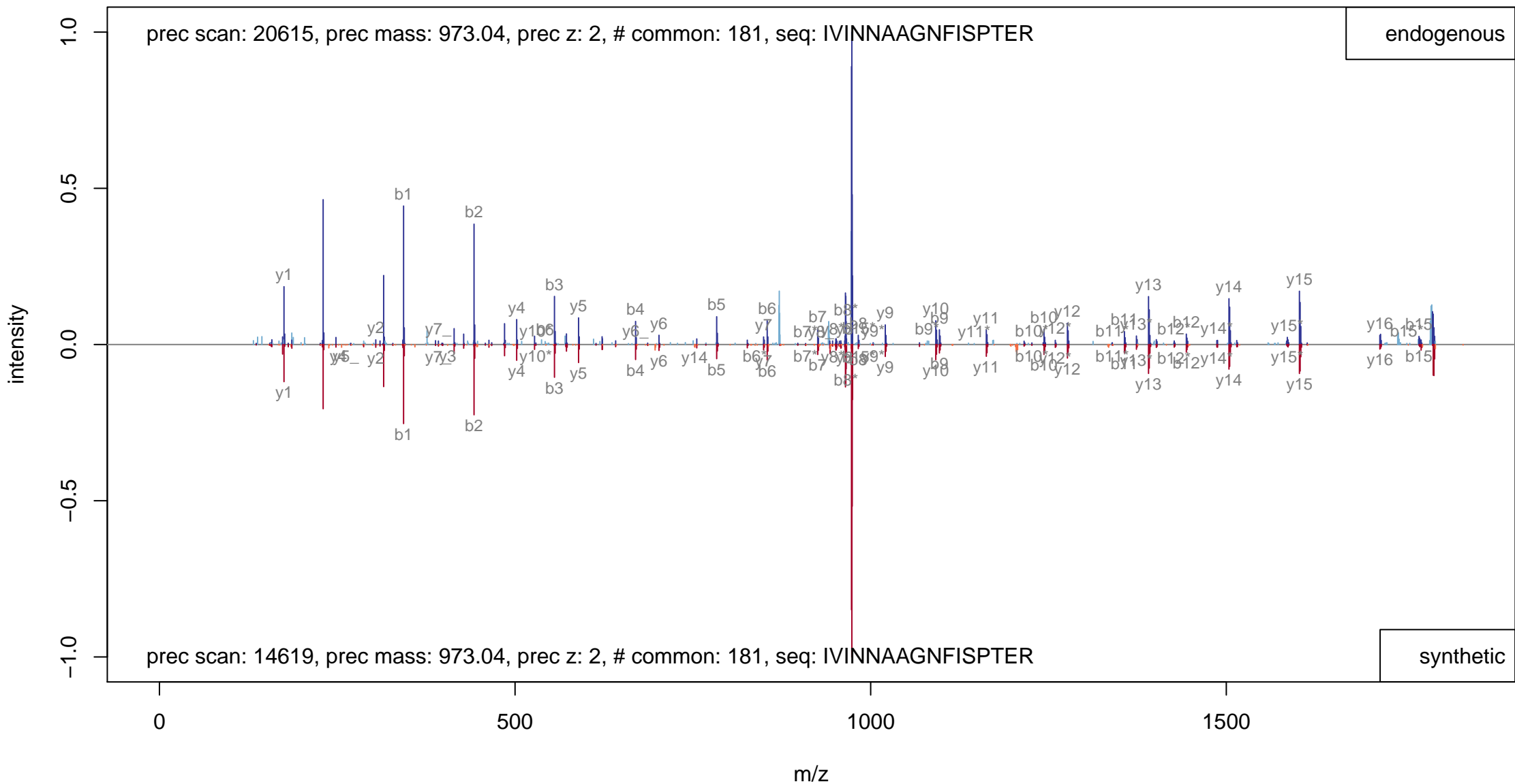

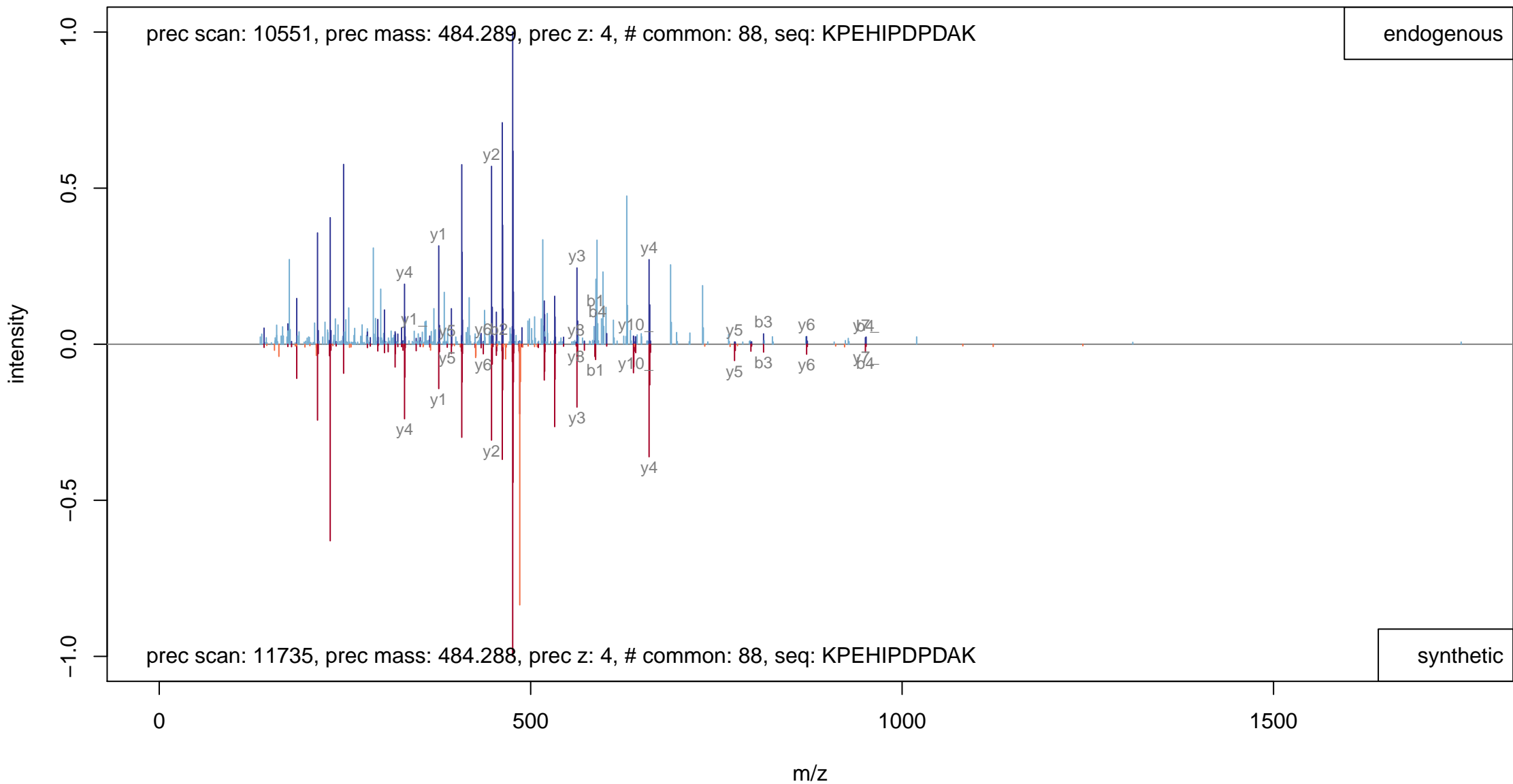

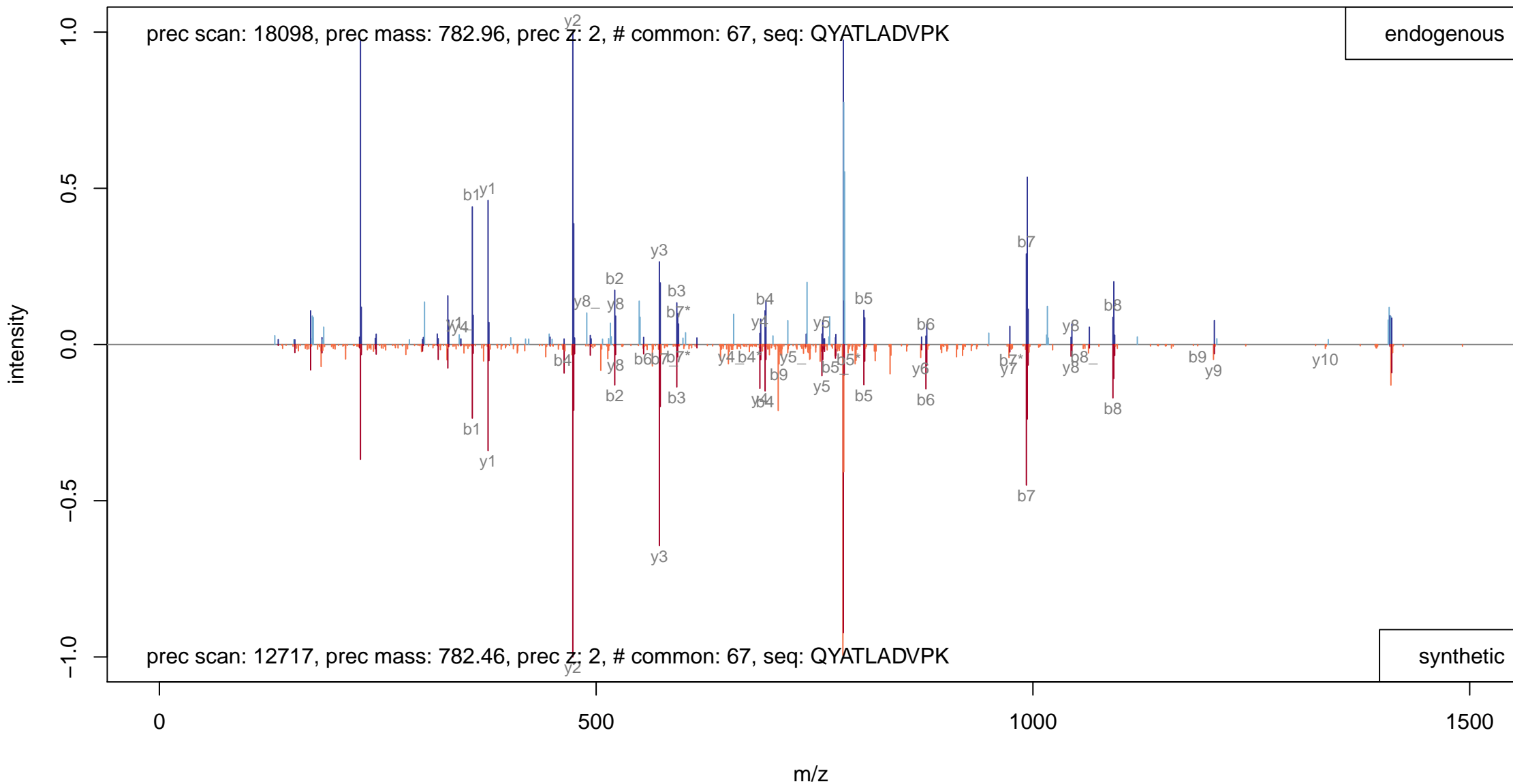

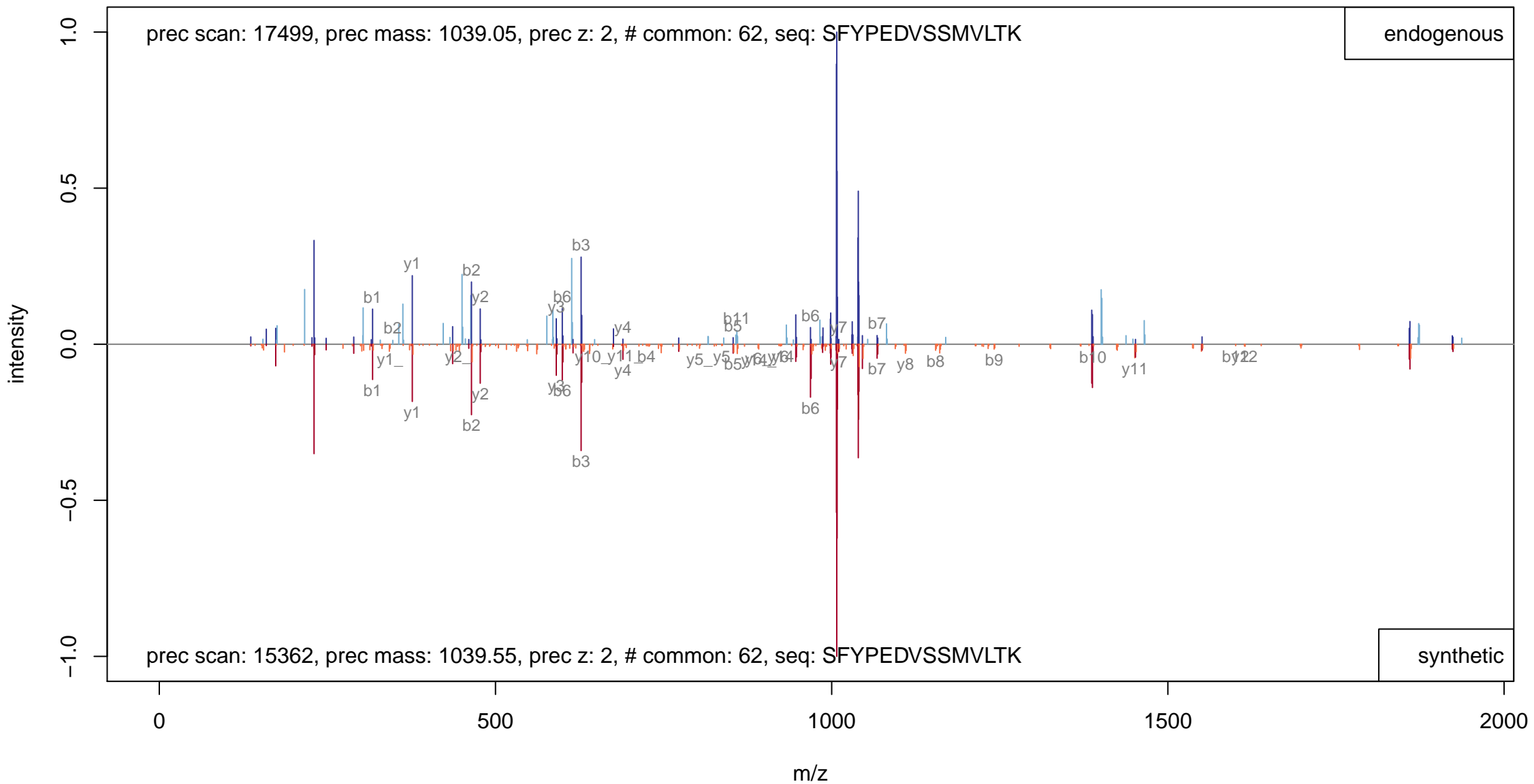

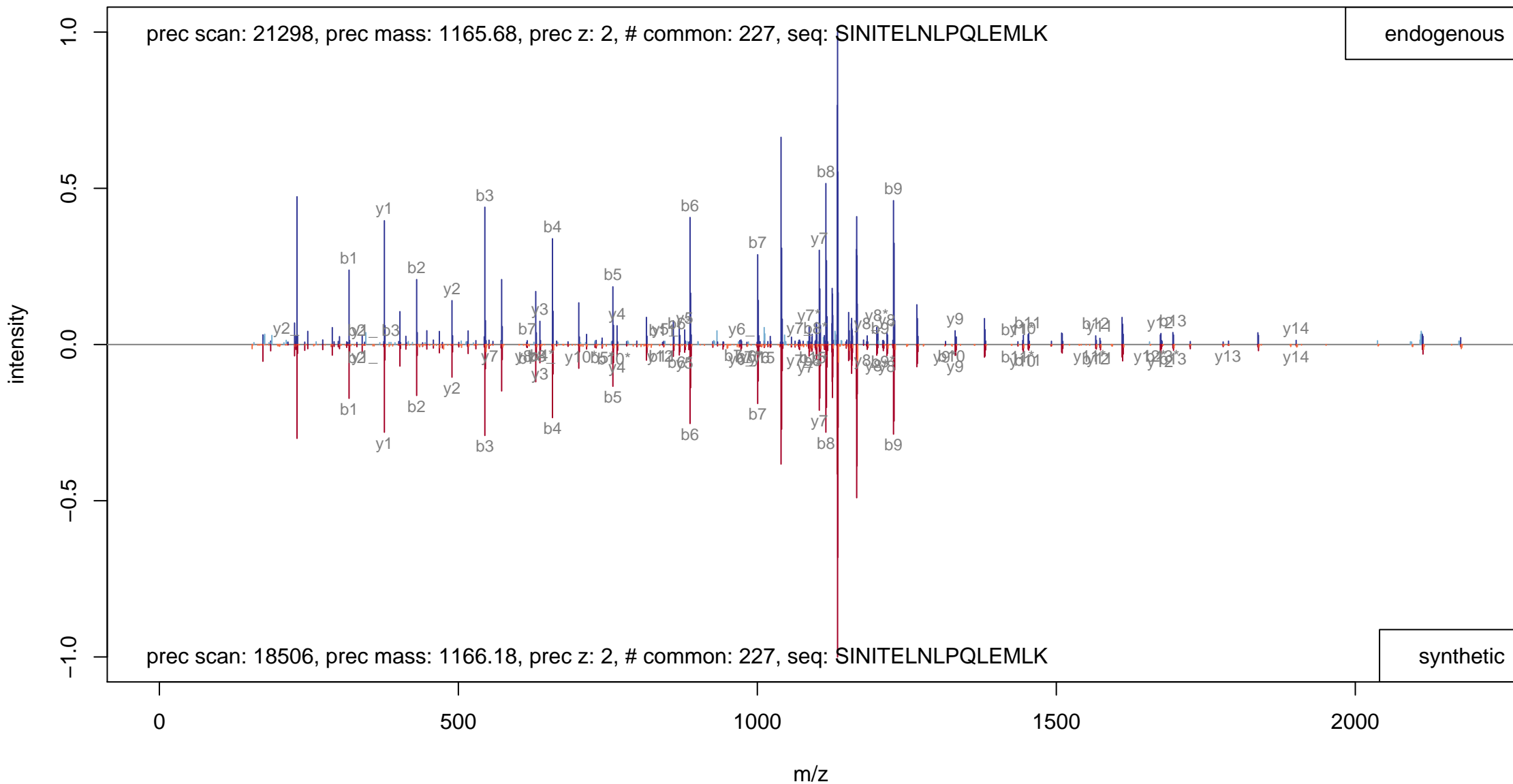

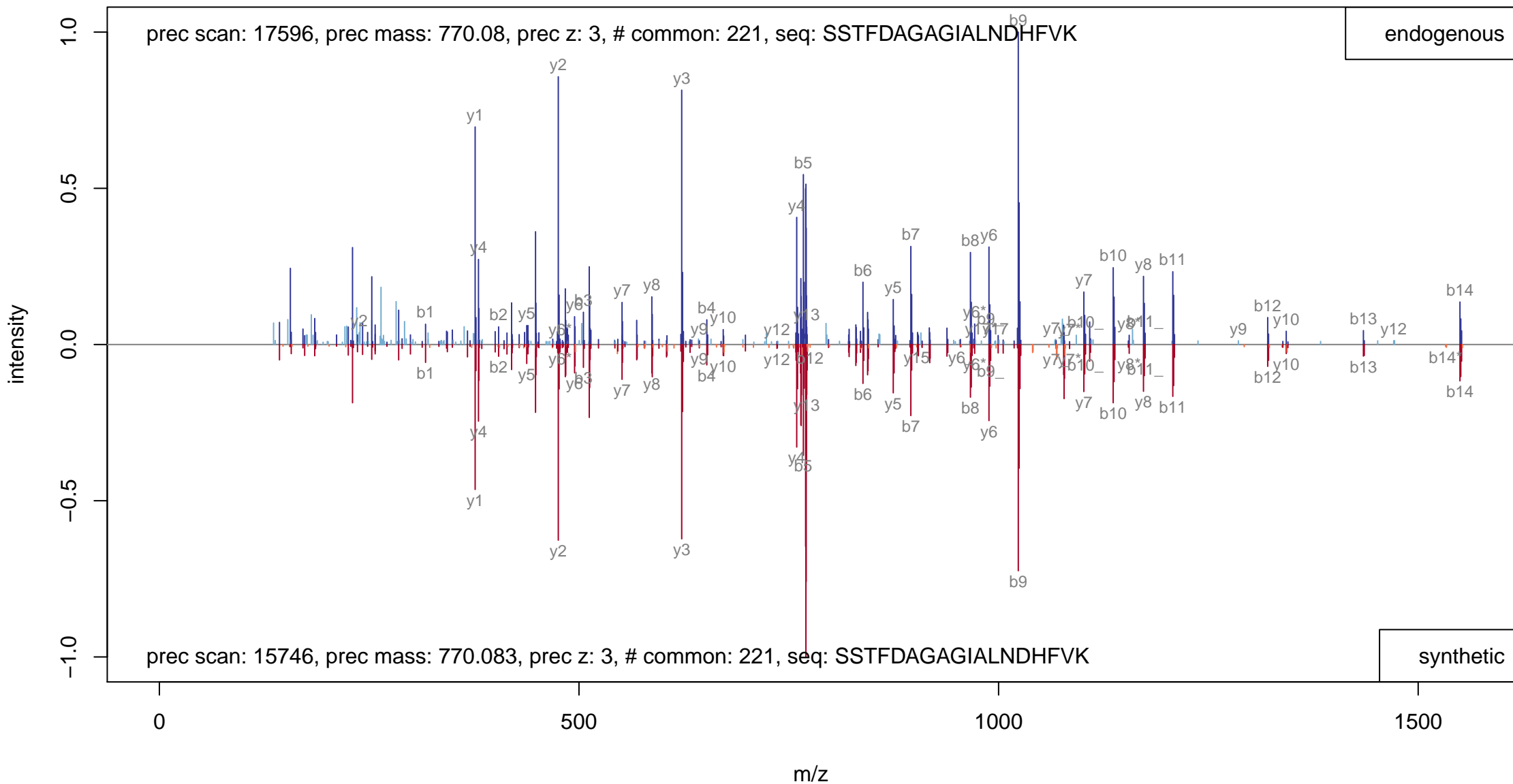

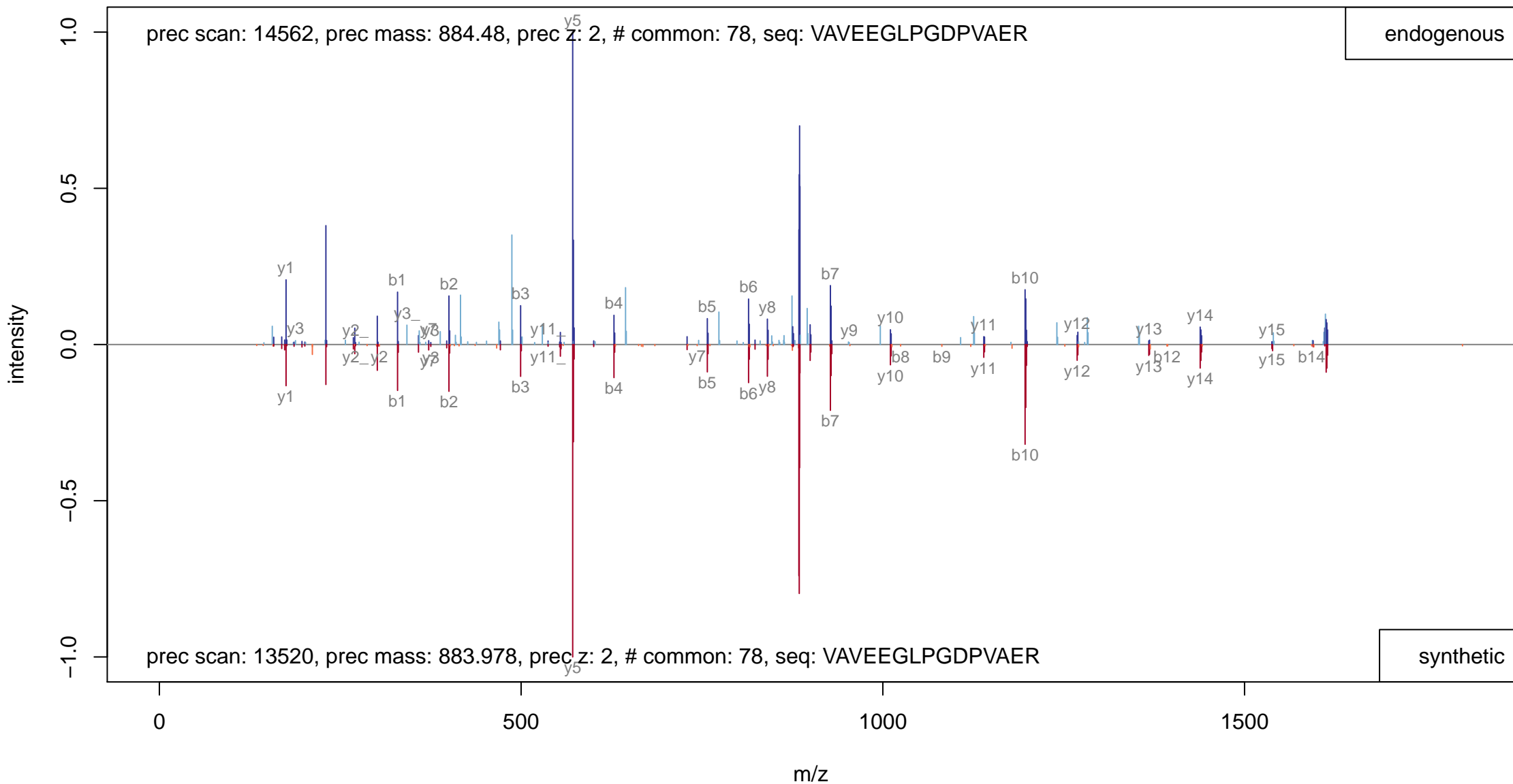

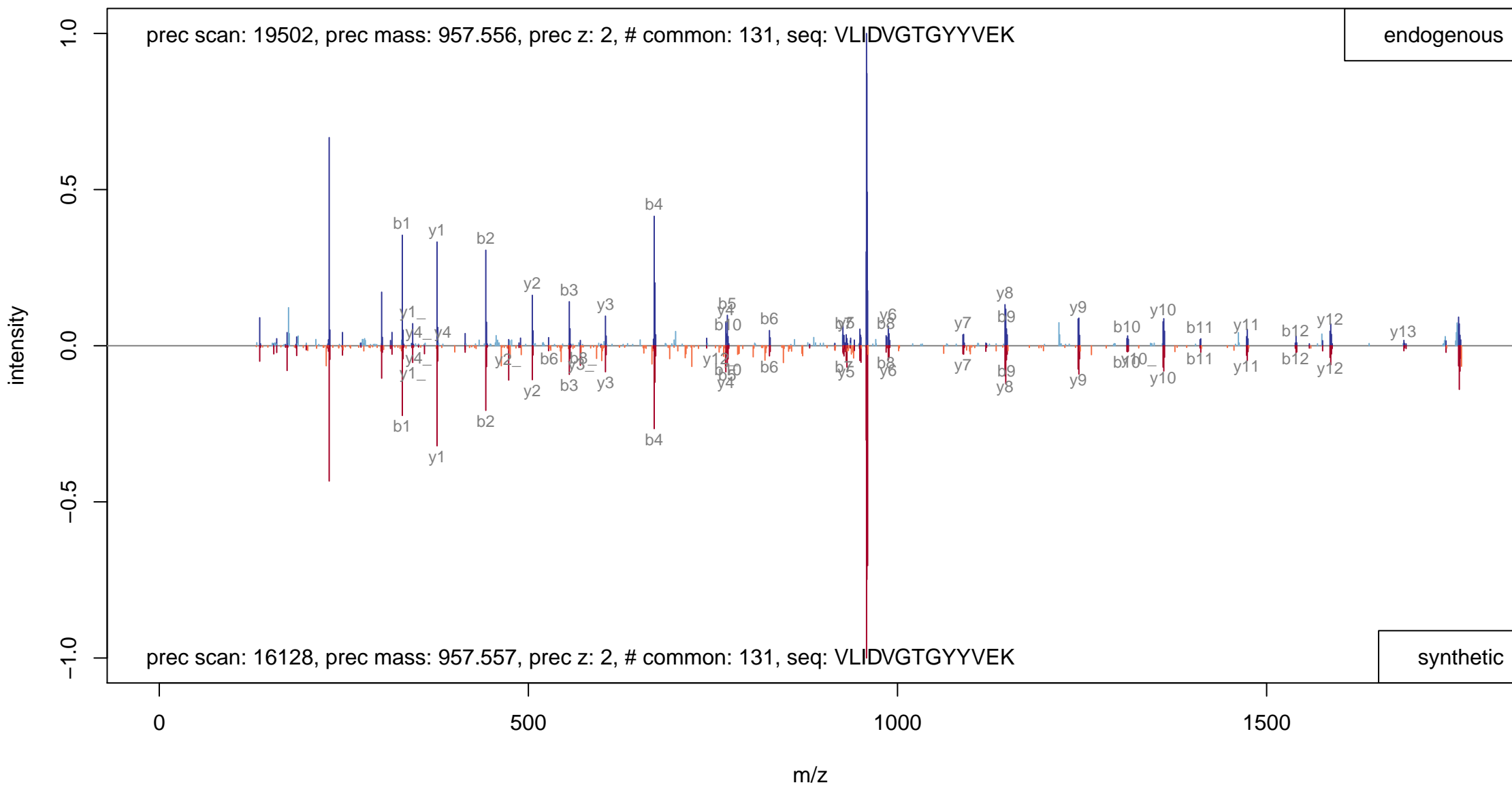

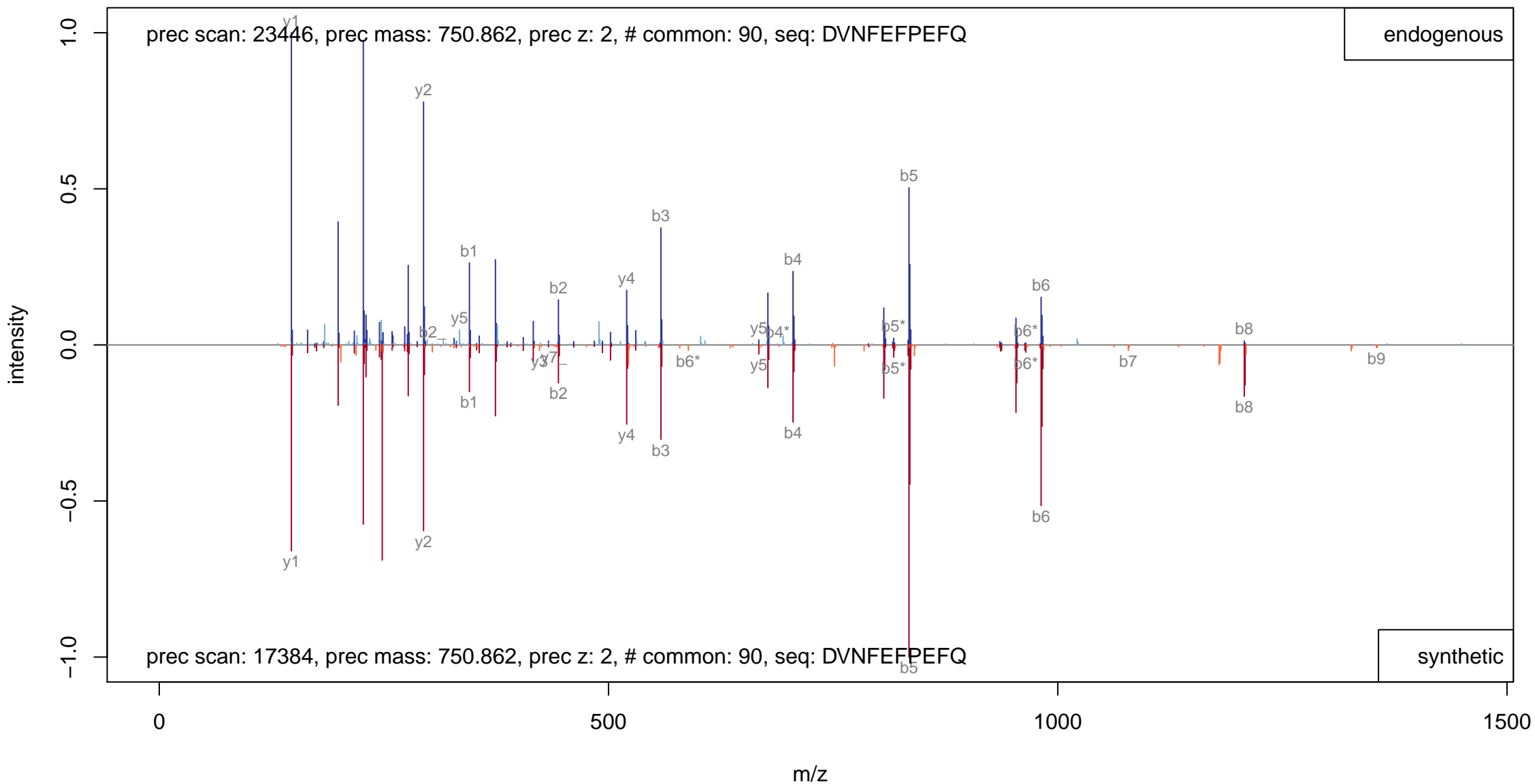

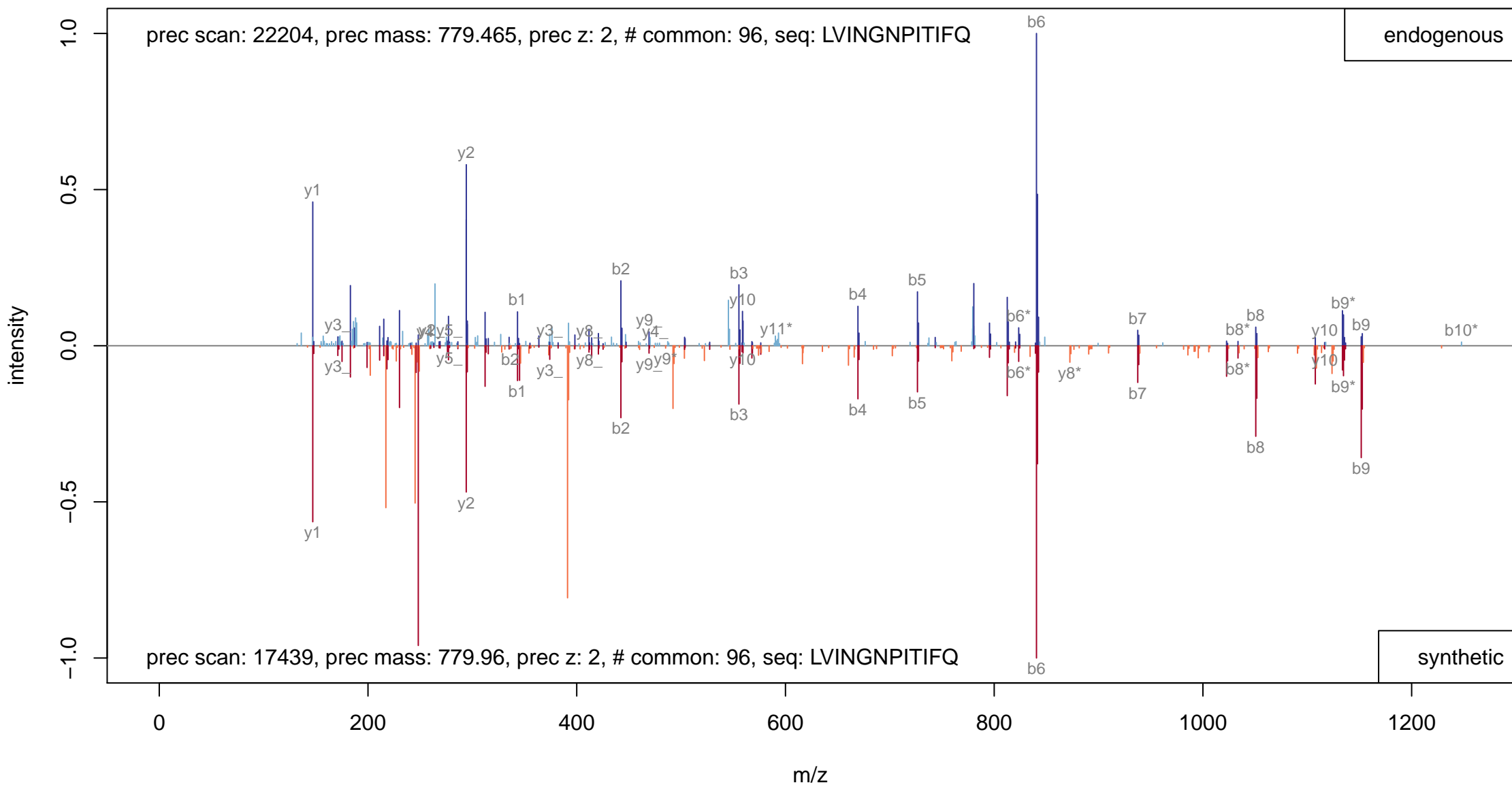

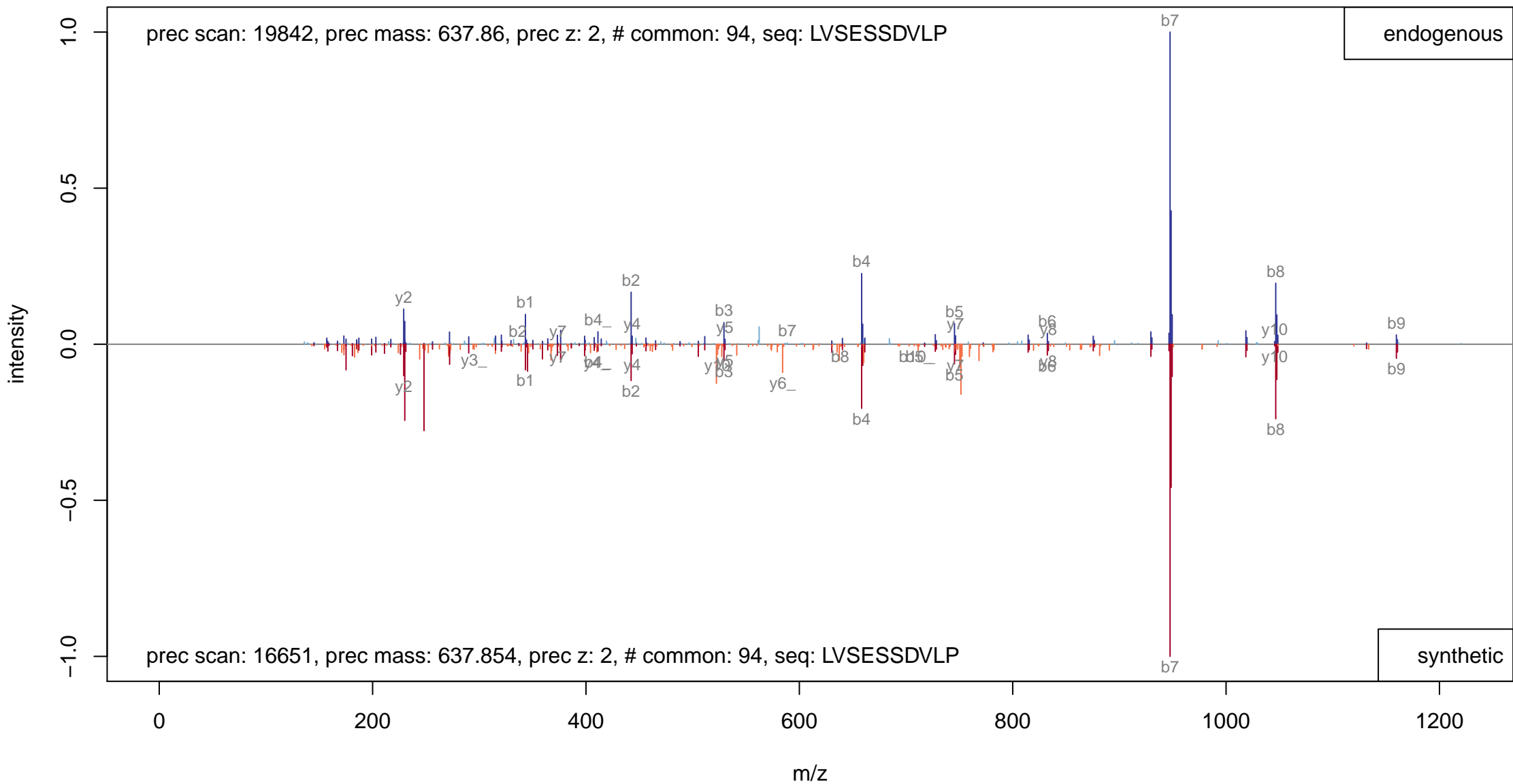

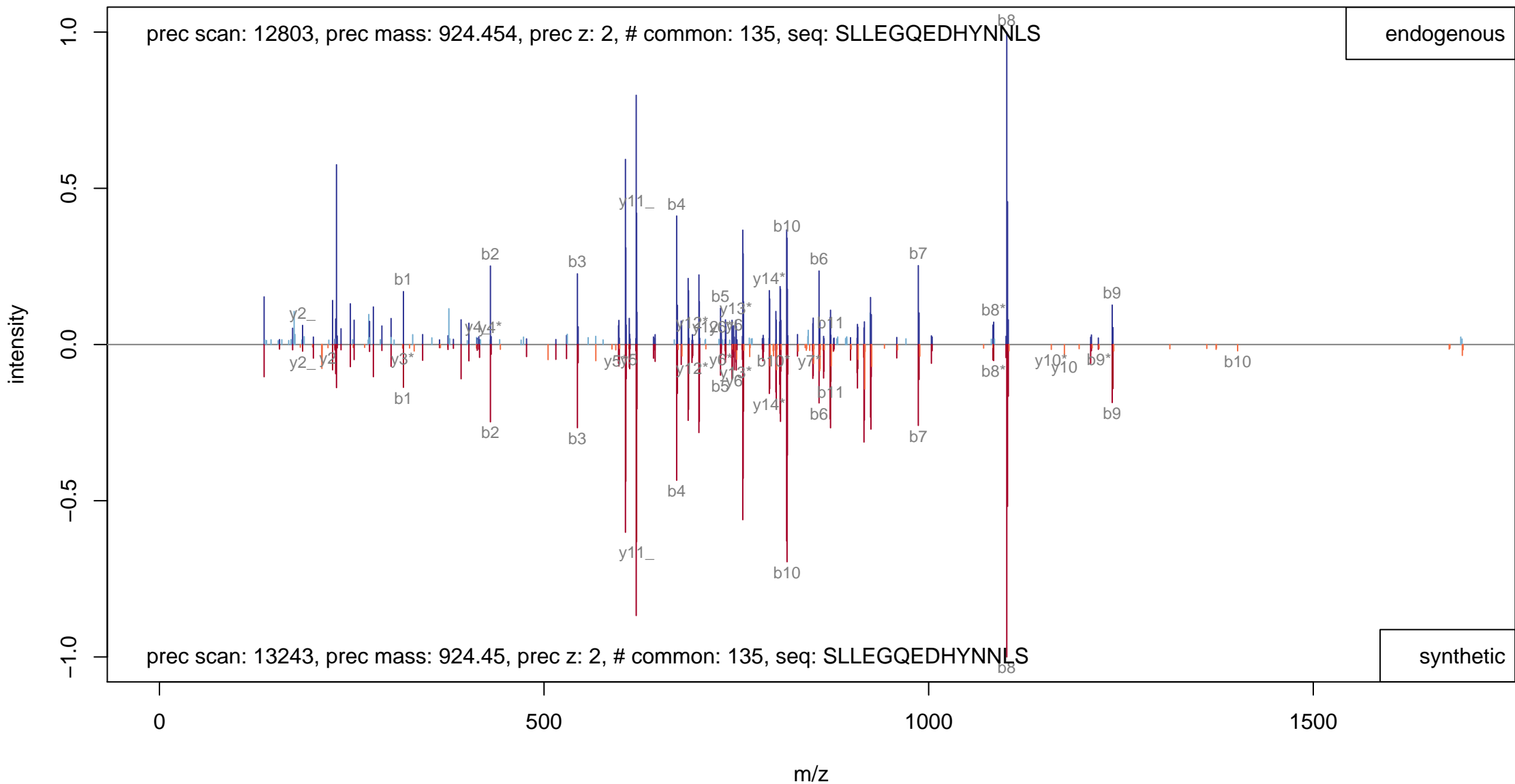

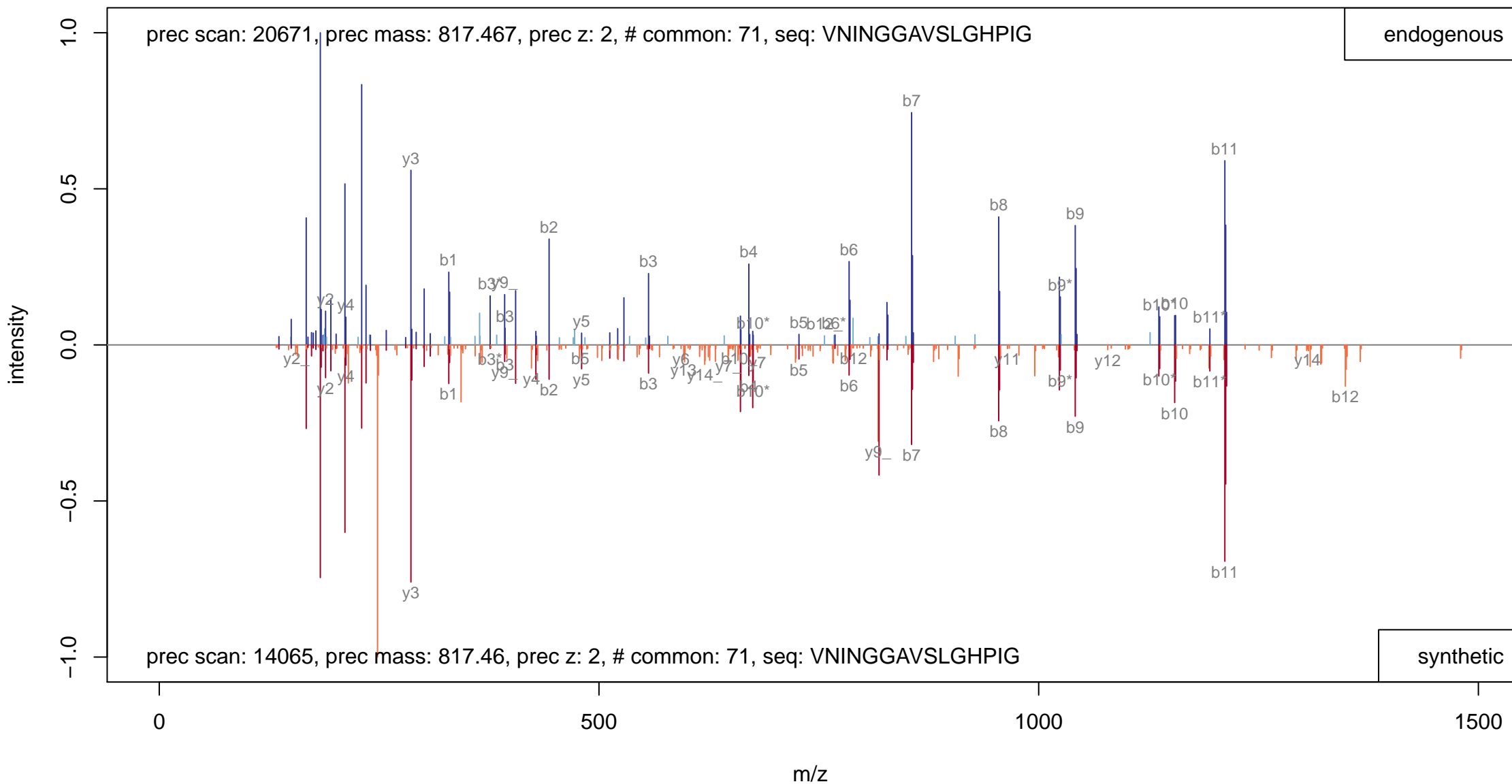

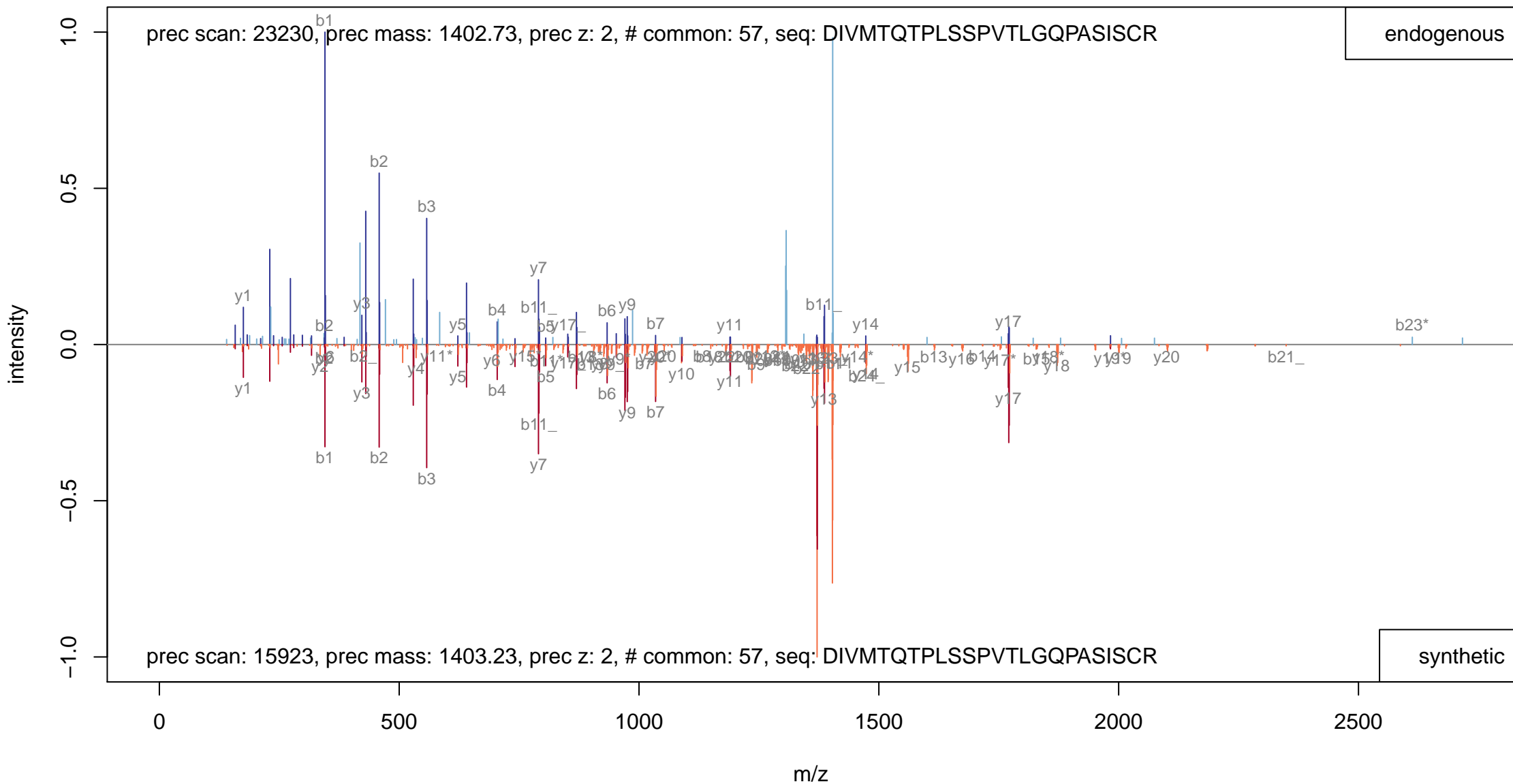

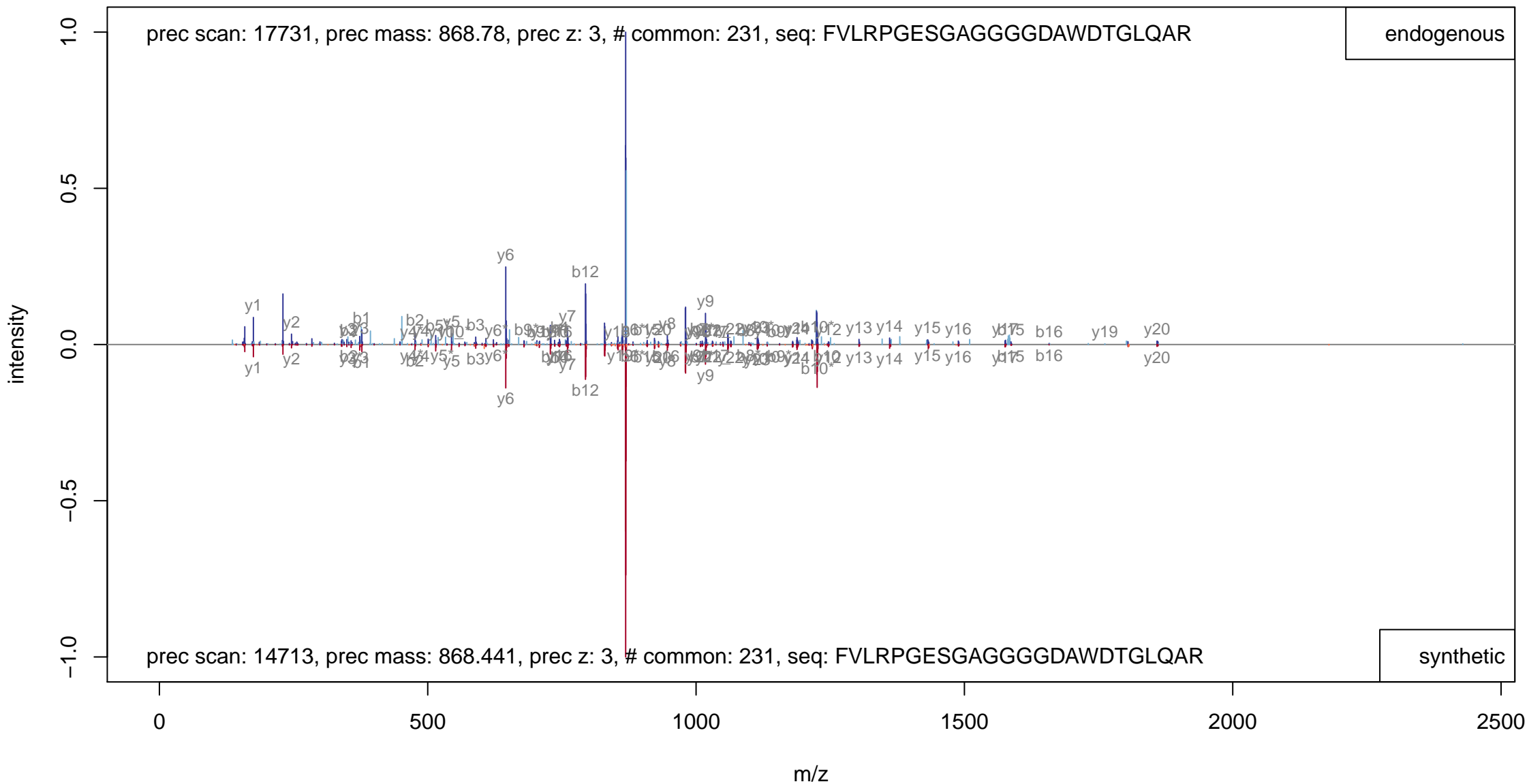

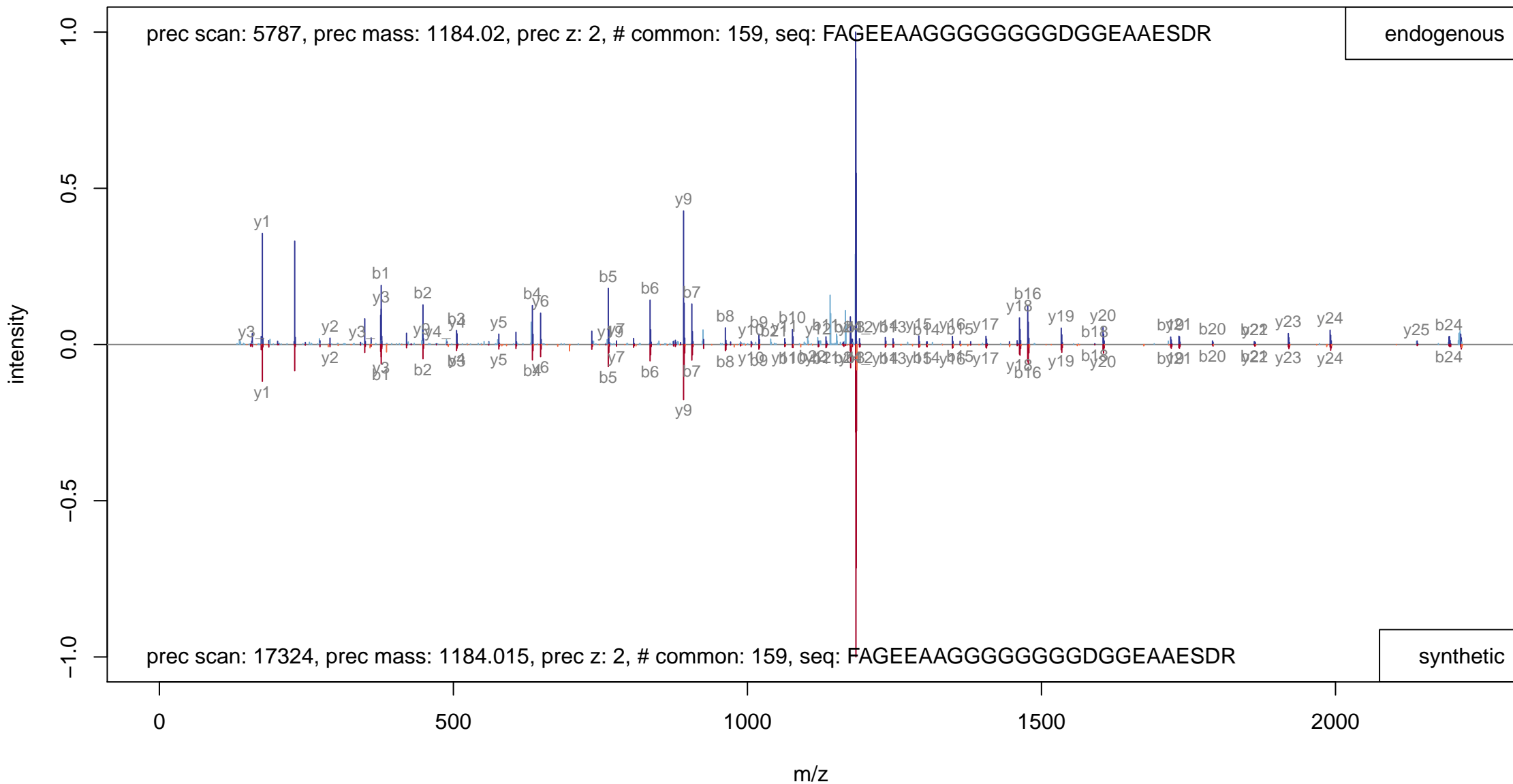

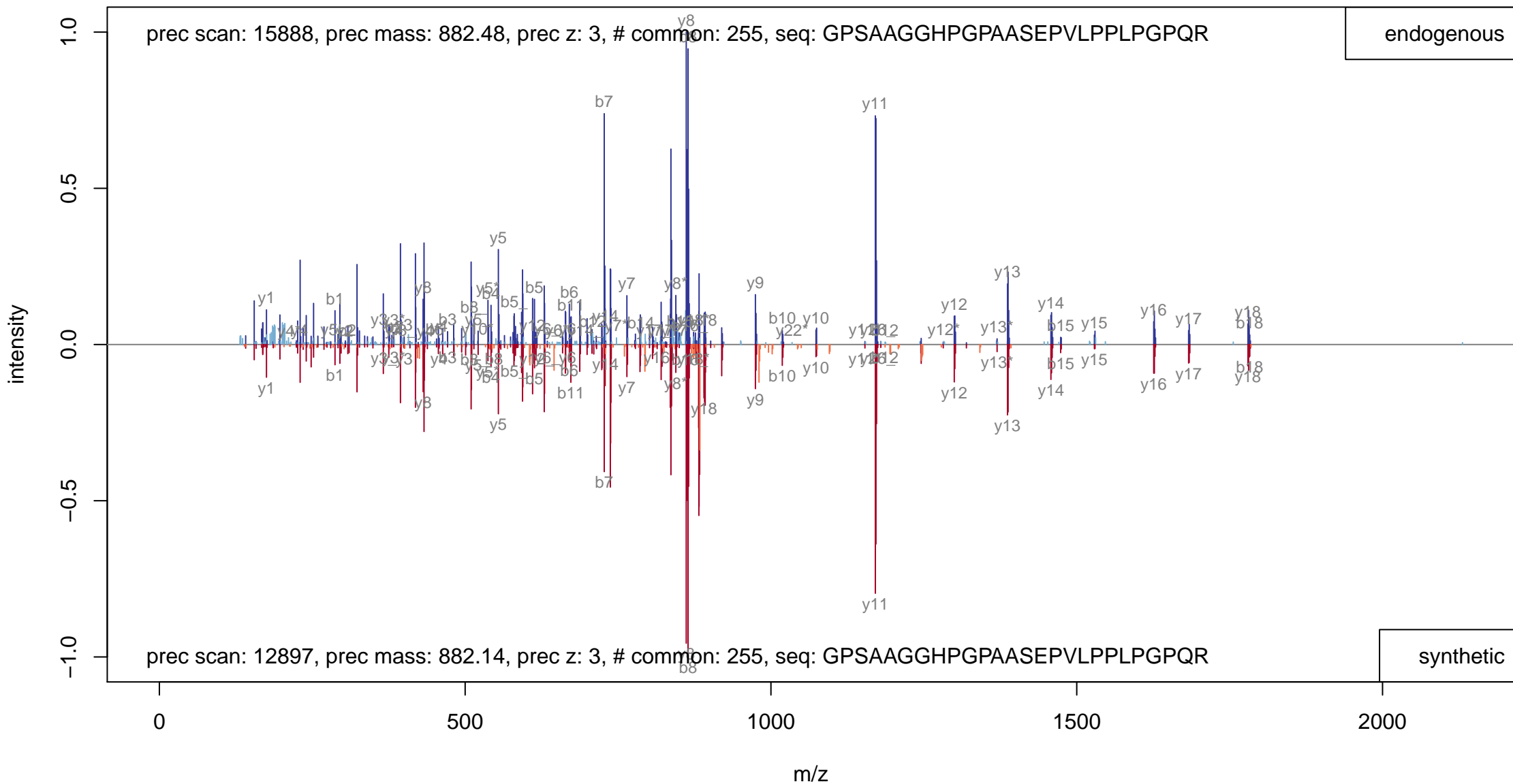

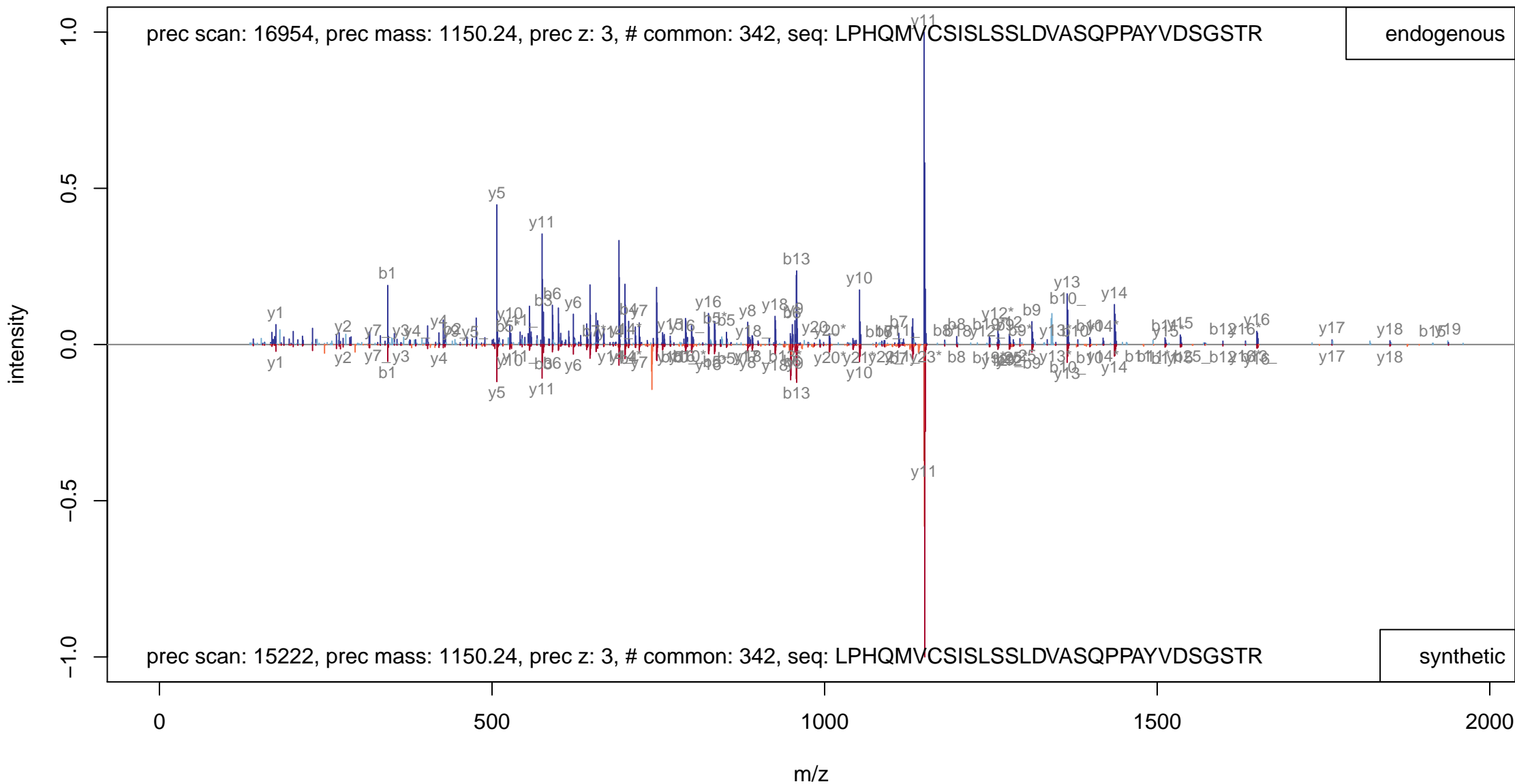

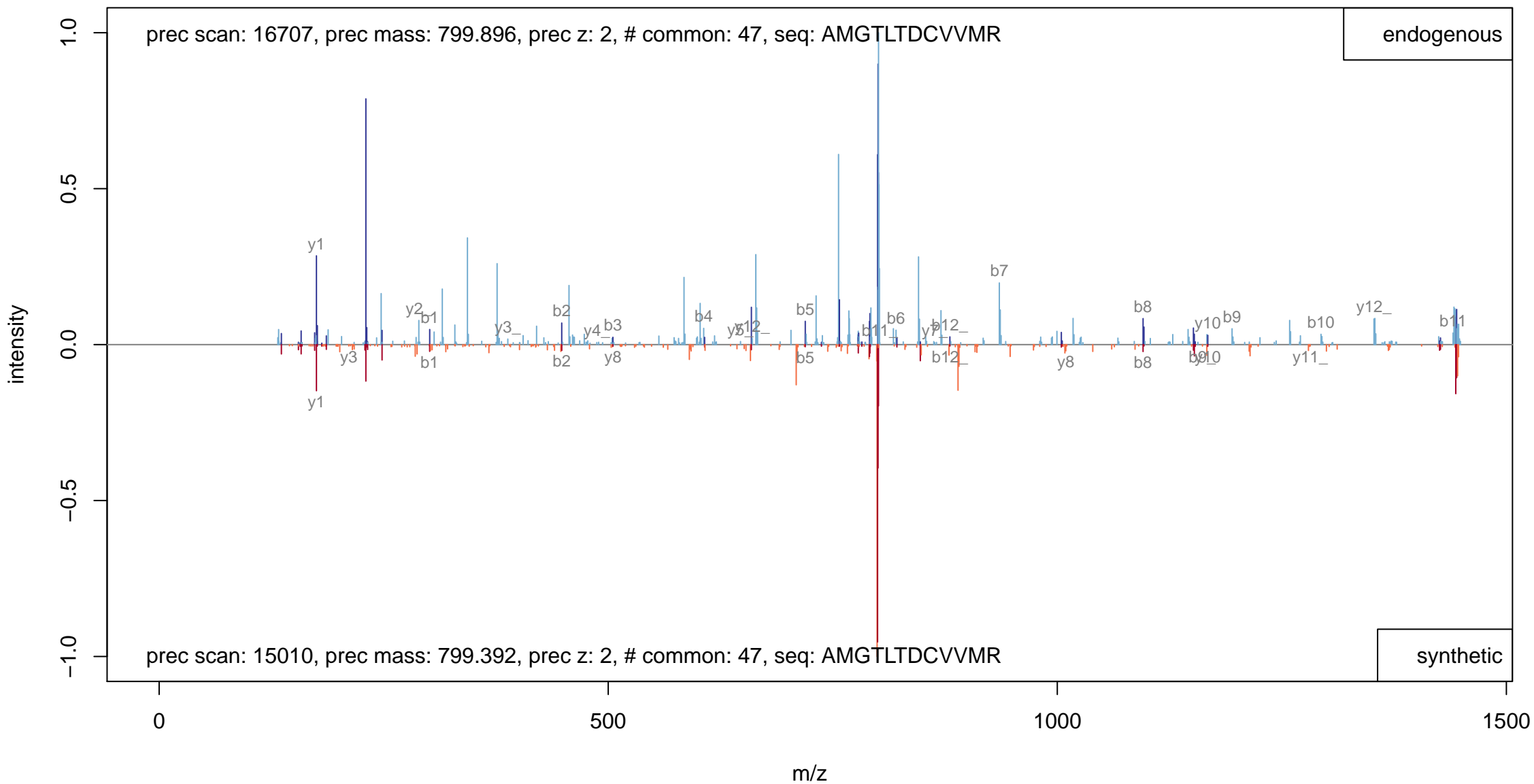

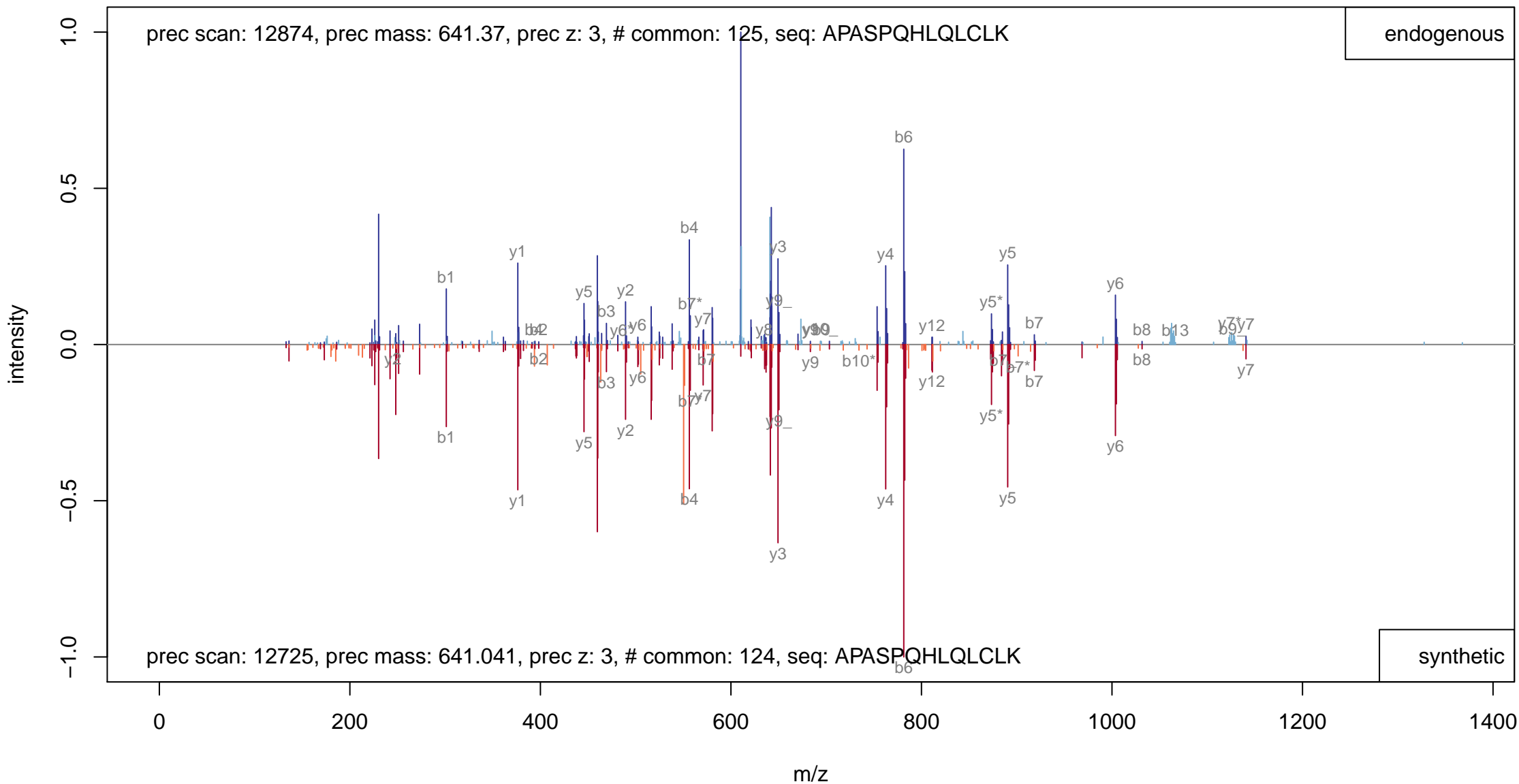

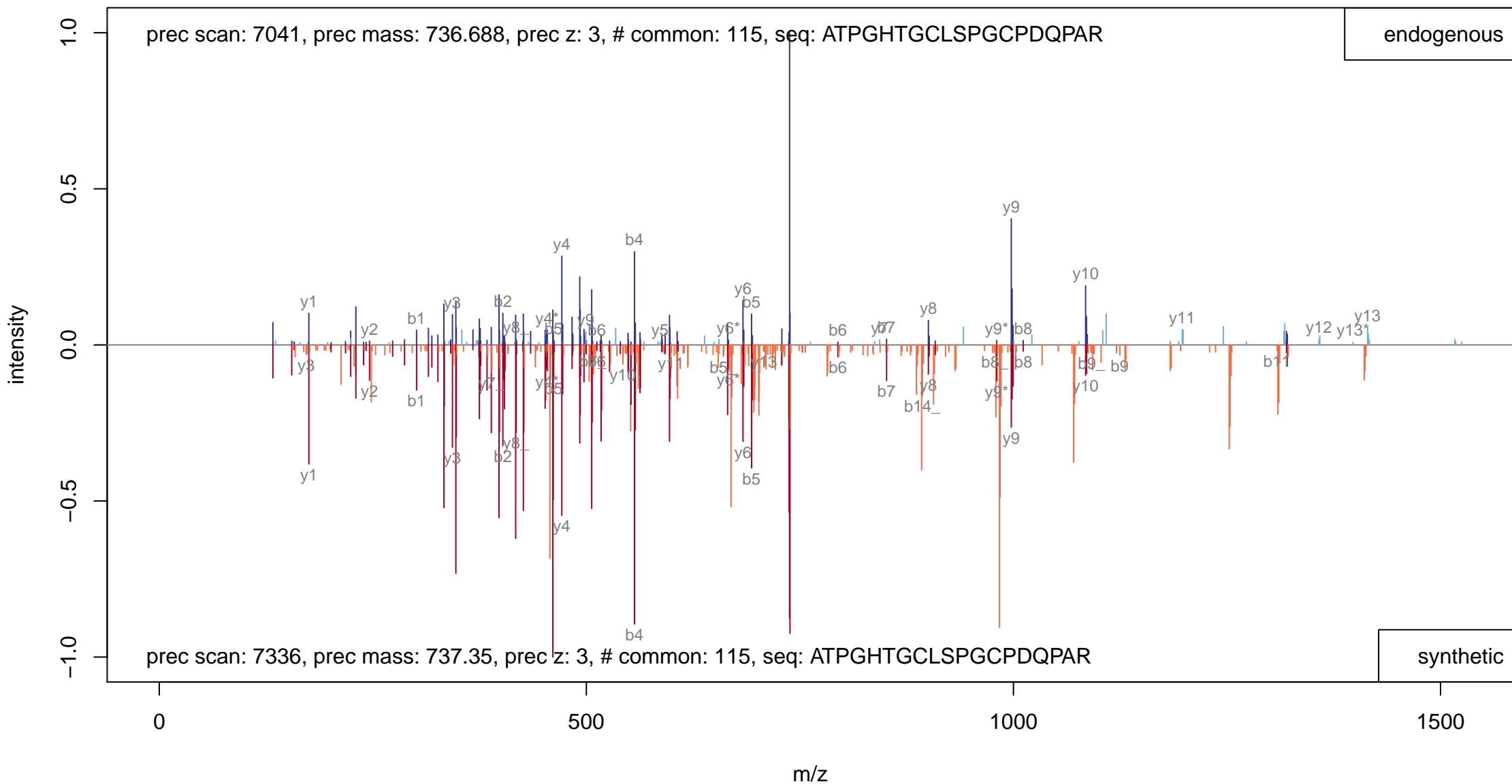

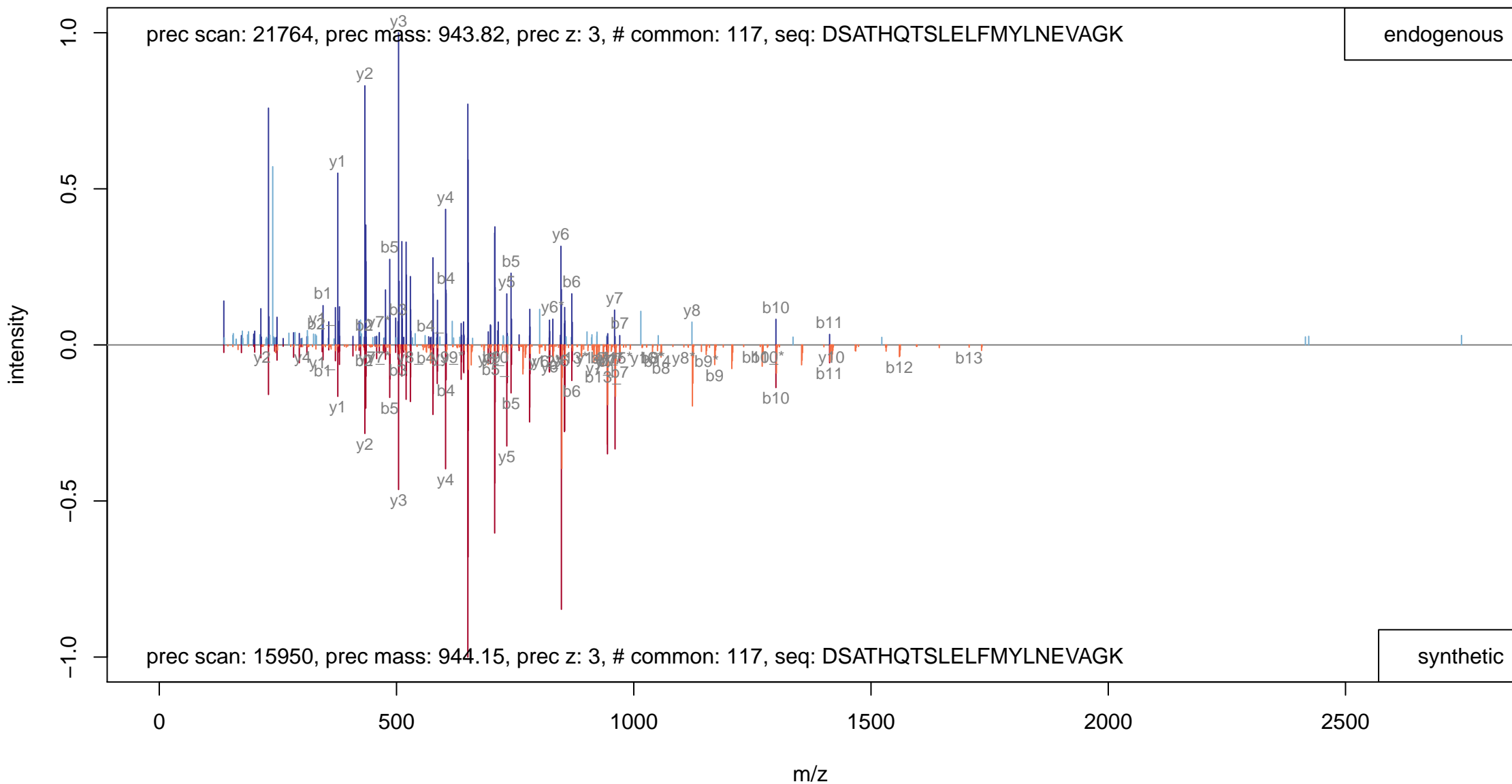

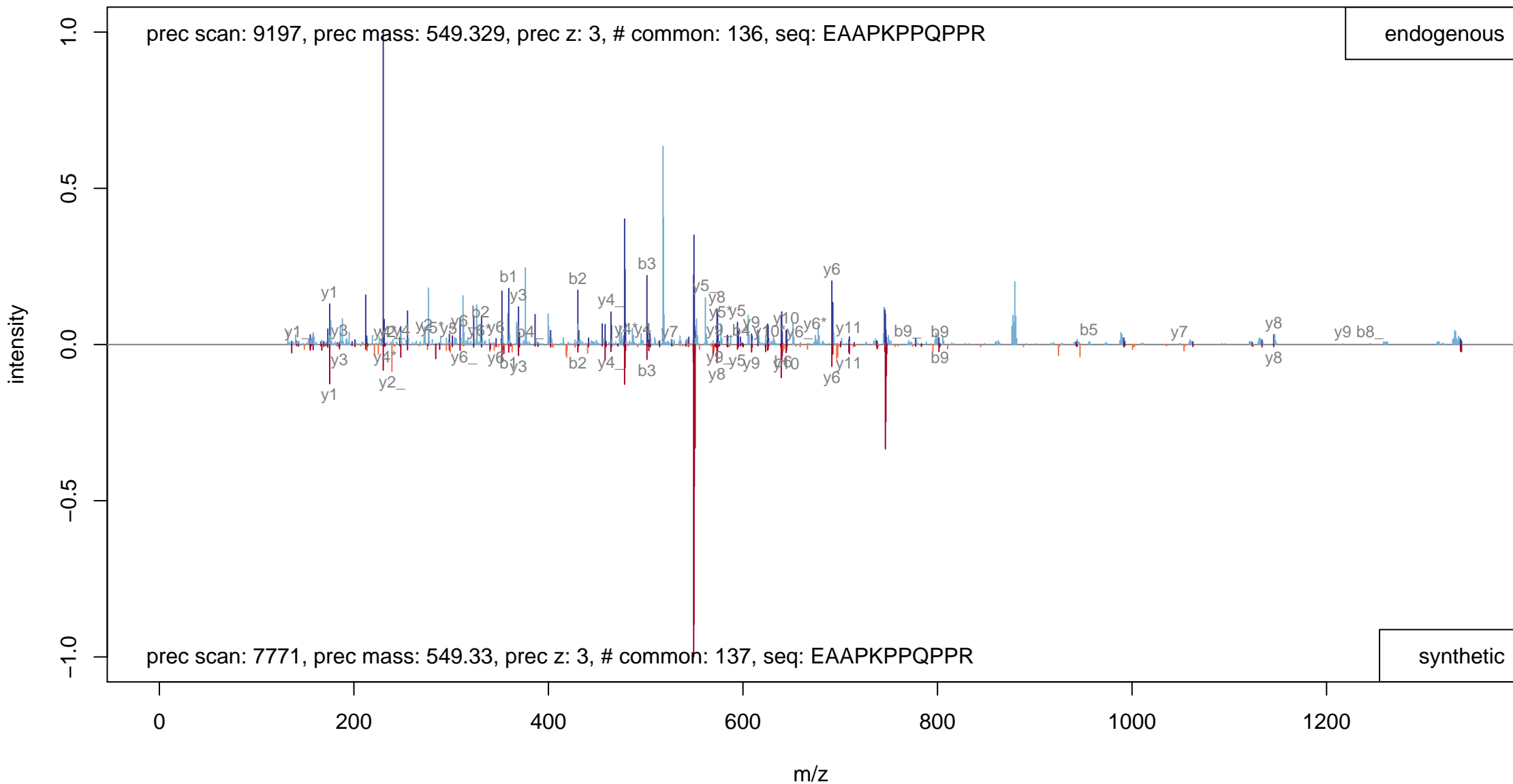

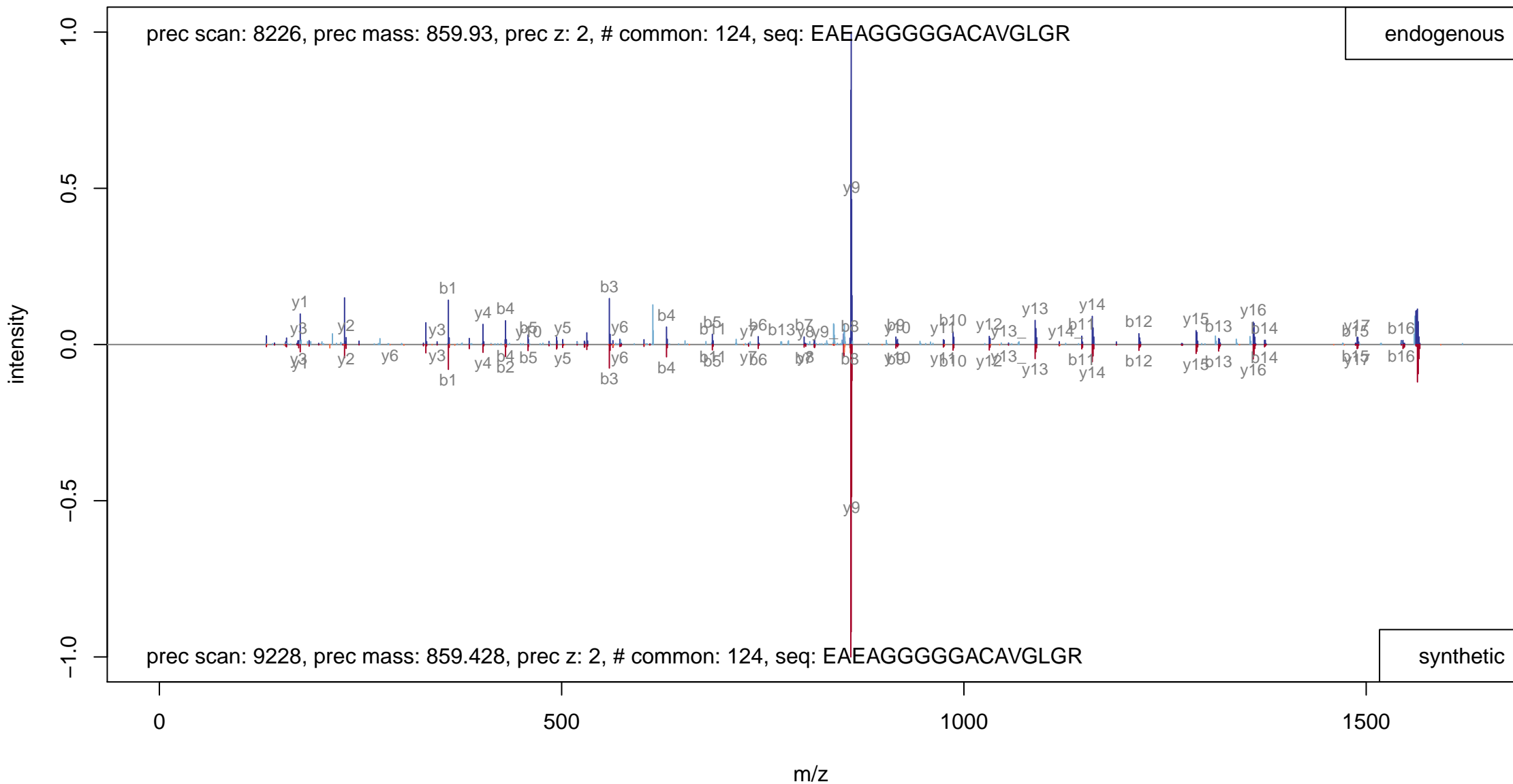

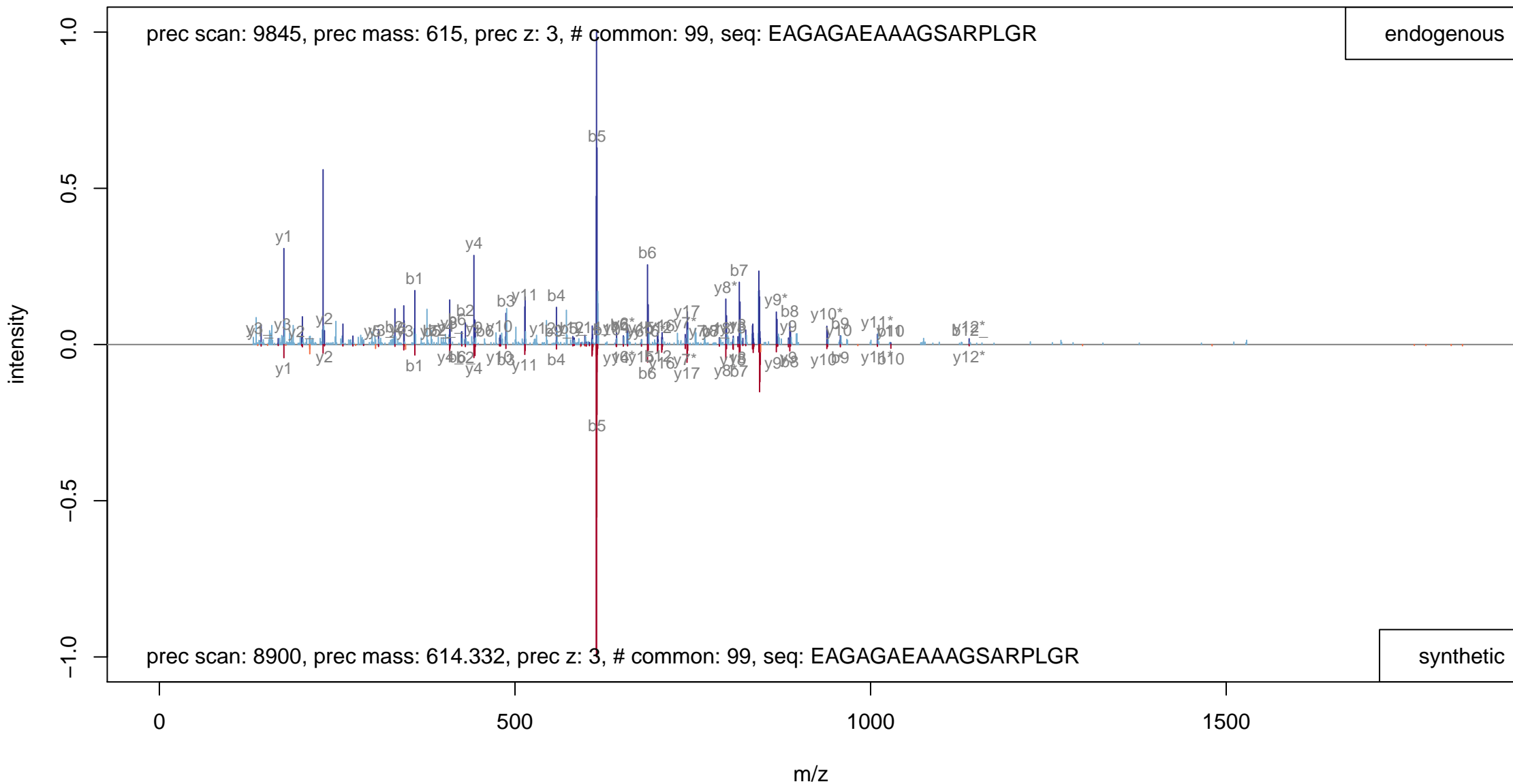

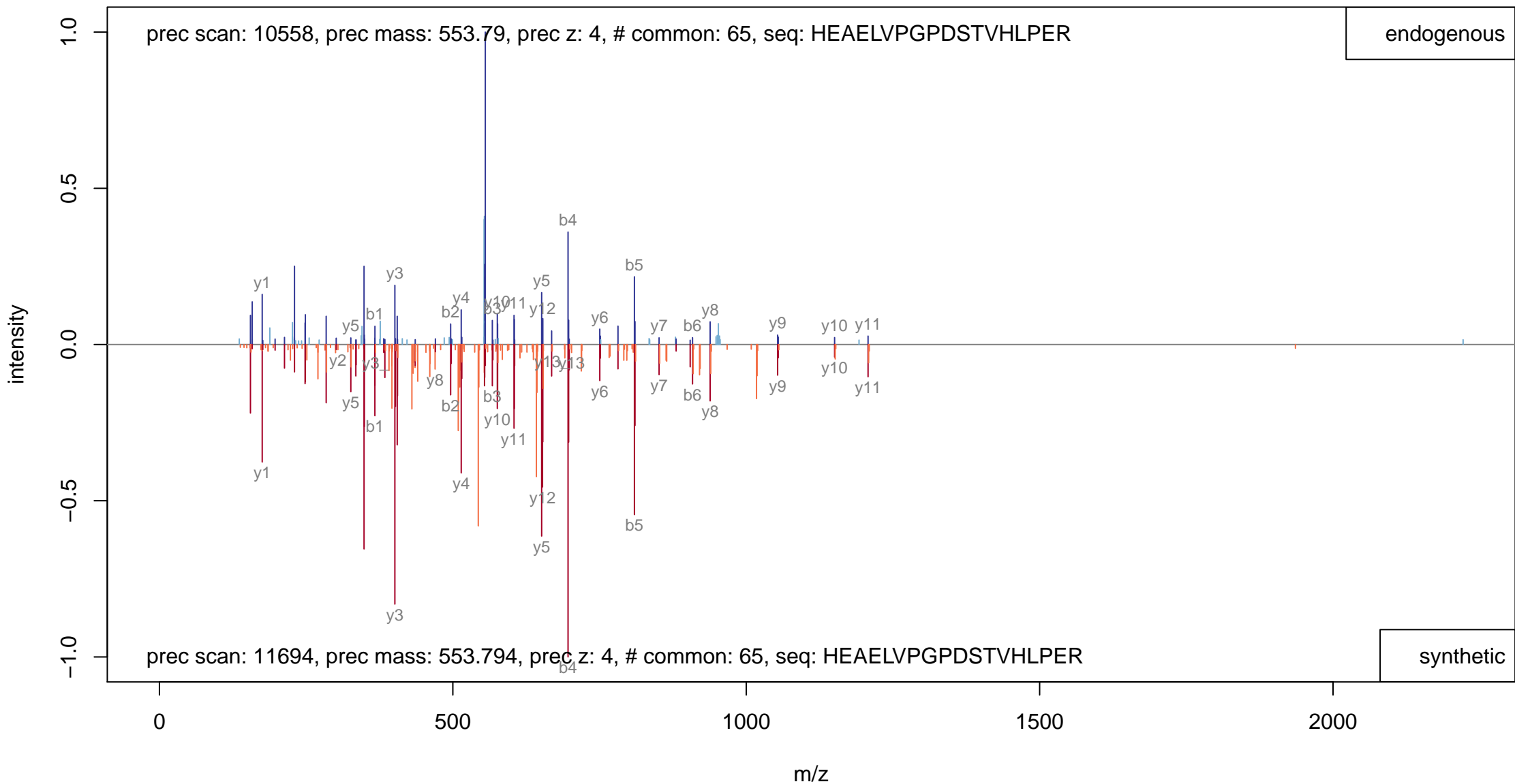

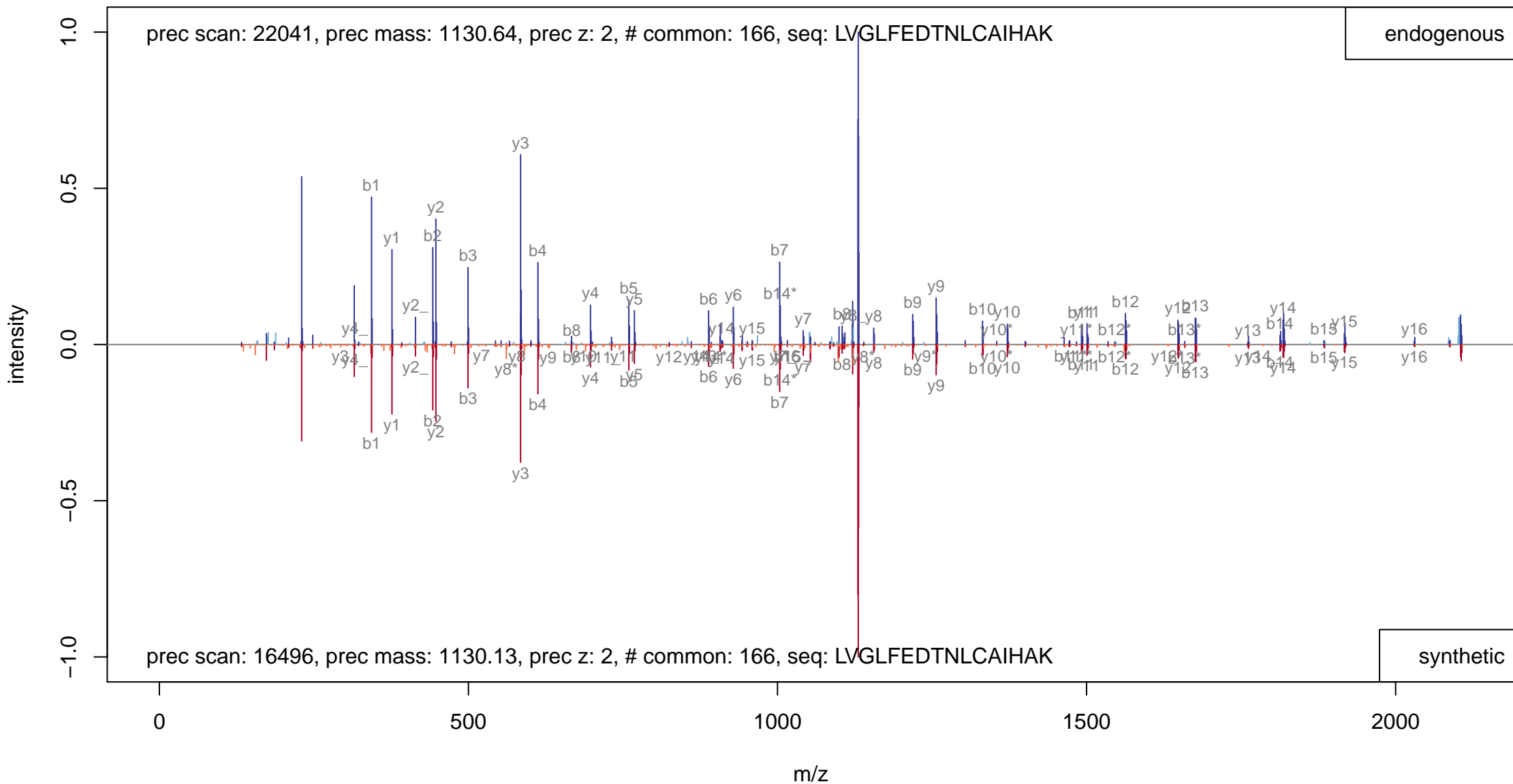

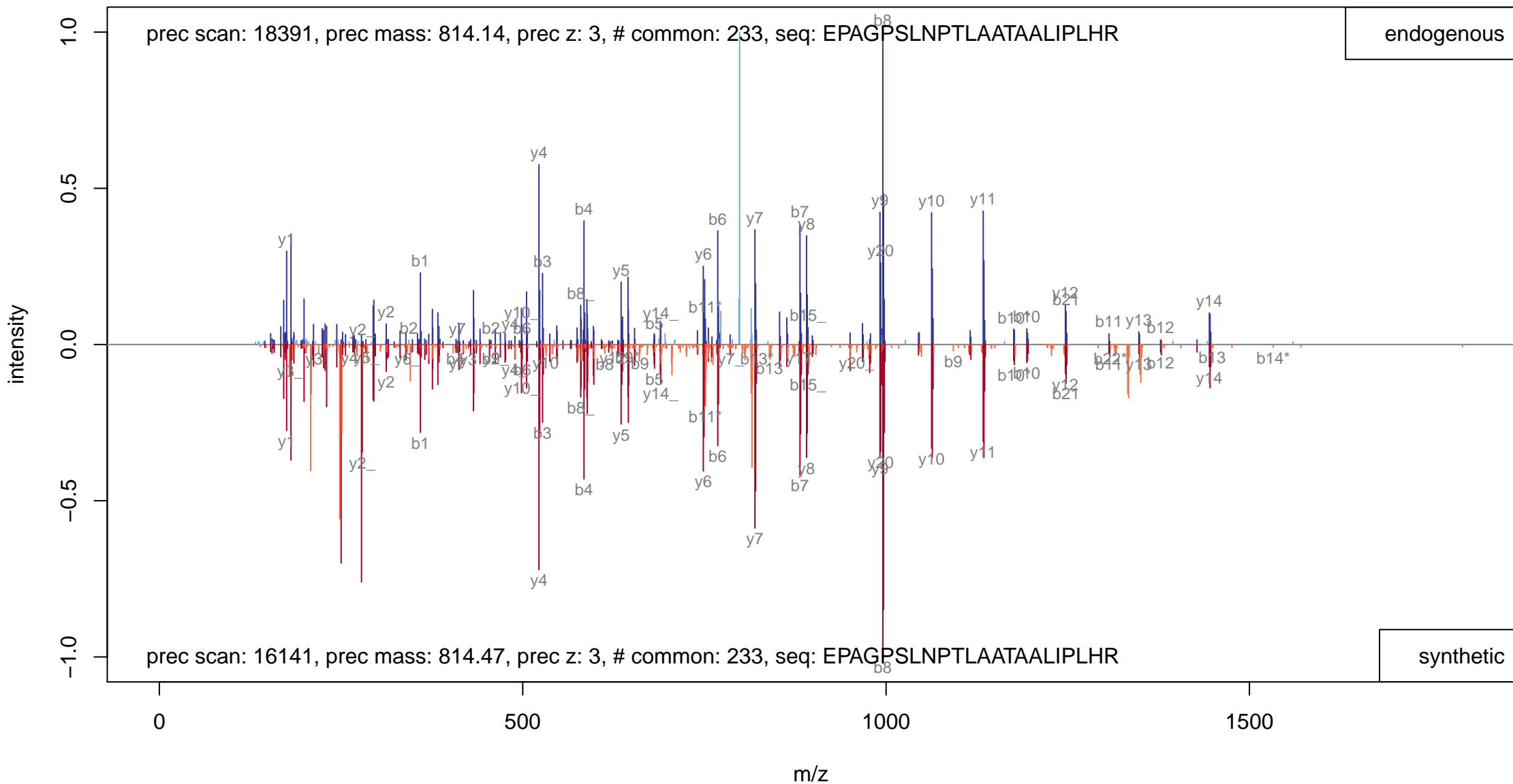

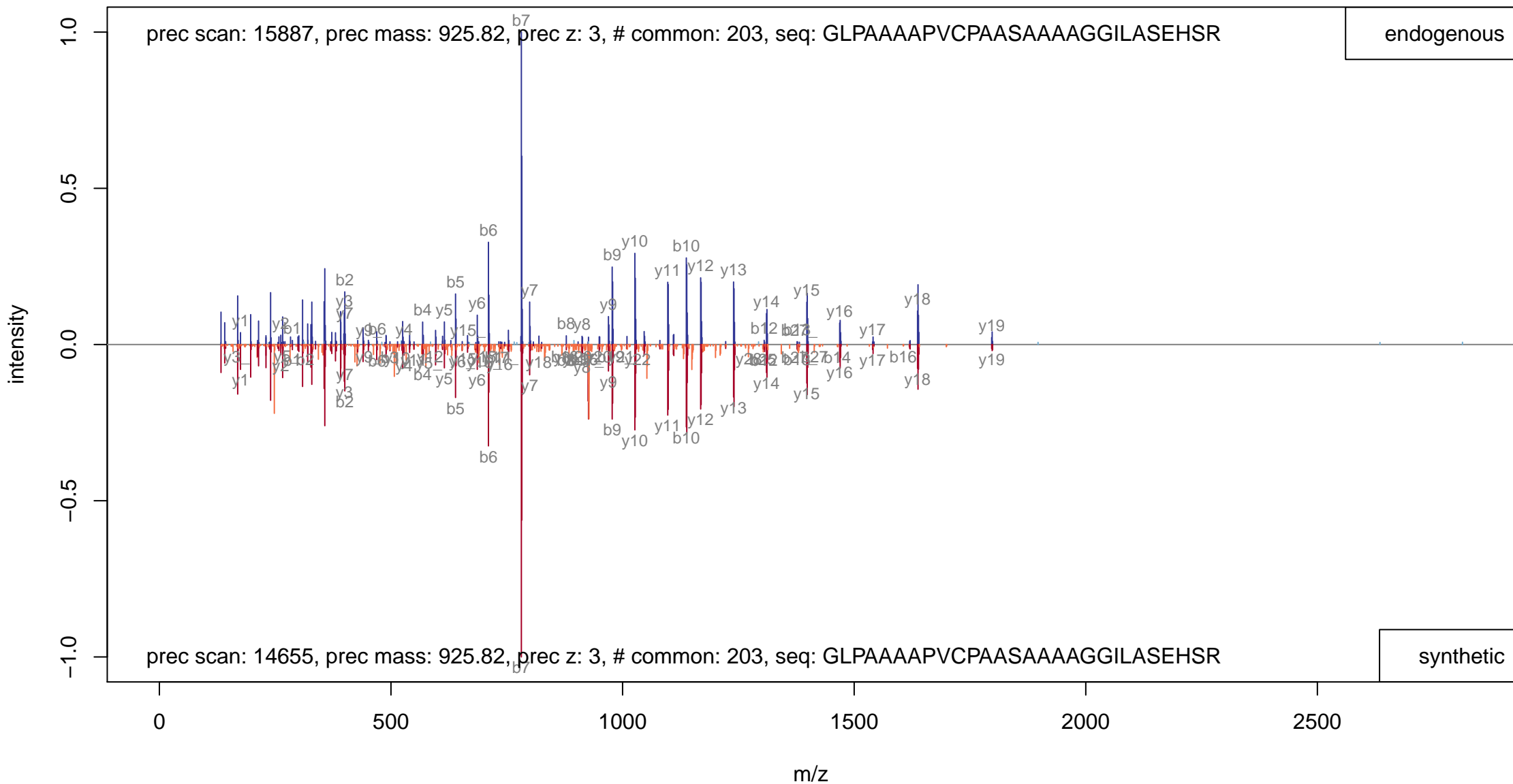

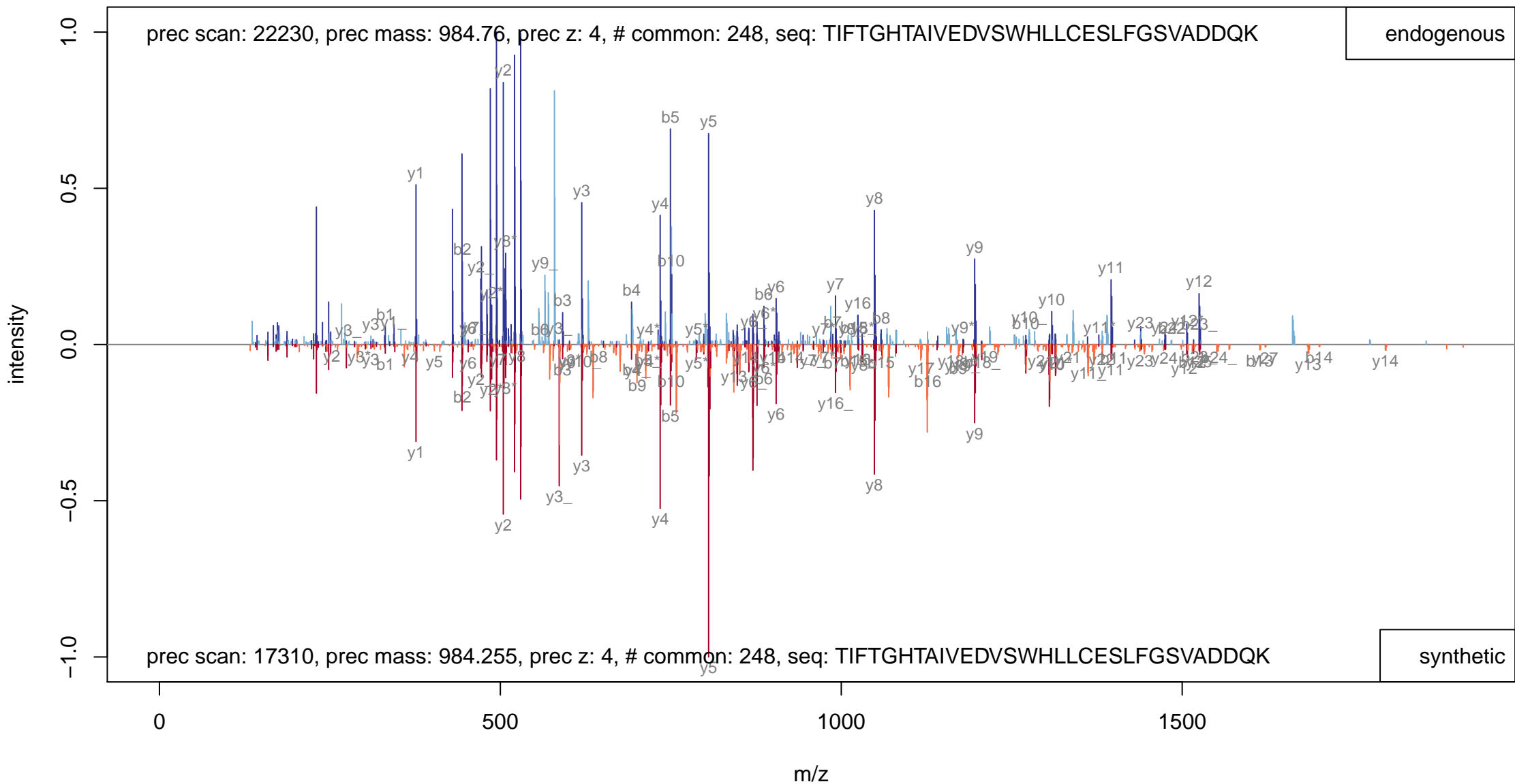

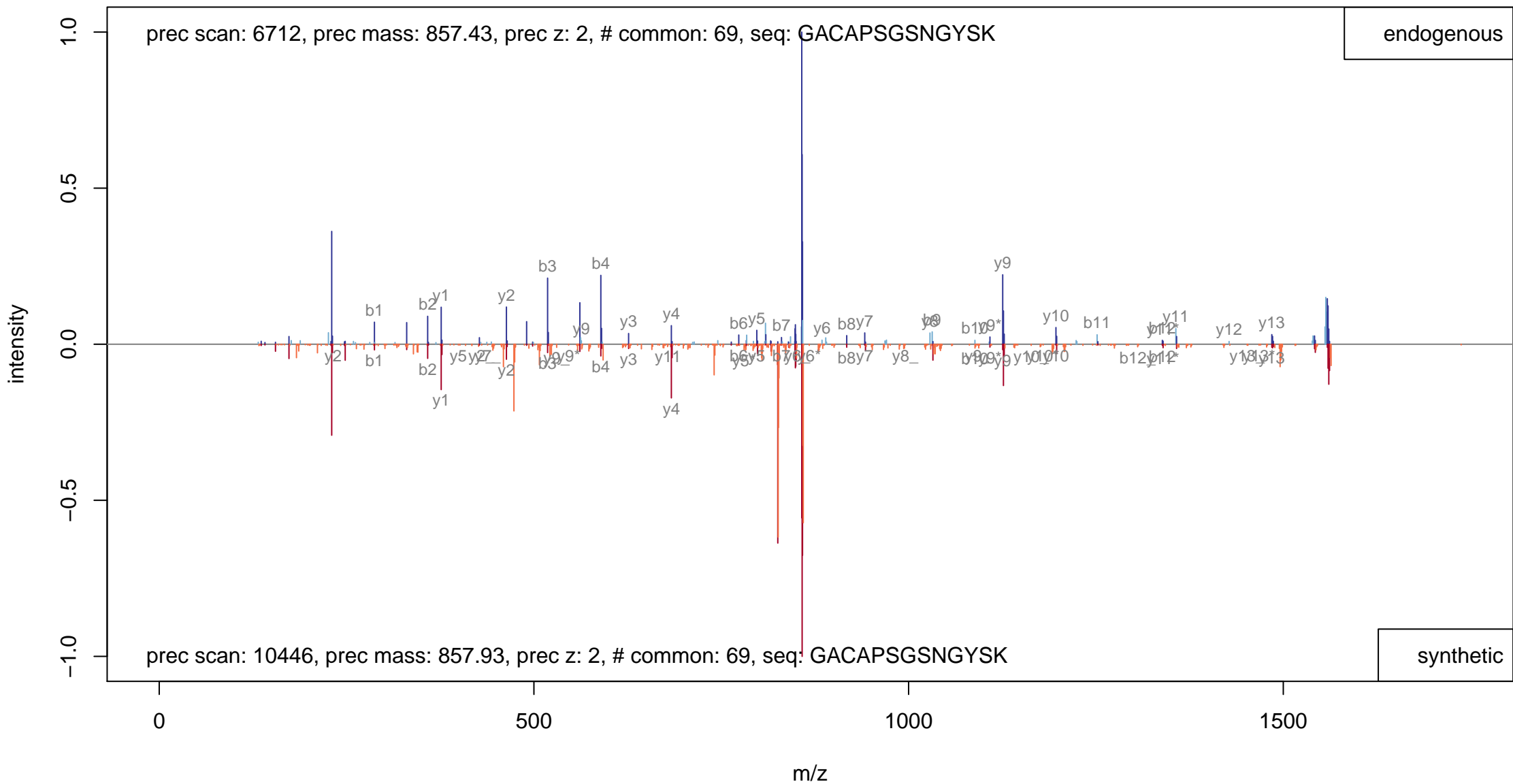

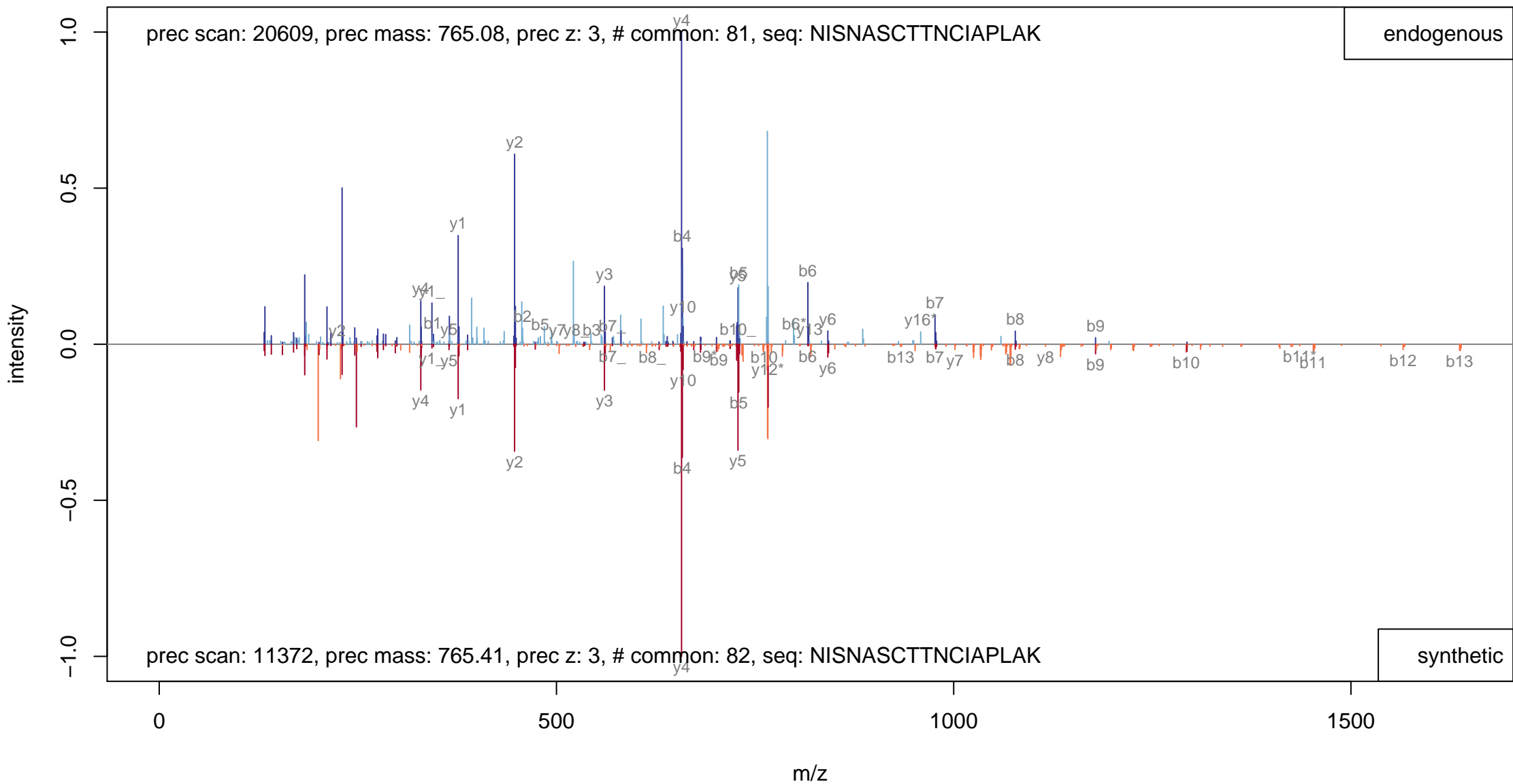

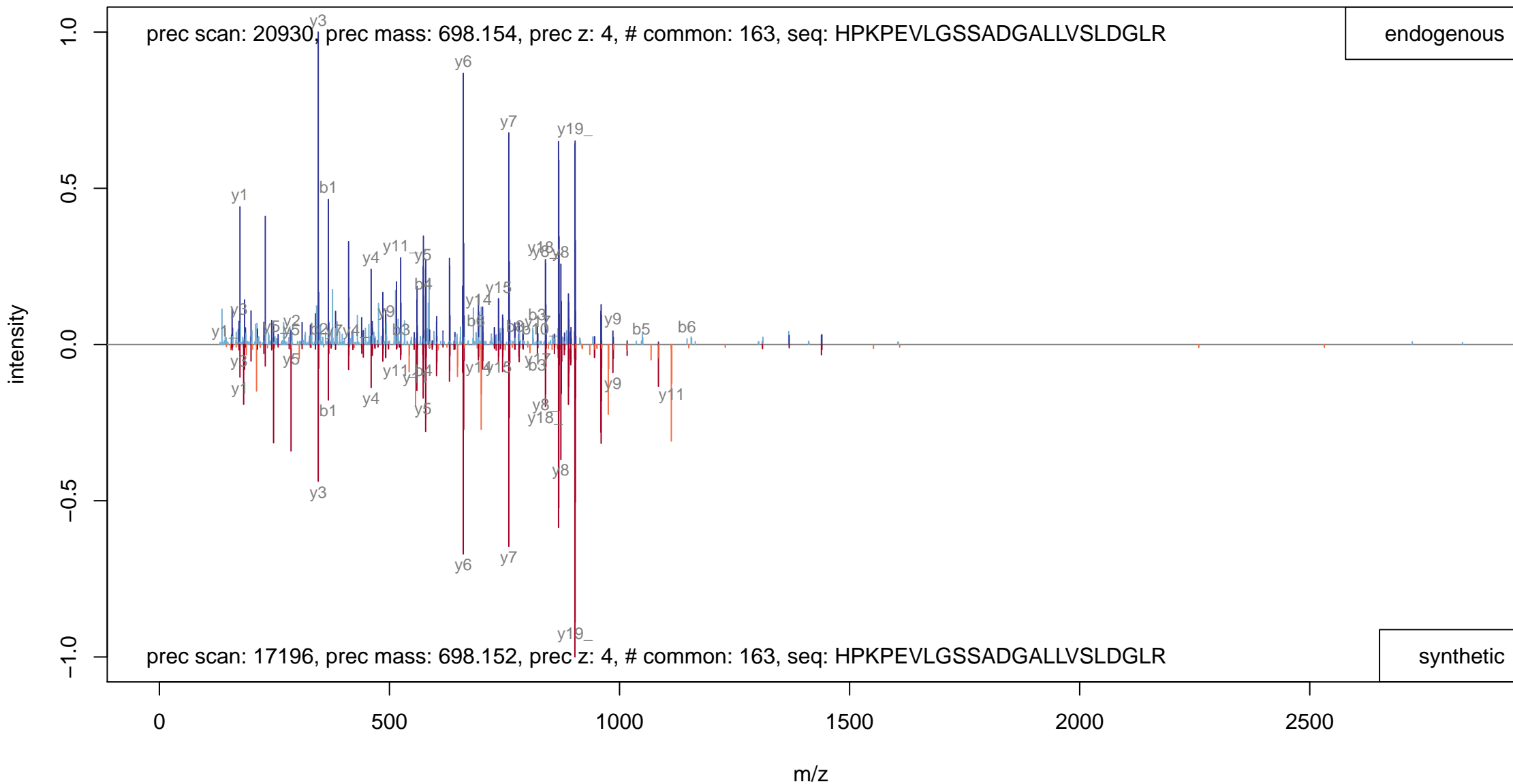

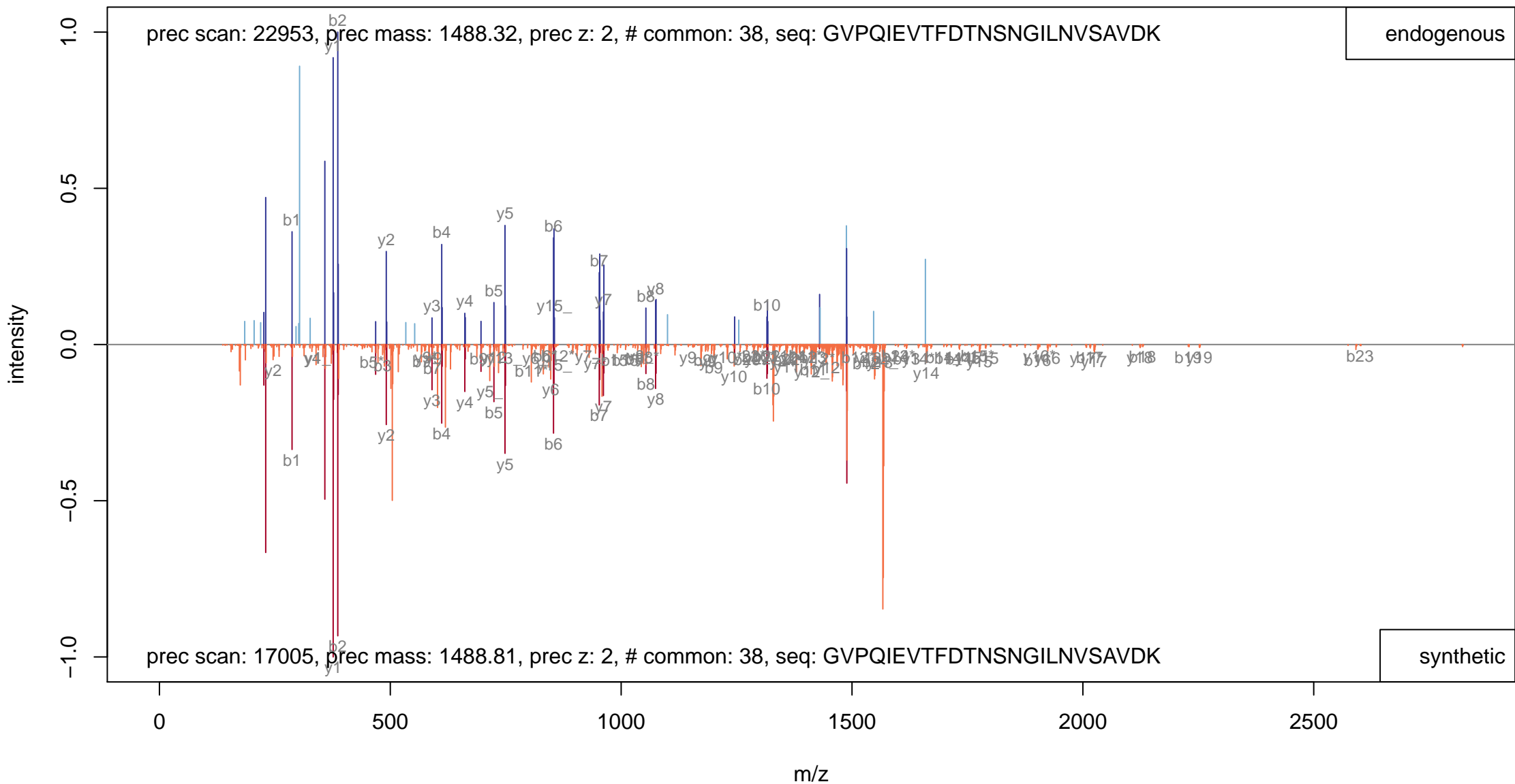

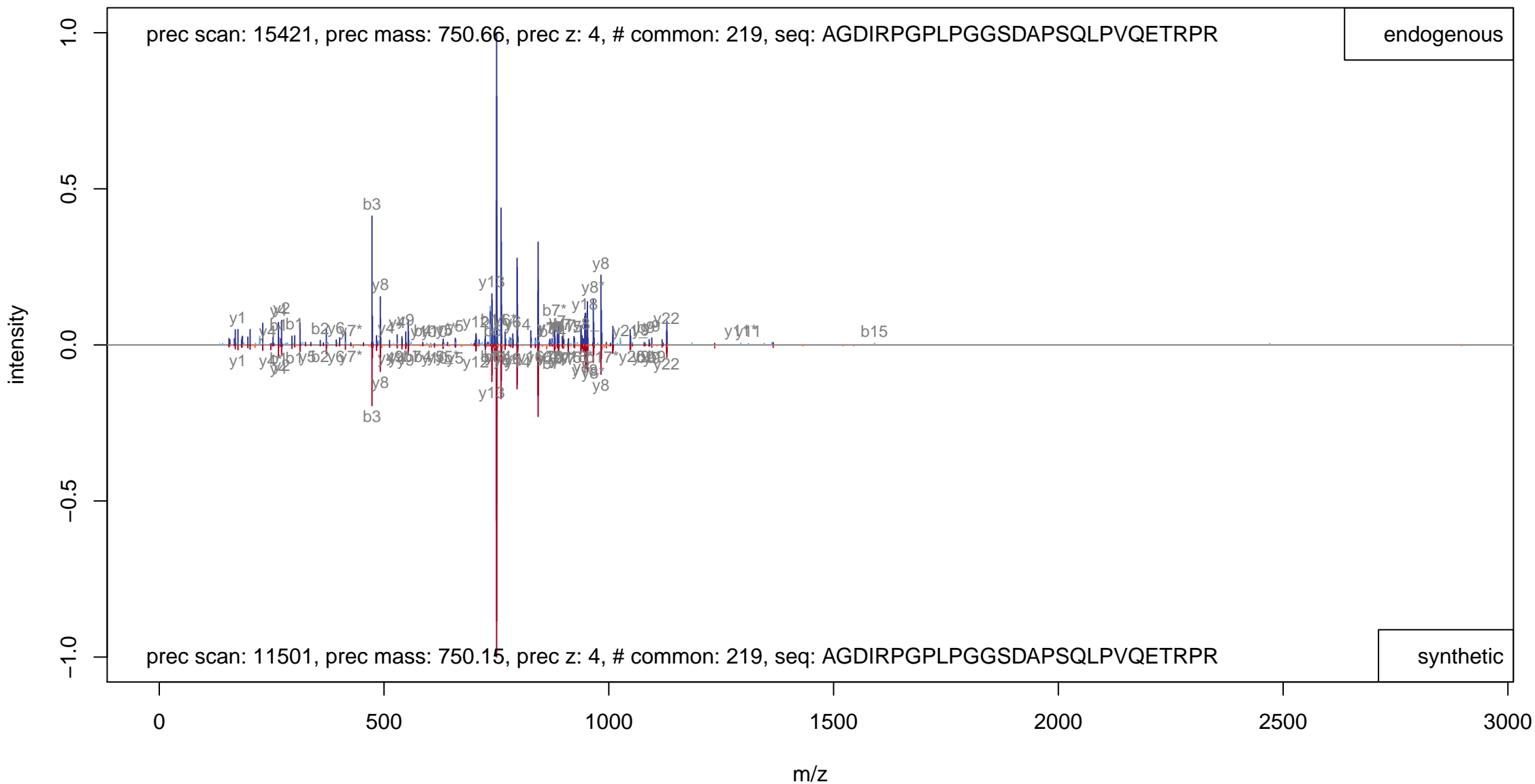

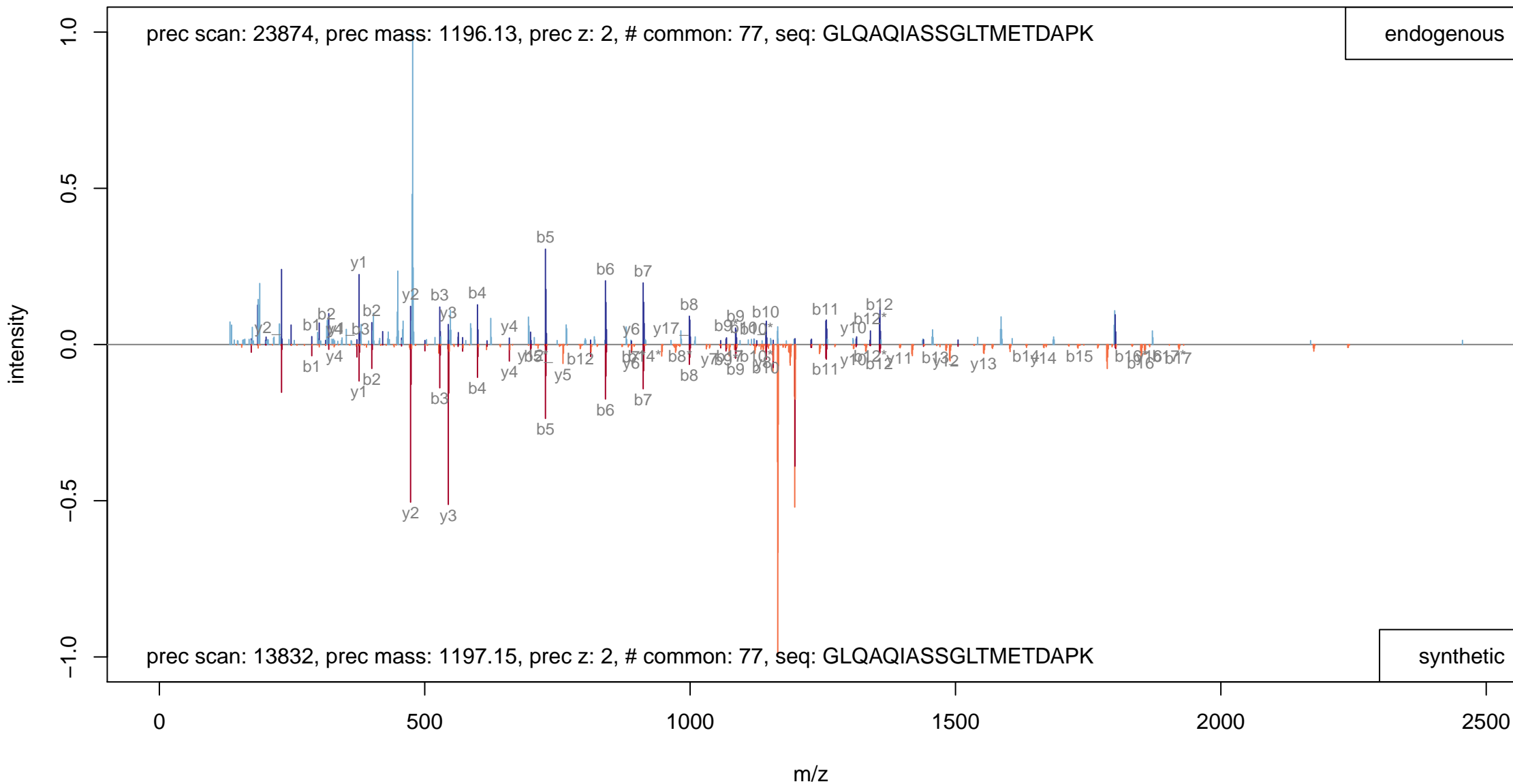

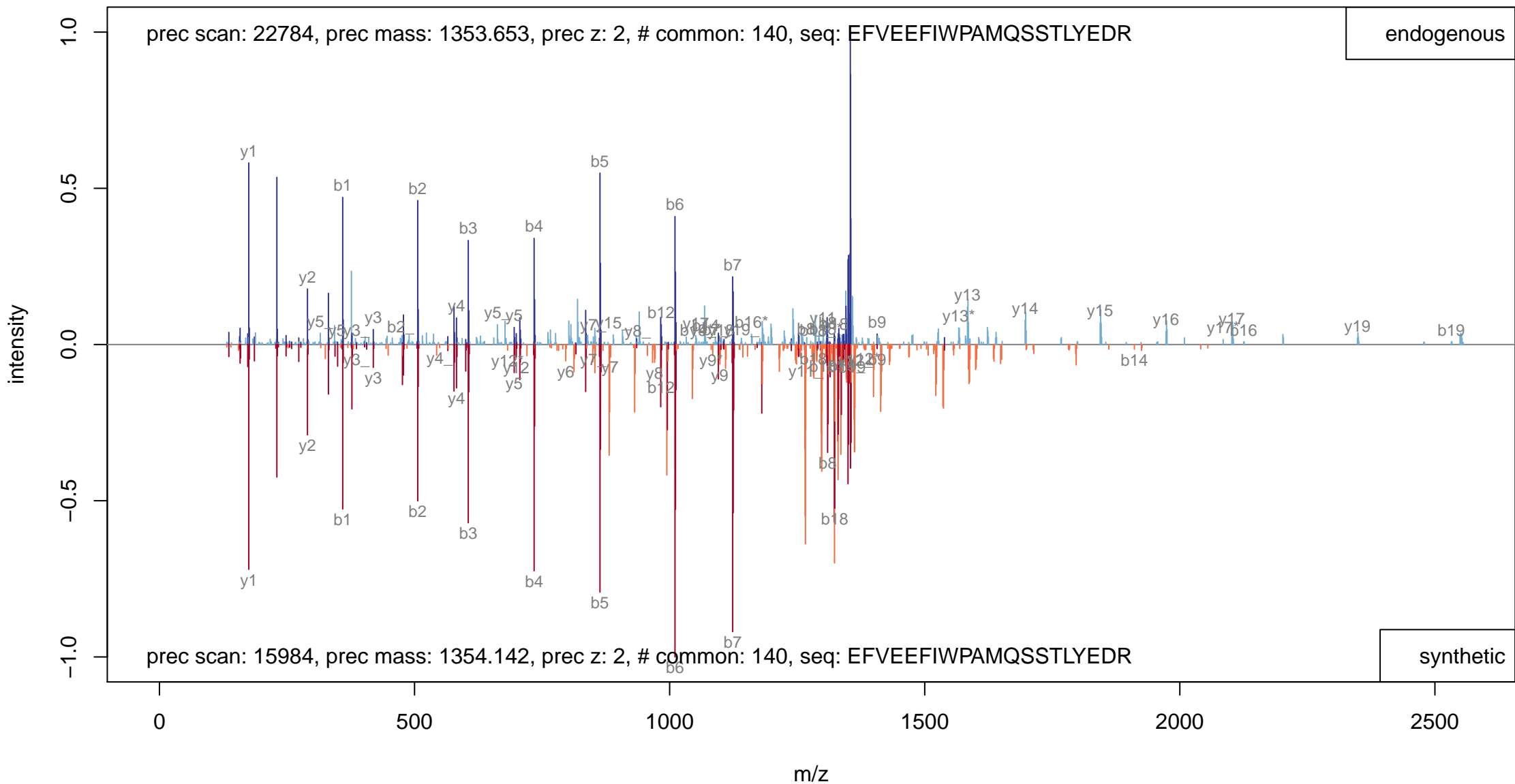

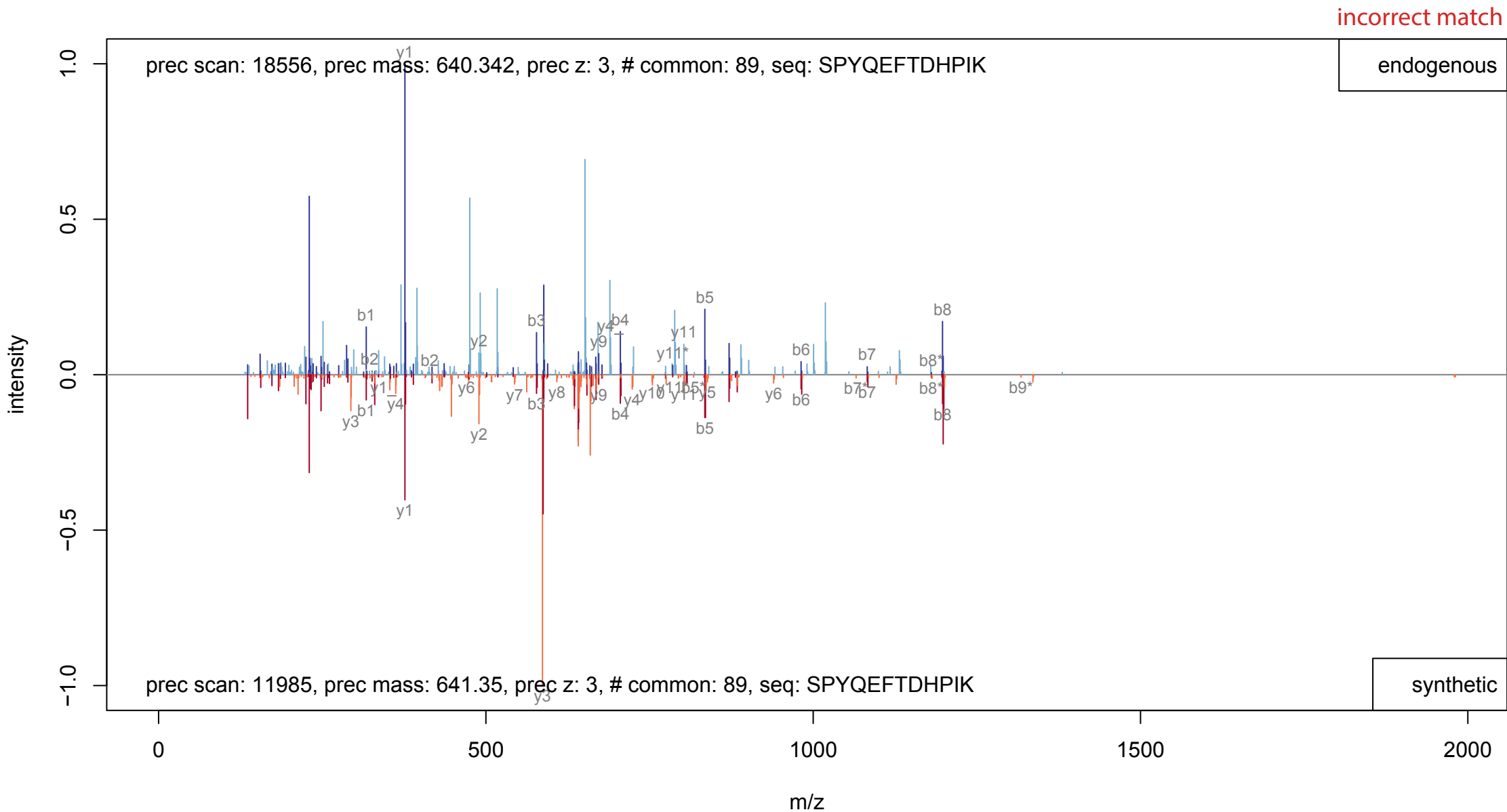

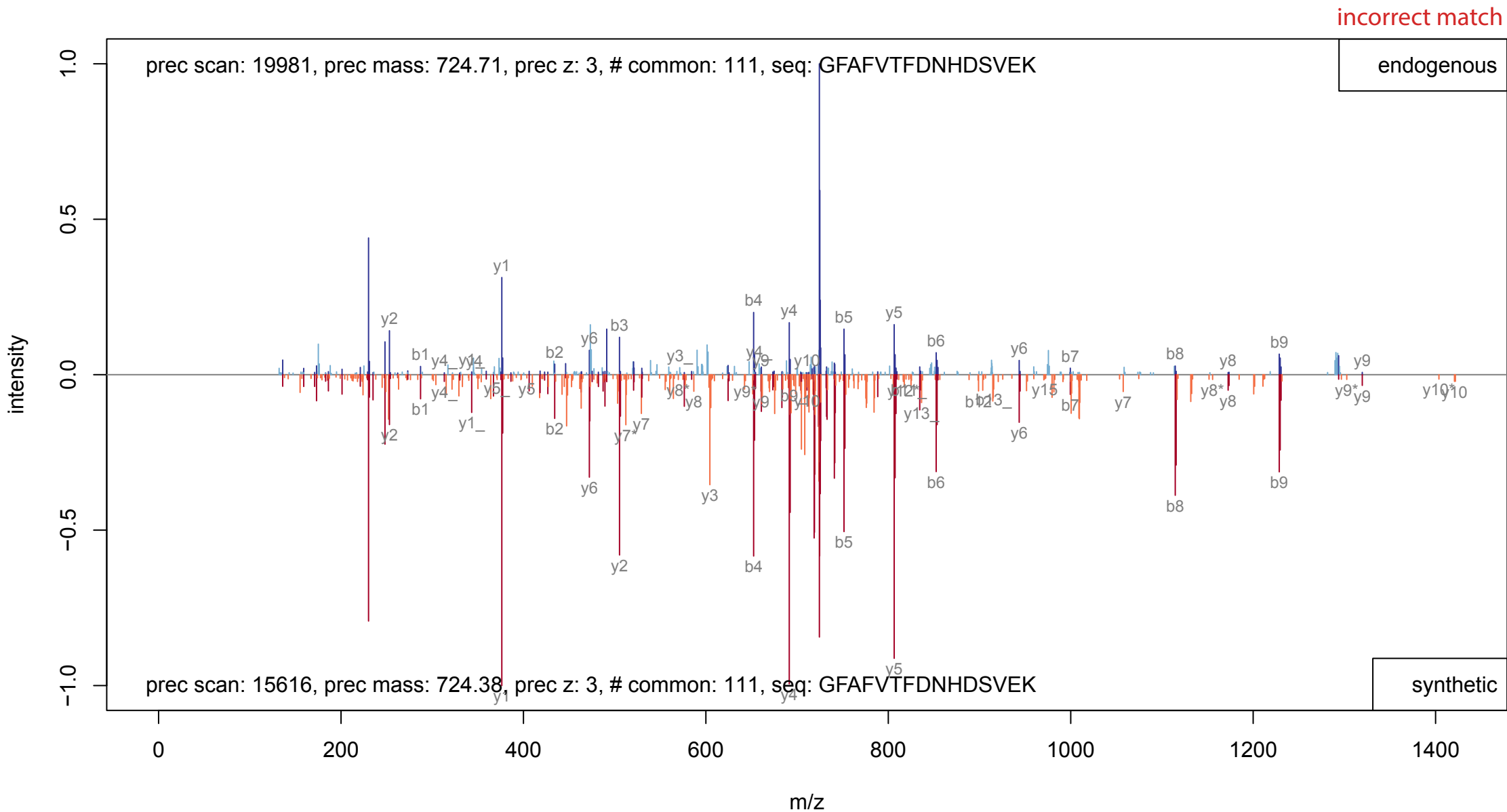

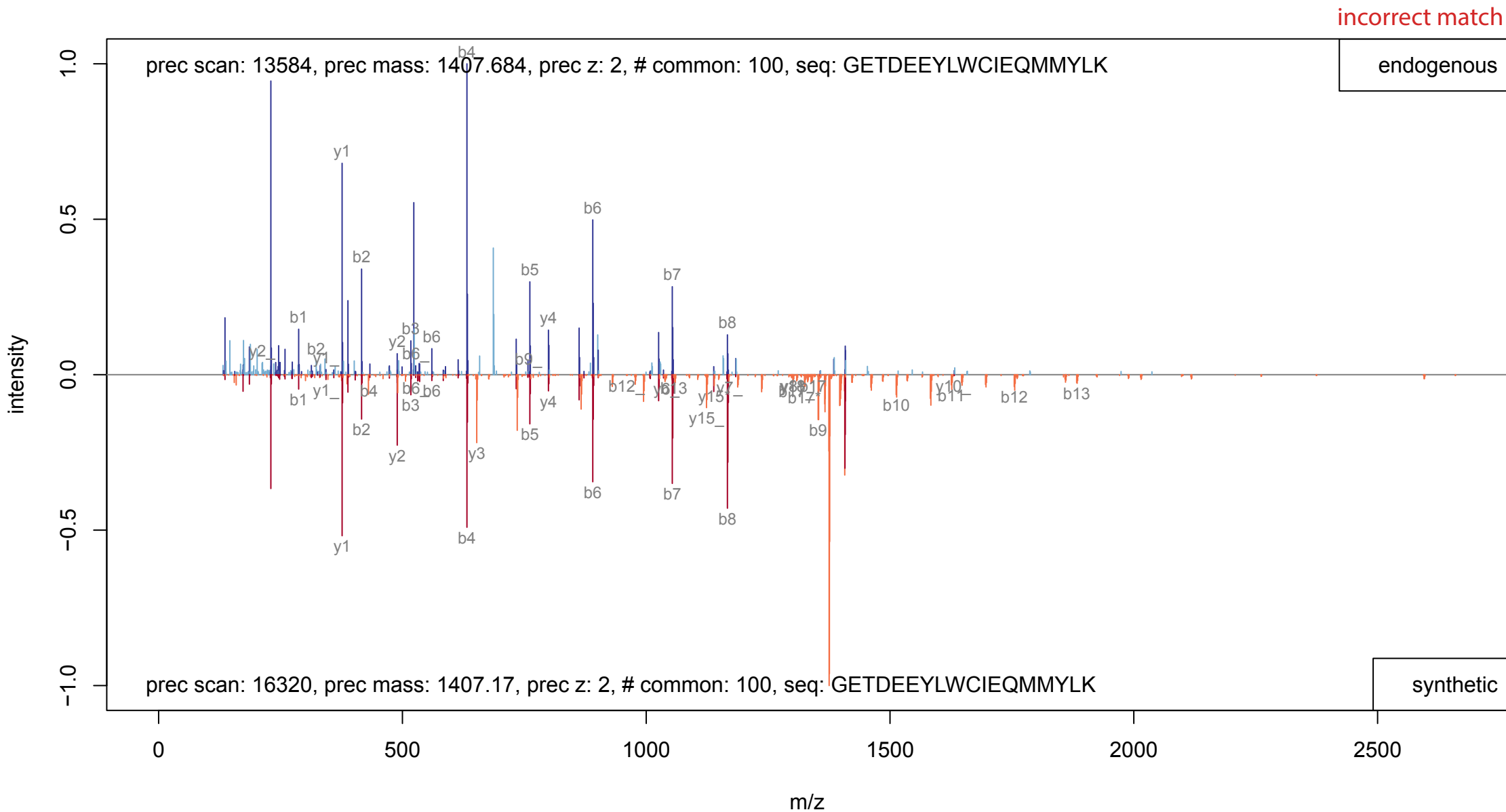

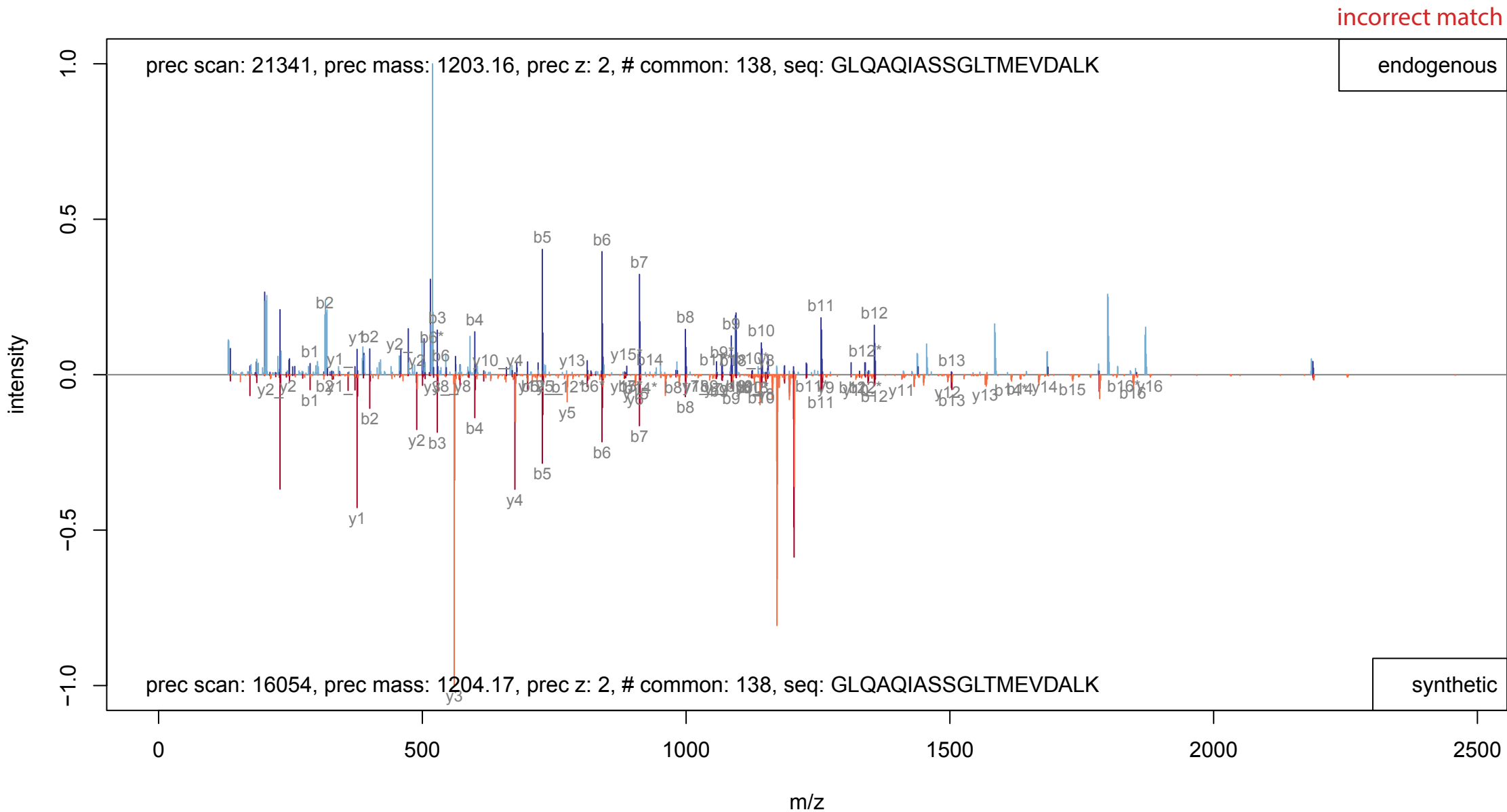

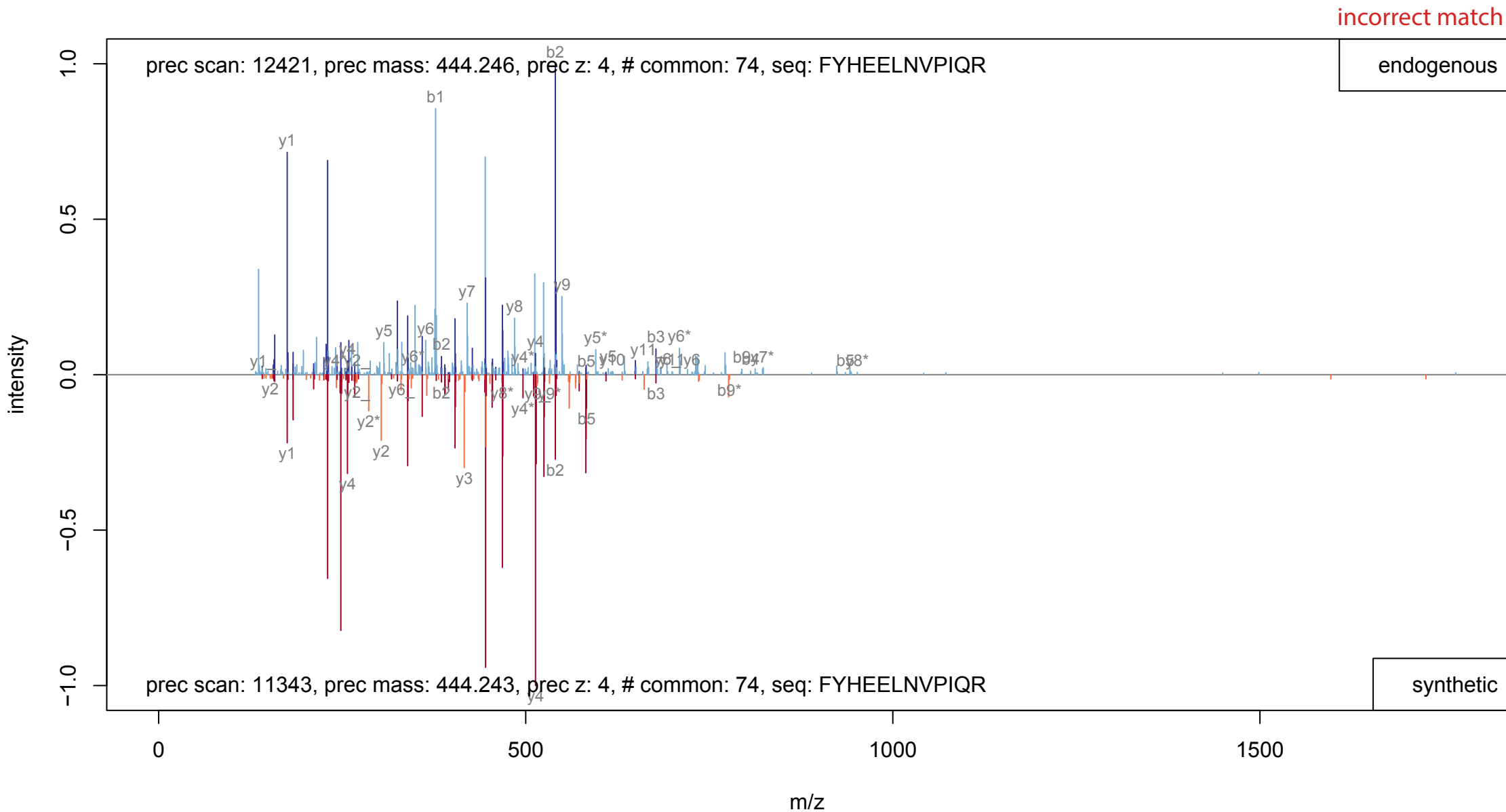

endogenous

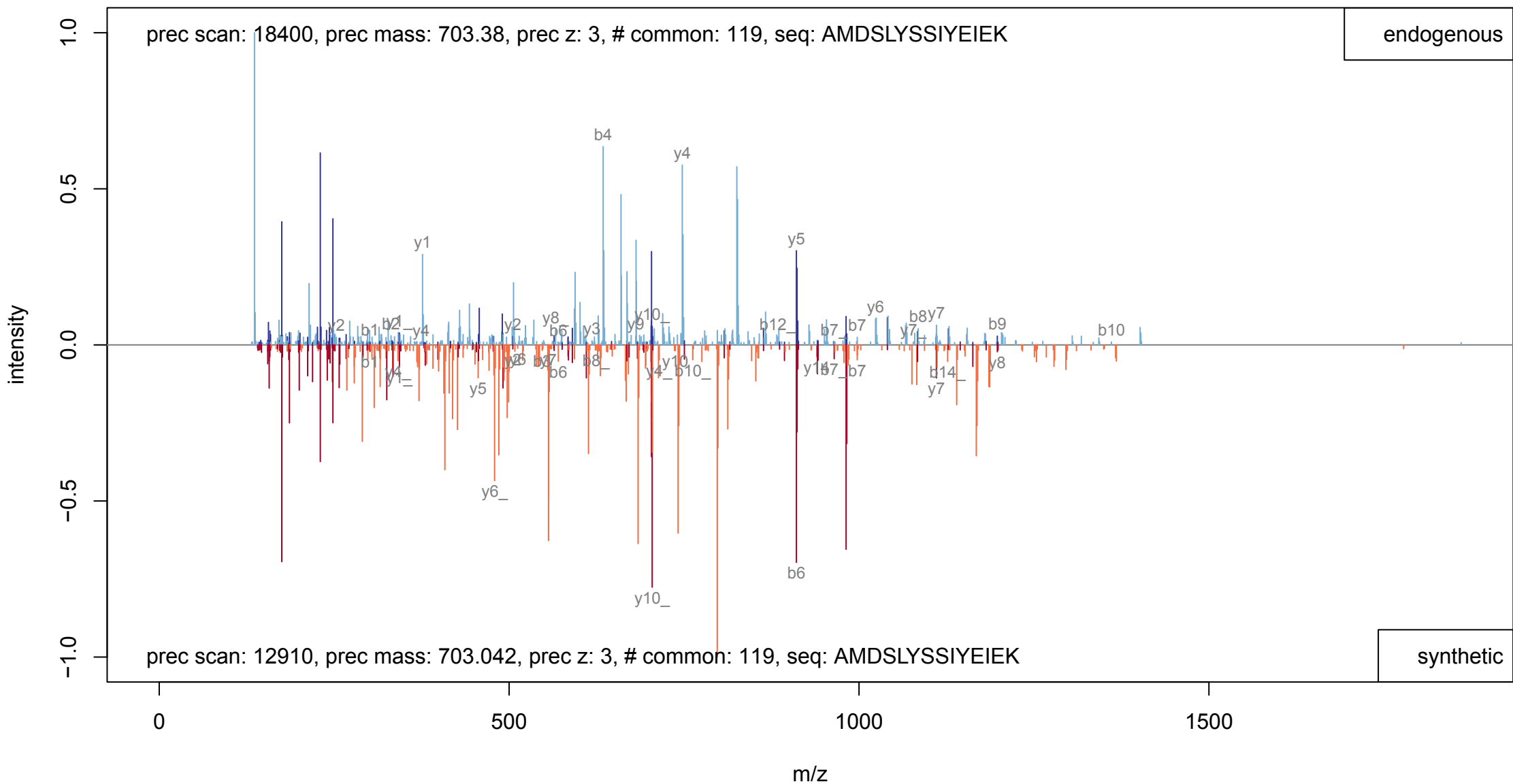

endogenous

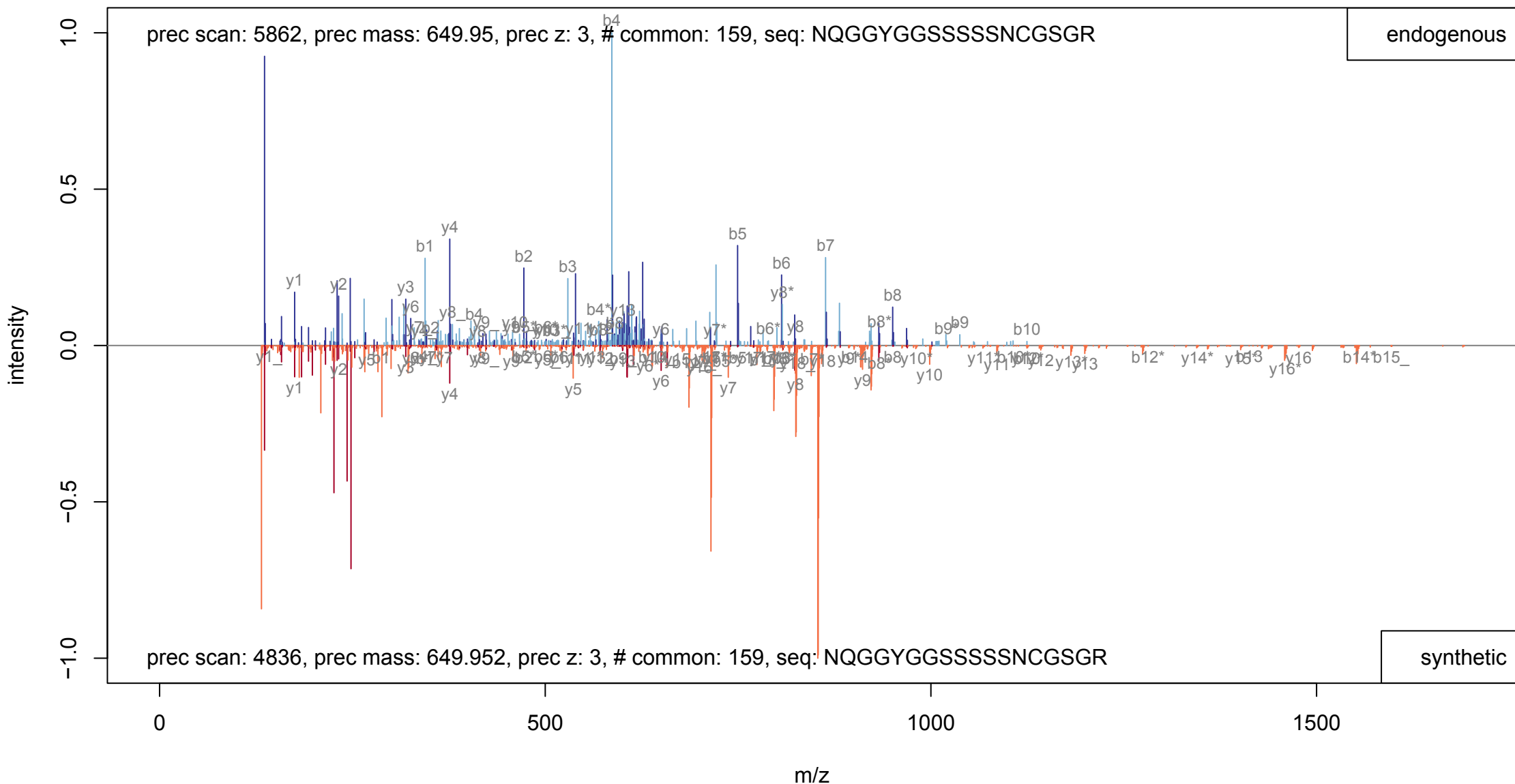

Supplement: Supplementary file 6 — Supplementary Data 3 [file 41467_2018_3311_MOESM6_ESM.pdf]
