# Supplementary material for: Discovery of coding regions in the human genome by integrated proteogenomics analysis workflow
Source: Nat Commun. 2018 Mar 2;9:903. doi: 10.1038/s41467-018-03311-y (PMC5834625; doi:10.1038/s41467-018-03311-y)
Supplement: Supplementary file 9 — Supplementary Data 6 [file 41467_2018_3311_MOESM9_ESM.pdf]

➢ N-terminally extended CDK16; length=613aa

MGSAATPRSD RSVPRAAVST RIQAGANESG GIARSGQAHG RLSGTPPPPQ PPPPPPPPPP QPAAARAQQP WLKTTGTGAC GEQEEKEVAR PHPGPPPQAP
PRRPRGSEVA RAPPPIA MDRMKKIKRQ LSMTLRGGRG IDKTNGAPEQ IGLDESGGGG GSDPGEAPTR AAPGELRSAR GPLSSAPEIV HEDLKMGSDG
ESDQASATSS DEVQSPVRVR MRNHPPRKIS TEDINKRLSL PADIRLPEGY LEKLTLNSPI FDKPLSRRLR RVSLSEIGFG KLETYIKLDK LGEGTYATVY
KGKSKLTDNL VALKEIRLEH EEGAPCTAIR EVSLLKDLKH ANIVTLHDII HTEKSLTLVF EYLDKDLKQY LDDCGNIINM HNVKLFLFQL LRGLAYCHRQ
KVLHRDLKPQ NLLINERGEL KLADFGLARA KSIPTKTYSN EVVTLWYRPP DILLGSTDYS TQIDMWGVGC IFYEMATGRP LFPGSTVEEQ LHFIFRILGT
PTEETWPGIL SNEEFKTYNY PKYRAEALLS HAPRLDSDGA DLLTKLLQFE GRNRISAEDA MKHPFFLSLG ERIHKLPDTT SIFALKEIQL QKEASLRSSS
MPDSGRPAFR VVDTEF

```
### targetp v1.1 prediction results ##################################
Number of query sequences:  1
Cleavage site predictions included.
Using NON-PLANT networks.

Name                  Len            mTP     SP  other  Loc  RC  TPlen
----------------------------------------------------------------------
Sequence               40          0.756  0.028  0.316   M     3     24
----------------------------------------------------------------------
cutoff                             0.000  0.000  0.000
```

➢ Canonical CDK16 (ENST00000357227.4); length=496aa

MDRMKKIKRQ LSMTLRGGRG IDKTNGAPEQ IGLDESGGGG GSDPGEAPTR AAPGELRSAR GPLSSAPEIV HEDLKMGSDG ESDQASATSS DEVQSPVRVR
MRNHPPRKIS TEDINKRLSL PADIRLPEGY LEKLTLNSPI FDKPLSRRLR RVSLSEIGFG KLETYIKLDK LGEGTYATVY KGKSKLTDNL VALKEIRLEH
EEGAPCTAIR EVSLLKDLKH ANIVTLHDII HTEKSLTLVF EYLDKDLKQY LDDCGNIINM HNVKLFLFQL LRGLAYCHRQ KVLHRDLKPQ NLLINERGEL
KLADFGLARA KSIPTKTYSN EVVTLWYRPP DILLGSTDYS TQIDMWGVGC IFYEMATGRP LFPGSTVEEQ LHFIFRILGT PTEETWPGIL SNEEFKTYNY
PKYRAEALLS HAPRLDSDGA DLLTKLLQFE GRNRISAEDA MKHPFFLSLG ERIHKLPDTT SIFALKEIQL QKEASLRSSS MPDSGRPAFR VVDTEF

```
### targetp v1.1 prediction results ##################################
Number of query sequences:  1
Cleavage site predictions included.
Using NON-PLANT networks.

Name                  Len            mTP     SP  other  Loc  RC  TPlen
----------------------------------------------------------------------
Sequence               40          0.235  0.047  0.802   _     3      -
----------------------------------------------------------------------
cutoff                             0.000  0.000  0.000
```

➢ N-terminally extended NPLOC4; length=651aa

LERRWRRRRE AGAGAEAAAG SARPLGRQAA AARGSSPEAG AAA MAESIIIRVQ SPDGVKRITA TKRETAATFL KKVAKEFGFQ NNGFSVYINR NKTGEITASS
NKSLNLLKIK HGDLLFLFPS SLAGPSSEME TSVPPGFKVF GAPNVVEDEI DQYLSKQDGK IYRSRDPQLC RHGPLGKCVH CVPLEPFDED YLNHLEPPVK
HMSFHAYIRK LTGGADKGKF VALENISCKI KSGCEGHLPW PNGICTKCQP SAITLNRQKY RHVDNIMFEN HTVADRFLDF WRKTGNQHFG YLYGRYTEHK
DIPLGIRAEV AAIYEPPQIG TQNSLELLED PKAEVVDEIA AKLGLRKVGW IFTDLVSEDT RKGTVRYSRN KDTYFLSSEE CITAGDFQNK HPNMCRLSPD
GHFGSKFVTA VATGGPDNQV HFEGYQVSNQ CMALVRDECL LPCKDAPELG YAKESSSEQY VPDVFYKDVD KFGNEITQLA RPLPVEYLII DITTTFPKDP
VYTFSISQNP FPIENRDVLG ETQDFHSLAT YLSQNTSSVF LDTISDFHLL LFLVTNEVMP LQDSISLLLE AVRTRNEELA QTWKRSEQWA TIEQLCSTVG
GQLPGLHEYG AVGGSTHTAT AAMWACQHCT FMNQPGTGHC EMCSLPRT

```
### targetp v1.1 prediction results ##################################
Number of query sequences:  1
Cleavage site predictions included.
Using NON-PLANT networks.

Name                  Len            mTP     SP  other  Loc  RC  TPlen
----------------------------------------------------------------------
Sequence               43          0.879  0.023  0.176   M    2     34
----------------------------------------------------------------------
cutoff                             0.000  0.000  0.000
```

➢ Canonical NPLOC4; length=608aa

MAESIIIRVQ SPDGVKRITA TKRETAATFL KKVAKEFGFQ NNGFSVYINR NKTGEITASS NKSLNLLKIK HGDLLFLFPS SLAGPSSEME TSVPPGFKVF
GAPNVVEDEI DQYLSKQDGK IYRSRDPQLC RHGPLGKCVH CVPLEPFDED YLNHLEPPVK HMSFHAYIRK LTGGADKGKF VALENISCKI KSGCEGHLPW
PNGICTKCQP SAITLNRQKY RHVDNIMFEN HTVADRFLDF WRKTGNQHFG YLYGRYTEHK DIPLGIRAEV AAIYEPPQIG TQNSLELLED PKAEVVDEIA
AKLGLRKVGW IFTDLVSEDT RKGTVRYSRN KDTYFLSSEE CITAGDFQNK HPNMCRLSPD GHFGSKFVTA VATGGPDNQV HFEGYQVSNQ CMALVRDECL
LPCKDAPELG YAKESSSEQY VPDVFYKDVD KFGNEITQLA RPLPVEYLII DITTTFPKDP VYTFSISQNP FPIENRDVLG ETQDFHSLAT YLSQNTSSVF
LDTISDFHLL LFLVTNEVMP LQDSISLLLE AVRTRNEELA QTWKRSEQWA TIEQLCSTVG GQLPGLHEYG AVGGSTHTAT AAMWACQHCT FMNQPGTGHC
EMCSLPRT

```
### targetp v1.1 prediction results ##################################
Number of query sequences:  1
Cleavage site predictions included.
Using NON-PLANT networks.

Name                  Len            mTP     SP  other  Loc  RC  TPlen
----------------------------------------------------------------------
Sequence               50          0.262  0.039  0.806   _    3      -
----------------------------------------------------------------------
cutoff                             0.000  0.000  0.000
```

➢ N-terminally extended THOP1; length=726aa

```
VAAVAAVGPR QAASVAEVAG RVAGGRREGA AGADPPA MKPPAACAGD MADAASPCSV VNDLRWDLSA QQIEERTREL IEQTKRVYDQ VGTQEFEDVS YESTLKALAD VEVTYTVQRN
ILDFPQHVSP SKDIRTASTE ADKKLSEFDV EMSMREDVYQ RIVWLQEKVQ KDSLRPEAAR YLERLIKLGR RNGLHLPRET QENIKRIKKK LSLLCIDFNK NLNEDTTFLP
FTLQELGGLP EDFLNSLEKM EDGKLKVTLK YPHYFPLLKK CHVPETRRKV EEAFNCRCKE ENCAILKELV TLRAQKSRLL GFHTHADYVL EMNMAKTSQT VATFLDELAQ
KLKPLGEQER AVILELKRAE CERRGLPFDG RIRAWDMRYY MNQVEETRYC VDQNLLKEYF PVQVVTHGLL GIYQELLGLA FHHEEGASAW HEDVRLYTAR DAASGEVVGK
FYLDLYPREG KYGHAACFGL QPGCLRQDGS RQIAIAAMVA NFTKPTADAP SLLQHDEVET YFHEFGHVMH QLCSQAEFAM FSGTHVERDF VEAPSQMLEN WVWEQEPLLR
MSRHYRTGSA VPRELLEKLI ESRQANTGLF NLRQIVLAKV DQALHTQTDA DPAEEYARLC QEILGVPATP GTNMPATFGH LAGGYDAQYY GYLWSEVYSM DMFHTRFKQE
GVLNSKVGMD YRSCILRPGG SEDASAMLRR FLGRDPKQDA FLLSKGLQVG GCEPEPQVC
```

```
### targetp v1.1 prediction results ##################################
Number of query sequences:  1
Cleavage site predictions included.
Using NON-PLANT networks.

Name                  Len            mTP     SP  other  Loc  RC  TPlen
----------------------------------------------------------------------
Sequence               40          0.527  0.059  0.478   M    5     11
----------------------------------------------------------------------
cutoff                             0.000  0.000  0.000
```

➢ Canonical THOP1; length=689aa

```
MKPPAACAGD MADAASPCSV VNDLRWDLSA QQIEERTREL IEQTKRVYDQ VGTQEFEDVS YESTLKALAD VEVTYTVQRN ILDFPQHVSP SKDIRTASTE ADKKLSEFDV
EMSMREDVYQ RIVWLQEKVQ KDSLRPEAAR YLERLIKLGR RNGLHLPRET QENIKRIKKK LSLLCIDFNK NLNEDTTFLP FTLQELGGLP EDFLNSLEKM EDGKLKVTLK
YPHYFPLLKK CHVPETRRKV EEAFNCRCKE ENCAILKELV TLRAQKSRLL GFHTHADYVL EMNMAKTSQT VATFLDELAQ KLKPLGEQER AVILELKRAE CERRGLPFDG
RIRAWDMRYY MNQVEETRYC VDQNLLKEYF PVQVVTHGLL GIYQELLGLA FHHEEGASAW HEDVRLYTAR DAASGEVVGK FYLDLYPREG KYGHAACFGL QPGCLRQDGS
RQIAIAAMVA NFTKPTADAP SLLQHDEVET YFHEFGHVMH QLCSQAEFAM FSGTHVERDF VEAPSQMLEN WVWEQEPLLR MSRHYRTGSA VPRELLEKLI ESRQANTGLF
NLRQIVLAKV DQALHTQTDA DPAEEYARLC QEILGVPATP GTNMPATFGH LAGGYDAQYY GYLWSEVYSM DMFHTRFKQE GVLNSKVGMD YRSCILRPGG SEDASAMLRR
FLGRDPKQDA FLLSKGLQVG GCEPEPQVC
```

```
### targetp v1.1 prediction results ##################################
Number of query sequences:  1
Cleavage site predictions included.
Using NON-PLANT networks.

Name                  Len            mTP     SP  other  Loc  RC  TPlen
----------------------------------------------------------------------
Sequence               40          0.089  0.084  0.886   _    2      -
----------------------------------------------------------------------
cutoff                             0.000  0.000  0.000
```

DESCRIPTION

The output is a table in plain text (see the example below). For each input sequence one table row is output. The columns are as follows:

Name	Sequence name truncated to 20 characters
Len	Sequence length
cTP, mTP, SP, other	Final NN scores on which the final prediction is based (Loc, see below). Note that the scores are not really probabilities, and they do not necessarily add to one. However, the location with the highest score is the most likely according to TargetP, and the relationship between the scores (the reliability class, see below) may be an indication of how certain the prediction is.
Loc	Prediction of localization, based on the scores above; the possible values are:<br>C Chloroplast, i.e. the sequence contains cTP, a chloroplast transit peptide;<br>M Mitochondrion, i.e. the sequence contains mTP, a mitochondrial targeting peptide;<br>S Secretory pathway, i.e. the sequence contains SP, a signal peptide;<br>_ Any other location;<br>* "don't know"; indicates that cutoff restrictions were set (see instructions) and the winning network output score was below the requested cutoff for that category.
RC	Reliability class, from 1 to 5, where 1 indicates the strongest prediction. RC is a measure of the size of the difference ('diff') between the highest (winning) and the second highest output scores. There are 5 reliability classes, defined as follows:<br>1 : diff > 0.800<br>2 : 0.800 > diff > 0.600<br>3 : 0.600 > diff > 0.400<br>4 : 0.400 > diff > 0.200<br>5 : 0.200 > diff<br>Thus, the lower the value of RC the safer the prediction.
TPlen	Predicted presequence length; it appears only when TargetP was asked to perform cleavage site predictions (see instructions).
