# Supplementary material for: Discovery of coding regions in the human genome by integrated proteogenomics analysis workflow
Source: Nat Commun. 2018 Mar 2;9:903. doi: 10.1038/s41467-018-03311-y (PMC5834625; doi:10.1038/s41467-018-03311-y)
Supplement: Supplementary file 10 — Supplementary Data 7 [file 41467_2018_3311_MOESM10_ESM.pdf]

40 novel protein coding loci with multiple peptides that are linked to in-frame initiation start codons

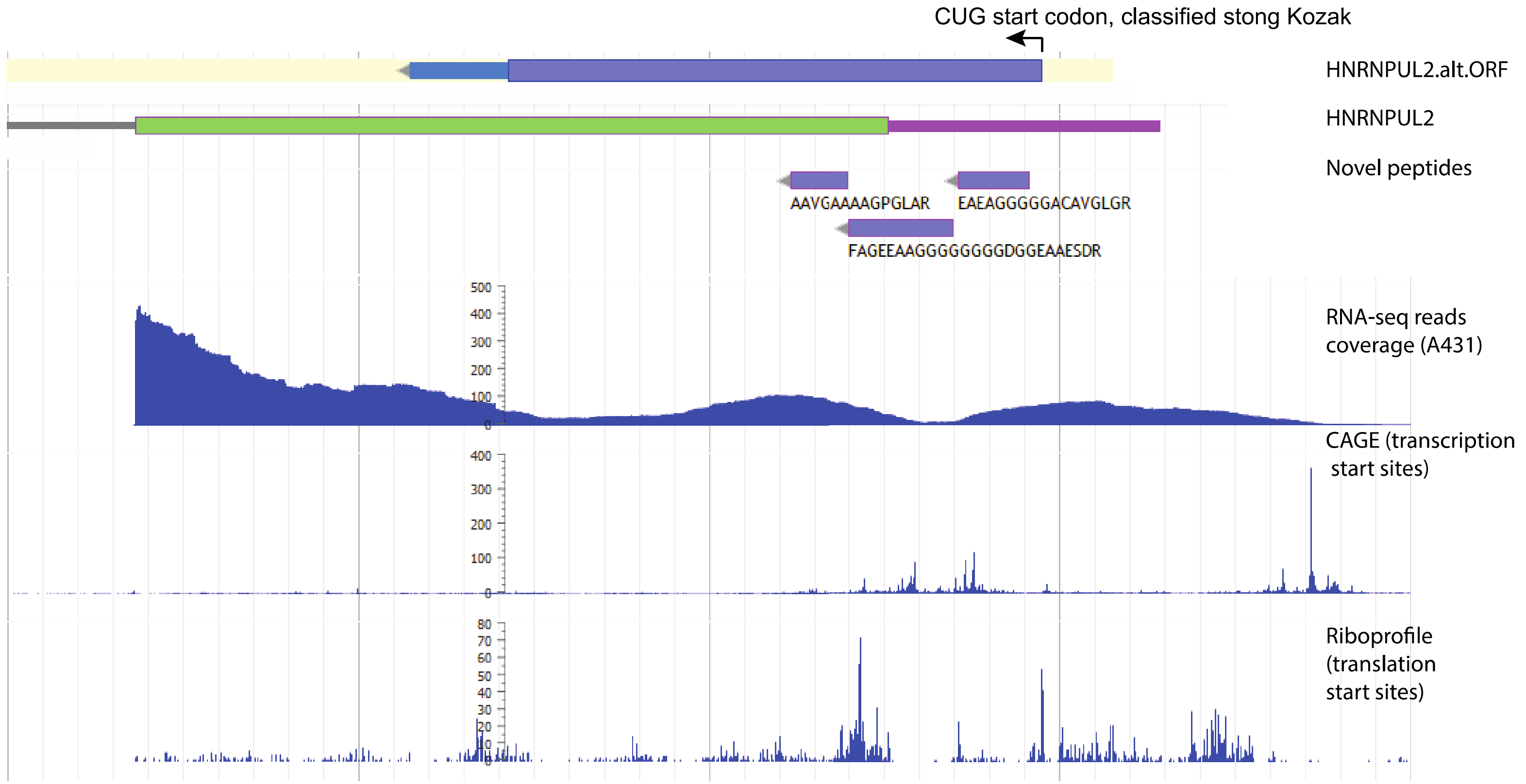

CUG start codon, classified stong Kozak
HNRNPUL2.alt.ORF
HNRNPUL2
Novel peptides
AAVGAAAAGPGLAR
EAEAGGGGGACAVGLGR
FAGEEAAGGGGGGGGDGGEAAESDR
RNA-seq reads coverage (A431)
CAGE (transcription start sites)
Riboprofile (translation start sites)

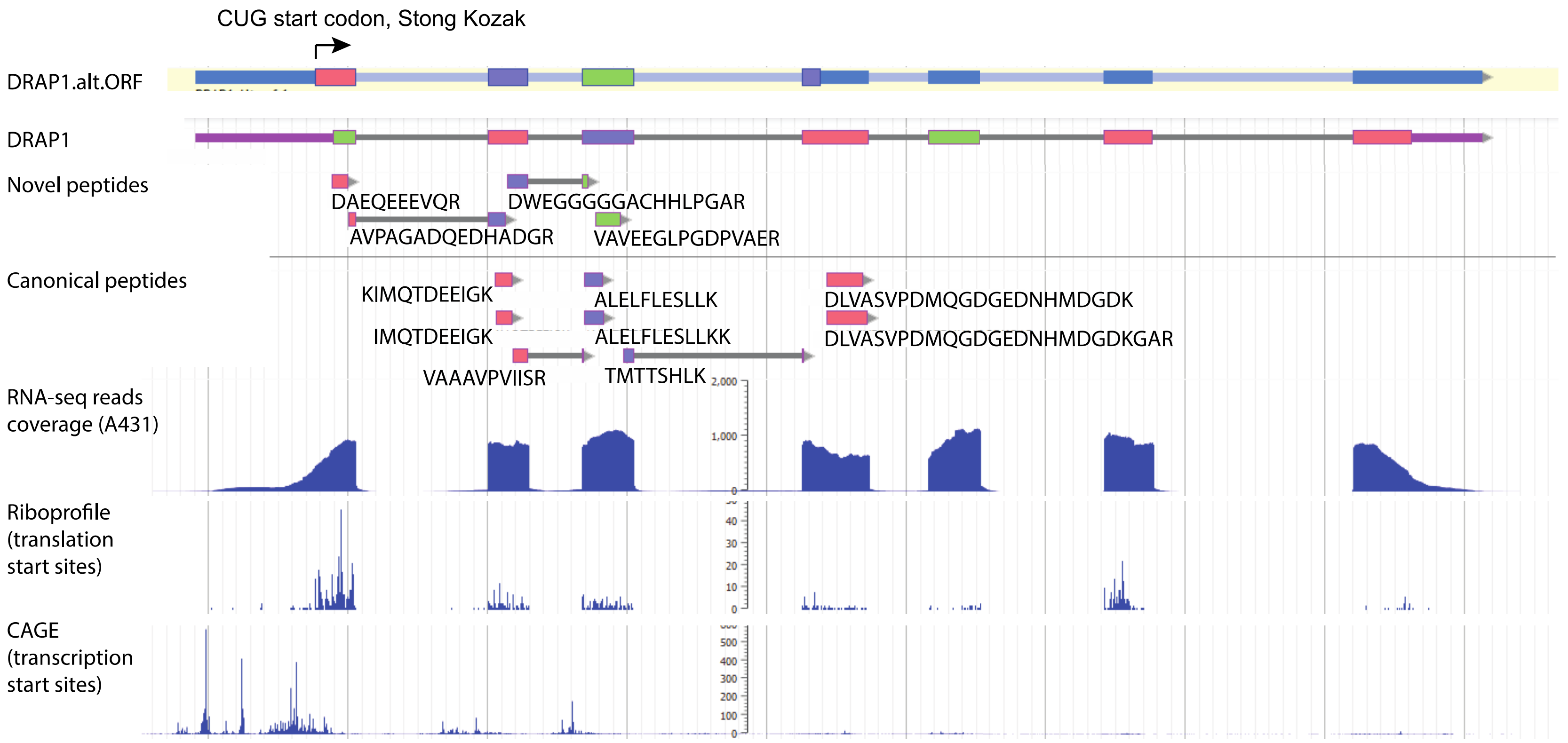

CUG start codon, Stong Kozak
DRAP1.alt.ORF
DRAP1
Novel peptides
DAEQEEEVQR
DWEGGGGGACHHLPGAR
AVPAGADQEDHADGR
VAVEEGLPGDPVAER
Canonical peptides
KIMQTDEEIGK
ALELFLESLLK
DLVASVPDMQGDGEDNHMDGDK
IMQTDEEIGK
ALELFLESLLKK
DLVASVPDMQGDGEDNHMDGDKGAR
VAAAVPVIISR
TMTTSHLK
RNA-seq reads coverage (A431)
2,000
1,000
0
Riboprofile (translation start sites)
40
30
20
10
0
CAGE (transcription start sites)
500
400
300
200
100
0

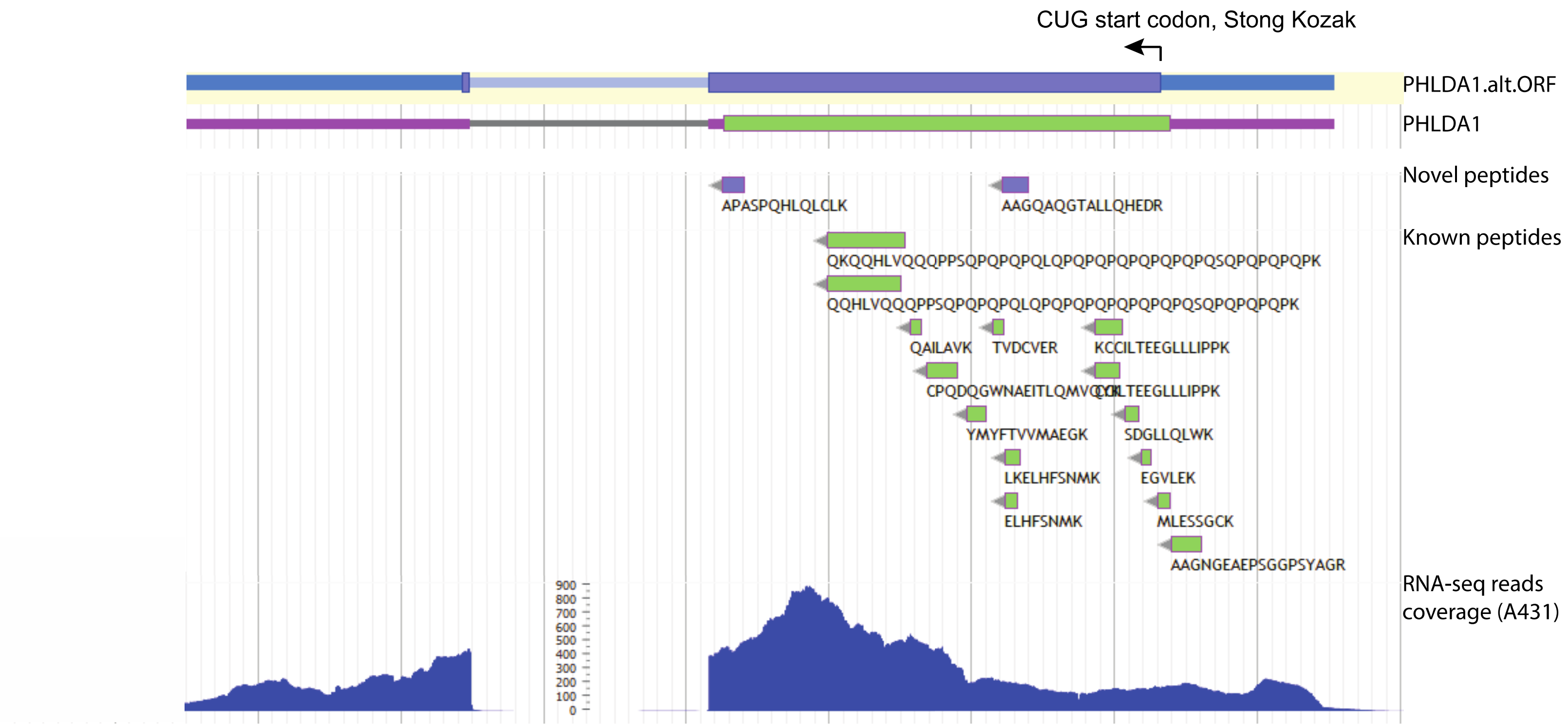
CUG start codon, Stong Kozak
PHLDA1.alt.ORF
PHLDA1
Novel peptides
APASPQHLQLCLK
AAGQAQGTALLQHEDR
Known peptides
QKQQHLVQQQPPSQPQPQPQLQPQPQPQPQPQPQSQPQPQPQPK
QQHLVQQQPPSQPQPQPQLQPQPQPQPQPQPQSQPQPQPQPK
QAILAVK
TVDCVER
KCCILTEEGLLLIPPK
CPQDQGWNAEITLQMVQCK
CCILTEEGLLLIPPK
YMYFTVVMAEGK
SDGLLQLWK
LKELHFSNMK
EGVLEK
ELHFSNMK
MLESSGCK
AAGNGEAEPSGGPSYAGR
RNA-seq reads coverage (A431)
900
800
700
600
500
400
300
200
100
0

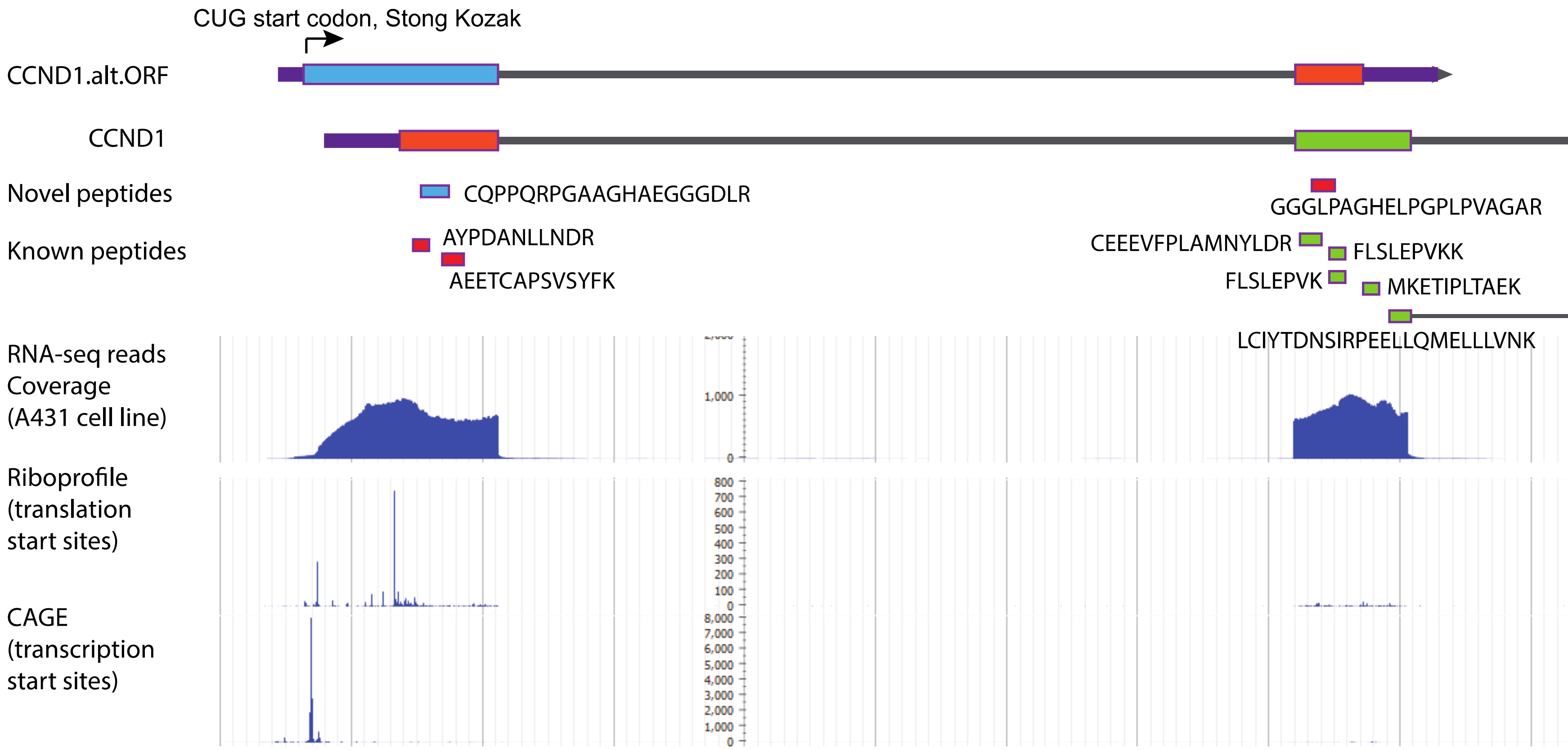
CUG start codon, Stong Kozak
CCND1.alt.ORF
CCND1
Novel peptides
CQPPQRPGAAGHAEGGGDLR
GGGLPAGHELPGPLPVAGAR
Known peptides
AYPDANLLNDR
AEETCAPSVSYFK
CEEEVFPLAMNYLDR
FLSLEPVKK
FLSLEPVK
MKETIPLTAEK
LCIYTDNSIRPEELLQMELLLVNK
RNA-seq reads
Coverage
(A431 cell line)
1,000
0
Riboprofile
(translation
start sites)
800
700
600
500
400
300
200
100
0
CAGE
(transcription
start sites)
8,000
7,000
6,000
5,000
4,000
3,000
2,000
1,000
0

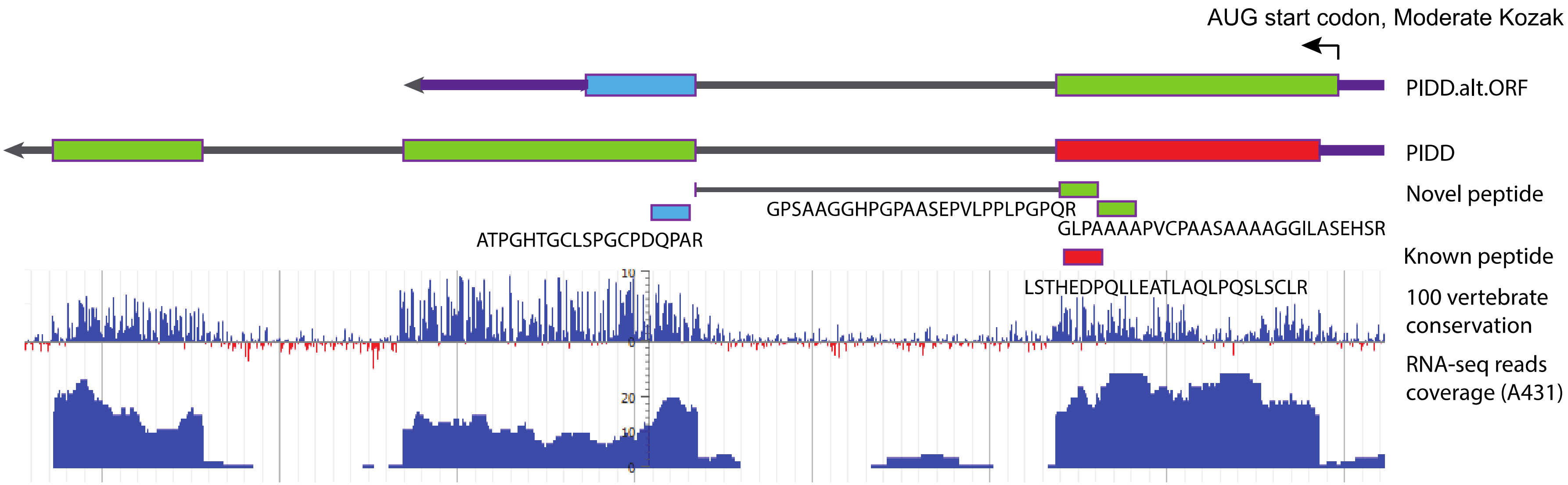

AUG start codon, Moderate Kozak
PIDD.alt.ORF
PIDD
Novel peptide
GPSAAGGHPGPAASEPVLPPLPGPQR
GLPAAAAPVCPAASAAAAGGILASEHSR
ATPGHTGCLSPGCPDQPAR
Known peptide
LSTHEDPQLLEATLAQLPQSLSCLR
100 vertebrate conservation
RNA-seq reads coverage (A431)
10
0
20
10
0

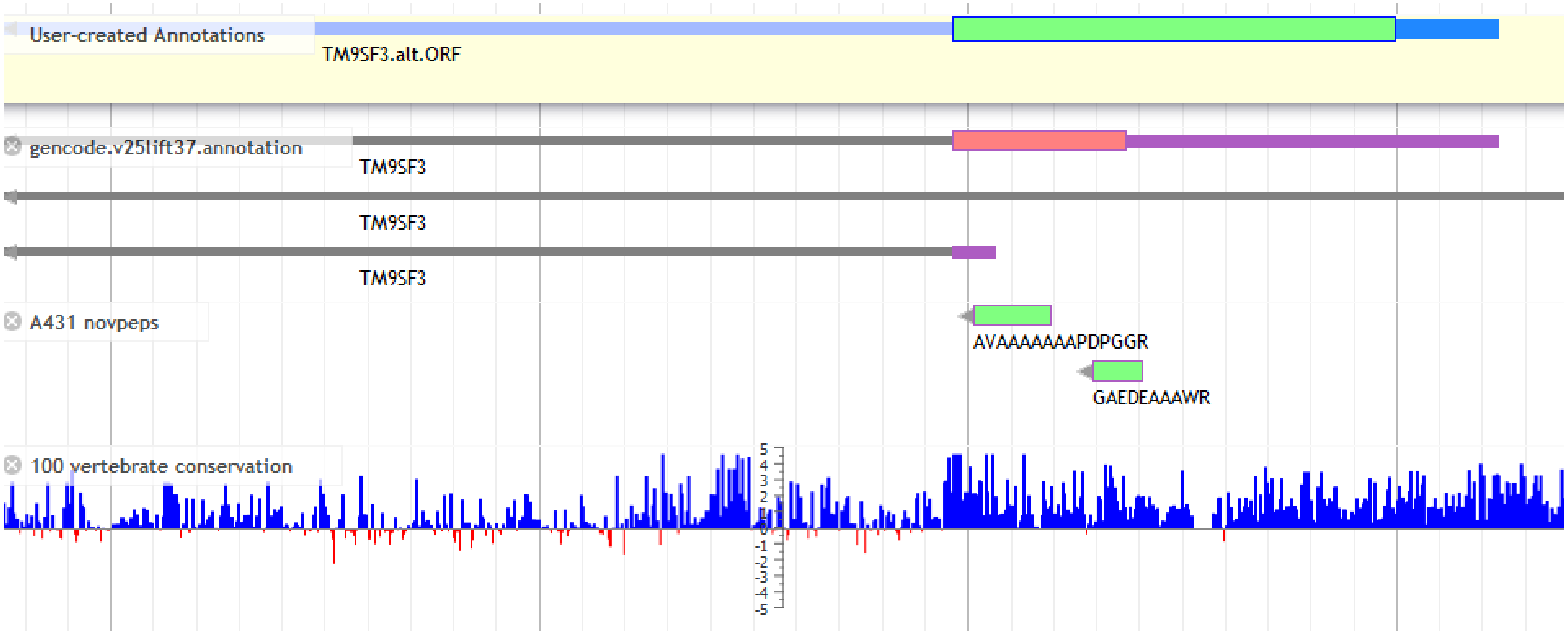
User-created Annotations
TM9SF3.alt.ORF
gencode.v25lift37.annotation
TM9SF3
TM9SF3
TM9SF3
A431 novpeps
AVAAAAAAAPDPGGR
GAEDEAAAWR
100 vertebrate conservation

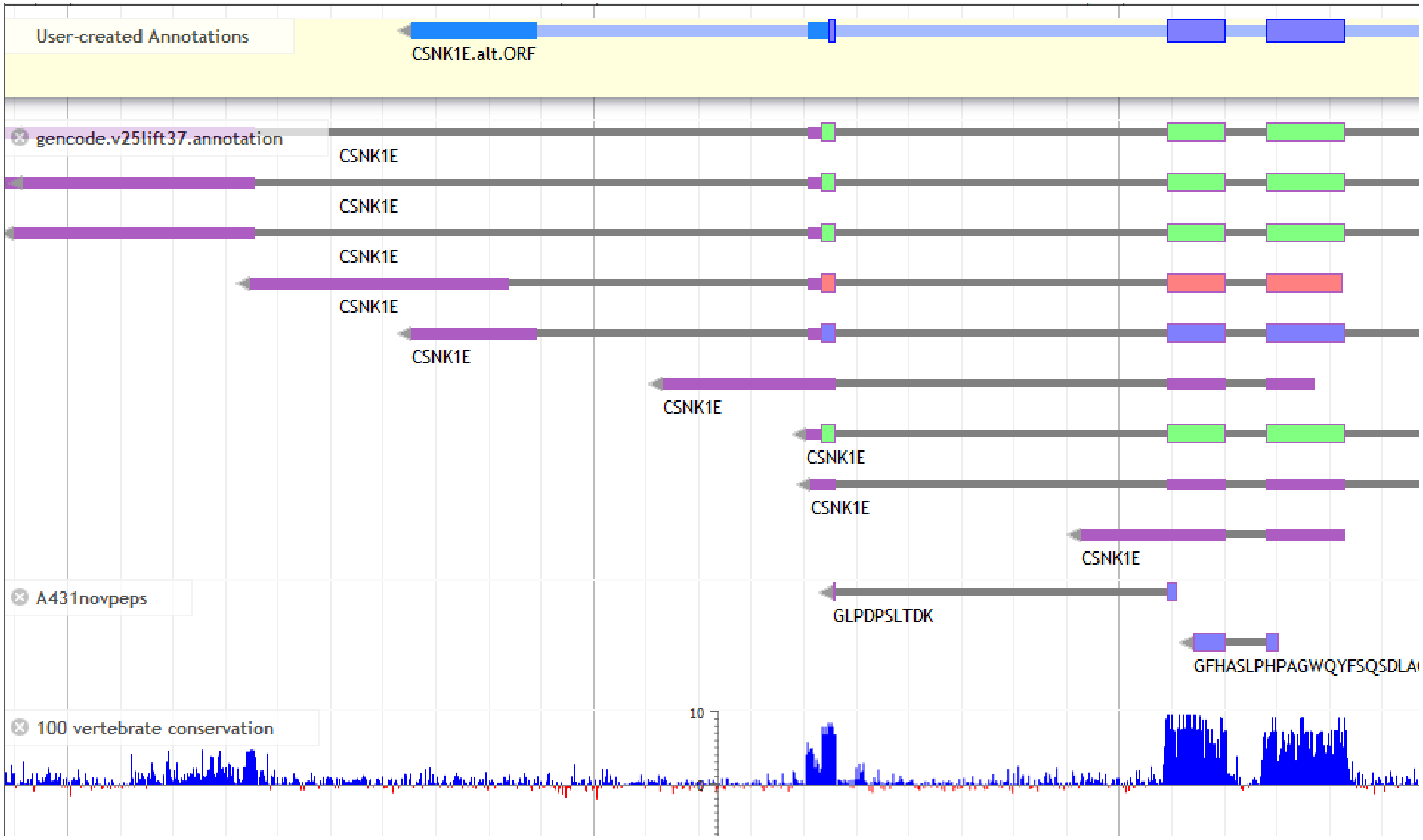

User-created Annotations
CSNK1E.alt.ORF
gencode.v25lift37.annotation
CSNK1E
CSNK1E
CSNK1E
CSNK1E
CSNK1E
CSNK1E
CSNK1E
CSNK1E
CSNK1E
A431novpeps
GLPDPSLTDK
100 vertebrate conservation
10
0

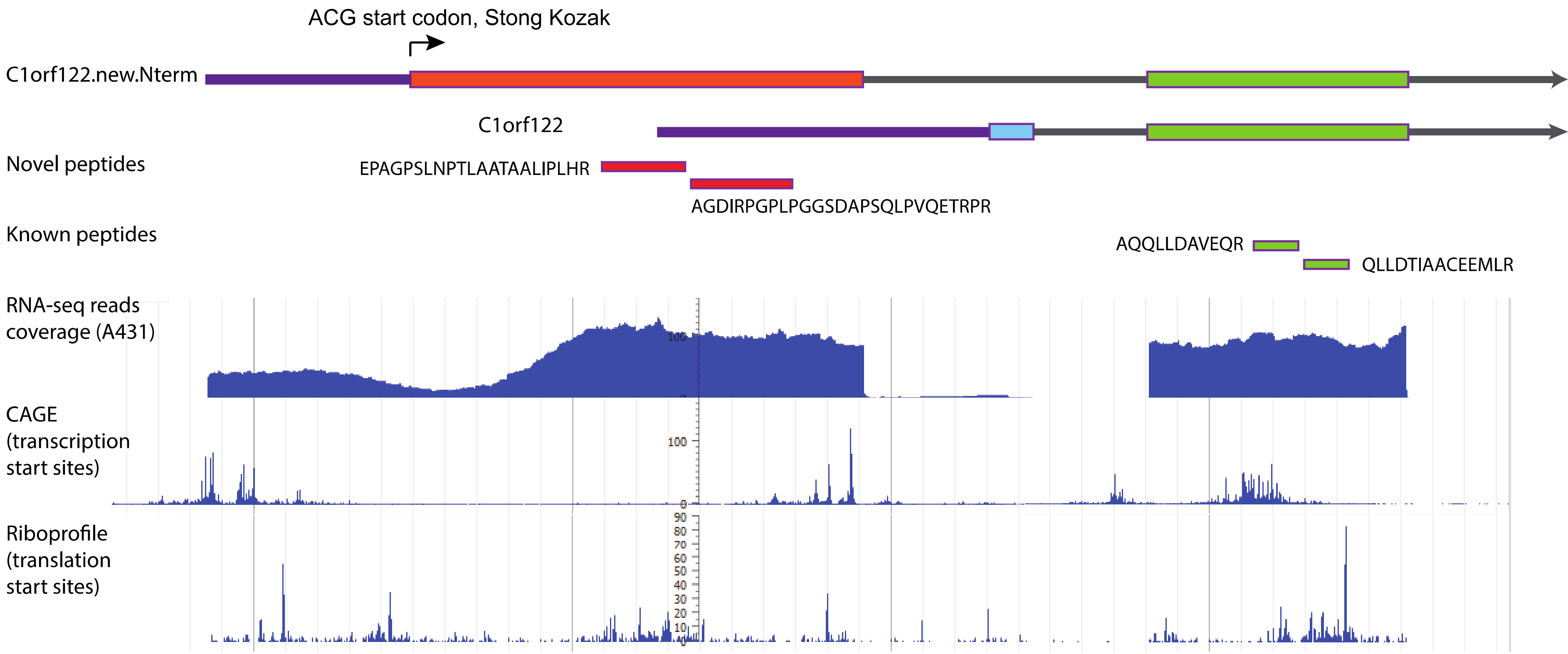

ACG start codon, Stong Kozak
C1orf122.new.Nterm
C1orf122
Novel peptides
EPAGPSLNPTLAATAALIPLHR
AGDIRPGPLPGGSDAPSQLPVQETRPR
Known peptides
AQQLLDAVEQR
QLLDTIAACEEMLR
RNA-seq reads coverage (A431)
CAGE (transcription start sites)
Riboprofile (translation start sites)

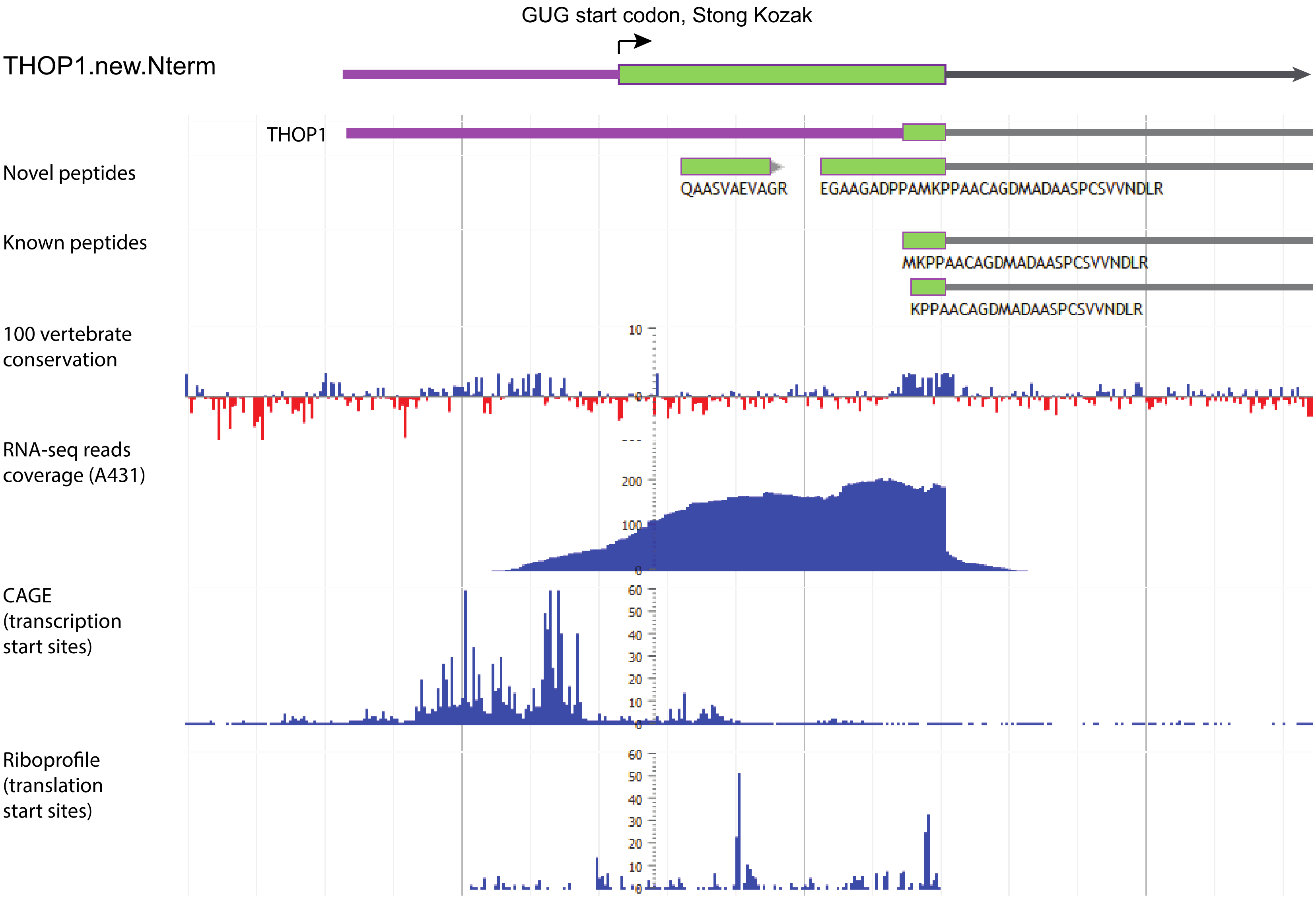
GUG start codon, Stong Kozak
THOP1.new.Nterm
THOP1
Novel peptides
QAASVAEVAGR
EGAAGADPPAMKPPAACAGDMADAASPCSVVNDLR
Known peptides
MKPPAACAGDMADAASPCSVVNDLR
KPPAACAGDMADAASPCSVVNDLR
100 vertebrate conservation
RNA-seq reads coverage (A431)
CAGE (transcription start sites)
Riboprofile (translation start sites)

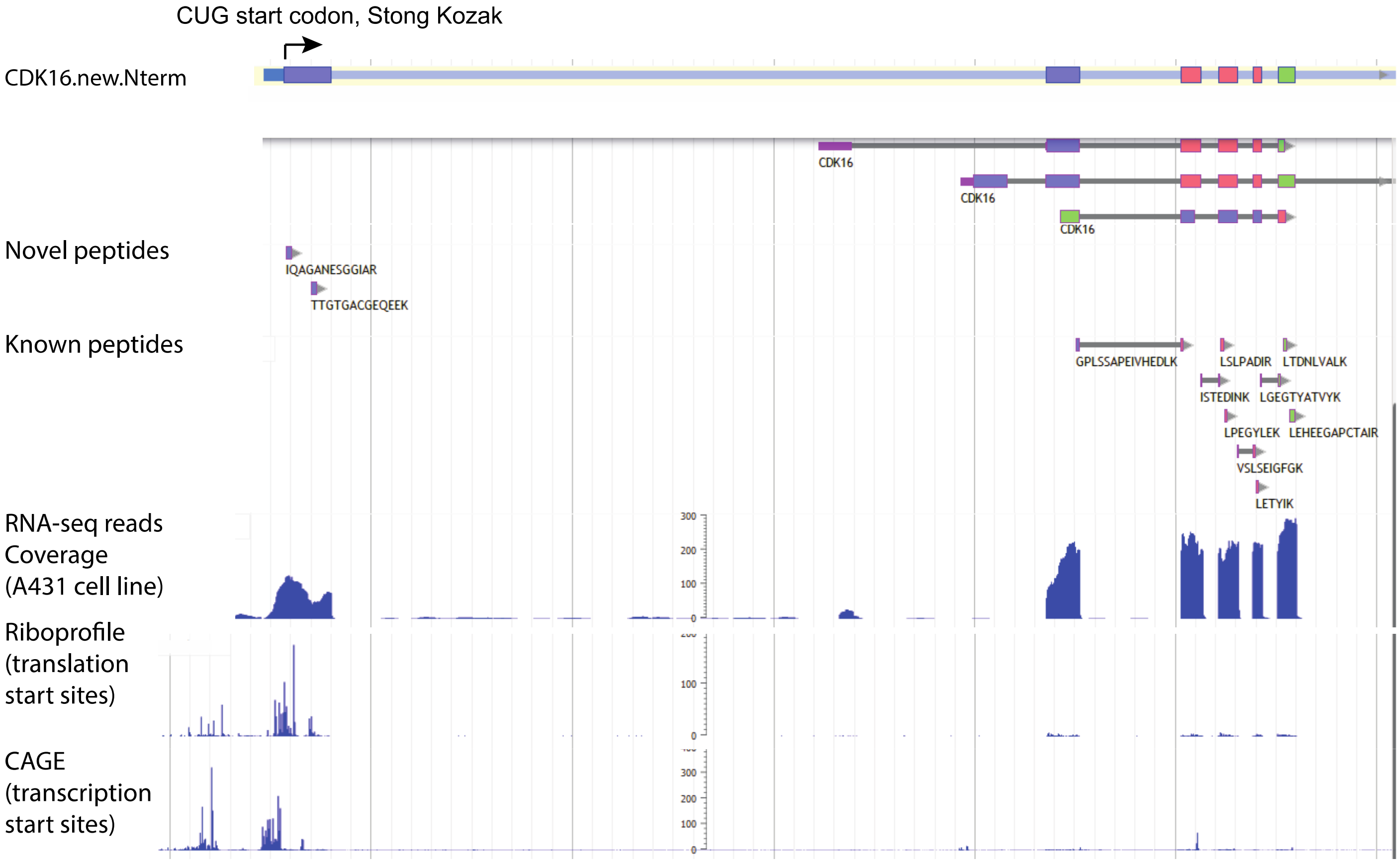
CUG start codon, Stong Kozak
CDK16.new.Nterm
CDK16
CDK16
CDK16
Novel peptides
IQAGANESGGIAR
TTGTGACGEQEEK
Known peptides
GPLSSAPEIVHEDLK
LSLPADIR
LTDNLVALK
ISTEDINK
LGEGTYATVYK
LPEGYLEK
LEHEEGAPCTAIR
VSLSEIGFGK
LETYIK
RNA-seq reads Coverage (A431 cell line)
Riboprofile (translation start sites)
CAGE (transcription start sites)

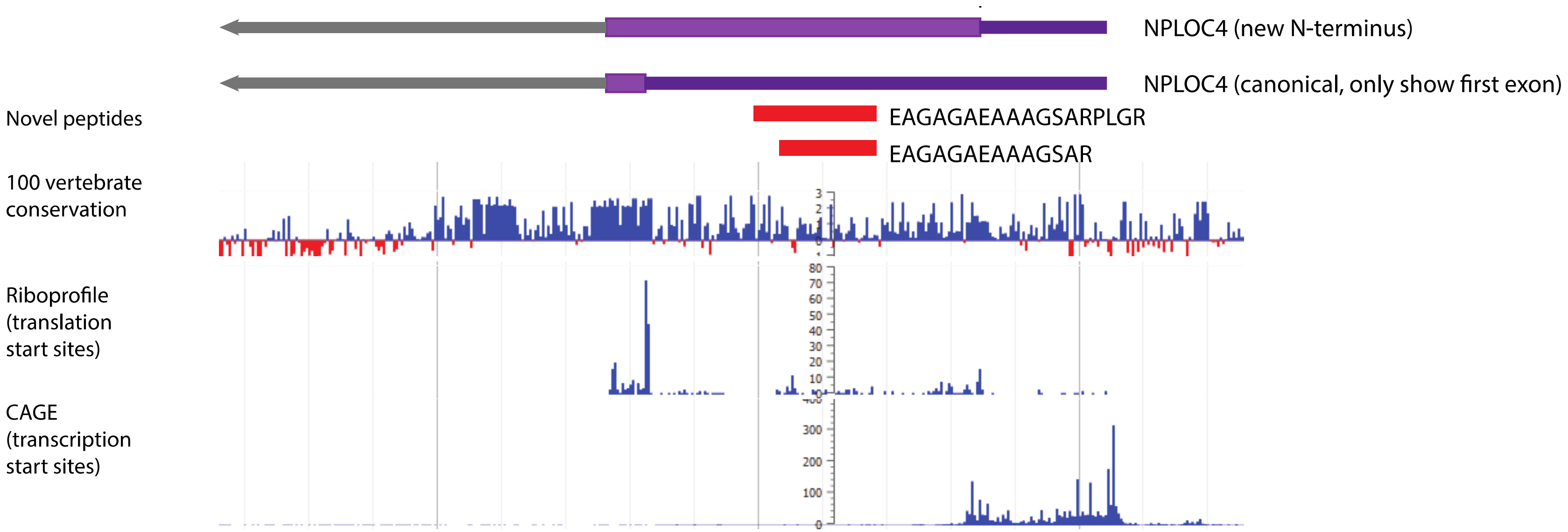
NPLOC4 (new N-terminus)
NPLOC4 (canonical, only show first exon)
Novel peptides
EAGAGAEAAAGSARPLGR
EAGAGAEAAAGSAR
100 vertebrate conservation
Riboprofile (translation start sites)
CAGE (transcription start sites)

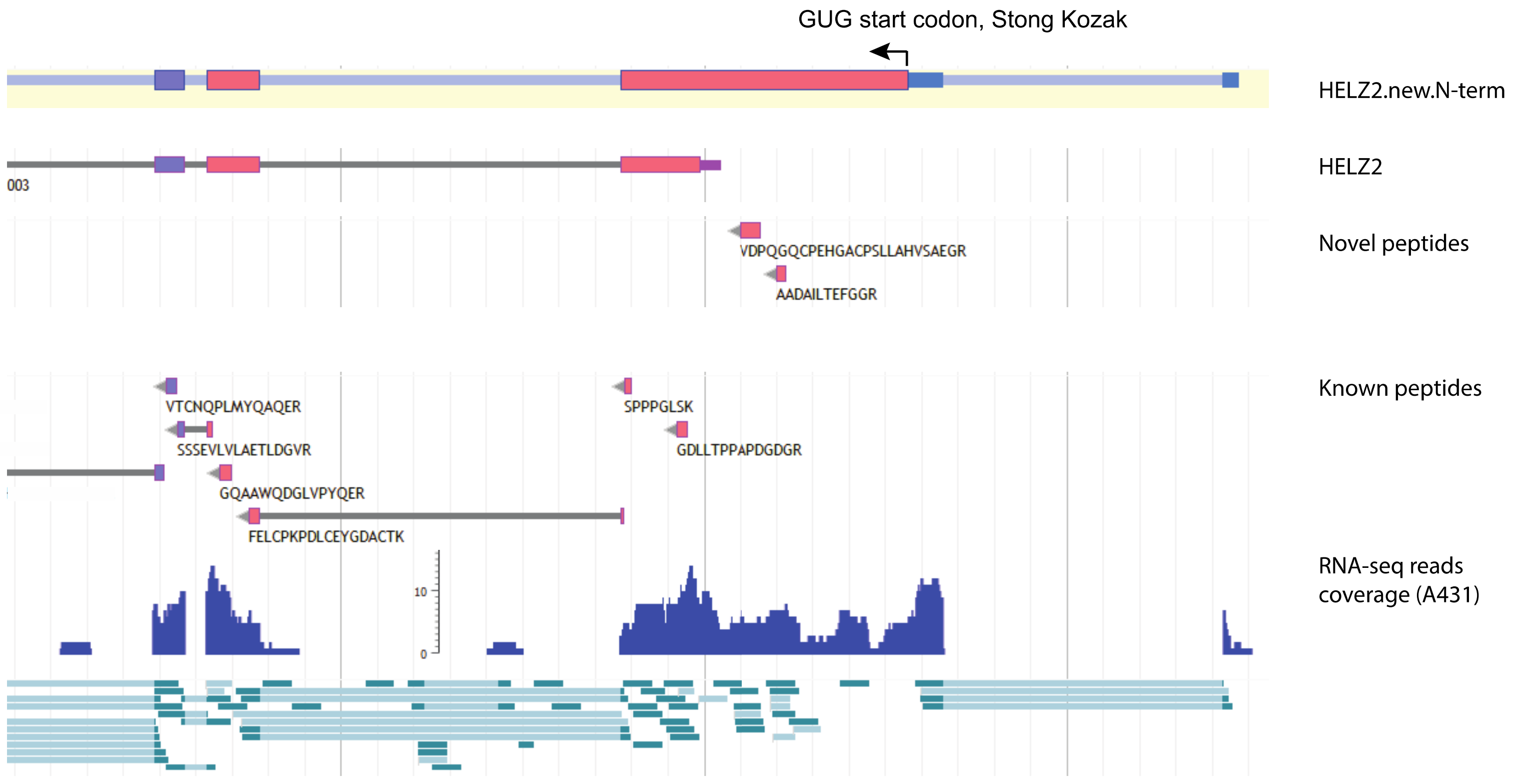
GUG start codon, Stong Kozak
HELZ2.new.N-term
HELZ2
003
Novel peptides
VDPQGQCPEHGACPSLLAHVSAEGR
AADAILTEFGGR
Known peptides
VTCNQPLMYQAQER
SPPPGLSK
SSSEVLVLAETLDGVR
GDLLTPPAPDGDGR
GQAAWQDGLVPYQER
FELCPKPDLCEYGDACTK
10
0
RNA-seq reads coverage (A431)

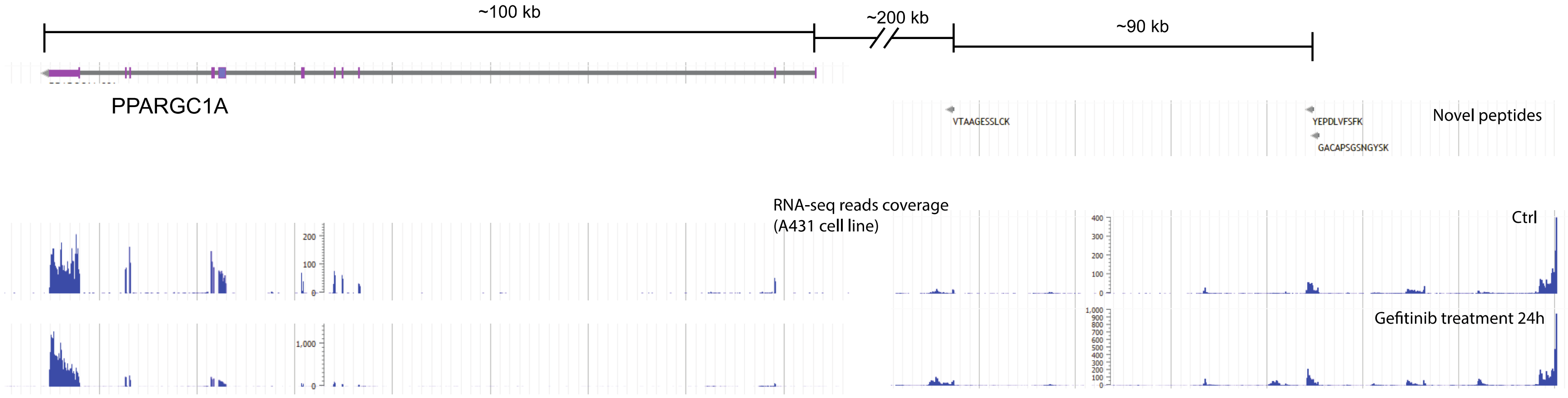
~100 kb
~200 kb
~90 kb
PPARGC1A
Novel peptides
VTAAGESSLCK
YEPDLVFSFK
GACAPSGSNGYSK
RNA-seq reads coverage
(A431 cell line)
Ctrl
Gefitinib treatment 24h

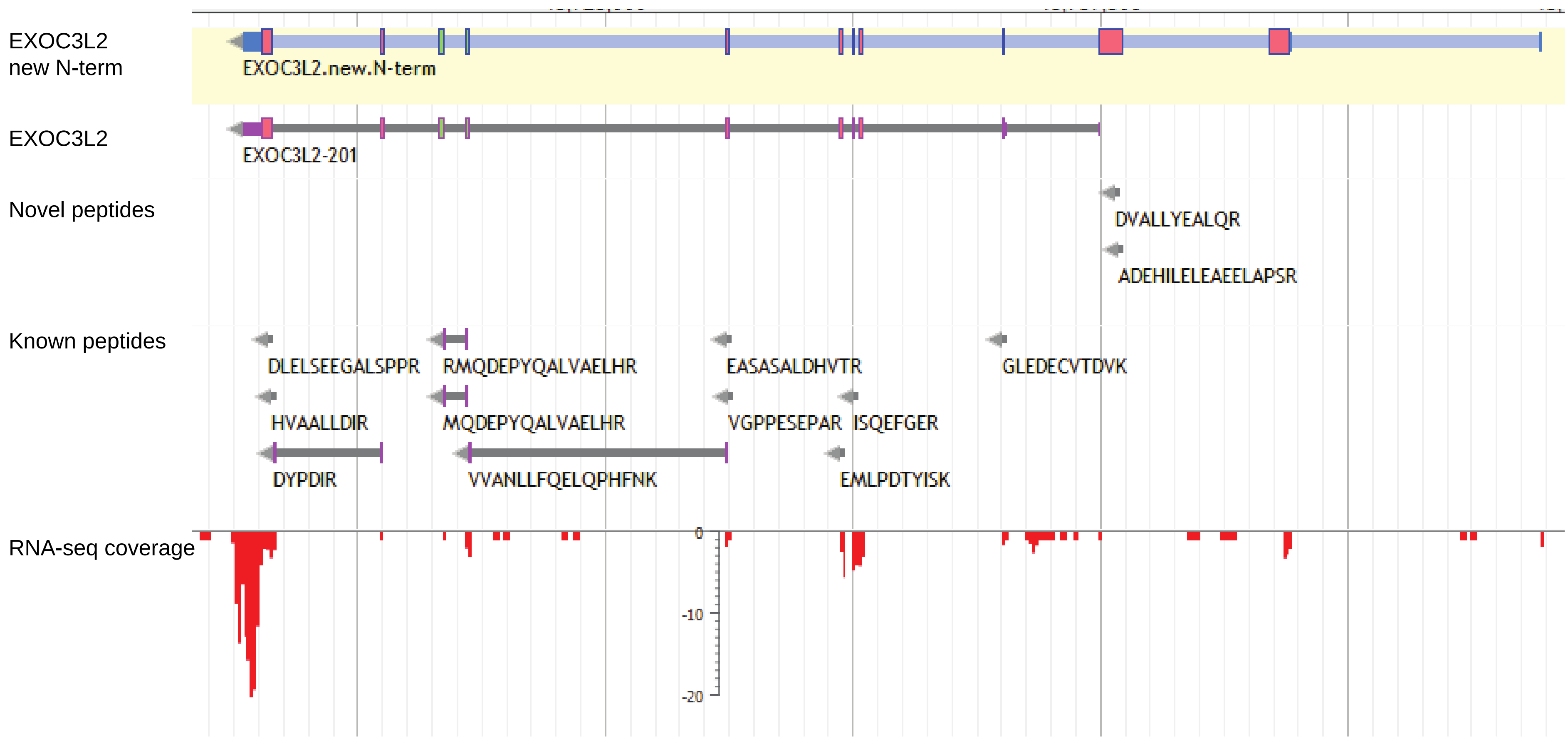

EXOC3L2 new N-term
EXOC3L2.new.N-term
EXOC3L2
EXOC3L2-201
Novel peptides
DVALLYEALQR
ADEHILELEAEELAPSR
Known peptides
DLELSEEGALSPPR
RMQDEPYQALVAELHR
EASASALDHVTR
GLEDECVTDVK
HVAALLDIR
MQDEPYQALVAELHR
VGPPESEPAR
ISQEFGER
DYPDIR
VVANLLFQELQPHFNK
EMLPDTYISK
RNA-seq coverage
0
-10
-20

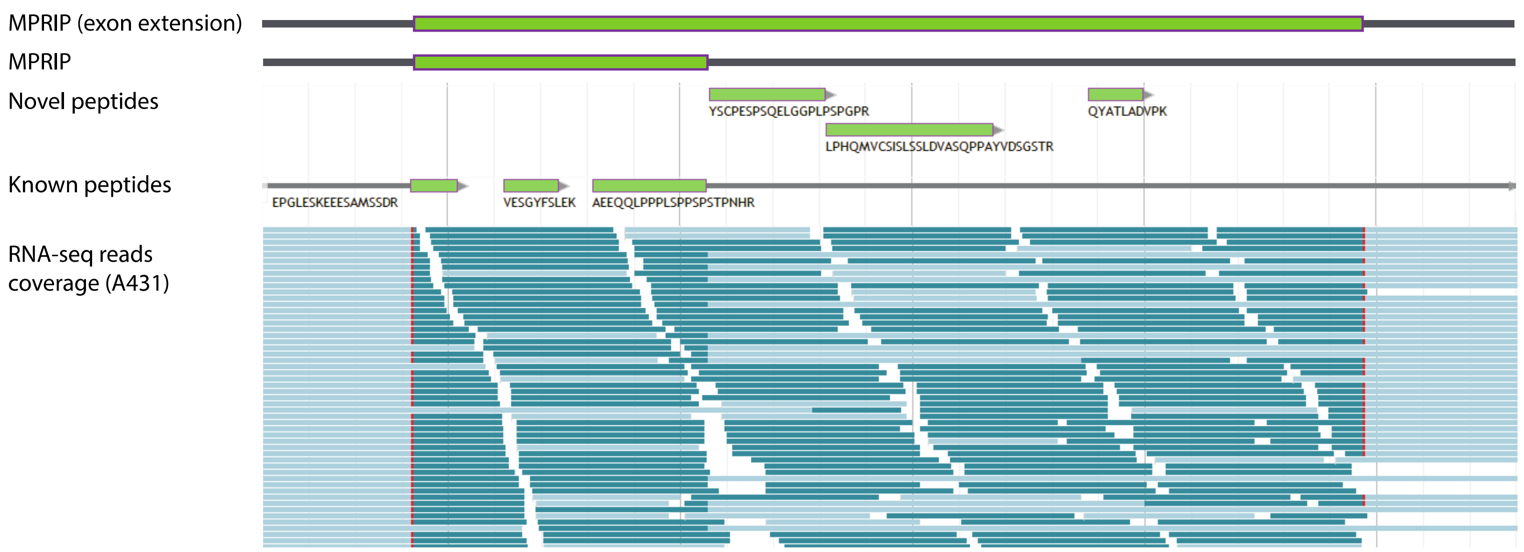

MPRIP (exon extension)
MPRIP
Novel peptides
YSCPESPSQELGGPLPSPGPR
QYATLADVPK
LPHQMVCSISLSSLDVASQPPAYVDSGSTR
Known peptides
EPGLESKEEESAMSSDR
VESGYFSLEK
AEEQQLPPPLSPPSPSTPNHR
RNA-seq reads
coverage (A431)

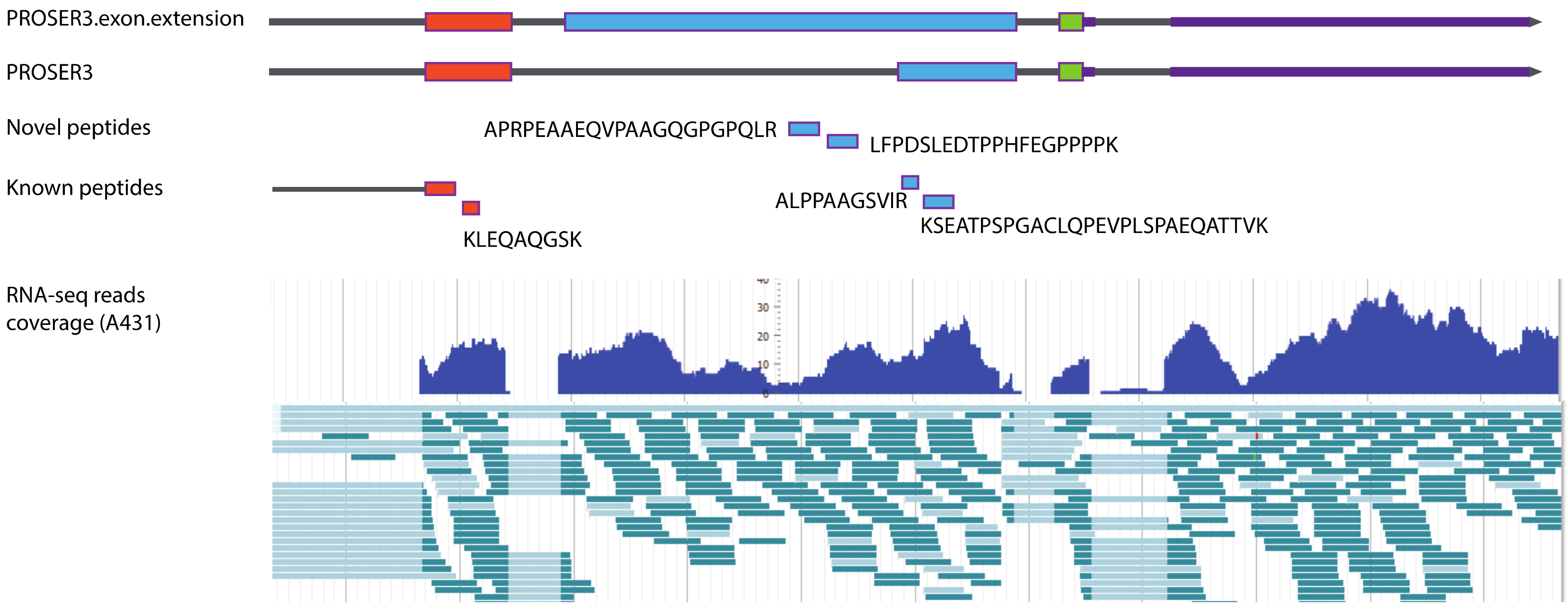

PROSER3.exon.extension
PROSER3
Novel peptides
APRPEAAEQVPAAGQGPGPQLR
LFPDSLEDTPPHFEGPPPPK
Known peptides
ALPPAAGSVIR
KSEATPSPGACLQPEVPLSPAEQATTVK
KLEQAQGSK
RNA-seq reads coverage (A431)
30
20
10
0

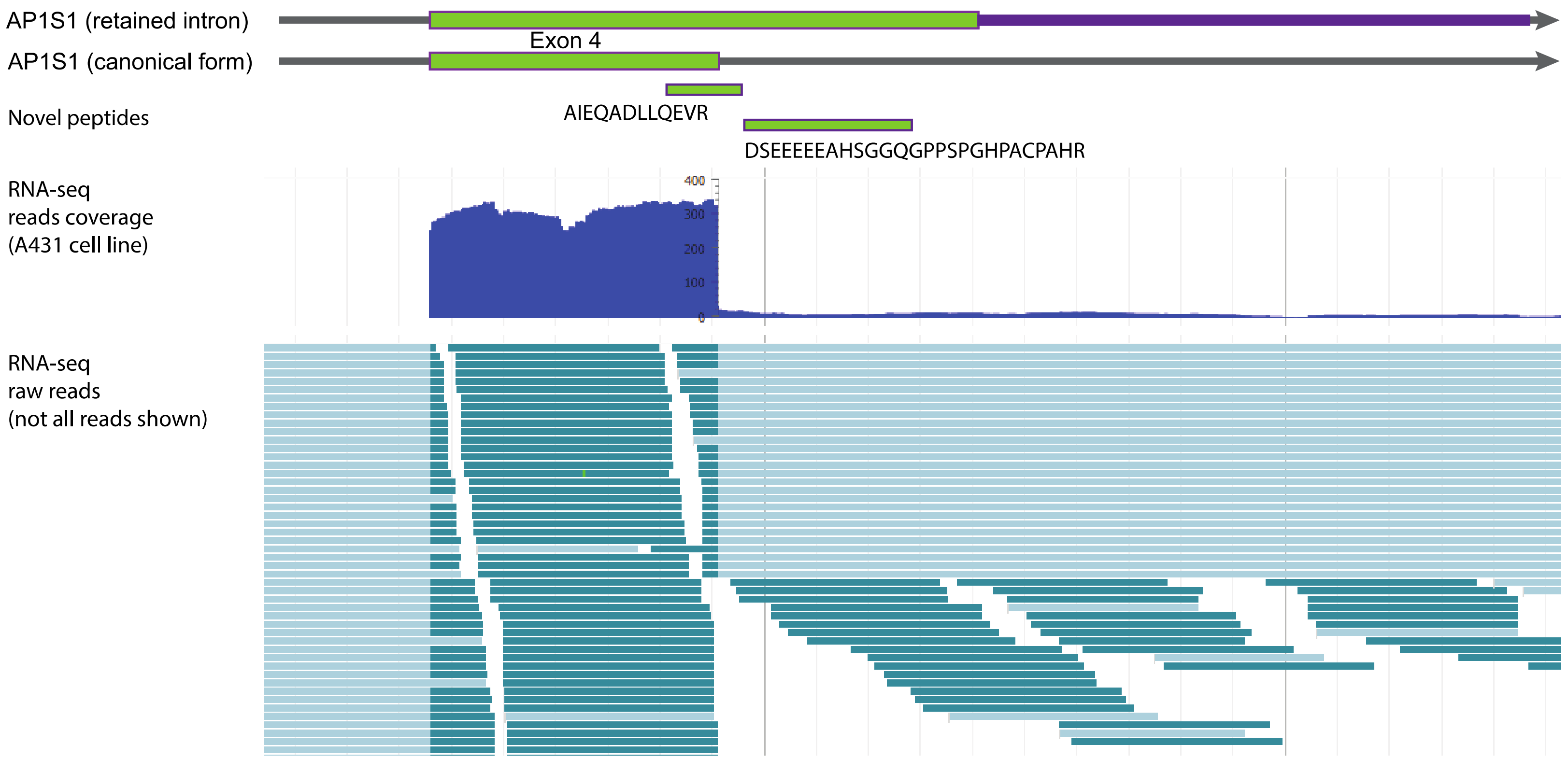
AP1S1 (retained intron)
Exon 4
AP1S1 (canonical form)
Novel peptides
AIEQADLLQEVR
DSEEEEEAHSGGQGPPSPGHPACPAHR
RNA-seq
reads coverage
(A431 cell line)
400
300
200
100
0
RNA-seq
raw reads
(not all reads shown)

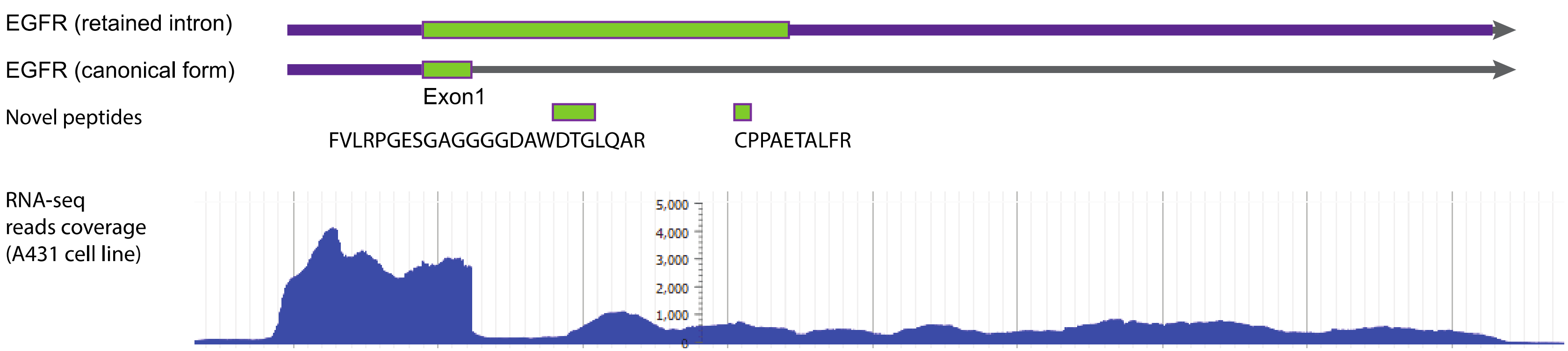

EGFR (retained intron)
EGFR (canonical form)
Exon1
Novel peptides
FVLRPGESGAGGGGDAWDTGLQAR
CPPAETALFR
RNA-seq
reads coverage
(A431 cell line)
5,000
4,000
3,000
2,000
1,000
0

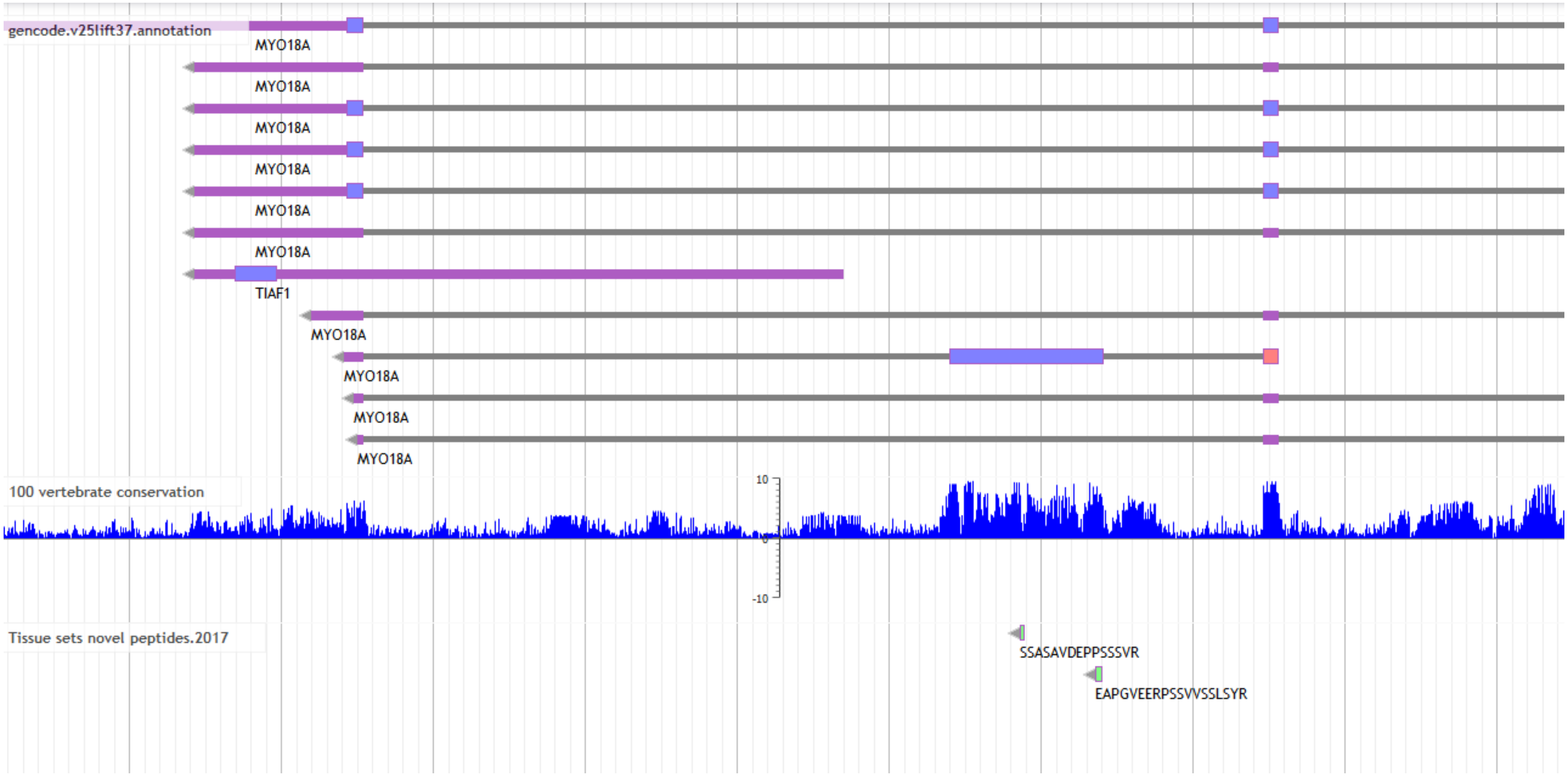
gencode.v25lift37.annotation
MYO18A
MYO18A
MYO18A
MYO18A
MYO18A
MYO18A
TIAF1
MYO18A
MYO18A
MYO18A
MYO18A
100 vertebrate conservation
10
0
-10
Tissue sets novel peptides.2017
SSASAVDEPPSSSVR
EAPGVEERPSSVVSSLSYR

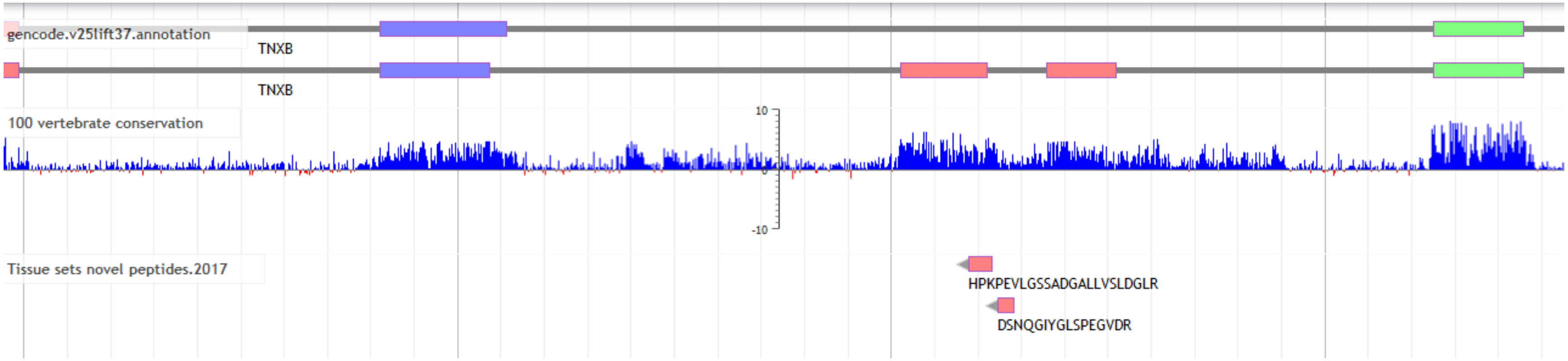
gencode.v25lift37.annotation
TNXB
TNXB
100 vertebrate conservation
10
0
-10
Tissue sets novel peptides.2017
HPKPEVLGSSADGALLVSLDGLR
DSNQGIYGLSPEGVDR

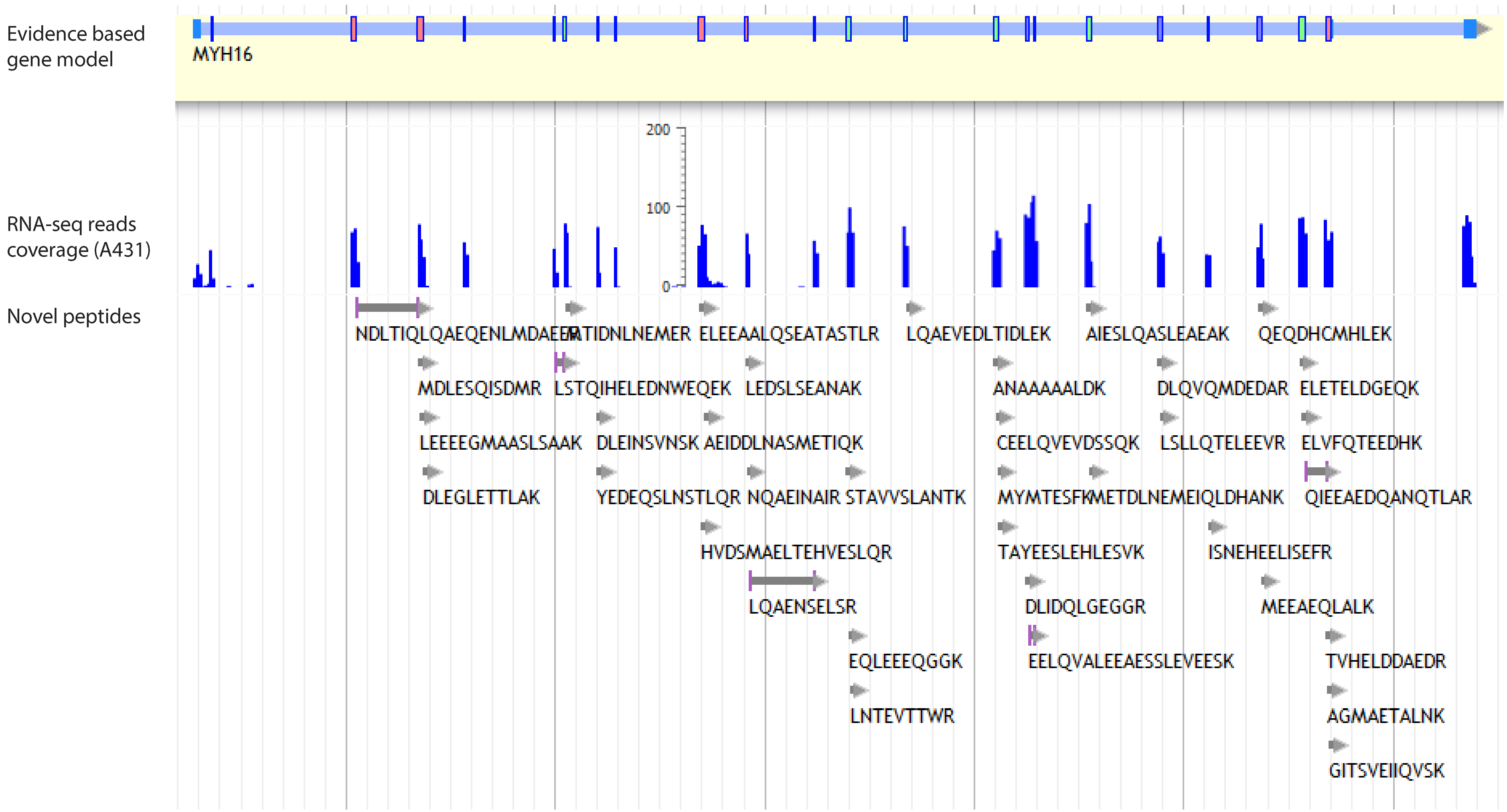

Evidence based gene model
MYH16
RNA-seq reads coverage (A431)
200
100
0
Novel peptides
NDLTIQLQAEQENLMDAEER
ATIDNLNEMER
ELEEAALQSEATASTLR
LQAEVEDLTIDLEK
AIESLQASLEAEAK
QEQDHCMHLEK
MDLESQISDMR
LSTQIHELEDNWEQEK
LEDSLSEANAK
ANAAAAALDK
DLQVQMDEDAR
ELETELDGEQK
LEEEEGMAASLSAAK
DLEINSVNSK
AEIDDLNASMETIQK
CEELQVEVDSSQK
LSLLQTELEEVR
ELVFQTEEDHK
DLEGLETTLAK
YEDEQSLNSTLQR
NQAEINAIR
STAVVSLANTK
MYMTESFKMETDLNEMEIQLDHANK
QIEEAEDQANQTLAR
HVDSMAELTEHVESLQR
TAYEESLEHLESVK
ISNEHEELISEFR
LQAENSELSR
DLIDQLGEGGR
MEEAEQLALK
EQLEEEQGGK
EELQVALEEAESSLEVEESK
TVHELDDAEDR
LNTEVTTWR
AGMAETALNK
GITSVEIIQVSK

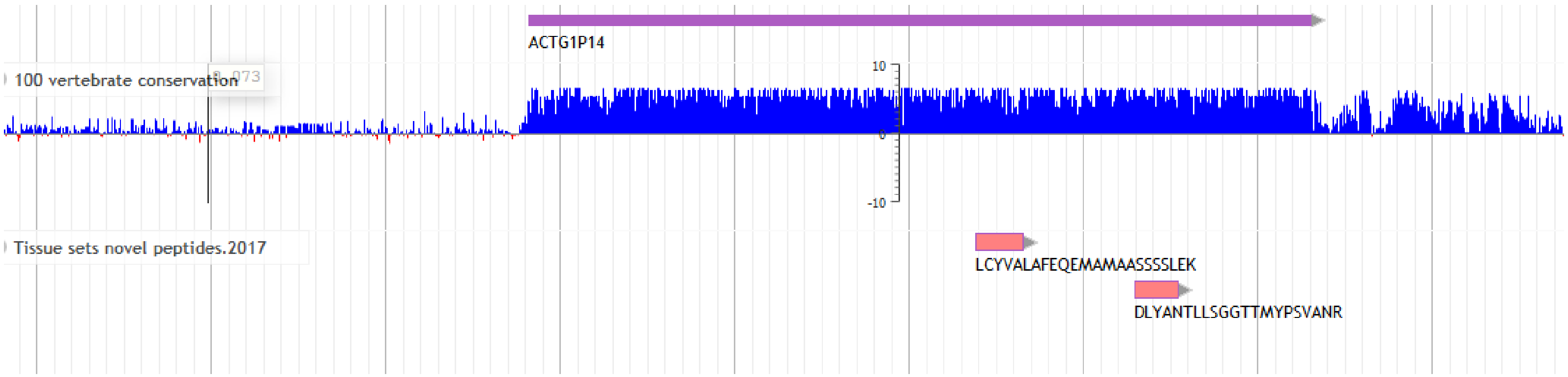
ACTG1P14
100 vertebrate conservation
10
0
-10
Tissue sets novel peptides.2017
LCYVALAFEQEMAMAASSSSLEK
DLYANTLLSGGTTMYPSVANR

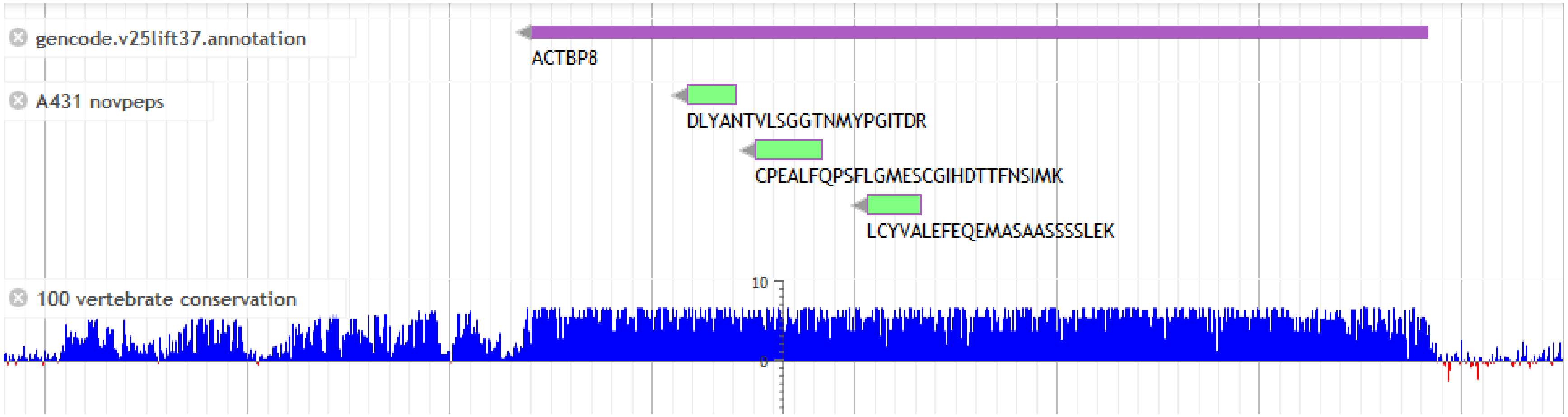
gencode.v25lift37.annotation
ACTBP8
A431 novpeps
DLYANTVLSGGTNMYPGITDR
CPEALFQPSFLGMESCGIHDTTFNSIMK
LCYVALEFEQEMASAASSSSLEK
100 vertebrate conservation
10
0

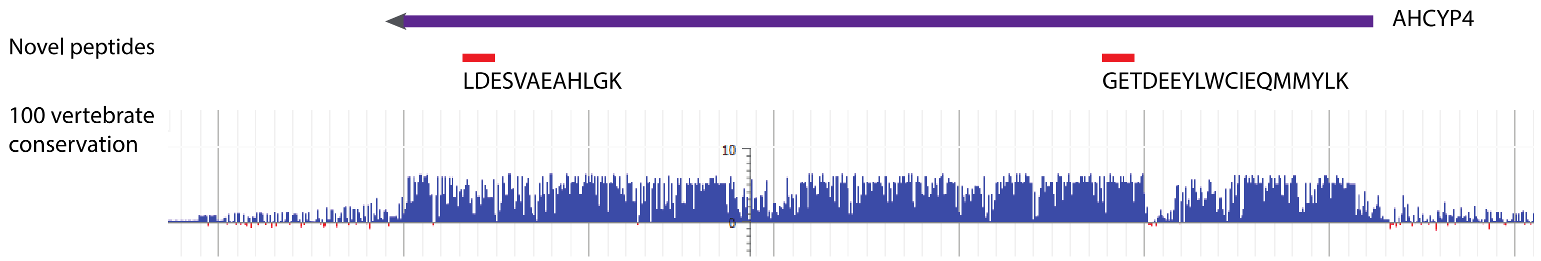
AHCYP4
Novel peptides
LDESVAEAHLGK
GETDEEYLWCIEQMMYLK
100 vertebrate conservation
10
0

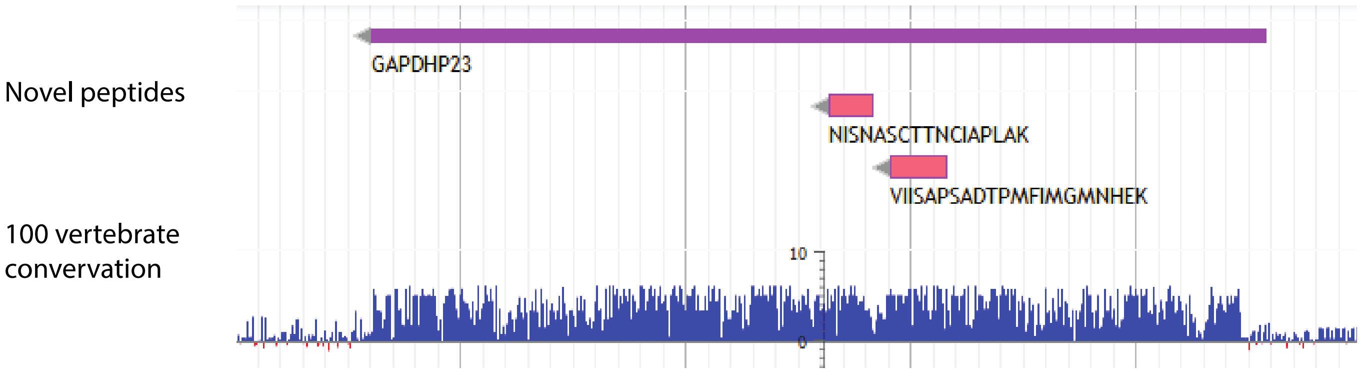
Novel peptides
100 vertebrate convervation
GAPDHP23
NISNASCTTNCIAPLAK
VIISAPSADTPMFIMGMNHEK
10
0

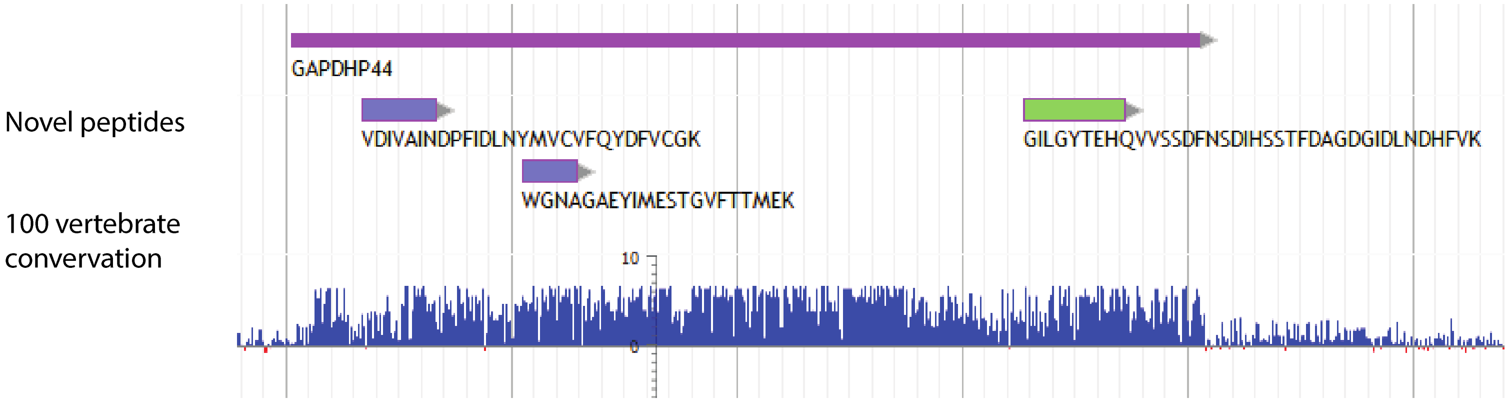
GAPDHP44
Novel peptides
VDIVAINDPFIDLNYMVCVFQYDFVCGK
WGNAGAEYIMESTGVFTTMEK
GILGYTEHQVVSSDFNSDIHSSTFDAGDGIDLNDHFVK
100 vertebrate convervation
10
0

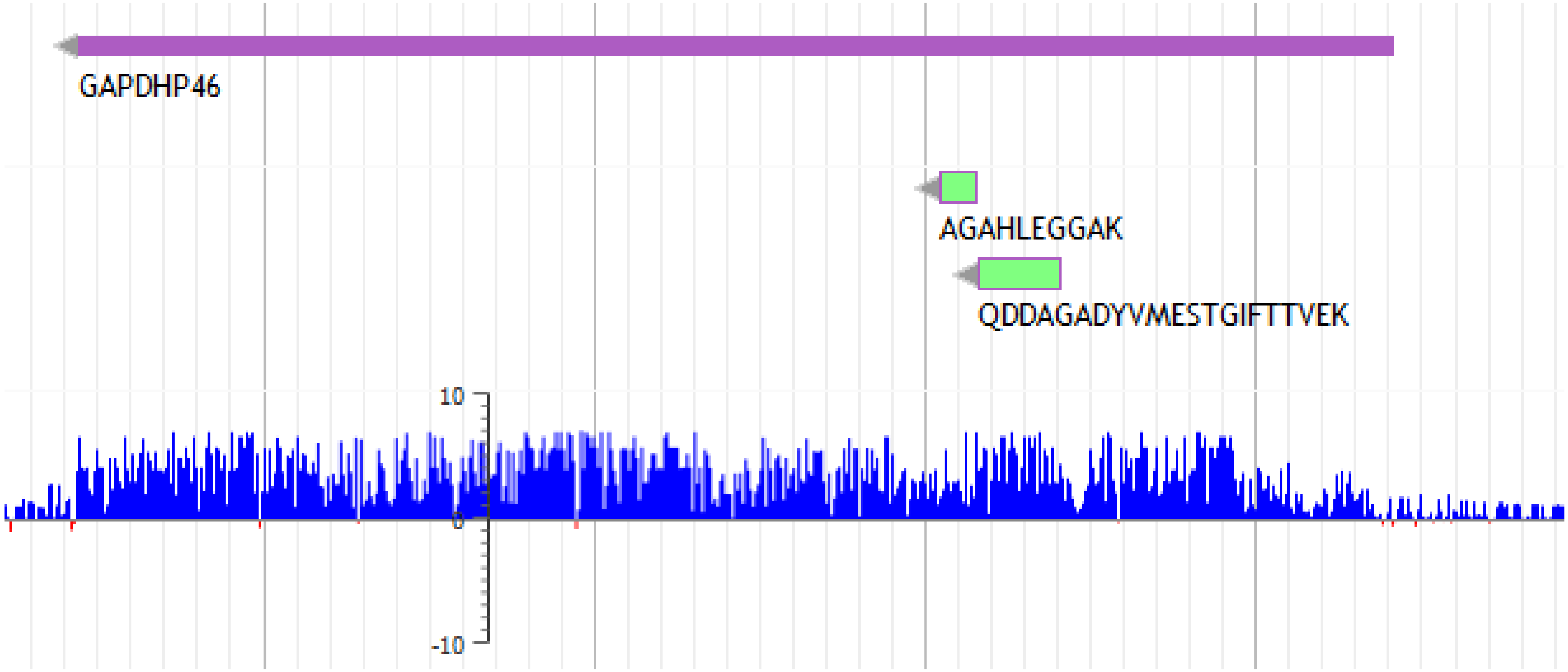

GAPDHP46
AGAHLEGGAK
QDDAGADYVMESTGIFTTVEK
10
0
-10

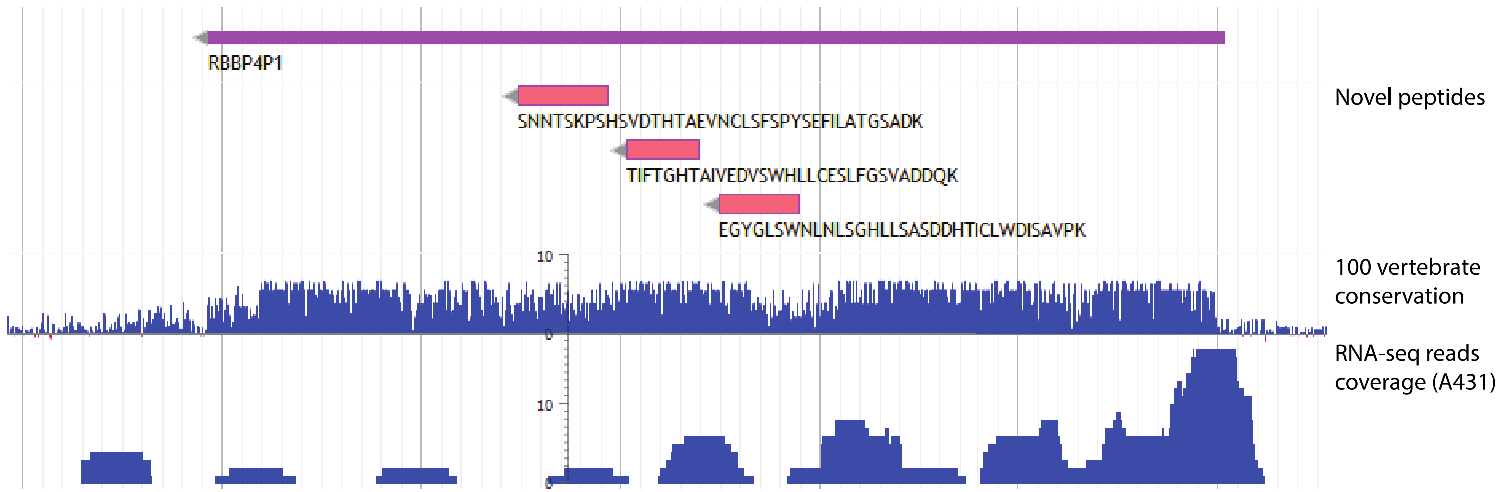

RBBP4P1
Novel peptides
SNNTSKPSHSVDTHTAEVNCLSFSPYSEFILATGSADK
TIFTGHTAIVEDVSWHLLCESLFGSVADDQK
EGYGLSWNLNLSGHLLSASDDHTICLWDISAVPK
10
0
100 vertebrate conservation
10
0
RNA-seq reads coverage (A431)

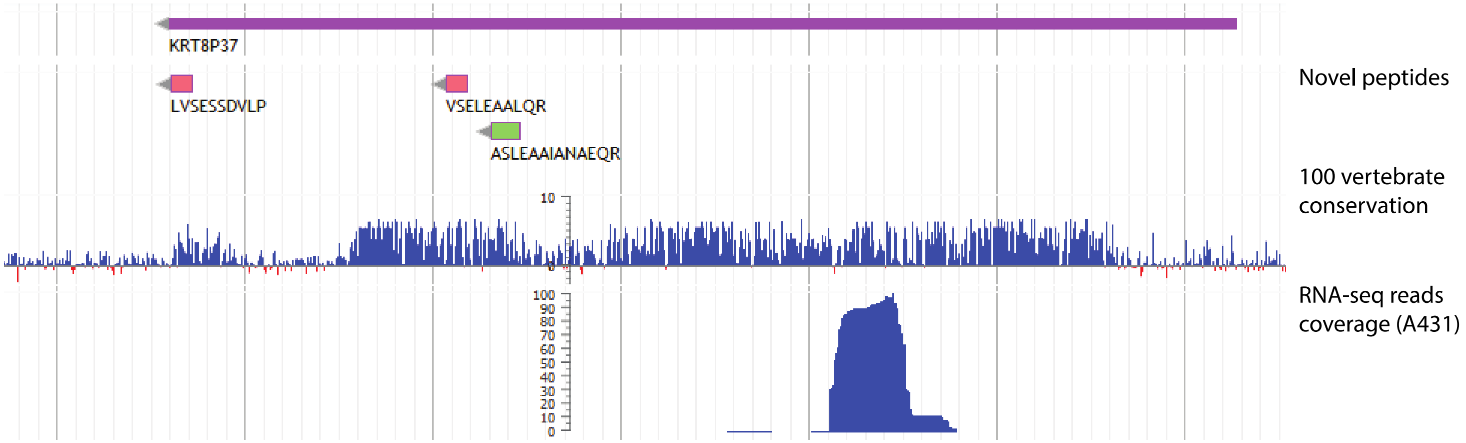
KRT8P37
Novel peptides
LVSESSDVLP
VSELEAALQR
ASLEAAIANAEQR
100 vertebrate conservation
10
0
RNA-seq reads coverage (A431)
100
90
80
70
60
50
40
30
20
10
0

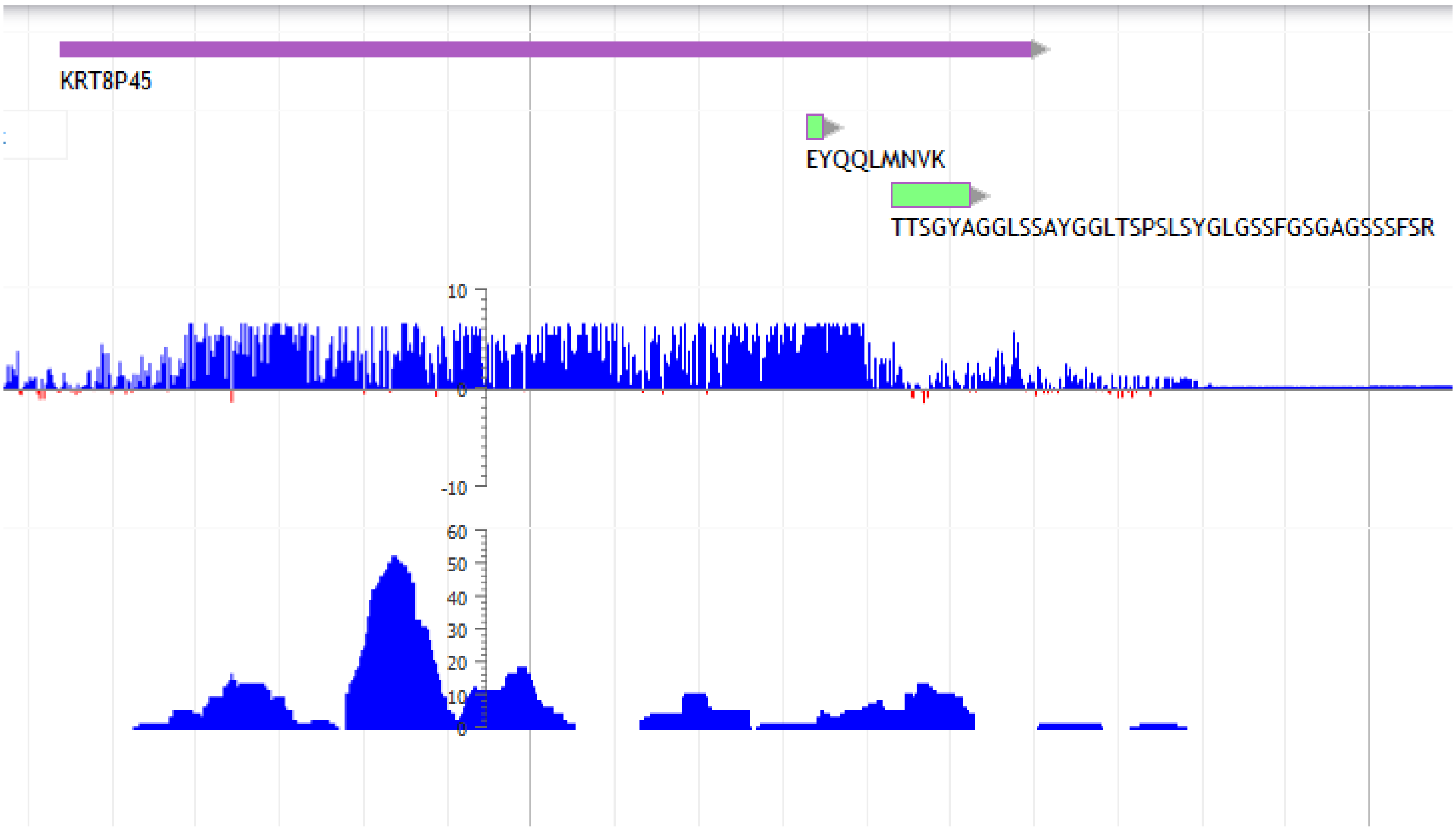
KRT8P45
EYQQLMNVK
TTSGYAGGLSSAYGGLTSPSLSYGLGSSFGSGAGSSSFSR
10
0
-10
60
50
40
30
20
10
0

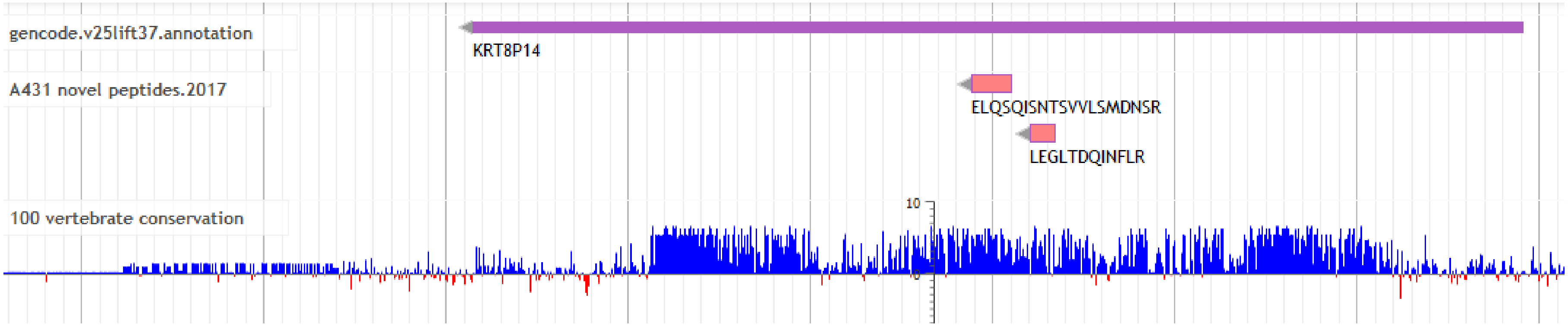
gencode.v25lift37.annotation
KRT8P14
A431 novel peptides.2017
ELQSQISNTSVVLSMDNSR
LEGLTDQINFLR
100 vertebrate conservation
10
0

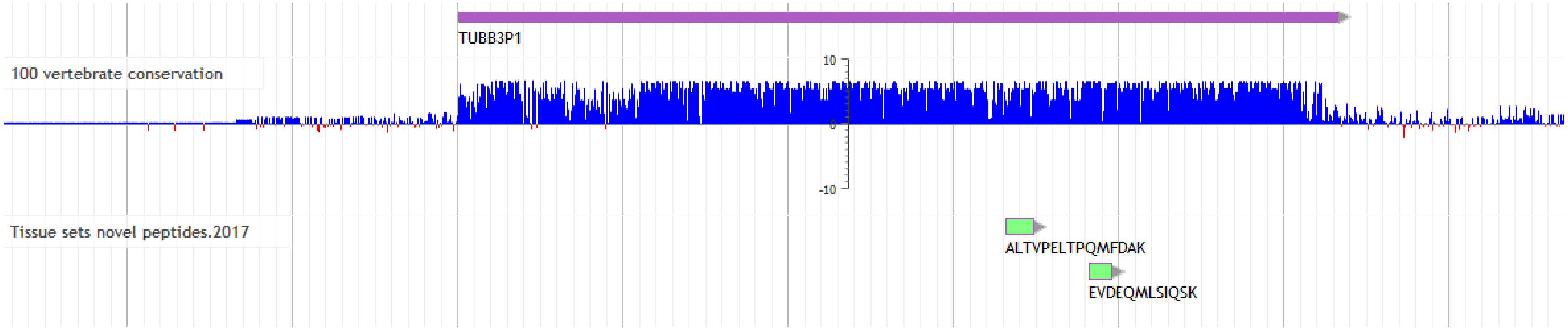
TUBB3P1
100 vertebrate conservation
10
0
-10
Tissue sets novel peptides.2017
ALTVPELTPQMFDAK
EVDEQMLSIQSK

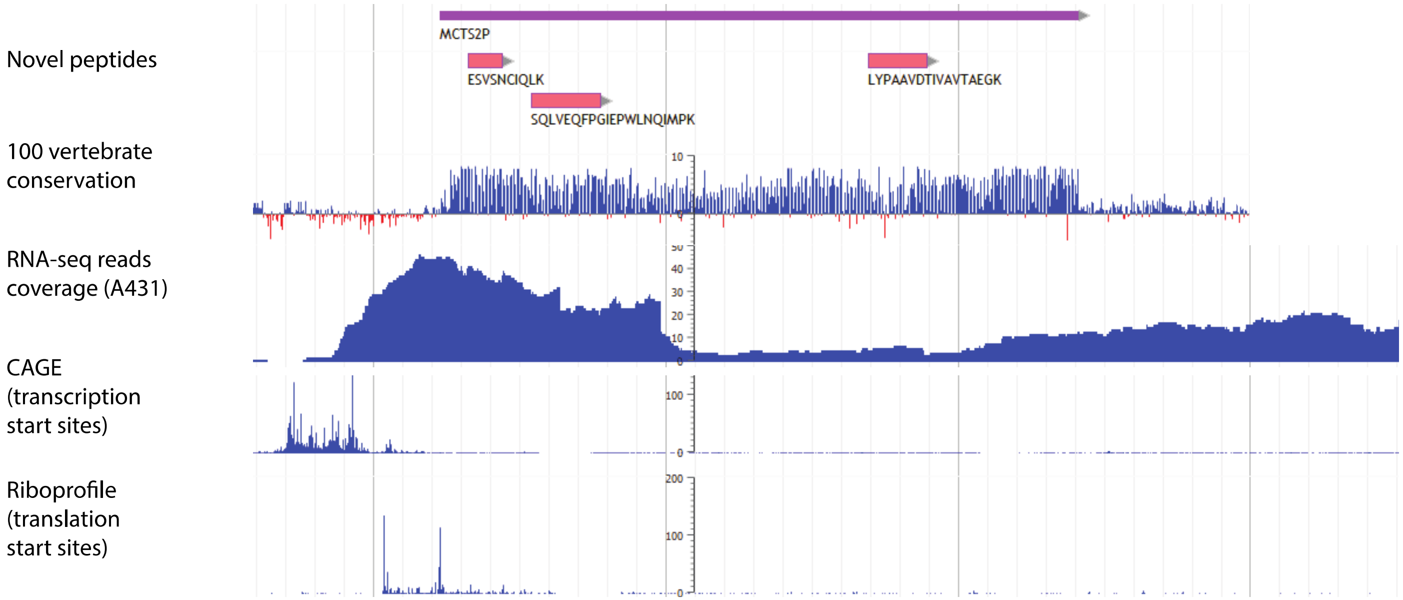

Novel peptides
100 vertebrate conservation
RNA-seq reads coverage (A431)
CAGE (transcription start sites)
Riboprofile (translation start sites)
MCTS2P
ESVSNCIQLK
SQLVEQFPGIEPWLNQIMPK
LYPAAVDTIVAVTAEGK
10
0
40
30
20
10
0
100
0
200
100
0

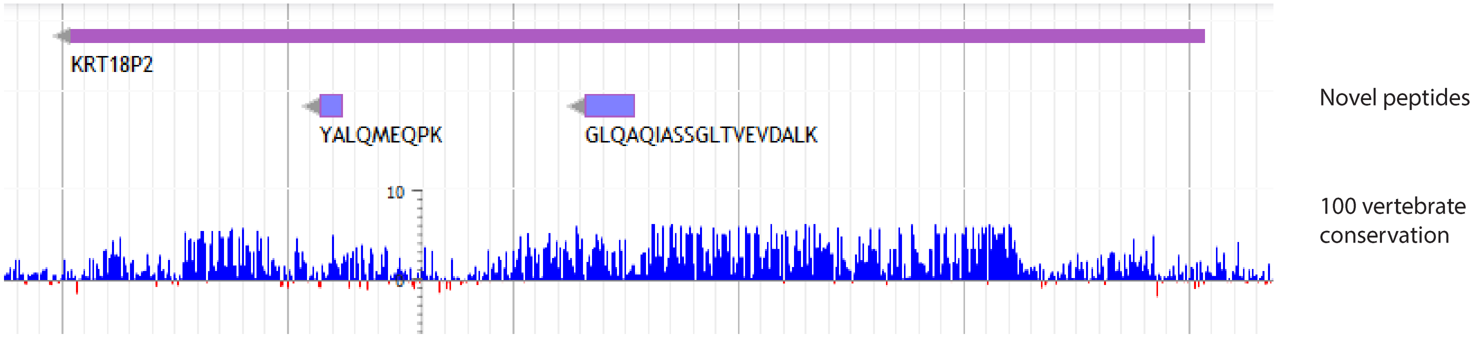
KRT18P2
YALQMEQPK
GLQAQIASSGLTVEVDALK
10
0
Novel peptides
100 vertebrate conservation

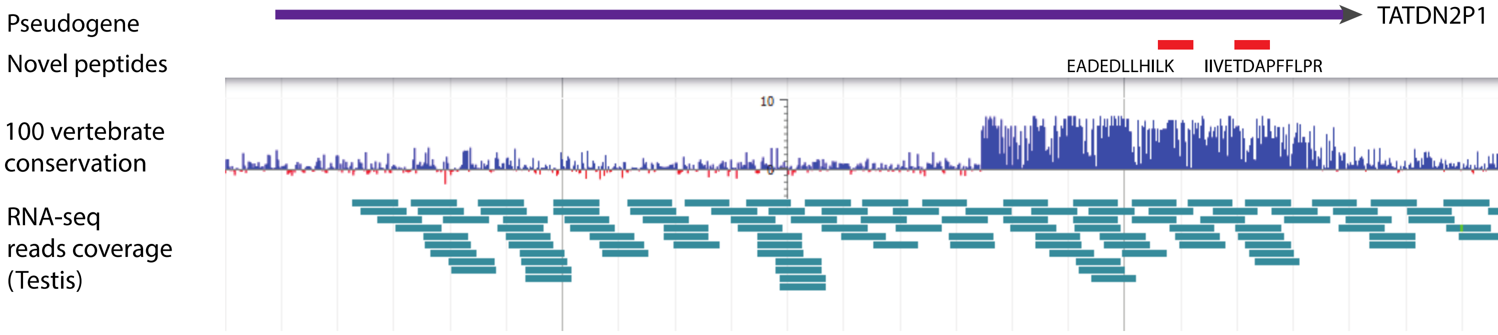

Pseudogene
TATDN2P1
Novel peptides
EADEDLLHILK
IIVETDAPFFLPR
100 vertebrate conservation
10
0
RNA-seq reads coverage (Testis)

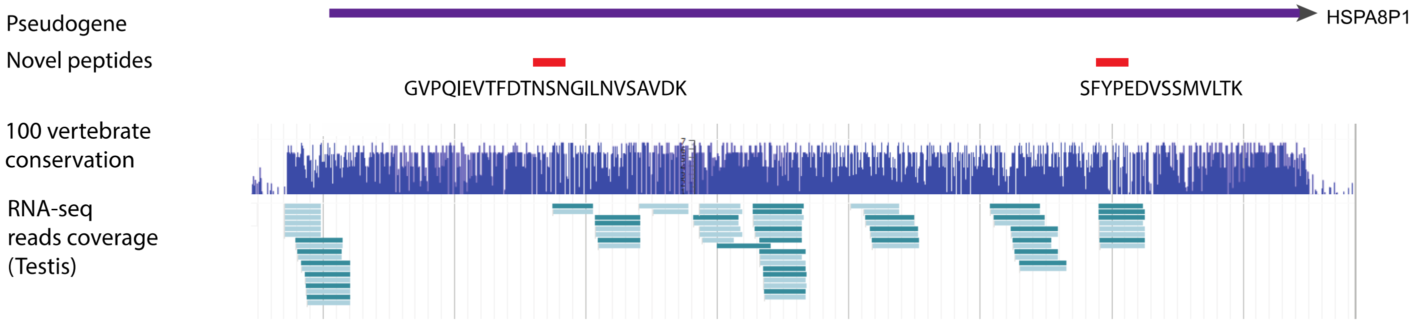
Pseudogene
HSPA8P1
Novel peptides
GVPQIEVTFDTNSNGILNVSAVDK
SFYPEDVSSMVLTK
100 vertebrate conservation
RNA-seq reads coverage (Testis)

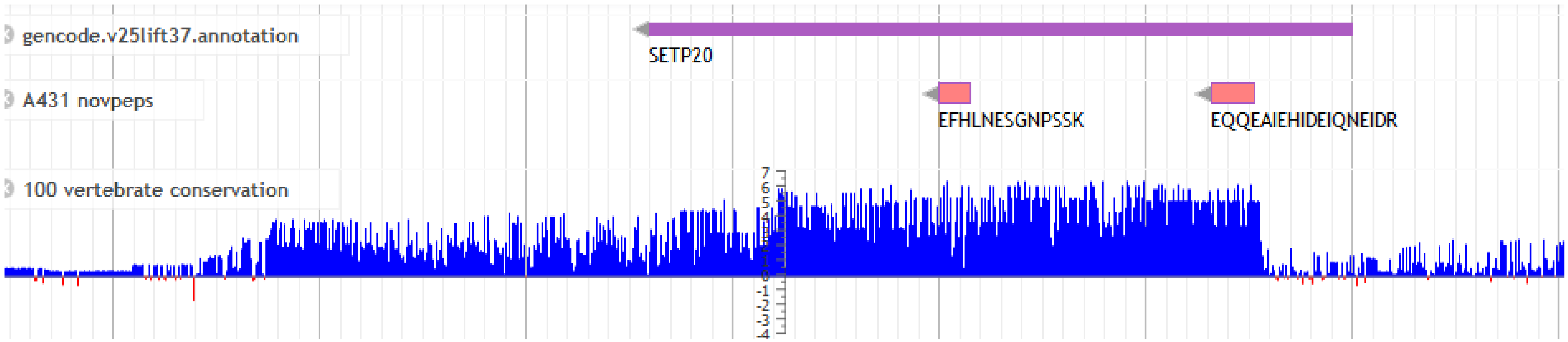
gencode.v25lift37.annotation
SETP20
A431 novpeps
EFHLNESGNPSSK
EQQEAIEHIDEIQNEIDR
100 vertebrate conservation
7
6
5
-1
-2
-3
-4

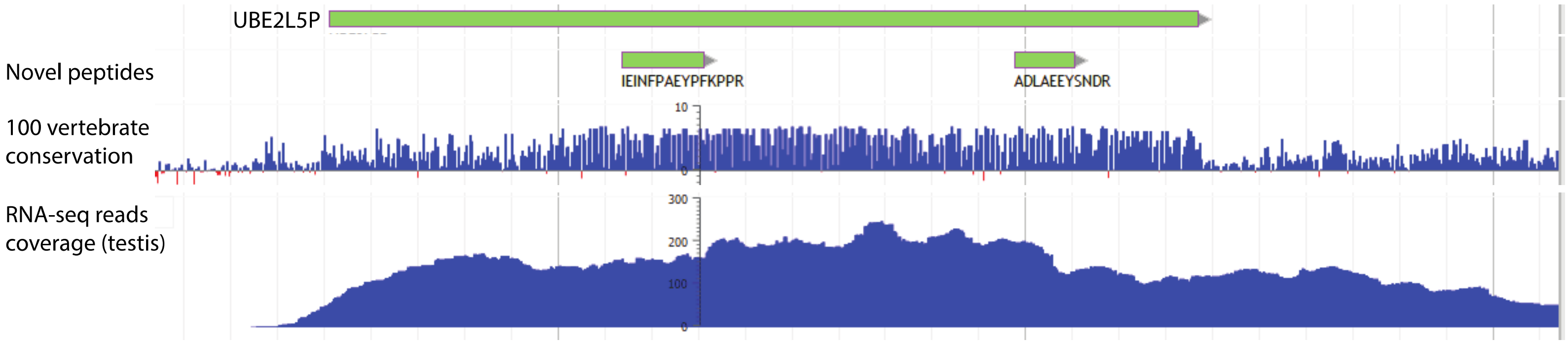

UBE2L5P
Novel peptides
IEINFPAEYPFKPPR
ADLAEEYSNDR
100 vertebrate conservation
10
0
RNA-seq reads coverage (testis)
300
200
100
0

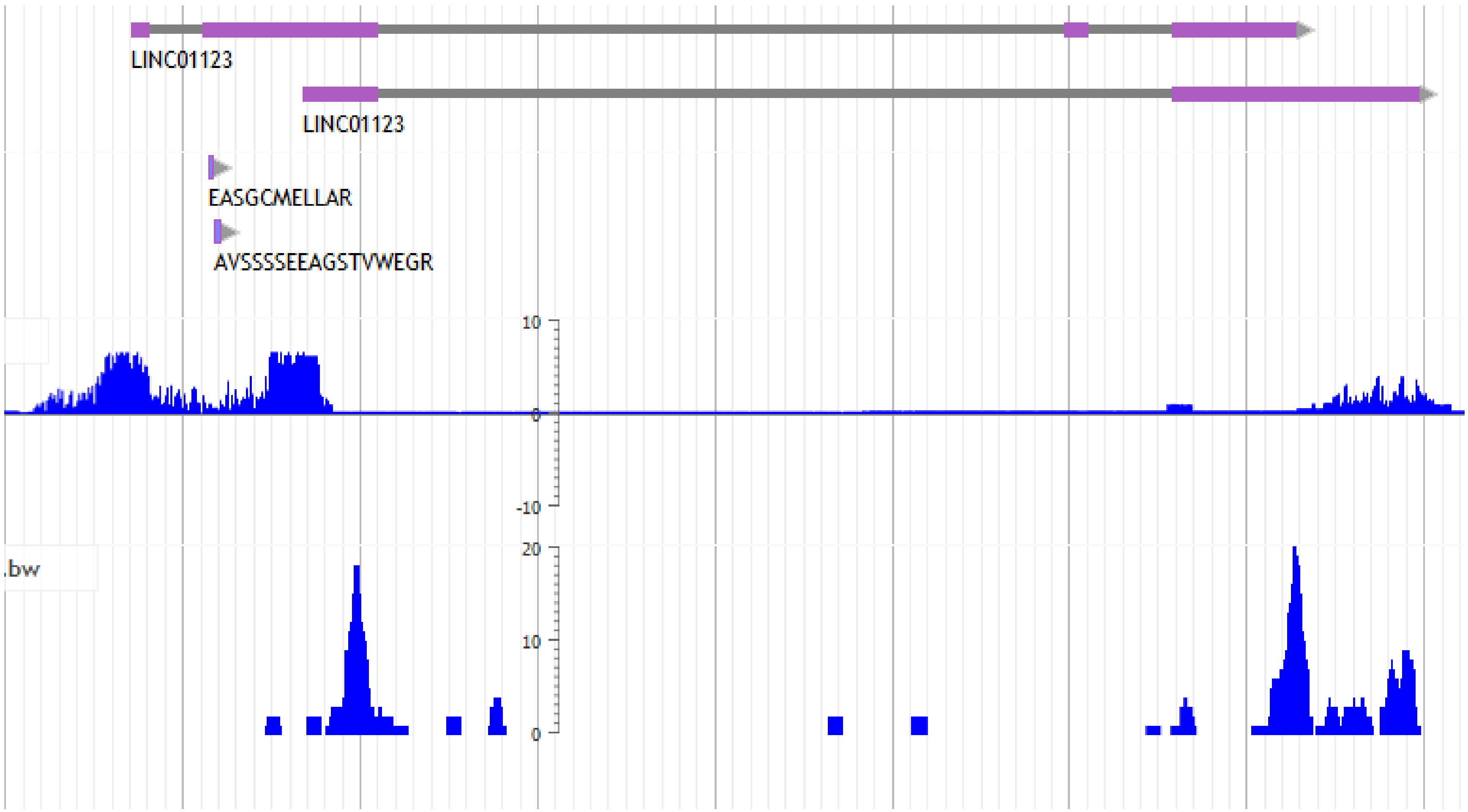
LINC01123
LINC01123
EASGCMELLAR
AVSSSSEEAGSTVWEGR
10
0
-10
.bw
20
10
0

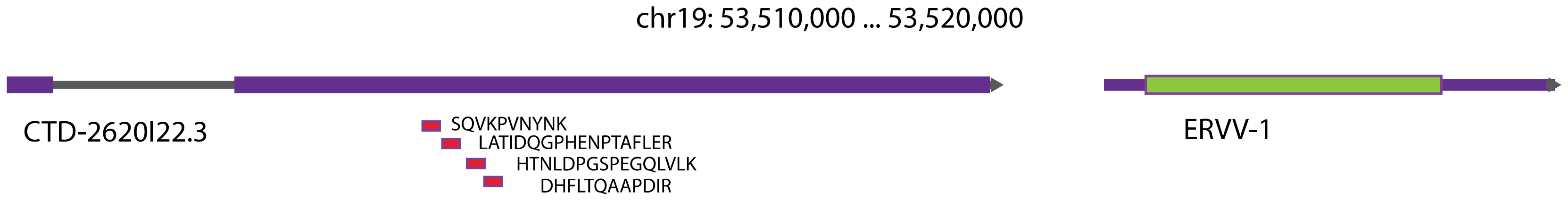

chr19: 53,510,000 ... 53,520,000
CTD-2620I22.3
SQVKPVNYNK
LATIDQGPHENPTAFLER
HTNLDPGSPEGQLVLK
DHFLTQAAPDIR
ERVV-1
