# Supplementary material for: Discovery of coding regions in the human genome by integrated proteogenomics analysis workflow
Source: Nat Commun. 2018 Mar 2;9:903. doi: 10.1038/s41467-018-03311-y (PMC5834625; doi:10.1038/s41467-018-03311-y)
Supplement: Supplementary file 11 — Supplementary Data 8 [file 41467_2018_3311_MOESM11_ESM.pdf]

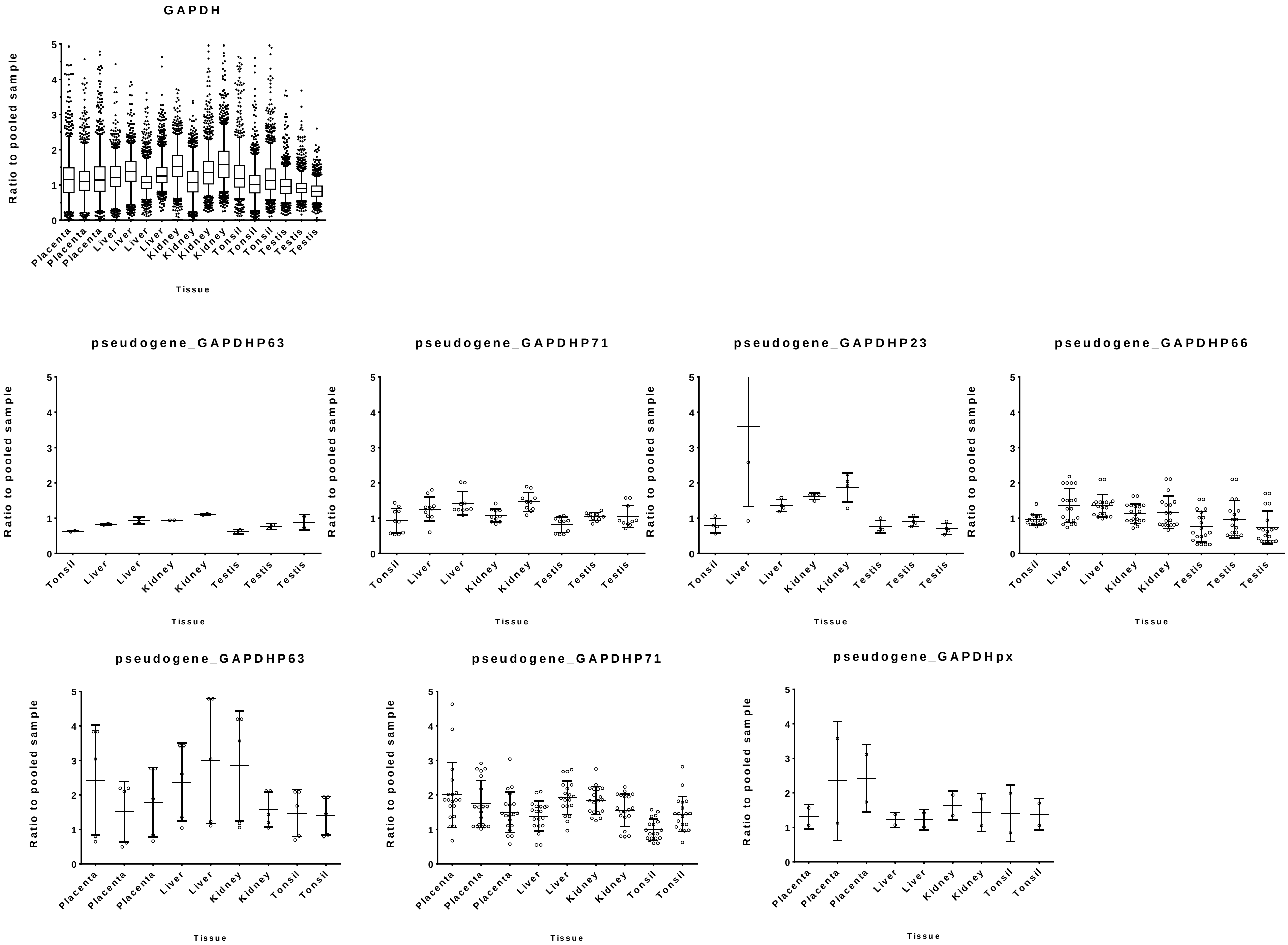
GAPDH
Ratio to pooled sample
Placenta Placenta Placenta Liver Liver Liver Liver Kidney Kidney Kidney Kidney Tonsil Tonsil Tonsil Testis Testis Testis
Tissue
pseudogene_GAPDHP63
Ratio to pooled sample
Tonsil Liver Liver Kidney Kidney Testis Testis Testis
Tissue
pseudogene_GAPDHP71
Ratio to pooled sample
Tonsil Liver Liver Kidney Kidney Testis Testis Testis
Tissue
pseudogene_GAPDHP23
Ratio to pooled sample
Tonsil Liver Liver Kidney Kidney Testis Testis Testis
Tissue
pseudogene_GAPDHP66
Ratio to pooled sample
Tonsil Liver Liver Kidney Kidney Testis Testis Testis
Tissue
pseudogene_GAPDHP63
Ratio to pooled sample
Placenta Placenta Placenta Liver Liver Kidney Kidney Tonsil Tonsil
Tissue
pseudogene_GAPDHP71
Ratio to pooled sample
Placenta Placenta Placenta Liver Liver Kidney Kidney Tonsil Tonsil
Tissue
pseudogene_GAPDHpx
Ratio to pooled sample
Placenta Placenta Placenta Liver Liver Kidney Kidney Tonsil Tonsil
Tissue

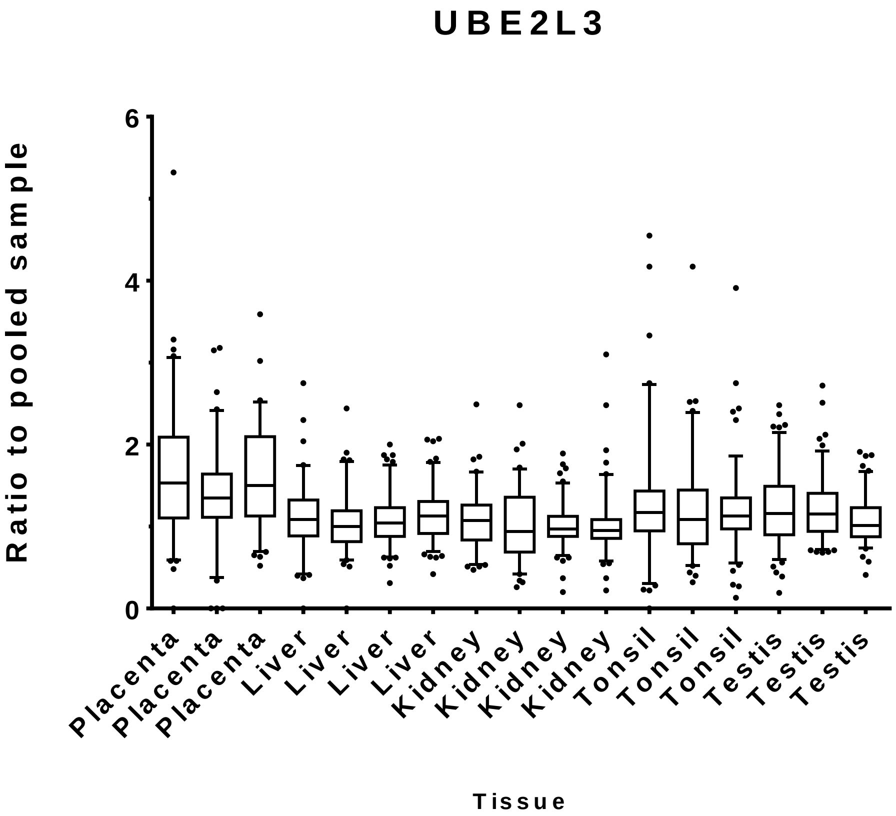
UBE2L3
Ratio to pooled sample
0
2
4
6
Placenta
Placenta
Placenta
Liver
Liver
Liver
Liver
Kidney
Kidney
Kidney
Kidney
Tonsil
Tonsil
Tonsil
Testis
Testis
Testis
Tissue

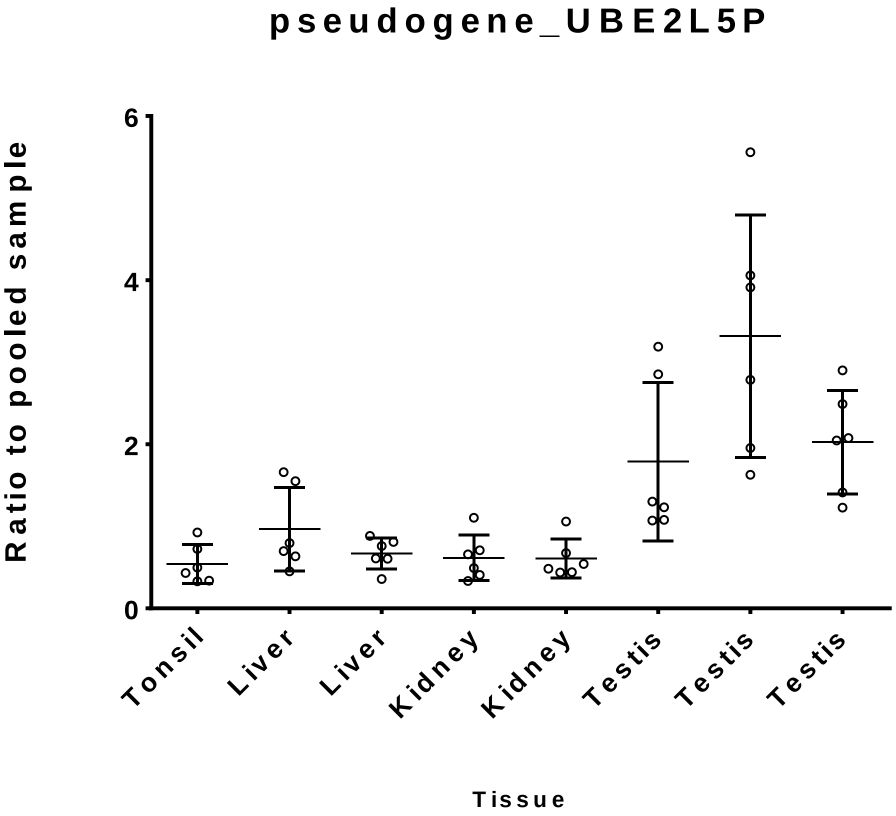
pseudogene_UBE2L5P
Ratio to pooled sample
0
2
4
6
Tonsil
Liver
Liver
Kidney
Kidney
Testis
Testis
Testis
Tissue

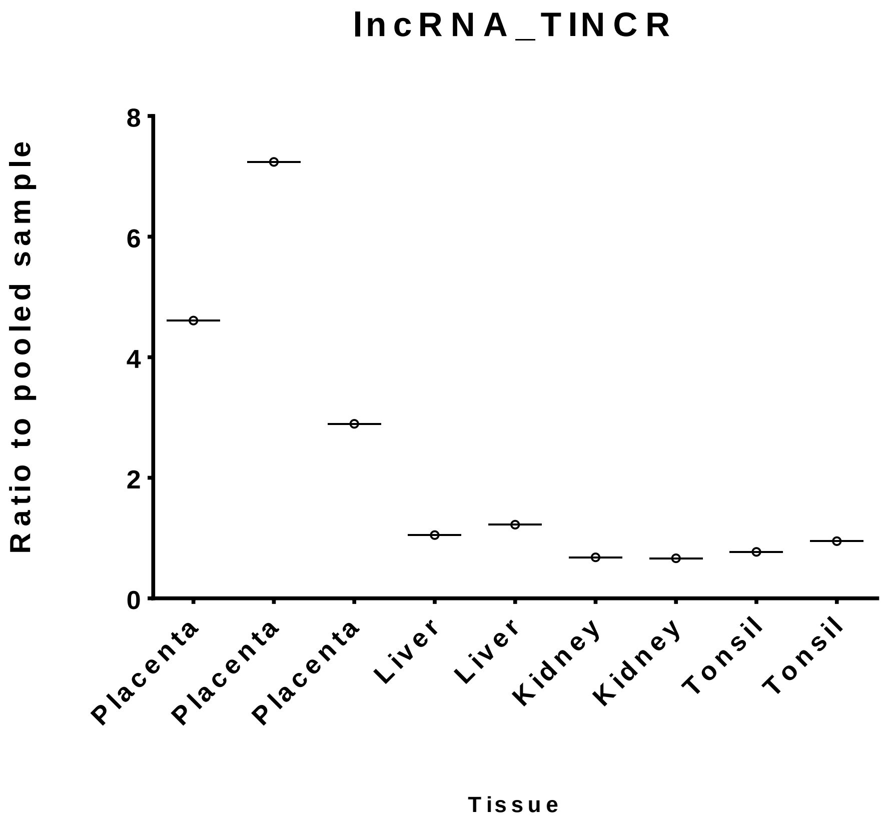
lncRNA_TINCR
Ratio to pooled sample
8
6
4
2
0
Placenta
Placenta
Placenta
Liver
Liver
Kidney
Kidney
Tonsil
Tonsil
Tissue

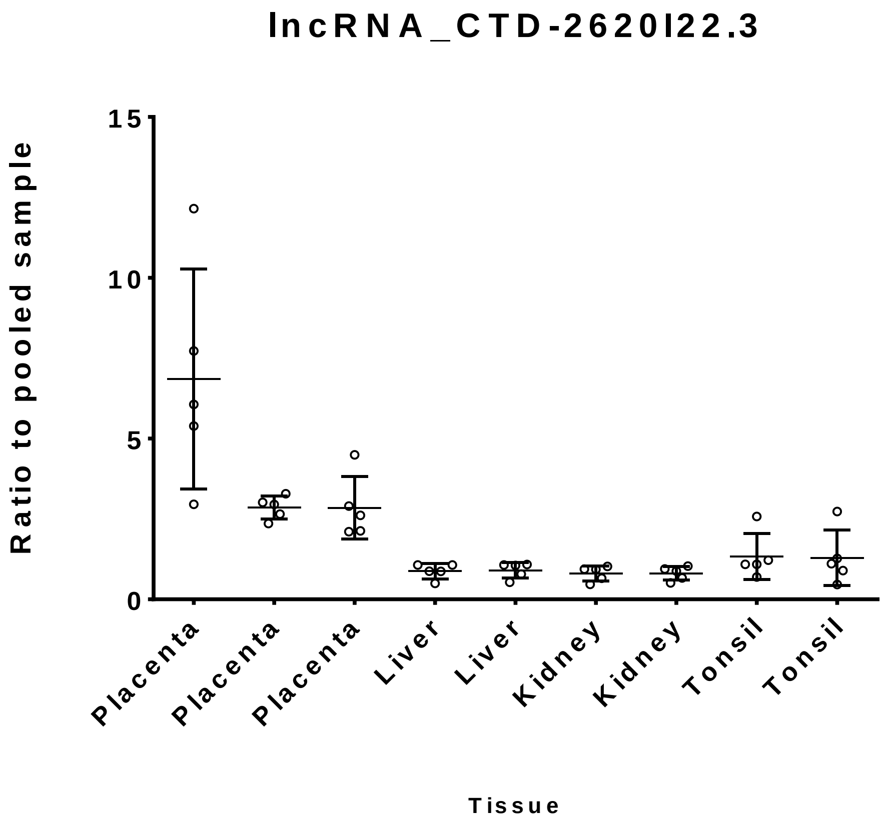
lncRNA_CTD-2620I22.3
Ratio to pooled sample
15
10
5
0
Placenta
Placenta
Placenta
Liver
Liver
Kidney
Kidney
Tonsil
Tonsil
Tissue

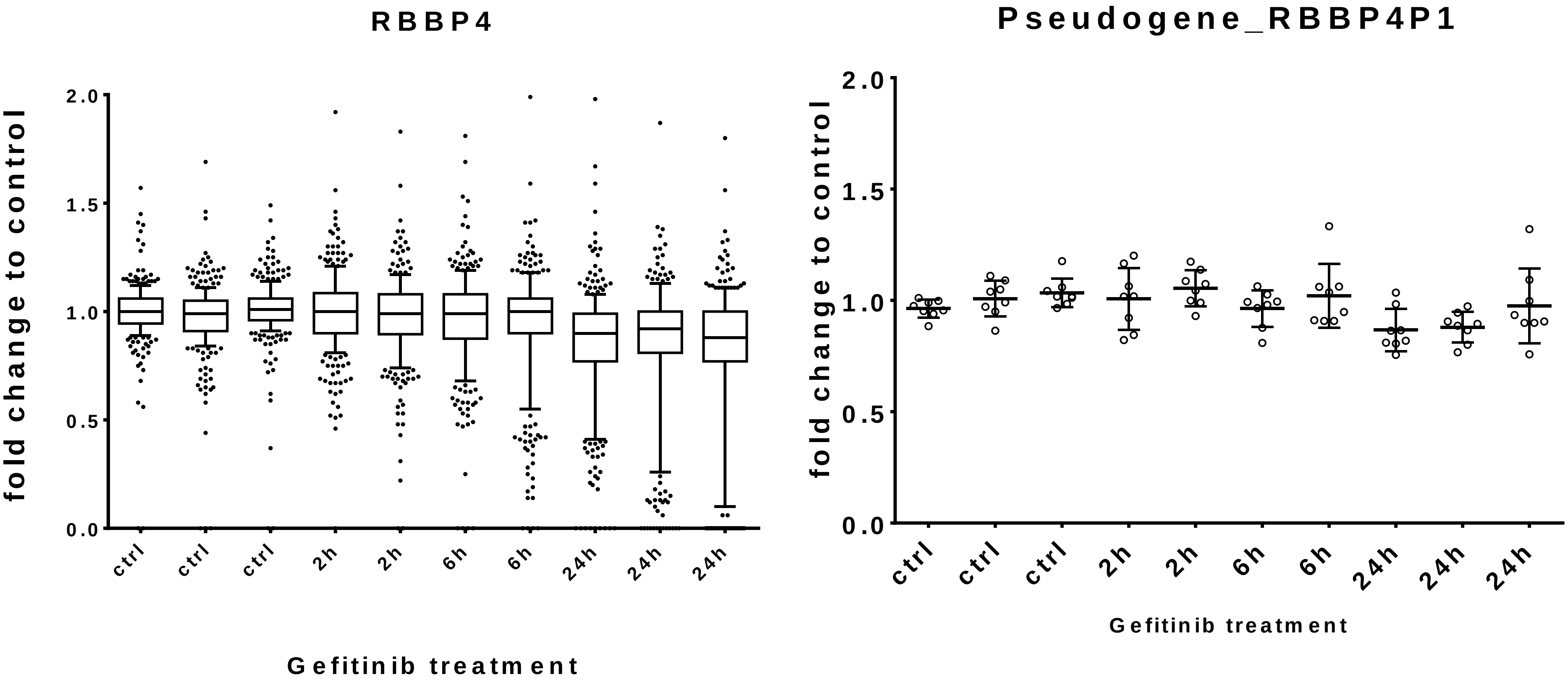

RBBP4
fold change to control
2.0
1.5
1.0
0.5
0.0
ctrl
ctrl
ctrl
2h
2h
6h
6h
24h
24h
24h
Gefitinib treatment
Pseudogene_RBBP4P1
fold change to control
2.0
1.5
1.0
0.5
0.0
ctrl
ctrl
ctrl
2h
2h
6h
6h
24h
24h
24h
Gefitinib treatment

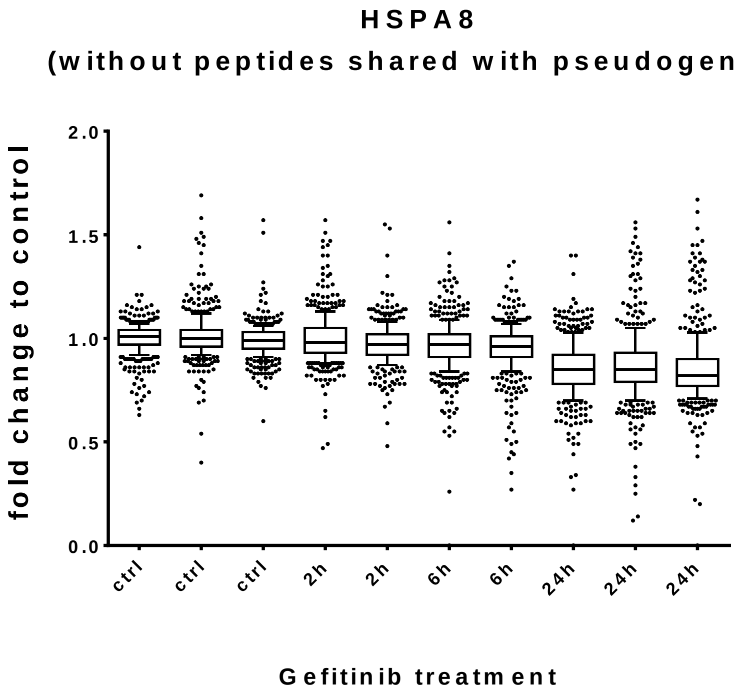
HSPA8
(without peptides shared with pseudogenes)
fold change to control
2.0
1.5
1.0
0.5
0.0
ctrl
ctrl
ctrl
2h
2h
6h
6h
24h
24h
24h
Gefitinib treatment

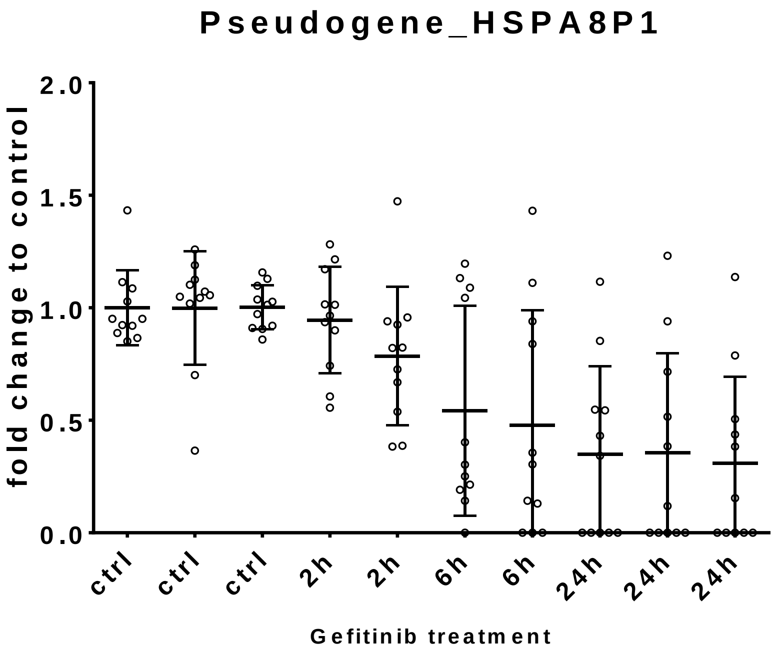
Pseudogene_HSPA8P1
fold change to control
2.0
1.5
1.0
0.5
0.0
ctrl
ctrl
ctrl
2h
2h
6h
6h
24h
24h
24h
Gefitinib treatment

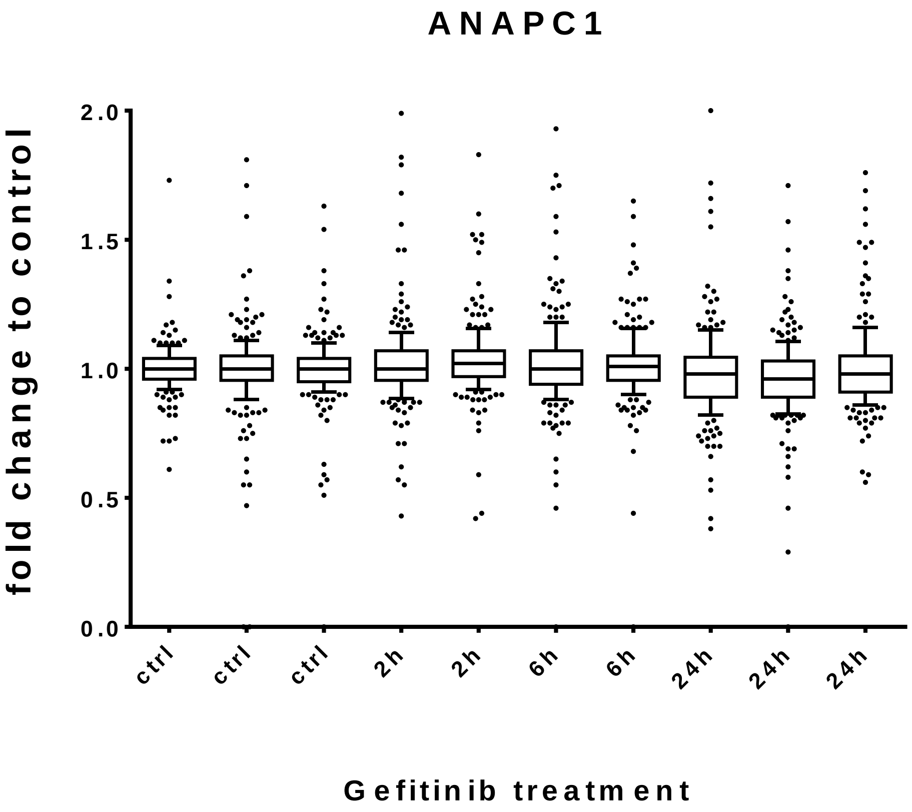

ANAPC1
fold change to control
2.0
1.5
1.0
0.5
0.0
ctrl
ctrl
ctrl
2h
2h
6h
6h
24h
24h
24h
Gefitinib treatment

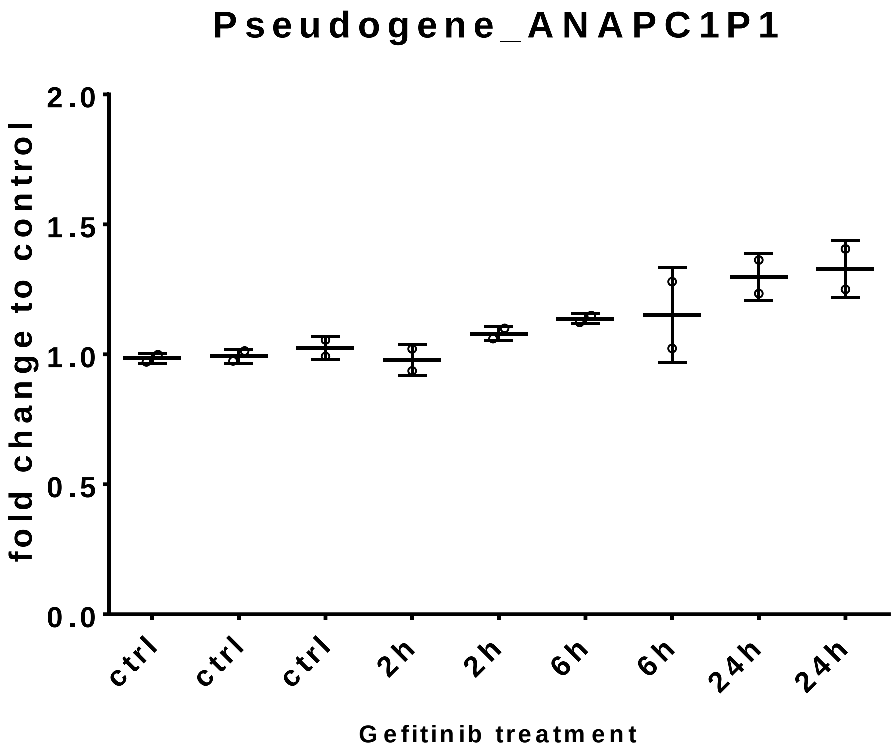

Pseudogene_ANAPC1P1
fold change to control
2.0
1.5
1.0
0.5
0.0
ctrl
ctrl
ctrl
2h
2h
6h
6h
24h
24h
Gefitinib treatment

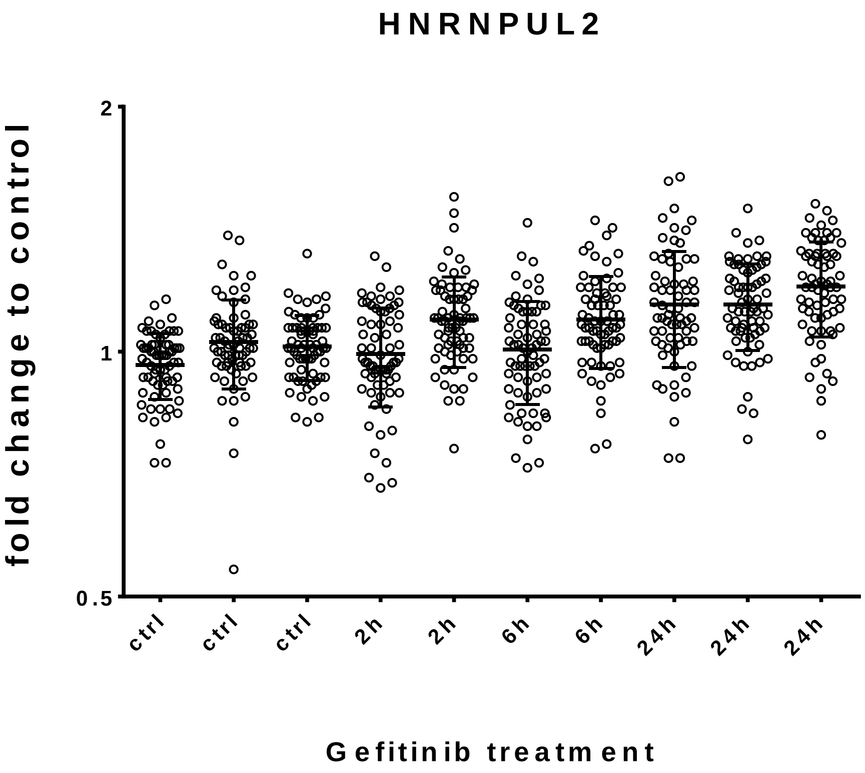
HNRNPUL2
fold change to control
2
1
0.5
ctrl
ctrl
ctrl
2h
2h
6h
6h
24h
24h
24h
Gefitinib treatment

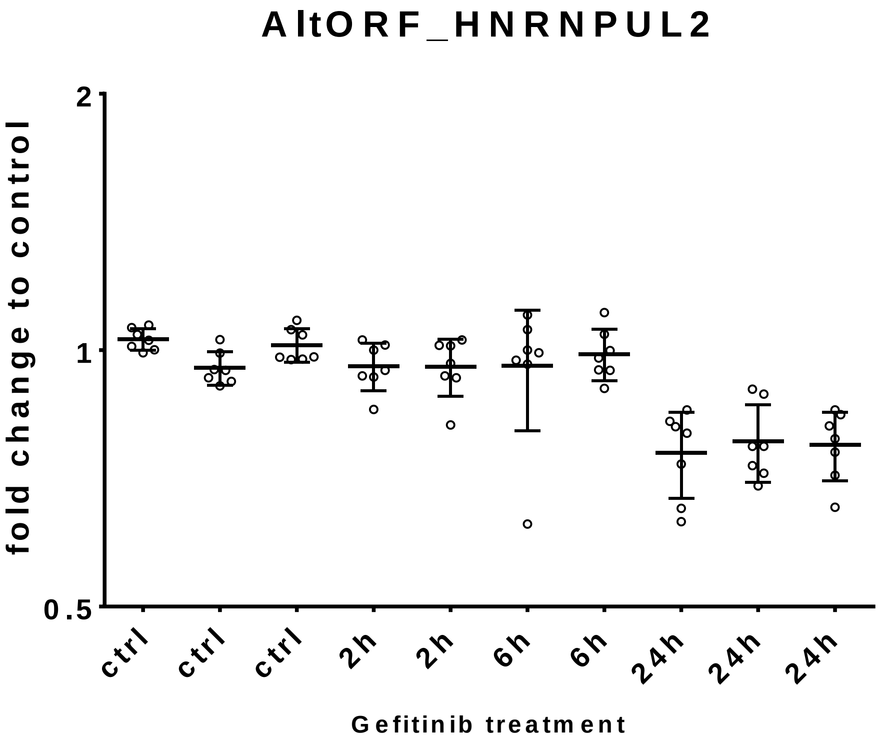
AltORF_HNRNPUL2
fold change to control
2
1
0.5
ctrl
ctrl
ctrl
2h
2h
6h
6h
24h
24h
24h
Gefitinib treatment

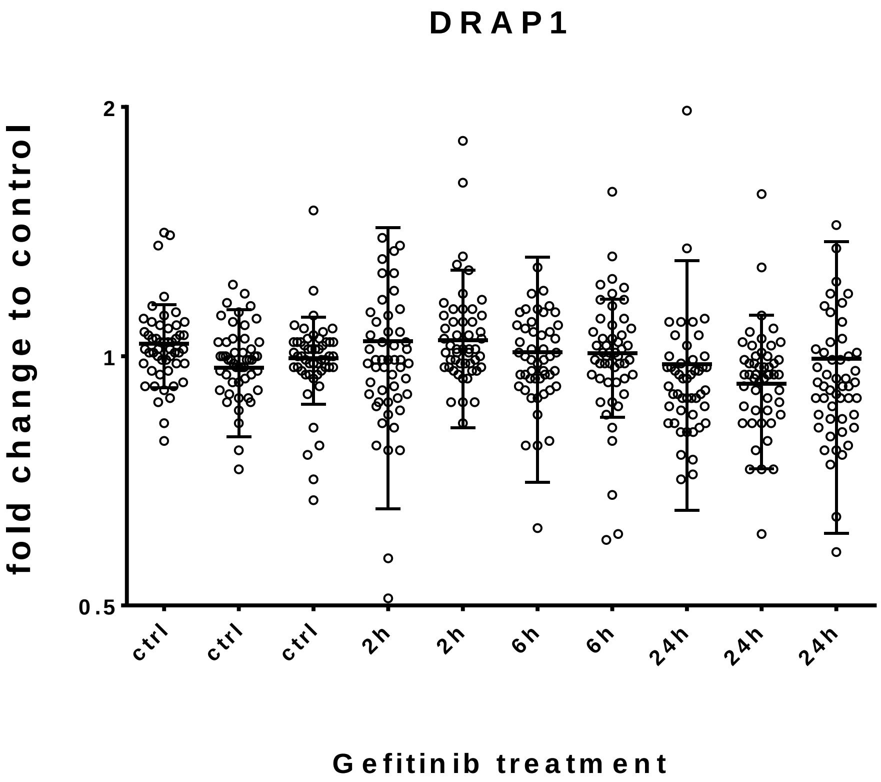
DRAP1
fold change to control
2
1
0.5
ctrl
ctrl
ctrl
2h
2h
6h
6h
24h
24h
24h
Gefitinib treatment

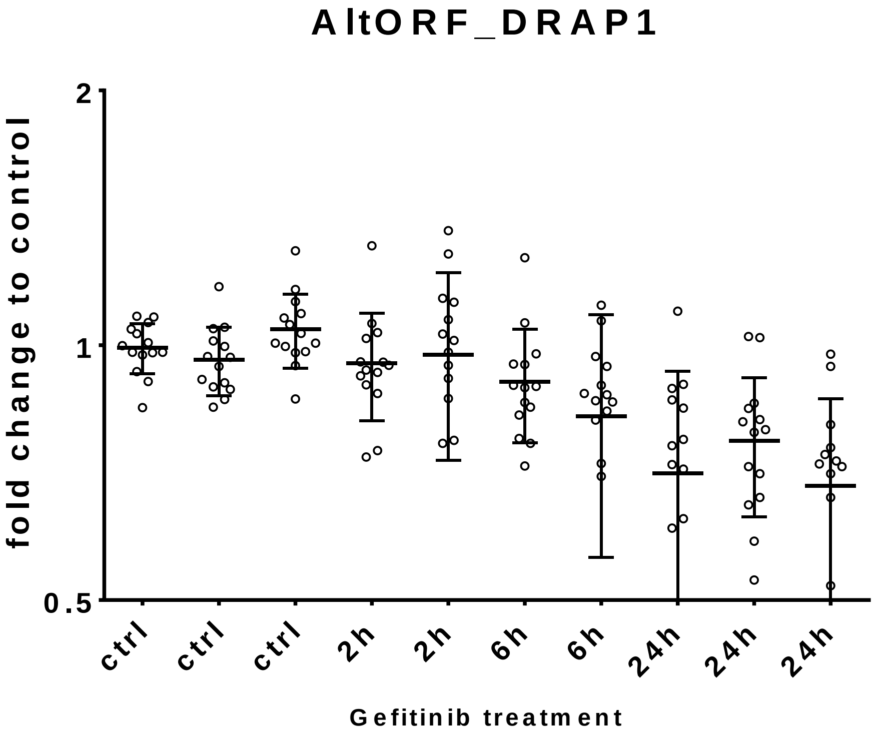
AltORF_DRAP1
fold change to control
2
1
0.5
ctrl
ctrl
ctrl
2h
2h
6h
6h
24h
24h
24h
Gefitinib treatment

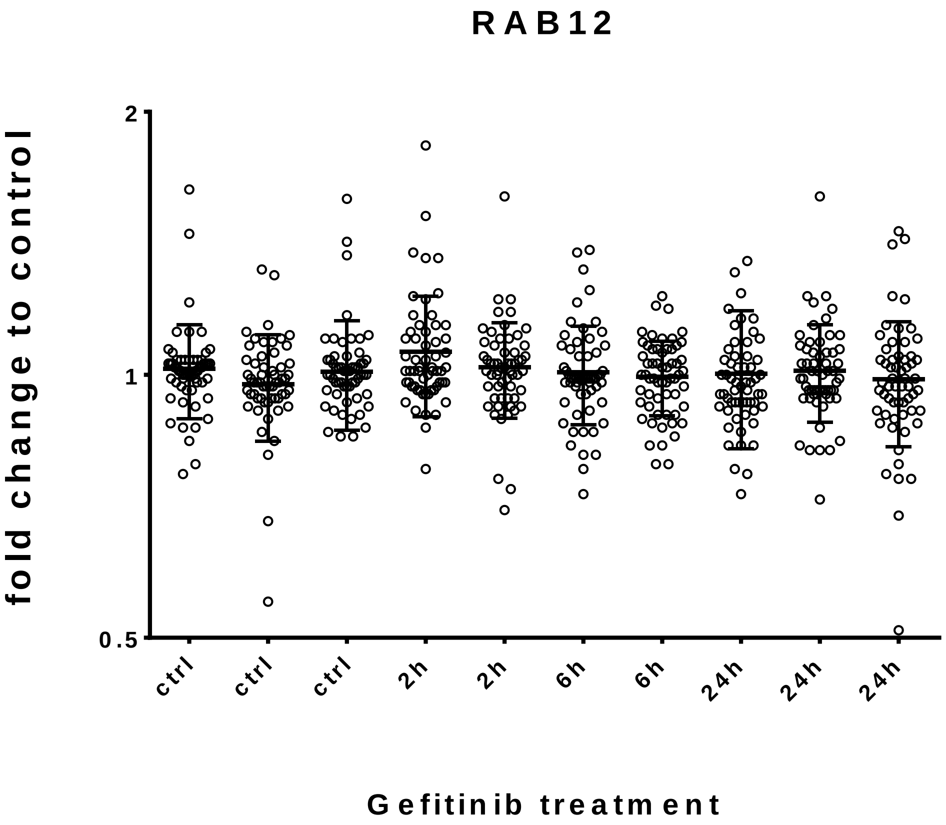
RAB12
fold change to control
2
1
0.5
ctrl
ctrl
ctrl
2h
2h
6h
6h
24h
24h
24h
Gefitinib treatment

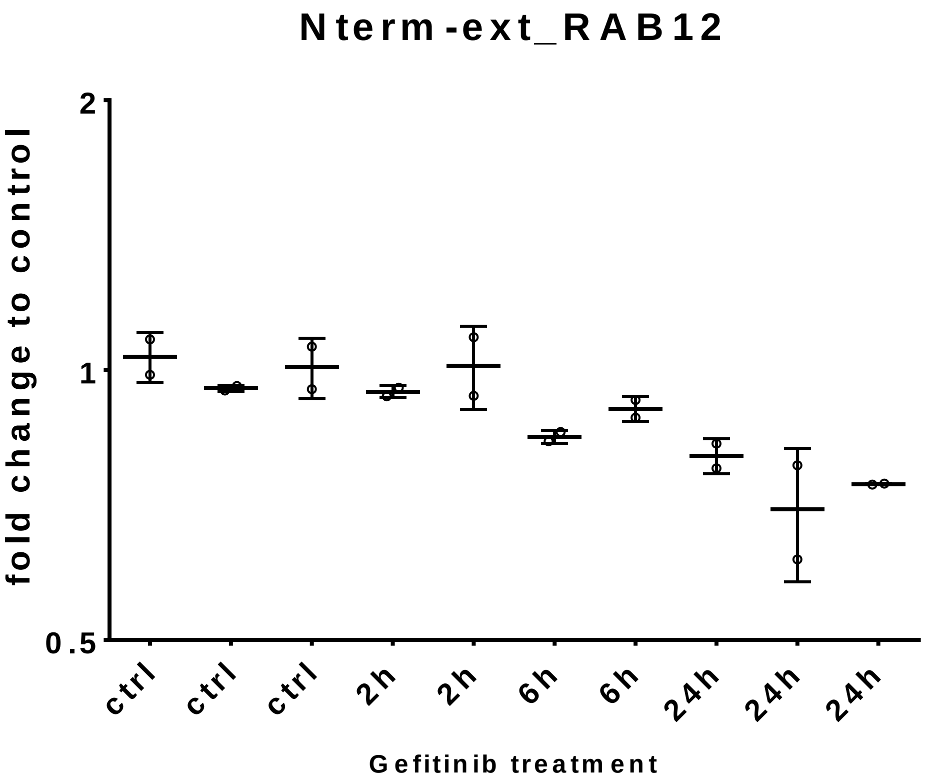
Nterm-ext_RAB12
fold change to control
2
1
0.5
ctrl
ctrl
ctrl
2h
2h
6h
6h
24h
24h
24h
Gefitinib treatment

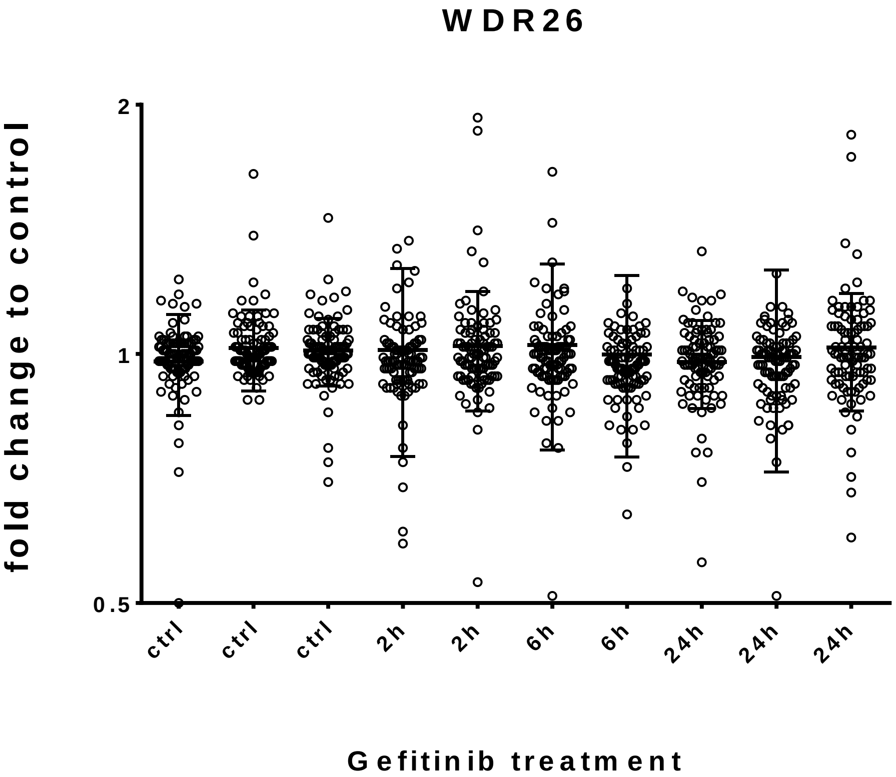

WDR26
fold change to control
2
1
0.5
ctrl
ctrl
ctrl
2h
2h
6h
6h
24h
24h
24h
Gefitinib treatment

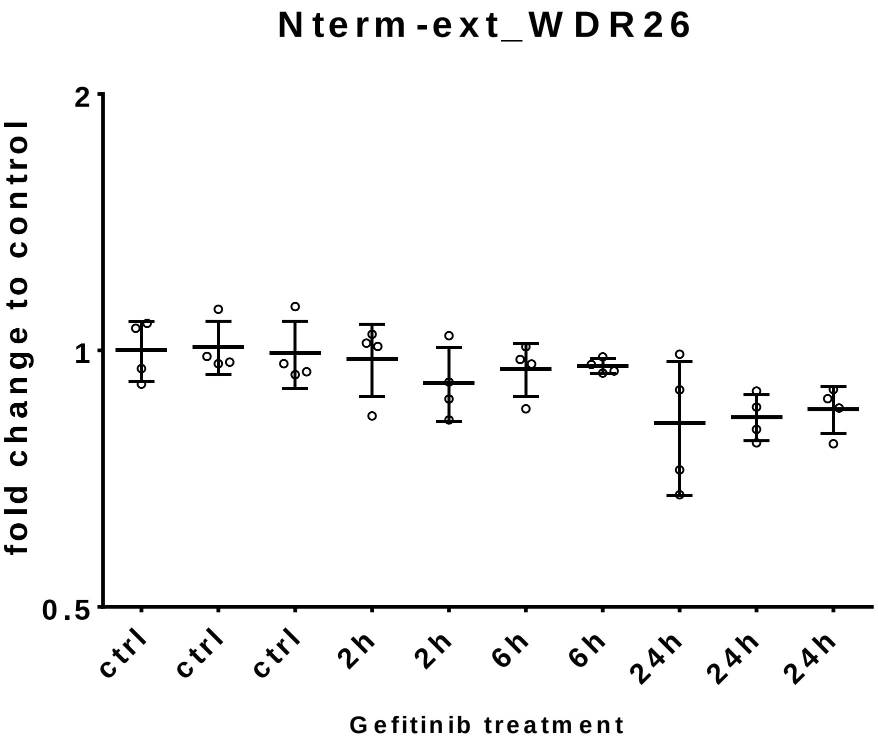

Nterm-ext_WDR26
fold change to control
2
1
0.5
ctrl
ctrl
ctrl
2h
2h
6h
6h
24h
24h
24h
Gefitinib treatment

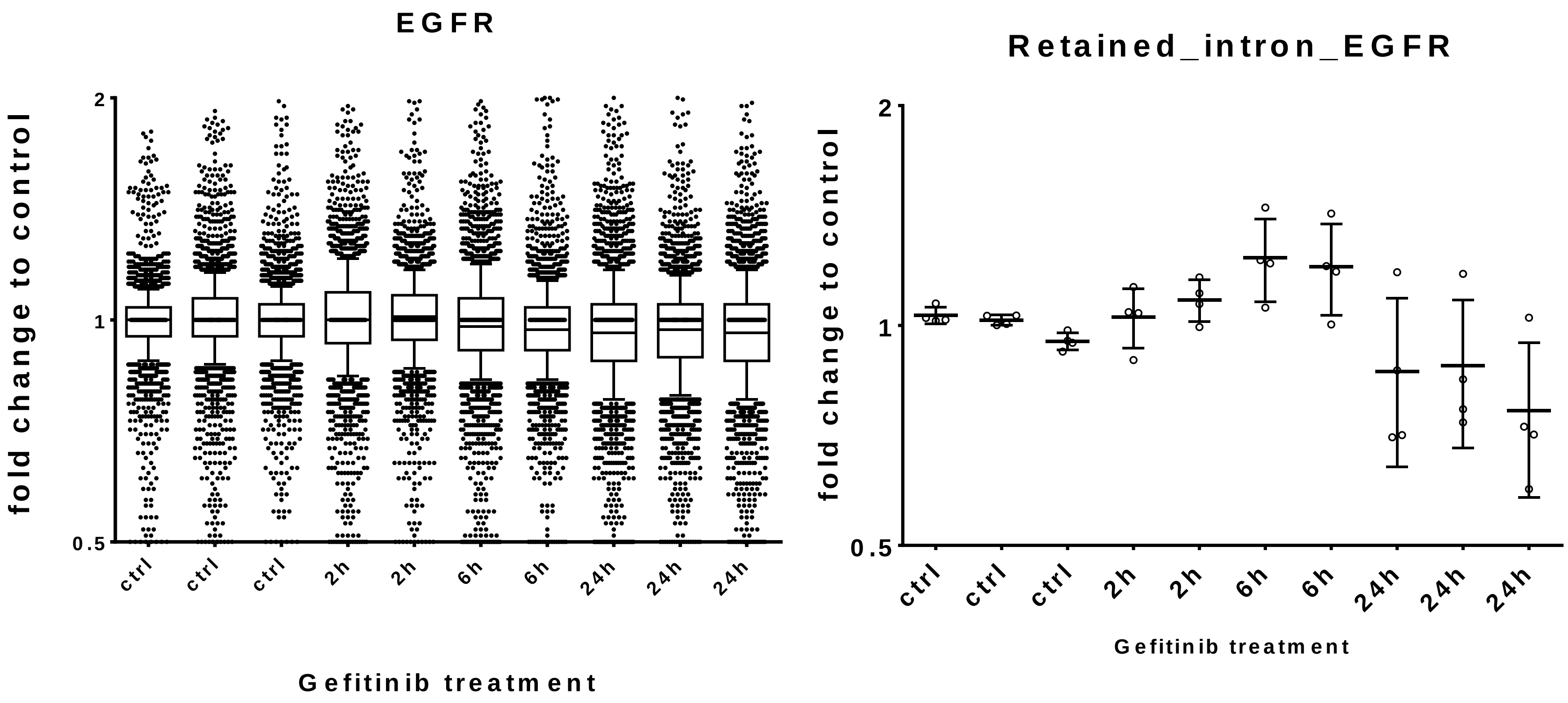

EGFR
fold change to control
2
1
0.5
ctrl
ctrl
ctrl
2h
2h
6h
6h
24h
24h
24h
Gefitinib treatment
Retained_intron_EGFR
fold change to control
2
1
0.5
ctrl
ctrl
ctrl
2h
2h
6h
6h
24h
24h
24h
Gefitinib treatment
